# Identification, design, and *in vivo* proof of concept of a shared APC neoantigen delivered via a self-amplifying RNA containing virus-like nanoparticle for cancer vaccination

**DOI:** 10.3389/fimmu.2026.1810178

**Published:** 2026-06-04

**Authors:** Alfred J. Simmons, Anna S. Nikonova, Chloe D. Jonas, Katherine A. Pniewski, Deepak Upreti, Maria I. Perez, Satish Adhikari

**Affiliations:** 1 Chimeron Bio Corporation, Philadelphia, PA, United States; 2 Nanotherapeutics Research Laboratory, Department of Biochemistry and Molecular Biology, University of Miami Miller School of Medicine, Miami, FL, United States

**Keywords:** adenomatous polyposis coli, cancer vaccine, colorectal cancer, familial adenomatous polyposis, self-amplifying RNA, virus-like nanoparticle

## Abstract

Colorectal Cancer (CRC) accounts for the second highest number of cancer-related mortalities rate worldwide, and its incidence and mortality is expected to continue to increase in the coming years. Over 80% of CRC cases are caused by mutation in the Adenomatous Polyposis Coli (APC) gene, a tumor suppressor gene involved in the Wnt signaling pathway that prevents uncontrolled cell growth. Familial Adenomatous Polyposis (FAP) is a rare, inherited, pediatric disorder characterized by the development of countless adenomatous (benign) polyps in the colon and/or rectum, and over the patient’s lifetime there is an almost 100% likelihood that the disorder will progress into CRC. In this study, we utilized bioinformatics to identify shared neoantigen epitopes present at the mutational cluster region (MCR) of the APC gene of multiple CRC patients, where FAP-associated mutation is typically observed. We then developed and optimized a chimeric virus-like particle (VLP) to encapsulate an saRNA replicon encoding a neoantigen construct featuring the identified mutated APC sequences inserted into a WT APC backbone. T-cell specific activation and restimulation against neoantigen peptide fragments were observed *in vitro*, indicating the capacity for a T-cell mediated response *in vivo*. Serum samples from intramuscularly-dosed BALB/c mice exhibited significant neoantigen-specific IgG titers compared against vehicle control and WT APC control mice, confirming VLP-expression of neoantigens and indicating neoantigen-specific *in vivo* immune activity. The significant response observed against the nanoparticle-expressed APC neoantigen serves as proof-of-concept for a saRNA-expressed cancer vaccine against APC-associated CRC.

## 1 Introduction

Colorectal cancer (CRC), both hereditary and sporadic, is the third most common cancer in the US, with an estimated number of 154,270 new cases and 52,900 deaths in 2025 (1). In most cases, these cancers arise because of hyperactivation of the Wnt pathway (>95% CRCs), which is mostly caused by inactivation of the Adenomatous Polyposis Coli (APC) gene (>80% CRCs), a tumor suppressor gene involved in Wnt Signaling pathway that prevents uncontrolled cell growth (2–4). Familial Adenomatous Polyposis (FAP) is a rare, inherited, pediatric CRC syndrome characterized by the formation of numerous polyps in the colon and rectum. This disorder is caused by germline mutation of the APC gene (5, 6). Once the mutated APC gene is inherited by a child, the likelihood of another spontaneous mutation in the second (normal) APC gene throughout the lifetime is close to 100%, leading to an almost 100% lifetime risk of developing CRC (6). Once both alleles of APC are mutated, cell growth is not controlled properly, which leads to the development of hundreds to thousands of polyps around the colon and/or rectum. With this in mind, vaccines targeting the APC gene represent a promising preventative approach for FAP and CRC in general.

**Figure 1:**
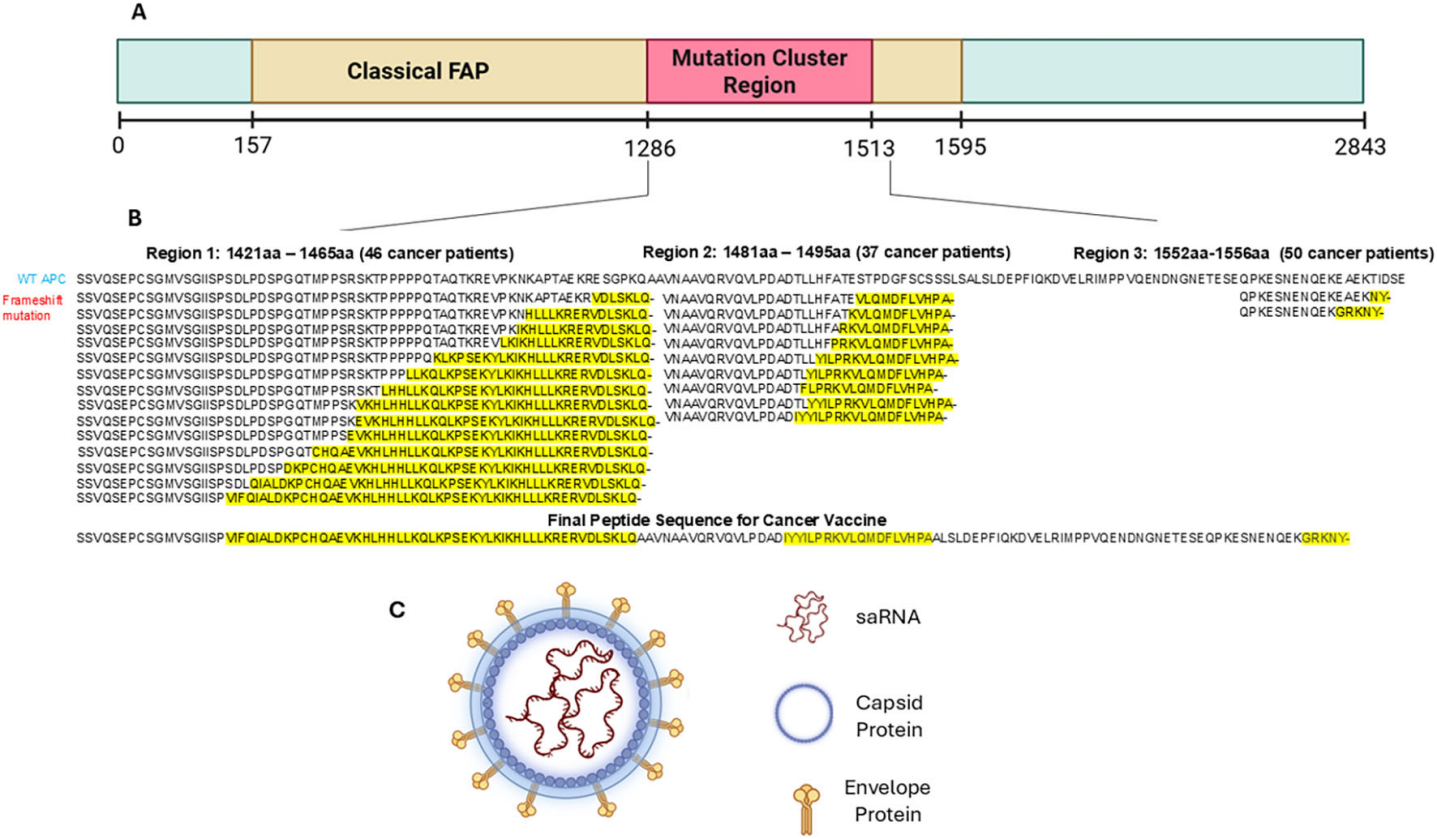
Bioinformatics analysis and identification of conserved APC mutations. **(A)** Different forms of Familial Adenomatous Polyposis (FAP) and its correlation with APC gene mutation position. Classical FAP is the most severe form (>100 polyps), and most mutations are found the middle region of APC gene, mostly at the MCR between 1250 aa and 1550 aa. Most mutations are nonsense mutation (stop gained) or frameshift mutation which ultimately leads to truncated APC protein. **(B)** Within MCR of APC gene, we have identified 3 regions that result in frameshift mutation leading to synthesis of new amino acids and ultimately ending in a stop codon. In addition, such frameshift mutations are not unique to a single patient but are instead shared among different patients (133 patients). This shared expression makes these excellent targets for a cancer vaccine. Our neoantigen combined all three regions and expresses the mutated APC protein as a cancer vaccine. **(C)** Composition of the nanoparticle vector used to deliver saRNA encoding the APC mutant GOI in this study features a chimeric design comprised of a viral envelope glycoprotein pseudotype, capsid protein, and saRNA replicase, which serves to deliver and amplify encoded RNA. **Figure 1C** was created using BioRender.

APC mutation frequently leads to cancer, and its severity depends largely on the location of the mutation within the APC sequence (5–7). The mutations that we have selected in this study are frameshift mutations (**Figures 1A, B**) that lead to synthesis of completely different amino acids downstream of the mutation that are not found in the normal (WT) protein and ultimately end in a stop codon. In addition, unlike other tumor suppressor genes, truncated APC is still expressed in tumor cells and may be necessary for tumor proliferation, particularly for severe forms of FAP (8–14). OncoDB database shows that APC gene expression is only 1.5-fold lower on average in colon tissues (n = 308) compared to normal tissues (n = 41) in patients with colon adenoma cancer. In fact, many of those patients had 2- to 5-fold higher expression of the mutated APC gene, making APC an excellent target for a cancer vaccine. Similarly, it has previously been demonstrated that truncated APC is positively selected in cancer cells and important for cancer cell survival, particularly if that truncation occurs around MCR and leads to a severe form of FAP (8–10). Shorter APC mutant sequences found within the neoantigen sequence developed and tested in this study have previously been shown to elicit robust T-cell mediated responses *in vitro*, suggesting a high likelihood of such a response to the expanded neoantigen sequence tested herein (15, 16).

Clinical manifestation of FAP is characterized by the formation of adenomas in the rectum and colon, with the most severe symptoms being observed in Classical FAP (7). These adenomas are initially benign and asymptomatic for several years, but genomic instability and uncontrolled growth inside such tumors ultimately leads to malignancy. Mortality in the case of FAP is primarily caused by adenocarcinoma development or desmoid tumor obstruction of blood vessels and nerves (17). At present, there is no treatment for this disorder, and management and delay of cancer development are the primary clinical approaches to FAP (6).

The feasibility of vaccination against FAP-associated CRC requires delivery of one or more neoantigens to stimulate robust immune recognition and response. As broad improvements have been made to their efficacy, safety, and expression capacity, nanoparticle constructs such as liposomes, self-assembling protein nanoparticles, and virus-like nanoparticles (VLPs) have gained increasing interest in the field of vaccines and immunology (18). Many such nanoparticle vectors are utilized for delivery of an mRNA payload, which in turn expresses and secretes an antigen of interest from transduced cells after dosing. This approach eliminates the risk of integration into the host cell genome that is commonly associated with DNA-based vaccines while also maintaining a simple, inexpensive, and rapid production process relative to traditional vaccine approaches (19). Moreover, implementation of alphavirus replicon elements into the mRNA vaccine approach facilitates self-amplification of mRNA following transduction into the host cell, thereby significantly increasing the antigenic titer and durability of expression from the vaccine platform (19, 20).

The virus-like nanoparticle platform utilized in this study is a biosynthetic, self-assembling delivery vector that is characterized by modified viral glycoproteins encasing a self-amplifying mRNA (saRNA) encoding the protein(s) of interest (POI) (**Figure 1C**). This approach differs from the more common lipid nanoparticle (LNP) vector approach used for saRNA vaccines and facilitates comparable POI expression and antigen-specific immunogenicity at a significantly lower dose (21–23). The particle is a non-lipid platform that amplifies expression and translation of an mRNA gene of interest (GOI) after transduction into host cells via the activity of a replicase protein encoded in the replicon sequence. The amplified expression subsequently produces high POI expression from a low initial dose. The durable protein expression and localization observed from IM dosing of saRNA vectors suggests vaccine dosing would likely require fewer repeated doses compared to recombinant protein or traditional mRNA vaccination methods, which typically exhibit shorter POI expression durability (19).

In this study, we identified multiple shared neoantigens observed within the mutational cluster region (MCR) of CRC patients where FAP-associated mutations are most commonly observed. We then designed a mutant APC protein with mutant regions inserted into a truncated WT APC backbone. This neoantigen was encoded in the saRNA contained within a VLP nanoparticle platform and was dosed into BALB/c mice for *in vivo* efficacy screening. A significant T-cell mediated response against the neoantigen was observed *in vitro* via PBMC restimulation assay, and we observed a significant humoral immune response against the mutant APC construct compared with vehicle and WT APC controls *in vivo*, indicating neoantigen-specific immune stimulation. Antigen-specific serum IgG1 responses have previously been shown to correlate with T-cell mediated responses *in vivo*, which is believed to be a result of cross priming of humoral and T-cell mediated immunity (24). This correlation and the significant neoantigen-specific response observed in this study suggest a high likelihood for T-cell activity in response to the APC neoantigen designed in this study. The data presented herein supports the efficacy of a novel neoantigen delivered via saRNA virus-like particle for vaccination against APC-associated CRC.

## 2 Methods

### 2.1 Bioinformatics analysis

Identification of shared APC mutants was performed via manual querying of the National Cancer Institute Genomic Data Commons database for frameshift mutations in APC proximal to the MCR (between aa1286 and aa1556) as observed in CRC patients. Translational analysis of the APC mutations was performed by comparing the WT APC amino acid sequence to the translated frameshift mutants (Expasy). Frameshift mutations resulting in common neoantigen peptide sequences were flagged for inclusion in the final neoantigen sequence, which ultimately included three “hot spot” mutant regions (**Supplementary Table 3**). These regions were inserted into a truncated WT APC backbone to ensure optimal expression and targeting of neoantigens.

### 2.2 saRNA expression plasmid design and cloning

Plasmid expressing Venezuelan Equine Encephalitis Virus (VEEV) saRNA was constructed by expressing it from CMV promoter. All the structural proteins of the virus were removed and replaced by gene encoding GFP. For expressing proteins of interest (**Supplementary Figure 1A**), GFP was replaced by genes encoding those proteins. Gblocks (double stranded gene fragments) were ordered from IDT. NEBuilder^®^ HiFi DNA Assembly kit was used from cloning. The sequences were verified by DNA sequencing (Azenta).

### 2.3 Nanoparticle production

Chimeric VLPs were produced via PEI-mediated transient transfection of modified HEK293 production cells (Thermo Fisher), which has been previously described, using three plasmids encoding the constituent envelope, capsid, and saRNA elements of the nanoparticle (25). Production cells were treated with lithium acetate, sodium propionate, and Ruxolitinib phosphate (Apex Bio) transfection enhancers during the transfection process to improve nanoparticle yield and reduce viral protein recognition. The self-assembling nanoparticles were harvested after 48 hours from the production cell supernatants, filtered to remove cellular debris, and concentrated via PEG-based centrifugation. Particles were purified using a resin-based batch-binding method that has previously been described (26). Following concentration and purification, nanoparticles were dialyzed to remove PEG and other remaining impurities.

### 2.4 Nanoparticle quantification

Following production and harvest, nanoparticles were quantified via digital PCR analysis of nanoparticle samples. Nanoparticle samples were lysed and RNA was isolated via MagMax Mirvana Total RNA Isolation kit (Thermo Fisher). The isolated RNA was converted to cDNA via Maxima H Minus cDNA Synthesis kit (Thermo Fisher), and the replicon-specific cDNA was quantified via digital PCR. The measured DNA concentrations were converted to replicon-specific RNA concentrations within particle samples, which was subsequently used in calculating dose concentrations.

### 2.5 *In vitro* nanoparticle transduction

Nanoparticle constructs were transduced in duplicate into BHK21 cells at 1:1000 volumetric ratio in 2 mL cell suspension containing 2.5E5 cells/well in a 6-well cell culture plate. Cells were incubated at 37 °C and 5% CO2 for 48 hours. Following incubation, cell supernatants were collected and centrifuged at 500 x g for 5 minutes to remove residual cell debris. Supernatants were then stored at -80 °C prior to analysis via ELISA.

### 2.6 Neoantigen expression ELISA

Harvested transduction supernatants were assessed for neoantigen expression relative to untransduced controls. High protein binding ELISA plates were coated with anti-C-tag conjugate antibody (Thermo Fisher) and incubated overnight at 4 °C, then blocked the following day with 1% bovine serum albumin in PBS (blocking buffer). Harvested transduction supernatants were serially diluted in blocking buffer, added to the plate, and incubated for 90 minutes at room temperature. Anti-APC OTI1B12 monoclonal antibody (Origene) was diluted 1:1000 in blocking buffer and added to each well to serve as a primary antibody, incubating for 1 hour at room temperature. Following primary antibody incubation, HRP-conjugated goat anti-mouse IgG secondary antibody (Thermo Fisher) was diluted 1:15,000 in blocking buffer and added to wells and incubated for 1 hour at room temperature. OD450 was measured and fold-change analysis of transduced cell supernatants over untransduced control supernatants was performed. Where necessary for construct comparison, fold-change analysis results were normalized to particle RNA concentrations.

### 2.7 T cell stimulation/restimulation assay

The procedure for stimulation and restimulation of T cells within a PBMC suspension and subsequent harvest of supernatants for IFN-γ has been previously described (27). PBMCs from multiple donors (IQ Biosciences, iXCells Biotechnology) were used to confirm responses between patients.

After harvest, cell supernatants were assessed for IFN-γ concentrations via human IFN-γ ELISA kit (Thermo Fisher), which was performed as described in the manufacturer’s instructions.

At time of restimulation, a set of PBMCs were treated with monensin solution (BioLegend) to block cytokine secretion from stimulated cells. T cells were fluorescently labeled with an anti-CD3 antibody (Thermo Fisher Scientific) prior to fixation and permeabilization. Intracellular IFN-γ was then labeled using a PE-conjugated anti-IFN-γ antibody (BioLegend) and cells were analyzed via flow cytometer to identify the specific PBMC populations responsible for IFN-γ secretion following restimulation.

### 2.8 Antigen-specific IgG ELISA

Harvested mouse serum samples were assessed for antigen-specific IgG titer via ELISA. Plates were coated with anti-C-tag conjugate antibody (Thermo Fisher) and incubated overnight at 4°C, then blocked the following day with 1% bovine serum albumin in PBS (blocking buffer). Nanoparticle-expressed neoantigens containing an EPEA C-terminal tag were bound by the capture antibody and incubated for 90 minutes at room temperature. Serial dilutions of treated mouse serum samples were prepared in blocking buffer. Anti-APC OTI1B12 monoclonal antibody (Origene) reference standard curves were prepared for interpolation of neoantigen-specific IgG in serum samples. Samples and reference standards were added to the plate in duplicate and incubated for 1 hour at room temperature. HRP-conjugated goat anti-mouse IgG secondary antibody was prepared at 1:15,000 dilution. Secondary antibody was added to the plate and incubated for 1 hour at room temperature. OD450 was measured and a 4-parameter logistic curve was calculated using the values reported for the reference standards. Test article concentrations were interpolated using the reference standard regression and fold-change analysis over control arms was performed.

## 3 Results

An ideal antigen for a cancer vaccine should fulfill three criteria: 1) antigen(s) should be mutated forms of a protein that is likely to be recognized as a foreign antigen (neoantigen) by immune cells; 2) antigen(s) should be shared among a group of patients (shared neoantigen) in order to be a cost effective target; and 3) antigen(s) should only be expressed in cancer cells (28–30). To this point, most antigens that are used for cancer vaccines do not fulfill at least one of the above criteria. Most mutations in such proteins are either point mutations or stop-gained mutations that disrupt the protein function but are not recognized as foreign antigen, which typically results in these antigens failing to elicit a robust immune response. There are several algorithms to predict if a particular point mutation will be good enough to elicit an immune response, but such algorithms are far from perfect and useful only as a guide to rank potential neoantigens for *in vitro* work. Even if a suitable neoantigen is verified by *in vitro* assessment, such neoantigens are typically not shared among enough patients to be useful for a broad patient population (17). However, the APC gene that we present in this study as a neoantigen for a cancer vaccine fits all of the above parameters.

The different forms of mutated APC gene have been well characterized via genome sequencing of tumors isolated from the cancerous colorectal polyps of thousands of patients with FAP disorder, which allows for identification of common APC mutations that can serve as targets for a FAP-associated CRC cancer vaccine. Previous work has shown that a frame-shift mutation (S246Ffs6X) in APC gene was highly immunogenic and elicited an immune response, which supports the concept of an MHC pathway-derived vaccine approach (31). Targeting of these antigens provides a basis of establishing a robust immune response against FAP adenoma development, which thereby inhibits polyp formation and development without requiring surgical intervention. Additionally, due to the easily identified at-risk populations (first-degree relatives of individuals with FAP) and the limited development of the disorder prior to the second decade of life, a vaccine approach would establish an immune response against FAP antigens long before the age at which development of clinical symptoms is most common. Recent studies targeting APC neoepitopes in populations at-risk for FAP development have shown promising yet selective immune response against APC mutants (31). Investigation into other FAP-related vaccine targets has yielded noteworthy results, including reduced polyp burden via vaccine-based immune targeting of the ERBB3 receptor in mouse models (32). These findings suggest that vaccination against multiple FAP-related antigens could yield robust immune protection against polyp (and subsequently cancer) development in at-risk populations.

### 3.1 Identification and design of a FAP neoantigen

Due to FAP’s relatively low prevalence compared to other conditions associated with CRC development as well as the multitude of different APC point mutations that give rise to the condition, there is a notably limited amount of previous investigation into FAP/CRC vaccine development. With this in mind, development of an efficacious vaccine against FAP-associated CRC necessitated identification and design of a novel APC mutant neoantigen. A bioinformatics-driven approach was utilized to identify three regions within the MCR between 1250 aa and 1550 aa of the APC gene that result in frameshift mutations ultimately ending in a stop codon. More importantly, these frameshift mutations are not unique to a single patient but are instead shared among different patients (shared neoantigens). The frameshift mutations identified via CRC patient databases were conserved across as many as 133 out of 4767 patients that exhibited APC mutations in queried databases (**Figure 1B**). This shared expression makes these excellent targets for a cancer vaccine.

These identified sequences were encoded within the saRNA sequence of a nanoparticle to serve as the basis for the vaccine against FAP. Multiple secretory peptides were incorporated into the saRNA replicon sequence to facilitate secretion of neoantigens into the extracellular space following expression by host cells (**Supplementary Figure 1B**). Particles were manufactured, concentrated, and purified, and particle RNA concentrations were quantified via RNA isolation and subsequent dPCR analysis (**Supplementary Figure 1C**). Observing highest APCmut expression from pCBIO-1264, this construct was selected for use in the mouse *in vivo* study.

### 3.2 Assessment of neoantigen immunogenicity

Following identification and design of a mutant APC sequence containing multiple conserved mutations observed in CRC patients, an assessment of the capacity for these mutated regions of the protein to stimulate a T-cell mediated response *in vitro* was performed. Shorter neoantigen sequences within the regions identified and encoded in this study have previously been shown to elicit a robust T-cell mediated response *in vitro*, indicating a strong likelihood of a comparable response to the more expansive neoantigens tested here (15, 16).

**Figure 2:**
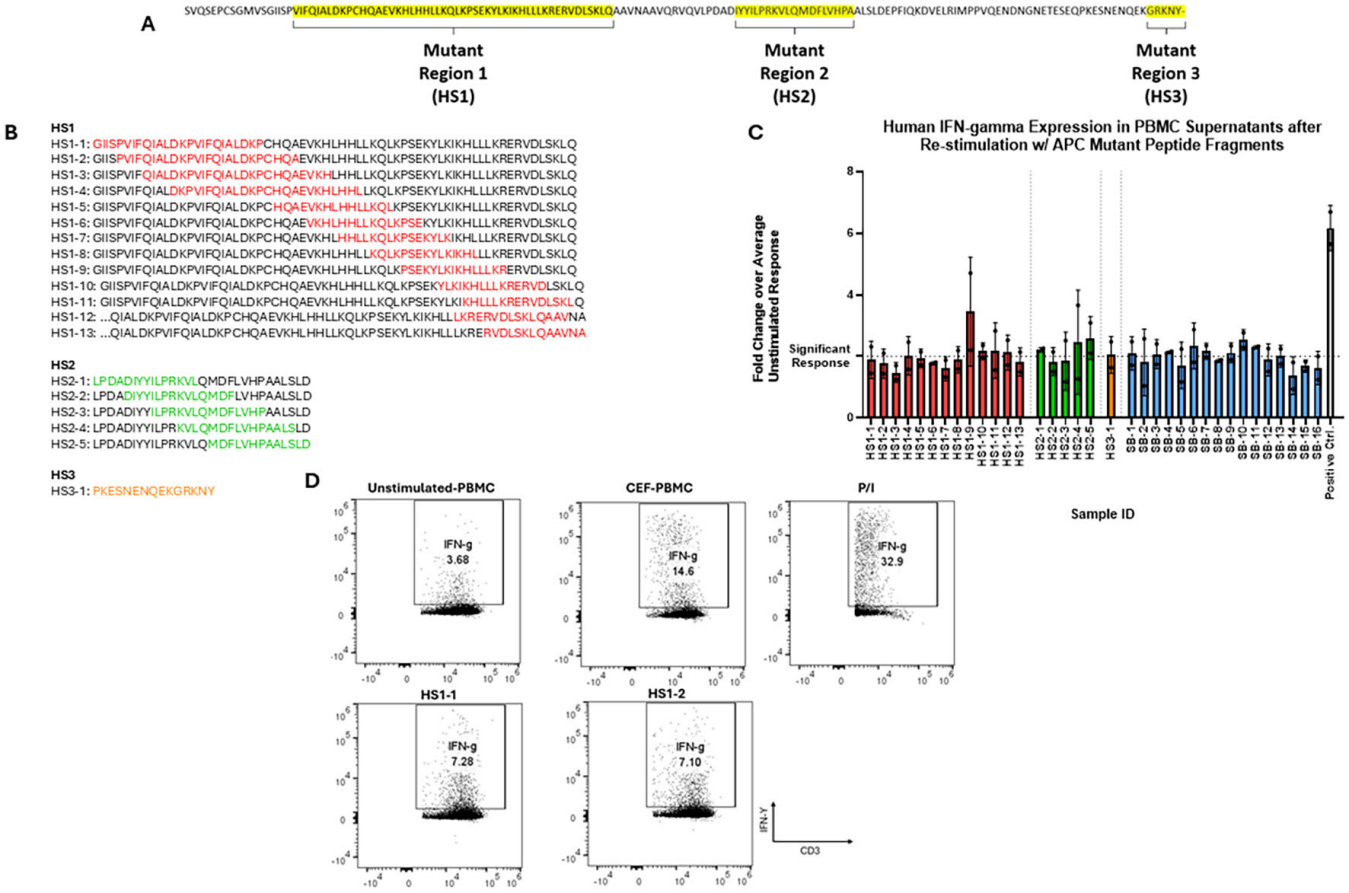
*In vitro* T-cell stimulation assay demonstrates capacity for neoantigen fragments to promote a T-cell mediated immune response. **(A)** The mutant APC neoantigen sequence features three mutant regions observed in CRC patient databases inserted into a WT APC backbone. These mutant regions were divided into short peptide fragments and assessed via bioinformatics sequence analysis for highest likelihood of APC presentation. **(B)** Each of the mutant region insertions were divided into overlapping peptide fragments (highlighted within the main sequence for the mutant region insert), and these fragments were synthesized to simulate possible immune presentation of the neoantigen *in vitro*. Joining the overlapping fragments of each mutant region, a series of fragments from the neoantigen identified via bioinformatics assessment as having the strongest MHC binding likelihood were synthesized and tested as well. **(C)** Human PBMCs were stimulated, activated, and re-activated with the 35 peptides with highest immunogenic likelihood derived from the mutated APC regions expressed in the APC mutant nanoparticle. Supernatant IFN-γ expression was subsequently measured via ELISA. Elevated IFN-γ concentrations (above unstimulated levels) were observed for peptide fragments derived from all three mutated APC regions, indicating antigen presentation and immune stimulation in response to the mutated epitopes. Of the 35 peptide fragments tested, 29 fragments exhibited significant response (defined as IFN-gamma concentration >2-fold greater than unstimulated) for one or more PBMC donors tested. A CEF control pool of 32 peptides derived from human Cytomegalovirus, Epstein-Barr Virus, and Influenza Virus was used as a positive control to demonstrate a robust response against recognized antigens. **D)** To assess intracellular expression of IFN-γ following restimulation with peptides, PBMCs were treated with monensin to block cytokine secretion. Cells were labeled with anti-CD3 surface markers, then fixed, permeabilized, and stained for intracellular IFN-γ, which demonstrated that T cells within the PBMC population were responsible for the expression and secretion of IFN-γ after restimulation with peptides.

Bioinformatics services (DTU Health Services) were utilized to identify fragments of the mutated APC regions with the highest likelihood to be presented by APCs and thereby elicit a robust immune response (**Supplementary Tables 1, 2**). A selection of 35 peptides was identified and synthesized by an external vendor for this purpose (Sino Biological). The peptide fragments selected were derived from each of the three mutated APC regions (HS1, HS2, and HS3 regions) as well as a selection of peptides from each region that were identified via bioinformatics assessment to have strong binding potential (SB peptides) (**Figures 2A, B**). Upon receipt of the peptides, human peripheral blood mononuclear cells (PBMCs) from multiple donors were cultured, stimulated, activated, and re-activated *in vitro* through the addition and subsequent re-addition of the peptides. The cell supernatants from the PBMCs for each treated condition were harvested and were subsequently assayed for human interferon-gamma (IFN-γ) expression—a type II interferon that is secreted from lymphocytes in response to restimulation by a recognized antigen.

Of the 35 peptide fragments tested, 29 exhibited IFN-γ concentrations greater than two-fold higher than unstimulated control cells for one or more PBMC donors, demonstrating robust T cell mediated responses against the mutant APC regions of the neoantigen. The recognized peptides were derived from all three of the mutated regions in the mutant APC protein expressed in the APC mutant replicon, indicating stimulation of T-cell mediated immunogenicity from each of the mutant epitopes in the APCmut sequence (**Figure 2C**). To confirm that the IFN-γ secretion measured following restimulation of PBMCs was released from T cells specifically, a set of PBMCs were treated with monensin to block cytokine secretion and subsequently labeled with anti-CD3 surface markers to identify the T cell populations. Cells were then fixed, permeabilized, and stained for intracellular IFN-γ and compared against unstimulated control, as well as CEF peptide pool and PMA/ionomycin positive controls. Significantly higher intracellular IFN-γ concentrations were observed in restimulated T cells as compared against unstimulated controls, further demonstrating T cell mediated activity against the APC mutant sequences in the neoantigen (**Figure 2D**; **Supplementary Table 4**).

### 3.3 *In vivo* expression and immune stimulation study

**Figure 3:**
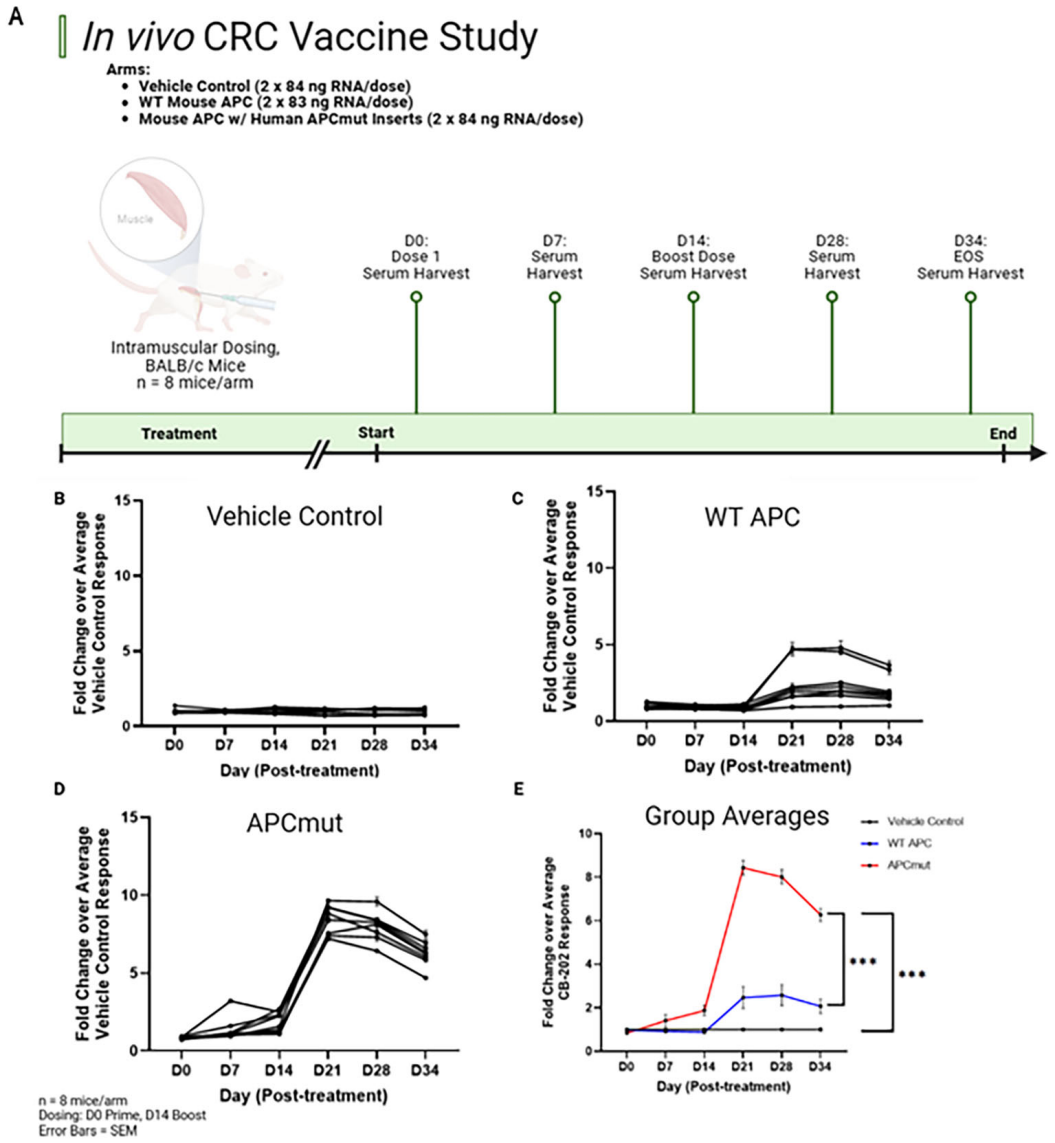
The nanoparticle APC vaccine dosing study confirmed successful transduction and neoantigen expression *in vivo*, while also providing evidence neoantigen-specific immune activation in BALB/c mice. **(A)** The *in vivo* study design featured a two-dose regimen in BALB/c mice, including a dose on D0 and boost on D14. Doses were delivered intramuscularly with 50 uL of nanoparticle suspension injected into each hindlimb. Weekly serum sampling was performed for use in antigen-specific IgG titer assessment. **(B–E)** Significant APC neoantigen-specific IgG concentrations were detected in serum samples drawn from mice at specified intervals following intramuscular dosing. Boost dosing at D14 yielded a significant increase in antigen-specific IgG titer. Titers were measured via ELISA with all serum samples diluted 1:200 to reduce nonspecific binding. Results are reported as the fold-change increase in antigen-specific IgG titer over serum from mice dosed with vehicle control nanoparticle. ***p < 0.001.

After confirming T-cell mediated immunogenicity against the mutant regions of the APCmut protein encoded in the nanoparticle replicon, it was necessary to confirm the nanoparticle platform’s capacity to express the neoantigen and elicit an immune response *in vivo*. The study design for *in vivo* dosing of BALB/c mice with nanoparticle constructs featured three study arms, each including 8 mice per arm. The arms included a vehicle control particle containing no gene-of-interest (CB-202), a WT APC control arm (pCBIO-1289), and the APC mutant arm (pCBIO-1264) (**Figure 3A**). Mice were dosed intramuscularly with 50 uL of nanoparticle suspension into each hindlimb for a total dose volume of 100 uL. Dosing concentrations noted in **Figure 3** specify the nanoparticle RNA concentration in each of the two 50 uL injections delivered on D0 and D14. Blood sampling was performed and processed to serum at weekly intervals and end-of-study. Due to the IM route of administration, POI concentrations in mouse serum were undetectable, and so serum samples were then analyzed via ELISA for APCmut neoantigen-specific IgG titer to confirm POI expression from nanoparticle dosing and immune stimulation compared against controls of empty nanoparticle vehicle and nanoparticles containing saRNA encoding WT mouse APC protein.

Significant APCmut neoantigen-specific IgG concentration was observed at end-of-study in treated mice compared against both vehicle control treated mice [t(14) = 17.54, p<0.001] and mice treated with nanoparticles encoding WT mouse APC (t(14) = 9.57, p<0.001) in comparison of fold change increase over vehicle control. Peak significance in response was observed at one week post boost [t(14) = 21.96, p<0.001 against vehicle control and t(14) = 9.90, p<0.001 against WT mouse APC control] (**Figure 3B**). Additionally, a significant increase in antigen-specific IgG titer was observed following boost dose at D21, suggesting high repeat-dose tolerance in treated mice and low nanoparticle-directed immunogenicity following initial dosing. As is typically observed during *in vivo* IgG quantitation for immune stimulation, IgG titers begin to diminish following completion of the dosing regimen. However, the prolonged expression of APCmut neoantigen resulting from saRNA-based transduction may facilitate a more gradual decrease in IgG titers compared to traditional protein subunit or mRNA-based vaccine approaches.

Mouse weights were tracked throughout performance of the study to monitor any potential toxicity resulting from nanoparticle dosing. Mice from each arm exhibited healthy weight progression with no observed outliers and no indication of toxicity or other adverse effects (**Supplementary Figure 2A**). Histopathological assessment via H&E staining of harvested mouse liver and kidney tissues has previously been performed to further assess toxicity at end of study following IM dosing of the chimeric nanoparticle. No signs indicating tissue toxicity have been observed, which further indicates well-tolerated IM dosing of the nanoparticle in mice (**Supplementary Figure 2B**).

## 4 Discussion

In this study, we utilized bioinformatics to process CRC patient databases and identify conserved frameshift mutations in the MCR of the APC gene, where FAP-associated mutation is highly observed. Identification of conserved frameshift mutations across multiple CRC patients and their inclusion within a single neoantigen is essential for an efficacious and broadly applicable vaccine. Our bioinformatics querying of patient databases identified three regions within the MCR that result in frameshift mutations in APC. Notably, the mutant APC protein resulting from these frameshift mutations featured conserved mutant sequences between multiple patients in the database. As a result, these mutations represent a high-value target for broad vaccine efficacy across a multitude of patients.

The identified conserved regions were used to design a novel APC mutant neoantigen, and this neoantigen was then encoded in a saRNA replicon and delivered via VLP to host cells. These nanoparticles were confirmed to transduce cells *in vitro*, which subsequently secreted APCmut neoantigen into the extracellular space. An *in vivo* dose/boost study was performed in BALB/c mice, which were dosed IM with nanoparticle expressing APCmut neoantigen, vehicle control nanoparticles, or control particles expression WT APC. Compared against control arms, APCmut treated mice exhibited significant increases in neoantigen-specific serum IgG following dose and boost treatments. It has been previously shown that neoantigen-specific serum IgG titers are indicative of a comparable T-cell mediated response *in vivo*, suggesting a high likelihood of such a response resulting from this vaccine approach (24).

A notably valuable finding in this study is in regard to the specificity of the observed immune response to the mutant inserts within APC. The significantly higher antigen-specific IgG titers observed in APCmut mice versus WT APC mice indicates that it is the specific mutant sequences within the APCmut construct that stimulate the most robust immune activation. The ability to stimulate a targeted immune response against the FAP-associated APC mutations within the endogenously expressed APC protein suggests noteworthy potential as a vaccine against FAP and CRC in general.

Joining this pivotal finding, the *in vitro* T cell stimulation assay performed using peptide fragments from within the mutant APC inserts revealed a measurable T cell mediated response against the neoantigen, detected via IFN-γ increases in PBMC supernatants following restimulation with APCmut peptide fragments. This suggests that the frameshift mutations encoded within the APCmut neoantigen may be recognized by T cells that have been previously stimulated. Given the intracellular expression of the APC protein and the requirement for MHC-based presentation of mutant APC for activity against CRC, this finding represents an essential immune stimulation feature for a successful response in the context of FAP-associated CRC.

## Data Availability

The original contributions presented in the study are included in the article/**Supplementary Material**. Further inquiries can be directed to the corresponding authors.
