# Supplementary material for: Identification, design, and in vivo proof of concept of a shared APC neoantigen delivered via a self-amplifying RNA containing virus-like nanoparticle for cancer vaccination
Source: Front Immunol. 2026 Jun 4;17:1810178. doi: 10.3389/fimmu.2026.1810178 (PMC13275407; doi:10.3389/fimmu.2026.1810178)
Supplement: Supplementary file 1 [file DataSheet1.pdf]

jobid=6679A091001D25E25ABB7415&wait=20 Server Output - DTU Health Tech

```
# NetMHC version 4.0

# Input is in FSA format

# Peptide length 8,9,10
# Rank Threshold for Strong binding peptides   0.500
# Rank Threshold for Weak binding peptides   2.000
-----------------------------------------------------------------------------------
```

pos	HLA	peptide	Core	Offset	I_pos	I_len	D_pos	D_len	iCore	Identity	1-log50k(aff)	Affinity(nM)	%Rank	BindLeve
51	HLA-A0101	RVDLSKLQA	RVDLSKLQA	0	0	0	0	0	RVDLSKLQA	Sequence	0.120	13701.00	4.50	
39	HLA-A0101	YLKIKHLLL	YLKIKHLLL	0	0	0	0	0	YLKIKHLLL	Sequence	0.113	14661.94	5.00	
0	HLA-A0101	MVSGIISPV	MVSGIISPV	0	0	0	0	0	MVSGIISPV	Sequence	0.111	15041.15	5.50	
5	HLA-A0101	ISPVIFQIA	ISPVIFQIA	0	0	0	0	0	ISPVIFQIA	Sequence	0.101	16785.82	6.50	
57	HLA-A0101	LQAAVNAAV	LQAAVNAAV	0	0	0	0	0	LQAAVNAAV	Sequence	0.096	17628.10	7.00	
53	HLA-A0101	DLSKLQAAV	DLSKLQAAV	0	0	0	0	0	DLSKLQAAV	Sequence	0.096	17698.23	7.00	
22	HLA-A0101	EVKHLHHLL	EVKHLHHLL	0	0	0	0	0	EVKHLHHLL	Sequence	0.084	20131.05	9.50	
4	HLA-A0101	IISPVIFQI	IISPVIFQI	0	0	0	0	0	IISPVIFQI	Sequence	0.084	20156.99	9.50	
56	HLA-A0101	KLQAAVNAA	KLQAAVNAA	0	0	0	0	0	KLQAAVNAA	Sequence	0.081	20855.37	11.00	
8	HLA-A0101	VIFQIALDK	VIFQIALDK	0	0	0	0	0	VIFQIALDK	Sequence	0.080	20975.31	11.00	
1	HLA-A0101	VSGIISPVI	VSGIISPVI	0	0	0	0	0	VSGIISPVI	Sequence	0.080	21139.82	11.00	
38	HLA-A0101	KYLKIKHLLL	YLKIKHLLL	1	0	0	0	0	YLKIKHLLL	Sequence	0.075	22225.31	13.00	
51	HLA-A0101	RVDLSKLQAA	RVDLSKLAA	0	0	0	7	1	RVDLSKLQAA	Sequence	0.072	22922.59	14.00	
1	HLA-A0101	VSGIISPVIF	VSGIISVIF	0	0	0	6	1	VSGIISPVIF	Sequence	0.070	23410.60	15.00	
44	HLA-A0101	HLLLKRERV	HLLLKRERV	0	0	0	0	0	HLLLKRERV	Sequence	0.070	23432.39	15.00	
2	HLA-A0101	SGIISPVIF	SGIISPVIF	0	0	0	0	0	SGIISPVIF	Sequence	0.070	23528.17	16.00	
50	HLA-A0101	ERVDLSKLQA	EVDLSKLQA	0	0	0	1	1	ERVDLSKLQA	Sequence	0.069	23695.75	16.00	
20	HLA-A0101	QAEVKHLHH	QAEVKHLHH	0	0	0	0	0	QAEVKHLHH	Sequence	0.068	24013.47	17.00	
38	HLA-A0101	KYLKIKHLL	KYLKIKHLL	0	0	0	0	0	KYLKIKHLL	Sequence	0.067	24328.87	17.00	
13	HLA-A0101	ALDKPCHQA	ALDKPCHQA	0	0	0	0	0	ALDKPCHQA	Sequence	0.066	24573.32	18.00	
39	HLA-A0101	YLKIKHLLLK	YLIKHLLLK	0	0	0	2	1	YLKIKHLLLK	Sequence	0.065	24862.15	19.00	
6	HLA-A0101	SPVIFQIAL	SPVIFQIAL	0	0	0	0	0	SPVIFQIAL	Sequence	0.064	24904.97	19.00	
25	HLA-A0101	HLHHLLKQL	HLHHLLKQL	0	0	0	0	0	HLHHLLKQL	Sequence	0.063	25267.30	20.00	
56	HLA-A0101	KLQAAVNAAV	KLAAVNAAV	0	0	0	2	1	KLQAAVNAAV	Sequence	0.062	25650.20	21.00	
40	HLA-A0101	LKIKHLLLK	LKIKHLLLK	0	0	0	0	0	LKIKHLLLK	Sequence	0.060	26112.80	22.00	
46	HLA-A0101	LLKRERVDL	LLKRERVDL	0	0	0	0	0	LLKRERVDL	Sequence	0.059	26300.79	23.00	
1	HLA-A0101	VSGIISPV	VSG-IISPV	0	3	1	0	0	VSGIISPV	Sequence	0.058	26798.30	24.00	
52	HLA-A0101	VDLSKLQAAV	VLSKLQAAV	0	0	0	1	1	VDLSKLQAAV	Sequence	0.057	26933.19	25.00	
5	HLA-A0101	ISPVIFQIAL	ISPVFQIAL	0	0	0	4	1	ISPVIFQIAL	Sequence	0.057	26988.30	25.00	
39	HLA-A0101	YLKIKHLL	YLKIK-HLL	0	5	1	0	0	YLKIKHLL	Sequence	0.057	27023.38	25.00	
32	HLA-A0101	QLKPSEKYL	QLKPSEKYL	0	0	0	0	0	QLKPSEKYL	Sequence	0.057	27034.78	25.00	
10	HLA-A0101	FQIALDKPC	FQIALDKPC	0	0	0	0	0	FQIALDKPC	Sequence	0.057	27037.12	25.00	
37	HLA-A0101	EKYLKIKHL	EKYLKIKHL	0	0	0	0	0	EKYLKIKHL	Sequence	0.057	27089.53	25.00	
26	HLA-A0101	LHHLLKQLK	LHHLLKQLK	0	0	0	0	0	LHHLLKQLK	Sequence	0.056	27166.44	25.00	
19	HLA-A0101	HQAEVKHLH	HQAEVKHLH	0	0	0	0	0	HQAEVKHLH	Sequence	0.056	27227.05	26.00	
4	HLA-A0101	IISPVIFQIA	IISVIFQIA	0	0	0	3	1	IISPVIFQIA	Sequence	0.056	27339.82	26.00	
49	HLA-A0101	RERVDLSKL	RERVDLSKL	0	0	0	0	0	RERVDLSKL	Sequence	0.056	27390.75	26.00	
52	HLA-A0101	VDLSKLQAA	VDLSKLQAA	0	0	0	0	0	VDLSKLQAA	Sequence	0.056	27424.27	26.00	
54	HLA-A0101	LSKLQAAV	LS-KLQAAV	0	2	1	0	0	LSKLQAAV	Sequence	0.055	27508.37	26.00	
3	HLA-A0101	GIISPVIFQ	GIISPVIFQ	0	0	0	0	0	GIISPVIFQ	Sequence	0.055	27648.00	27.00	
35	HLA-A0101	PSEKYLKIK	PSEKYLKIK	0	0	0	0	0	PSEKYLKIK	Sequence	0.055	27657.58	27.00	
34	HLA-A0101	KPSEKYLKI	KPSEKYLKI	0	0	0	0	0	KPSEKYLKI	Sequence	0.054	27888.36	28.00	
48	HLA-A0101	KRERVDLSK	KRERVDLSK	0	0	0	0	0	KRERVDLSK	Sequence	0.053	28272.09	29.00	
7	HLA-A0101	PVIFQIALD	PVIFQIALD	0	0	0	0	0	PVIFQIALD	Sequence	0.053	28325.96	29.00	
31	HLA-A0101	KQLKPSEKY	KQLKPSEKY	0	0	0	0	0	KQLKPSEKY	Sequence	0.052	28427.29	30.00	
58	HLA-A0101	QAAVNAAVQ	QAAVNAAVQ	0	0	0	0	0	QAAVNAAVQ	Sequence	0.052	28553.06	30.00	
18	HLA-A0101	CHQAEVKHL	CHQAEVKHL	0	0	0	0	0	CHQAEVKHL	Sequence	0.051	28716.64	31.00	
0	HLA-A0101	MVSGIISPVI	MSGIISPVI	0	0	0	1	1	MVSGIISPVI	Sequence	0.051	28751.46	31.00	
21	HLA-A0101	AEVKHLHHL	AEVKHLHHL	0	0	0	0	0	AEVKHLHHL	Sequence	0.051	28846.20	31.00	
37	HLA-A0101	EKYLKIKHLL	EKYLKIHLL	0	0	0	6	1	EKYLKIKHLL	Sequence	0.049	29389.32	34.00	
15	HLA-A0101	DKPCHQAEV	DKPCHQAEV	0	0	0	0	0	DKPCHQAEV	Sequence	0.049	29402.35	34.00	
23	HLA-A0101	VKHLHHLLK	VKHLHHLLK	0	0	0	0	0	VKHLHHLLK	Sequence	0.049	29408.40	34.00	
35	HLA-A0101	PSEKYLKI	PSE-KYLKI	0	3	1	0	0	PSEKYLKI	Sequence	0.049	29495.39	34.00	
20	HLA-A0101	QAEVKHLHHL	QAEVKHLHL	0	0	0	7	1	QAEVKHLHHL	Sequence	0.048	29752.46	35.00	
3	HLA-A0101	GIISPVIFQI	IISPVIFQI	1	0	0	0	0	IISPVIFQI	Sequence	0.048	29834.33	36.00	
7	HLA-A0101	PVIFQIALDK	VIFQIALDK	1	0	0	0	0	VIFQIALDK	Sequence	0.046	30490.26	38.00	
47	HLA-A0101	LKRERVDLS	LKRERVDLS	0	0	0	0	0	LKRERVDLS	Sequence	0.044	30907.78	40.00	
41	HLA-A0101	KIKHLLLKR	KIKHLLLKR	0	0	0	0	0	KIKHLLLKR	Sequence	0.044	30930.19	40.00	
5	HLA-A0101	ISPVIFQI	IS-PVIFQI	0	2	1	0	0	ISPVIFQI	Sequence	0.044	31038.15	41.00	
21	HLA-A0101	AEVKHLHHLL	EVKHLHHLL	1	0	0	0	0	EVKHLHHLL	Sequence	0.044	31088.90	41.00	
50	HLA-A0101	ERVDLSKLQ	ERVDLSKLQ	0	0	0	0	0	ERVDLSKLQ	Sequence	0.044	31204.82	42.00	
55	HLA-A0101	SKLQAAVNAA	KLQAAVNAA	1	0	0	0	0	KLQAAVNAA	Sequence	0.043	31346.62	43.00	
57	HLA-A0101	LQAAVNAAVQ	LQAAVNAAV	0	0	0	0	0	LQAAVNAAV	Sequence	0.043	31351.71	43.00	
9	HLA-A0101	IFQIALDKP	IFQIALDKP	0	0	0	0	0	IFQIALDKP	Sequence	0.042	31599.96	44.00	
40	HLA-A0101	LKIKHLLL	-LKIKHLLL	0	0	1	0	0	LKIKHLLL	Sequence	0.042	31648.22	44.00	
7	HLA-A0101	PVIFQIAL	PVIF-QIAL	0	4	1	0	0	PVIFQIAL	Sequence	0.042	31679.40	44.00	
3	HLA-A0101	GIISPVIF	GIISPV-IF	0	6	1	0	0	GIISPVIF	Sequence	0.042	31775.86	45.00	
24	HLA-A0101	KHLHHLLKQ	KHLHHLLKQ	0	0	0	0	0	KHLHHLLKQ	Sequence	0.042	31821.61	45.00	
28	HLA-A0101	HLLKQLKPS	HLLKQLKPS	0	0	0	0	0	HLLKQLKPS	Sequence	0.041	32114.59	46.00	
54	HLA-A0101	LSKLQAAVN	LSKLQAAVN	0	0	0	0	0	LSKLQAAVN	Sequence	0.041	32139.95	46.00	
27	HLA-A0101	HHLLKQLKP	HHLLKQLKP	0	0	0	0	0	HHLLKQLKP	Sequence	0.041	32152.14	47.00	
16	HLA-A0101	KPCHQAEVK	KPCHQAEVK	0	0	0	0	0	KPCHQAEVK	Sequence	0.041	32156.66	47.00	
32	HLA-A0101	QLKPSEKY	QLKPS-EKY	0	5	1	0	0	QLKPSEKY	Sequence	0.040	32273.77	47.00	
45	HLA-A0101	LLLKRERVDL	LLKRERVDL	1	0	0	0	0	LLKRERVDL	Sequence	0.040	32401.49	48.00	
55	HLA-A0101	SKLQAAVNA	SKLQAAVNA	0	0	0	0	0	SKLQAAVNA	Sequence	0.040	32579.36	49.00	

43	HLA-A0101	KHLLLKRERV	HLLLKRERV	1	0	0	0	0	HLLLKRERV	Sequence	0.040	32599.45	49.00
58	HLA-A0101	QAAVNAAV	-QAAVNAAV	0	0	1	0	0	QAAVNAAV	Sequence	0.039	32763.87	50.00
6	HLA-A0101	SPVIFQIA	-SPVIFQIA	0	0	1	0	0	SPVIFQIA	Sequence	0.039	32773.11	50.00
11	HLA-A0101	QIALDKPCH	QIALDKPCH	0	0	0	0	0	QIALDKPCH	Sequence	0.039	32802.18	50.00
57	HLA-A0101	LQAAVNAA	-LQAAVNAA	0	0	1	0	0	LQAAVNAA	Sequence	0.038	33000.12	55.00
24	HLA-A0101	KHLHHLLKQL	HLHHLLKQL	1	0	0	0	0	HLHHLLKQL	Sequence	0.038	33128.55	55.00
53	HLA-A0101	DLSKLQAAVN	DLSKLQAAV	0	0	0	0	0	DLSKLQAAV	Sequence	0.038	33229.06	55.00
33	HLA-A0101	LKPSEKYLK	LKPSEKYLK	0	0	0	0	0	LKPSEKYLK	Sequence	0.038	33229.79	55.00
43	HLA-A0101	KHLLLKRER	KHLLLKRER	0	0	0	0	0	KHLLLKRER	Sequence	0.038	33262.87	55.00
8	HLA-A0101	VIFQIALD	VIFQIALD-	0	8	1	0	0	VIFQIALD	Sequence	0.037	33488.21	55.00
22	HLA-A0101	EVKHLHHLLK	EVKHLHHLL	0	0	0	0	0	EVKHLHHLL	Sequence	0.037	33535.71	55.00
31	HLA-A0101	KQLKPSEKYL	QLKPSEKYL	1	0	0	0	0	QLKPSEKYL	Sequence	0.037	33569.12	55.00
30	HLA-A0101	LKQLKPSEK	LKQLKPSEK	0	0	0	0	0	LKQLKPSEK	Sequence	0.037	33670.24	55.00
52	HLA-A0101	VDLSKLQA	-VDLSKLQA	0	0	1	0	0	VDLSKLQA	Sequence	0.036	33804.57	55.00
25	HLA-A0101	HLHHLLKQLK	HLHHLLQLK	0	0	0	6	1	HLHHLLKQLK	Sequence	0.036	33878.89	60.00
48	HLA-A0101	KRERVDLSKL	KRERVDLSL	0	0	0	8	1	KRERVDLSKL	Sequence	0.036	33930.62	60.00
54	HLA-A0101	LSKLQAAVNA	LSKLQAAVA	0	0	0	8	1	LSKLQAAVNA	Sequence	0.036	33930.99	60.00
14	HLA-A0101	LDKPCHQAEV	LDPCHQAEV	0	0	0	2	1	LDKPCHQAEV	Sequence	0.036	33976.17	60.00
19	HLA-A0101	HQAEVKHLHH	HAEVKHLHH	0	0	0	1	1	HQAEVKHLHH	Sequence	0.036	34034.30	60.00
45	HLA-A0101	LLLKRERV	LL-LKRERV	0	2	1	0	0	LLLKRERV	Sequence	0.035	34054.94	60.00
12	HLA-A0101	IALDKPCHQ	IALDKPCHQ	0	0	0	0	0	IALDKPCHQ	Sequence	0.035	34079.26	60.00
9	HLA-A0101	IFQIALDK	-IFQIALDK	0	0	1	0	0	IFQIALDK	Sequence	0.035	34082.58	60.00
30	HLA-A0101	LKQLKPSEKY	LKQLKPSEY	0	0	0	8	1	LKQLKPSEKY	Sequence	0.035	34108.39	60.00
16	HLA-A0101	KPCHQAEV	KP-CHQAEV	0	2	1	0	0	KPCHQAEV	Sequence	0.035	34160.11	60.00
6	HLA-A0101	SPVIFQIALD	SPVIFQIAL	0	0	0	0	0	SPVIFQIAL	Sequence	0.035	34167.13	60.00
53	HLA-A0101	DLSKLQAA	DL-SKLQAA	0	2	1	0	0	DLSKLQAA	Sequence	0.035	34178.97	60.00
45	HLA-A0101	LLLKRERVD	LLLKRERVD	0	0	0	0	0	LLLKRERVD	Sequence	0.035	34247.81	60.00
8	HLA-A0101	VIFQIALDKP	VIFQIALDK	0	0	0	0	0	VIFQIALDK	Sequence	0.035	34265.97	60.00
36	HLA-A0101	SEKYLKIKHL	EKYLKIKHL	1	0	0	0	0	EKYLKIKHL	Sequence	0.035	34382.23	60.00
50	HLA-A0101	ERVDLSKL	ERVDL-SKL	0	5	1	0	0	ERVDLSKL	Sequence	0.035	34408.27	60.00
2	HLA-A0101	SGIISPVIFQ	SGIISPVIF	0	0	0	0	0	SGIISPVIF	Sequence	0.034	34450.75	60.00
14	HLA-A0101	LDKPCHQAE	LDKPCHQAE	0	0	0	0	0	LDKPCHQAE	Sequence	0.034	34501.84	60.00
2	HLA-A0101	SGIISPVI	-SGIISPVI	0	0	1	0	0	SGIISPVI	Sequence	0.034	34657.12	60.00
17	HLA-A0101	PCHQAEVKH	PCHQAEVKH	0	0	0	0	0	PCHQAEVKH	Sequence	0.034	34713.78	65.00
44	HLA-A0101	HLLLKRERVD	HLLLKRERV	0	0	0	0	0	HLLLKRERV	Sequence	0.034	34770.17	65.00
29	HLA-A0101	LLKQLKPSE	LLKQLKPSE	0	0	0	0	0	LLKQLKPSE	Sequence	0.033	34899.44	65.00
56	HLA-A0101	KLQAAVNA	KLQAAVN-A	0	7	1	0	0	KLQAAVNA	Sequence	0.033	35033.75	65.00
23	HLA-A0101	VKHLHHLL	-VKHLHHLL	0	0	1	0	0	VKHLHHLL	Sequence	0.033	35110.02	65.00
22	HLA-A0101	EVKHLHHL	EV-KHLHHL	0	2	1	0	0	EVKHLHHL	Sequence	0.032	35278.34	65.00
9	HLA-A0101	IFQIALDKPC	FQIALDKPC	1	0	0	0	0	FQIALDKPC	Sequence	0.032	35528.85	65.00
32	HLA-A0101	QLKPSEKYLK	QLKPSEYLK	0	0	0	6	1	QLKPSEKYLK	Sequence	0.032	35543.47	65.00
35	HLA-A0101	PSEKYLKIKH	PSEKYLIKH	0	0	0	6	1	PSEKYLKIKH	Sequence	0.031	35594.26	70.00
38	HLA-A0101	KYLKIKHL	K-YLKIKHL	0	1	1	0	0	KYLKIKHL	Sequence	0.031	35606.59	70.00
46	HLA-A0101	LLKRERVDLS	LLRERVDLS	0	0	0	2	1	LLKRERVDLS	Sequence	0.031	35780.77	70.00
0	HLA-A0101	MVSGIISP	MVSGIISP-	0	8	1	0	0	MVSGIISP	Sequence	0.030	36198.59	70.00
29	HLA-A0101	LLKQLKPSEK	LLQLKPSEK	0	0	0	2	1	LLKQLKPSEK	Sequence	0.030	36278.20	70.00
33	HLA-A0101	LKPSEKYLKI	KPSEKYLKI	1	0	0	0	0	KPSEKYLKI	Sequence	0.030	36335.94	70.00
26	HLA-A0101	LHHLLKQL	-LHHLLKQL	0	0	1	0	0	LHHLLKQL	Sequence	0.029	36386.68	70.00
34	HLA-A0101	KPSEKYLKIK	PSEKYLKIK	1	0	0	0	0	PSEKYLKIK	Sequence	0.029	36660.93	75.00
4	HLA-A0101	IISPVIFQ	IISPVIFQ-	0	8	1	0	0	IISPVIFQ	Sequence	0.028	36737.56	75.00
47	HLA-A0101	LKRERVDLSK	LRERVDLSK	0	0	0	1	1	LKRERVDLSK	Sequence	0.028	36770.98	75.00
12	HLA-A0101	IALDKPCHQA	ALDKPCHQA	1	0	0	0	0	ALDKPCHQA	Sequence	0.028	36827.11	75.00
20	HLA-A0101	QAEVKHLH	QAEVK-HLH	0	5	1	0	0	QAEVKHLH	Sequence	0.028	36887.72	75.00
19	HLA-A0101	HQAEVKHL	HQAEVKH-L	0	7	1	0	0	HQAEVKHL	Sequence	0.028	36942.45	75.00
49	HLA-A0101	RERVDLSKLQ	RERVDLSKL	0	0	0	0	0	RERVDLSKL	Sequence	0.027	37142.04	75.00
24	HLA-A0101	KHLHHLLK	KHL-HHLLK	0	3	1	0	0	KHLHHLLK	Sequence	0.027	37313.63	80.00
36	HLA-A0101	SEKYLKIKH	SEKYLKIKH	0	0	0	0	0	SEKYLKIKH	Sequence	0.027	37476.68	80.00
11	HLA-A0101	QIALDKPCHQ	QIADKPCHQ	0	0	0	3	1	QIALDKPCHQ	Sequence	0.027	37517.67	80.00
33	HLA-A0101	LKPSEKYL	-LKPSEKYL	0	0	1	0	0	LKPSEKYL	Sequence	0.026	37581.46	80.00
41	HLA-A0101	KIKHLLLK	KI-KHLLLK	0	2	1	0	0	KIKHLLLK	Sequence	0.026	37640.47	80.00
17	HLA-A0101	PCHQAEVKHL	CHQAEVKHL	1	0	0	0	0	CHQAEVKHL	Sequence	0.026	37722.00	80.00
13	HLA-A0101	ALDKPCHQ	ALD-KPCHQ	0	3	1	0	0	ALDKPCHQ	Sequence	0.026	37758.34	80.00
18	HLA-A0101	CHQAEVKHLH	HQAEVKHLH	1	0	0	0	0	HQAEVKHLH	Sequence	0.026	37775.93	80.00
10	HLA-A0101	FQIALDKPCH	FQIALDPCH	0	0	0	6	1	FQIALDKPCH	Sequence	0.026	37790.64	80.00
47	HLA-A0101	LKRERVDL	LKRERVD-L	0	7	1	0	0	LKRERVDL	Sequence	0.026	37812.73	80.00
40	HLA-A0101	LKIKHLLLKR	LKIKHLLLK	0	0	0	0	0	LKIKHLLLK	Sequence	0.026	37883.15	80.00
27	HLA-A0101	HHLLKQLK	HHLLKQ-LK	0	6	1	0	0	HHLLKQLK	Sequence	0.026	37901.60	80.00
15	HLA-A0101	DKPCHQAEVK	DKPCHQAEV	0	0	0	0	0	DKPCHQAEV	Sequence	0.025	38157.94	80.00
44	HLA-A0101	HLLLKRER	HLLLKRER-	0	8	1	0	0	HLLLKRER	Sequence	0.024	38514.67	85.00
28	HLA-A0101	HLLKQLKP	HLL-KQLKP	0	3	1	0	0	HLLKQLKP	Sequence	0.024	38724.86	85.00
10	HLA-A0101	FQIALDKP	FQIALDKP-	0	8	1	0	0	FQIALDKP	Sequence	0.023	38852.01	85.00
37	HLA-A0101	EKYLKIKH	EKYLKI-KH	0	6	1	0	0	EKYLKIKH	Sequence	0.023	38865.46	85.00
51	HLA-A0101	RVDLSKLQ	RVDLSKLQ-	0	8	1	0	0	RVDLSKLQ	Sequence	0.023	38882.29	85.00
49	HLA-A0101	RERVDLSK	-RERVDLSK	0	0	1	0	0	RERVDLSK	Sequence	0.023	39080.11	85.00
11	HLA-A0101	QIALDKPC	-QIALDKPC	0	0	1	0	0	QIALDKPC	Sequence	0.023	39133.42	85.00
25	HLA-A0101	HLHHLLKQ	HL-HHLLKQ	0	2	1	0	0	HLHHLLKQ	Sequence	0.022	39205.90	90.00
13	HLA-A0101	ALDKPCHQAE	ALDPCHQAE	0	0	0	3	1	ALDKPCHQAE	Sequence	0.022	39247.05	90.00
27	HLA-A0101	HHLLKQLKPS	HHLLKQLPS	0	0	0	7	1	HHLLKQLKPS	Sequence	0.022	39286.99	90.00
14	HLA-A0101	LDKPCHQA	-LDKPCHQA	0	0	1	0	0	LDKPCHQA	Sequence	0.022	39527.05	90.00
21	HLA-A0101	AEVKHLHH	-AEVKHLHH	0	0	1	0	0	AEVKHLHH	Sequence	0.022	39579.24	90.00
42	HLA-A0101	IKHLLLKRE	IKHLLLKRE	0	0	0	0	0	IKHLLLKRE	Sequence	0.021	39696.34	90.00
26	HLA-A0101	LHHLLKQLKP	LHHLLKQLK	0	0	0	0	0	LHHLLKQLK	Sequence	0.021	39735.02	90.00
31	HLA-A0101	KQLKPSEK	KQL-KPSEK	0	3	1	0	0	KQLKPSEK	Sequence	0.021	39880.58	90.00
48	HLA-A0101	KRERVDLS	KRE-RVDLS	0	3	1	0	0	KRERVDLS	Sequence	0.021	39912.10	90.00
34	HLA-A0101	KPSEKYLK	KP-SEKYLK	0	2	1	0	0	KPSEKYLK	Sequence	0.020	40186.84	90.00
23	HLA-A0101	VKHLHHLLKQ	KHLHHLLKQ	1	0	0	0	0	KHLHHLLKQ	Sequence	0.020	40260.82	95.00
28	HLA-A0101	HLLKQLKPSE	LLKQLKPSE	1	0	0	0	0	LLKQLKPSE	Sequence	0.020	40438.49	95.00
59	HLA-A0101	AAVNAAVQ	-AAVNAAVQ	0	0	1	0	0	AAVNAAVQ	Sequence	0.019	40572.16	95.00
42	HLA-A0101	IKHLLLKRER	KHLLLKRER	1	0	0	0	0	KHLLLKRER	Sequence	0.019	40909.84	95.00
36	HLA-A0101	SEKYLKIK	-SEKYLKIK	0	0	1	0	0	SEKYLKIK	Sequence	0.018	40951.02	95.00

29	HLA-A0101	LLKQLKPS	LLKQLK-PS	0	6	1	0	0	LLKQLKPS	Sequence	0.018	40958.09	95.00
17	HLA-A0101	PCHQAEVK	PCHQA-EVK	0	5	1	0	0	PCHQAEVK	Sequence	0.018	41108.16	95.00
16	HLA-A0101	KPCHQAEVKH	KPCHQAEVK	0	0	0	0	0	KPCHQAEVK	Sequence	0.018	41257.43	95.00
15	HLA-A0101	DKPCHQAE	DKPCHQAE-	0	8	1	0	0	DKPCHQAE	Sequence	0.017	41395.59	95.00
43	HLA-A0101	KHLLLKRE	KHLLLKRE-	0	8	1	0	0	KHLLLKRE	Sequence	0.017	41563.90	99.00
12	HLA-A0101	IALDKPCH	IALDKPCH-	0	8	1	0	0	IALDKPCH	Sequence	0.017	41669.73	99.00
41	HLA-A0101	KIKHLLLKRE	KIKHLLLKR	0	0	0	0	0	KIKHLLLKR	Sequence	0.017	41760.45	99.00
46	HLA-A0101	LLKRERVD	LLKRERVD-	0	8	1	0	0	LLKRERVD	Sequence	0.016	41878.09	99.00
42	HLA-A0101	IKHLLLKR	-IKHLLLKR	0	0	1	0	0	IKHLLLKR	Sequence	0.016	41948.82	99.00
55	HLA-A0101	SKLQAAVN	SKLQAAVN-	0	8	1	0	0	SKLQAAVN	Sequence	0.016	42249.92	99.00
18	HLA-A0101	CHQAEVKH	CHQAEVKH-	0	8	1	0	0	CHQAEVKH	Sequence	0.015	42361.15	99.00
30	HLA-A0101	LKQLKPSE	LKQLKPSE-	0	8	1	0	0	LKQLKPSE	Sequence	0.013	43274.69	99.00

Protein Sequence. Allele HLA-A0101. Number of high binders 0. Number of weak binders 0. Number of peptides 177

Link to Allele Frequencies in Worldwide Populations HLA-A0101

Rank Threshold for Strong binding peptides 0.500
Rank Threshold for Weak binding peptides 2.000

pos	HLA	peptide	Core	Offset	I_pos	I_len	D_pos	D_len	iCore	Identity	1-log50k(aff)	Affinity(nM)	%Rank	BindLeve
0	HLA-A0201	MVSGIISPV	MVSGIISPV	0	0	0	0	0	MVSGIISPV	Sequence	0.762	13.09	0.17	<= SB
56	HLA-A0201	KLQAAVNAAV	KLAAVNAAV	0	0	0	2	1	KLQAAVNAAV	Sequence	0.651	43.53	0.60	<= WB
4	HLA-A0201	IISPVIFQI	IISPVIFQI	0	0	0	0	0	IISPVIFQI	Sequence	0.640	49.43	0.60	<= WB
57	HLA-A0201	LQAAVNAAV	LQAAVNAAV	0	0	0	0	0	LQAAVNAAV	Sequence	0.606	70.83	0.80	<= WB
56	HLA-A0201	KLQAAVNAA	KLQAAVNAA	0	0	0	0	0	KLQAAVNAA	Sequence	0.508	205.90	1.70	<= WB
3	HLA-A0201	GIISPVIFQI	GIIPVIFQI	0	0	0	3	1	GIISPVIFQI	Sequence	0.468	314.96	2.50	
52	HLA-A0201	VDLSKLQAAV	VLSKLQAAV	0	0	0	1	1	VDLSKLQAAV	Sequence	0.424	509.33	3.00	
0	HLA-A0201	MVSGIISPVI	MVSGIISPV	0	0	0	0	0	MVSGIISPV	Sequence	0.419	535.30	3.00	
4	HLA-A0201	IISPVIFQIA	IISPVIFQA	0	0	0	8	1	IISPVIFQIA	Sequence	0.351	1115.19	4.50	
39	HLA-A0201	YLKIKHLLL	YLKIKHLLL	0	0	0	0	0	YLKIKHLLL	Sequence	0.337	1310.53	5.00	
13	HLA-A0201	ALDKPCHQA	ALDKPCHQA	0	0	0	0	0	ALDKPCHQA	Sequence	0.332	1372.90	5.00	
39	HLA-A0201	YLKIKHLL	YL-KIKHLL	0	2	1	0	0	YLKIKHLL	Sequence	0.281	2382.34	6.50	
44	HLA-A0201	HLLLKRERV	HLLLKRERV	0	0	0	0	0	HLLLKRERV	Sequence	0.281	2384.69	6.50	
55	HLA-A0201	SKLQAAVNAA	KLQAAVNAA	1	0	0	0	0	KLQAAVNAA	Sequence	0.273	2614.12	7.00	
57	HLA-A0201	LQAAVNAAVQ	LQAAVNAAV	0	0	0	0	0	LQAAVNAAV	Sequence	0.258	3071.80	7.50	
25	HLA-A0201	HLHHLLKQL	HLHHLLKQL	0	0	0	0	0	HLHHLLKQL	Sequence	0.256	3141.17	7.50	
53	HLA-A0201	DLSKLQAAV	DLSKLQAAV	0	0	0	0	0	DLSKLQAAV	Sequence	0.250	3344.61	7.50	
43	HLA-A0201	KHLLLKRERV	KLLLKRERV	0	0	0	1	1	KHLLLKRERV	Sequence	0.240	3735.91	8.00	
54	HLA-A0201	LSKLQAAV	-LSKLQAAV	0	0	1	0	0	LSKLQAAV	Sequence	0.235	3942.73	8.50	
38	HLA-A0201	KYLKIKHLLL	YLKIKHLLL	1	0	0	0	0	YLKIKHLLL	Sequence	0.233	4014.54	8.50	
24	HLA-A0201	KHLHHLLKQL	KLHHLLKQL	0	0	0	1	1	KHLHHLLKQL	Sequence	0.226	4351.40	9.00	
45	HLA-A0201	LLLKRERVDL	LLLKREVDL	0	0	0	6	1	LLLKRERVDL	Sequence	0.215	4868.70	9.50	
12	HLA-A0201	IALDKPCHQA	ILDKPCHQA	0	0	0	1	1	IALDKPCHQA	Sequence	0.207	5329.11	10.00	
39	HLA-A0201	YLKIKHLLLK	YLIKHLLLK	0	0	0	2	1	YLKIKHLLLK	Sequence	0.204	5474.81	10.00	
1	HLA-A0201	VSGIISPV	-VSGIISPV	0	0	1	0	0	VSGIISPV	Sequence	0.199	5786.59	11.00	
5	HLA-A0201	ISPVIFQI	-ISPVIFQI	0	0	1	0	0	ISPVIFQI	Sequence	0.195	6051.82	11.00	
5	HLA-A0201	ISPVIFQIAL	ISVIFQIAL	0	0	0	2	1	ISPVIFQIAL	Sequence	0.193	6186.08	11.00	
31	HLA-A0201	KQLKPSEKYL	KQLKPSEYL	0	0	0	7	1	KQLKPSEKYL	Sequence	0.182	6964.85	12.00	
10	HLA-A0201	FQIALDKPC	FQIALDKPC	0	0	0	0	0	FQIALDKPC	Sequence	0.171	7881.12	13.00	
58	HLA-A0201	QAAVNAAV	-QAAVNAAV	0	0	1	0	0	QAAVNAAV	Sequence	0.159	8938.51	14.00	
45	HLA-A0201	LLLKRERV	LLLKR-ERV	0	5	1	0	0	LLLKRERV	Sequence	0.151	9783.13	15.00	
6	HLA-A0201	SPVIFQIAL	SPVIFQIAL	0	0	0	0	0	SPVIFQIAL	Sequence	0.143	10695.18	16.00	
32	HLA-A0201	QLKPSEKYL	QLKPSEKYL	0	0	0	0	0	QLKPSEKYL	Sequence	0.140	11029.52	16.00	
51	HLA-A0201	RVDLSKLQAA	RVLSKLQAA	0	0	0	2	1	RVDLSKLQAA	Sequence	0.136	11538.81	17.00	
56	HLA-A0201	KLQAAVNA	KL-QAAVNA	0	2	1	0	0	KLQAAVNA	Sequence	0.134	11725.70	17.00	
28	HLA-A0201	HLLKQLKPS	HLLKQLKPS	0	0	0	0	0	HLLKQLKPS	Sequence	0.127	12608.53	18.00	
1	HLA-A0201	VSGIISPVI	VSGIISPVI	0	0	0	0	0	VSGIISPVI	Sequence	0.121	13529.67	19.00	
44	HLA-A0201	HLLLKRERVD	HLLLKRERV	0	0	0	0	0	HLLLKRERV	Sequence	0.117	14074.68	20.00	
13	HLA-A0201	ALDKPCHQAE	ALDKPCHQA	0	0	0	0	0	ALDKPCHQA	Sequence	0.117	14082.00	20.00	
5	HLA-A0201	ISPVIFQIA	ISPVIFQIA	0	0	0	0	0	ISPVIFQIA	Sequence	0.116	14258.77	20.00	
3	HLA-A0201	GIISPVIFQ	GIISPVIFQ	0	0	0	0	0	GIISPVIFQ	Sequence	0.113	14780.77	21.00	
57	HLA-A0201	LQAAVNAA	-LQAAVNAA	0	0	1	0	0	LQAAVNAA	Sequence	0.112	14875.58	21.00	
25	HLA-A0201	HLHHLLKQLK	HLHHLLKQL	0	0	0	0	0	HLHHLLKQL	Sequence	0.109	15363.52	21.00	
53	HLA-A0201	DLSKLQAAVN	DLSKLQAAV	0	0	0	0	0	DLSKLQAAV	Sequence	0.108	15559.42	22.00	
8	HLA-A0201	VIFQIALDK	VIFQIALDK	0	0	0	0	0	VIFQIALDK	Sequence	0.105	16138.27	22.00	
51	HLA-A0201	RVDLSKLQA	RVDLSKLQA	0	0	0	0	0	RVDLSKLQA	Sequence	0.099	17163.22	23.00	
9	HLA-A0201	IFQIALDKPC	FQIALDKPC	1	0	0	0	0	FQIALDKPC	Sequence	0.095	17825.85	24.00	
46	HLA-A0201	LLKRERVDL	LLKRERVDL	0	0	0	0	0	LLKRERVDL	Sequence	0.094	18130.26	25.00	
8	HLA-A0201	VIFQIALDKP	VIFQIALDP	0	0	0	8	1	VIFQIALDKP	Sequence	0.093	18326.50	25.00	
26	HLA-A0201	LHHLLKQL	-LHHLLKQL	0	0	1	0	0	LHHLLKQL	Sequence	0.092	18521.25	25.00	
46	HLA-A0201	LLKRERVDLS	LLRERVDLS	0	0	0	2	1	LLKRERVDLS	Sequence	0.088	19286.10	26.00	
2	HLA-A0201	SGIISPVIFQ	SIISPVIFQ	0	0	0	1	1	SGIISPVIFQ	Sequence	0.088	19393.24	27.00	
38	HLA-A0201	KYLKIKHLL	KYLKIKHLL	0	0	0	0	0	KYLKIKHLL	Sequence	0.087	19491.69	27.00	
40	HLA-A0201	LKIKHLLL	-LKIKHLLL	0	0	1	0	0	LKIKHLLL	Sequence	0.086	19782.12	27.00	
2	HLA-A0201	SGIISPVIF	SGIISPVIF	0	0	0	0	0	SGIISPVIF	Sequence	0.084	20212.68	28.00	
6	HLA-A0201	SPVIFQIALD	SPVIFQIAL	0	0	0	0	0	SPVIFQIAL	Sequence	0.084	20257.13	28.00	
14	HLA-A0201	LDKPCHQA	-LDKPCHQA	0	0	1	0	0	LDKPCHQA	Sequence	0.083	20323.21	28.00	
4	HLA-A0201	IISPVIFQ	IISPVIFQ-	0	8	1	0	0	IISPVIFQ	Sequence	0.079	21201.65	29.00	
1	HLA-A0201	VSGIISPVIF	VSIISPVIF	0	0	0	2	1	VSGIISPVIF	Sequence	0.077	21644.13	30.00	
21	HLA-A0201	AEVKHLHHL	AEVKHLHHL	0	0	0	0	0	AEVKHLHHL	Sequence	0.076	21909.21	30.00	
7	HLA-A0201	PVIFQIALDK	VIFQIALDK	1	0	0	0	0	VIFQIALDK	Sequence	0.073	22753.57	32.00	
8	HLA-A0201	VIFQIALD	VIFQIALD-	0	8	1	0	0	VIFQIALD	Sequence	0.069	23794.68	33.00	
10	HLA-A0201	FQIALDKPCH	FQIALDKPC	0	0	0	0	0	FQIALDKPC	Sequence	0.067	24113.97	34.00	
20	HLA-A0201	QAEVKHLHHL	QAVKHLHHL	0	0	0	2	1	QAEVKHLHHL	Sequence	0.065	24658.02	35.00	
0	HLA-A0201	MVSGIISP	-MVSGIISP	0	0	1	0	0	MVSGIISP	Sequence	0.065	24846.83	35.00	
54	HLA-A0201	LSKLQAAVNA	LSLQAAVNA	0	0	0	2	1	LSKLQAAVNA	Sequence	0.064	25050.08	36.00	
3	HLA-A0201	GIISPVIF	GIISPVIF-	0	8	1	0	0	GIISPVIF	Sequence	0.064	25063.09	36.00	

27	HLA-A0201	HHLLKQLKPS	HLLKQLKPS	0	0	0	1	1	HHLLKQLKPS	Sequence	0.064	25091.04	36.00
7	HLA-A0201	PVIFQIAL	-PVIFQIAL	0	0	1	0	0	PVIFQIAL	Sequence	0.064	25146.21	36.00
37	HLA-A0201	EKYLKIKHLL	EKYLKIHLL	0	0	0	6	1	EKYLKIKHLL	Sequence	0.063	25418.94	36.00
21	HLA-A0201	AEVKHLHHLL	AVKHLHHLL	0	0	0	1	1	AEVKHLHHLL	Sequence	0.062	25446.74	37.00
52	HLA-A0201	VDLSKLQAA	VDLSKLQAA	0	0	0	0	0	VDLSKLQAA	Sequence	0.062	25456.92	37.00
40	HLA-A0201	LKIKHLLLK	LKIKHLLLK	0	0	0	0	0	LKIKHLLLK	Sequence	0.059	26327.84	38.00
34	HLA-A0201	KPSEKYLKI	KPSEKYLKI	0	0	0	0	0	KPSEKYLKI	Sequence	0.059	26428.58	38.00
36	HLA-A0201	SEKYLKIKHL	SEYLKIKHL	0	0	0	2	1	SEKYLKIKHL	Sequence	0.058	26664.10	39.00
55	HLA-A0201	SKLQAAVNA	SKLQAAVNA	0	0	0	0	0	SKLQAAVNA	Sequence	0.057	27049.12	40.00
37	HLA-A0201	EKYLKIKHL	EKYLKIKHL	0	0	0	0	0	EKYLKIKHL	Sequence	0.055	27595.40	41.00
6	HLA-A0201	SPVIFQIA	SPVIFQI-A	0	7	1	0	0	SPVIFQIA	Sequence	0.054	27770.04	41.00
50	HLA-A0201	ERVDLSKLQA	RVDLSKLQA	1	0	0	0	0	RVDLSKLQA	Sequence	0.053	28285.86	42.00
41	HLA-A0201	KIKHLLLKR	KIKHLLLKR	0	0	0	0	0	KIKHLLLKR	Sequence	0.052	28591.09	43.00
19	HLA-A0201	HQAEVKHL	HQAEVK-HL	0	6	1	0	0	HQAEVKHL	Sequence	0.051	28655.81	43.00
28	HLA-A0201	HLLKQLKPSE	HLLKQLPSE	0	0	0	6	1	HLLKQLKPSE	Sequence	0.051	28813.44	44.00
38	HLA-A0201	KYLKIKHL	K-YLKIKHL	0	1	1	0	0	KYLKIKHL	Sequence	0.050	29036.26	44.00
9	HLA-A0201	IFQIALDK	-IFQIALDK	0	0	1	0	0	IFQIALDK	Sequence	0.050	29176.40	45.00
22	HLA-A0201	EVKHLHHLL	EVKHLHHLL	0	0	0	0	0	EVKHLHHLL	Sequence	0.048	29768.24	46.00
9	HLA-A0201	IFQIALDKP	IFQIALDKP	0	0	0	0	0	IFQIALDKP	Sequence	0.046	30543.44	48.00
53	HLA-A0201	DLSKLQAA	DL-SKLQAA	0	2	1	0	0	DLSKLQAA	Sequence	0.045	30587.42	48.00
32	HLA-A0201	QLKPSEKYLK	QLKPSEKYL	0	0	0	0	0	QLKPSEKYL	Sequence	0.044	30935.56	49.00
48	HLA-A0201	KRERVDLSKL	KRERVDLSL	0	0	0	8	1	KRERVDLSKL	Sequence	0.044	30973.07	49.00
14	HLA-A0201	LDKPCHQAEV	LDPCHQAEV	0	0	0	2	1	LDKPCHQAEV	Sequence	0.044	31001.57	49.00
40	HLA-A0201	LKIKHLLLKR	LIKHLLLKR	0	0	0	1	1	LKIKHLLLKR	Sequence	0.044	31073.42	50.00
25	HLA-A0201	HLHHLLKQ	HL-HHLLKQ	0	2	1	0	0	HLHHLLKQ	Sequence	0.042	31627.33	55.00
29	HLA-A0201	LLKQLKPSEK	LLQLKPSEK	0	0	0	2	1	LLKQLKPSEK	Sequence	0.042	31659.86	55.00
10	HLA-A0201	FQIALDKP	FQIALDKP-	0	8	1	0	0	FQIALDKP	Sequence	0.042	31670.15	55.00
2	HLA-A0201	SGIISPVI	S-GIISPVI	0	1	1	0	0	SGIISPVI	Sequence	0.042	31745.95	55.00
49	HLA-A0201	RERVDLSKL	RERVDLSKL	0	0	0	0	0	RERVDLSKL	Sequence	0.042	31880.89	55.00
33	HLA-A0201	LKPSEKYL	-LKPSEKYL	0	0	1	0	0	LKPSEKYL	Sequence	0.041	32030.61	55.00
41	HLA-A0201	KIKHLLLK	KI-KHLLLK	0	2	1	0	0	KIKHLLLK	Sequence	0.041	32129.17	55.00
23	HLA-A0201	VKHLHHLLK	VKHLHHLLK	0	0	0	0	0	VKHLHHLLK	Sequence	0.040	32440.77	55.00
13	HLA-A0201	ALDKPCHQ	ALDKPCHQ-	0	8	1	0	0	ALDKPCHQ	Sequence	0.040	32443.22	55.00
18	HLA-A0201	CHQAEVKHL	CHQAEVKHL	0	0	0	0	0	CHQAEVKHL	Sequence	0.040	32480.80	55.00
41	HLA-A0201	KIKHLLLKRE	KIKHLLLRE	0	0	0	7	1	KIKHLLLKRE	Sequence	0.040	32520.18	55.00
31	HLA-A0201	KQLKPSEKY	KQLKPSEKY	0	0	0	0	0	KQLKPSEKY	Sequence	0.039	32689.16	55.00
16	HLA-A0201	KPCHQAEV	K-PCHQAEV	0	1	1	0	0	KPCHQAEV	Sequence	0.039	32791.54	55.00
29	HLA-A0201	LLKQLKPS	LL-KQLKPS	0	2	1	0	0	LLKQLKPS	Sequence	0.038	33012.26	55.00
42	HLA-A0201	IKHLLLKRE	IKHLLLKRE	0	0	0	0	0	IKHLLLKRE	Sequence	0.038	33043.36	55.00
12	HLA-A0201	IALDKPCHQ	IALDKPCHQ	0	0	0	0	0	IALDKPCHQ	Sequence	0.038	33243.10	60.00
19	HLA-A0201	HQAEVKHLH	HQAEVKHLH	0	0	0	0	0	HQAEVKHLH	Sequence	0.037	33373.55	60.00
33	HLA-A0201	LKPSEKYLKI	LPSEKYLKI	0	0	0	1	1	LKPSEKYLKI	Sequence	0.037	33395.23	60.00
26	HLA-A0201	LHHLLKQLK	LHHLLKQLK	0	0	0	0	0	LHHLLKQLK	Sequence	0.036	33852.16	60.00
28	HLA-A0201	HLLKQLKP	HLLKQLKP-	0	8	1	0	0	HLLKQLKP	Sequence	0.036	33924.76	60.00
19	HLA-A0201	HQAEVKHLHH	HQAEVHLHH	0	0	0	5	1	HQAEVKHLHH	Sequence	0.036	34028.79	60.00
29	HLA-A0201	LLKQLKPSE	LLKQLKPSE	0	0	0	0	0	LLKQLKPSE	Sequence	0.035	34083.68	60.00
42	HLA-A0201	IKHLLLKRER	IKHLLLRER	0	0	0	6	1	IKHLLLKRER	Sequence	0.034	34758.88	65.00
7	HLA-A0201	PVIFQIALD	PVIFQIALD	0	0	0	0	0	PVIFQIALD	Sequence	0.034	34767.17	65.00
24	HLA-A0201	KHLHHLLKQ	KHLHHLLKQ	0	0	0	0	0	KHLHHLLKQ	Sequence	0.033	35007.62	65.00
42	HLA-A0201	IKHLLLKR	IKHL-LLKR	0	4	1	0	0	IKHLLLKR	Sequence	0.032	35306.61	65.00
45	HLA-A0201	LLLKRERVD	LLLKRERVD	0	0	0	0	0	LLLKRERVD	Sequence	0.032	35444.77	65.00
23	HLA-A0201	VKHLHHLL	-VKHLHHLL	0	0	1	0	0	VKHLHHLL	Sequence	0.031	35595.43	65.00
23	HLA-A0201	VKHLHHLLKQ	VKHLHLLKQ	0	0	0	4	1	VKHLHHLLKQ	Sequence	0.031	35809.81	65.00
47	HLA-A0201	LKRERVDLSK	LKREVDLSK	0	0	0	4	1	LKRERVDLSK	Sequence	0.031	35820.28	65.00
18	HLA-A0201	CHQAEVKHLH	CQAEVKHLH	0	0	0	1	1	CHQAEVKHLH	Sequence	0.031	35829.59	65.00
44	HLA-A0201	HLLLKRER	HLLLKRER-	0	8	1	0	0	HLLLKRER	Sequence	0.031	35870.70	65.00
47	HLA-A0201	LKRERVDLS	LKRERVDLS	0	0	0	0	0	LKRERVDLS	Sequence	0.030	36171.97	70.00
48	HLA-A0201	KRERVDLSK	KRERVDLSK	0	0	0	0	0	KRERVDLSK	Sequence	0.029	36339.87	70.00
22	HLA-A0201	EVKHLHHL	EV-KHLHHL	0	2	1	0	0	EVKHLHHL	Sequence	0.029	36402.43	70.00
52	HLA-A0201	VDLSKLQA	-VDLSKLQA	0	0	1	0	0	VDLSKLQA	Sequence	0.029	36565.86	70.00
22	HLA-A0201	EVKHLHHLLK	EVHLHHLLK	0	0	0	2	1	EVKHLHHLLK	Sequence	0.029	36632.00	70.00
17	HLA-A0201	PCHQAEVKHL	CHQAEVKHL	1	0	0	0	0	CHQAEVKHL	Sequence	0.028	36920.07	70.00
30	HLA-A0201	LKQLKPSEKY	LQLKPSEKY	0	0	0	1	1	LKQLKPSEKY	Sequence	0.028	36951.25	70.00
58	HLA-A0201	QAAVNAAVQ	QAAVNAAVQ	0	0	0	0	0	QAAVNAAVQ	Sequence	0.028	36989.66	70.00
47	HLA-A0201	LKRERVDL	-LKRERVDL	0	0	1	0	0	LKRERVDL	Sequence	0.028	37066.98	70.00
34	HLA-A0201	KPSEKYLKIK	KPSEKYLKI	0	0	0	0	0	KPSEKYLKI	Sequence	0.027	37142.86	70.00
20	HLA-A0201	QAEVKHLHH	QAEVKHLHH	0	0	0	0	0	QAEVKHLHH	Sequence	0.027	37394.07	75.00
49	HLA-A0201	RERVDLSKLQ	RERVDLSKL	0	0	0	0	0	RERVDLSKL	Sequence	0.027	37490.90	75.00
26	HLA-A0201	LHHLLKQLKP	LHHLLQLKP	0	0	0	5	1	LHHLLKQLKP	Sequence	0.026	37593.65	75.00
11	HLA-A0201	QIALDKPCHQ	QIALKPCHQ	0	0	0	4	1	QIALDKPCHQ	Sequence	0.026	37851.21	75.00
32	HLA-A0201	QLKPSEKY	QL-KPSEKY	0	2	1	0	0	QLKPSEKY	Sequence	0.025	37961.53	75.00
54	HLA-A0201	LSKLQAAVN	LSKLQAAVN	0	0	0	0	0	LSKLQAAVN	Sequence	0.024	38399.39	75.00
43	HLA-A0201	KHLLLKRE	K-HLLLKRE	0	1	1	0	0	KHLLLKRE	Sequence	0.024	38763.41	80.00
43	HLA-A0201	KHLLLKRER	KHLLLKRER	0	0	0	0	0	KHLLLKRER	Sequence	0.023	38791.09	80.00
49	HLA-A0201	RERVDLSK	R-ERVDLSK	0	1	1	0	0	RERVDLSK	Sequence	0.023	38986.34	80.00
15	HLA-A0201	DKPCHQAEV	DKPCHQAEV	0	0	0	0	0	DKPCHQAEV	Sequence	0.023	39086.86	80.00
27	HLA-A0201	HHLLKQLKP	HHLLKQLKP	0	0	0	0	0	HHLLKQLKP	Sequence	0.022	39341.43	80.00
35	HLA-A0201	PSEKYLKIK	PSEKYLKIK	0	0	0	0	0	PSEKYLKIK	Sequence	0.022	39425.39	80.00
24	HLA-A0201	KHLHHLLK	K-HLHHLLK	0	1	1	0	0	KHLHHLLK	Sequence	0.022	39430.94	80.00
50	HLA-A0201	ERVDLSKL	-ERVDLSKL	0	0	1	0	0	ERVDLSKL	Sequence	0.022	39536.02	80.00
36	HLA-A0201	SEKYLKIKH	SEKYLKIKH	0	0	0	0	0	SEKYLKIKH	Sequence	0.021	39686.87	85.00
51	HLA-A0201	RVDLSKLQ	RVDLSKLQ-	0	8	1	0	0	RVDLSKLQ	Sequence	0.021	39845.21	85.00
35	HLA-A0201	PSEKYLKIKH	PSEKYLIKH	0	0	0	6	1	PSEKYLKIKH	Sequence	0.021	39883.62	85.00
35	HLA-A0201	PSEKYLKI	-PSEKYLKI	0	0	1	0	0	PSEKYLKI	Sequence	0.021	39900.44	85.00
11	HLA-A0201	QIALDKPC	-QIALDKPC	0	0	1	0	0	QIALDKPC	Sequence	0.020	40149.89	85.00
16	HLA-A0201	KPCHQAEVK	KPCHQAEVK	0	0	0	0	0	KPCHQAEVK	Sequence	0.020	40156.83	85.00
30	HLA-A0201	LKQLKPSEK	LKQLKPSEK	0	0	0	0	0	LKQLKPSEK	Sequence	0.020	40219.01	85.00
37	HLA-A0201	EKYLKIKH	EKYLKIKH-	0	8	1	0	0	EKYLKIKH	Sequence	0.019	40511.19	85.00
16	HLA-A0201	KPCHQAEVKH	KCHQAEVKH	0	0	0	1	1	KPCHQAEVKH	Sequence	0.019	40856.73	90.00
12	HLA-A0201	IALDKPCH	IALDKPCH-	0	8	1	0	0	IALDKPCH	Sequence	0.018	40952.79	90.00

18	HLA-A0201	CHQAEVKH	CHQAEVKH-	0	8	1	0	0	CHQAEVKH	Sequence	0.018	41064.60	90.00
14	HLA-A0201	LDKPCHQAE	LDKPCHQAE	0	0	0	0	0	LDKPCHQAE	Sequence	0.018	41115.72	90.00
48	HLA-A0201	KRERVDLS	KRERVDLS-	0	8	1	0	0	KRERVDLS	Sequence	0.018	41341.00	90.00
17	HLA-A0201	PCHQAEVKH	PCHQAEVKH	0	0	0	0	0	PCHQAEVKH	Sequence	0.017	41379.04	90.00
27	HLA-A0201	HHLLKQLK	H-HLLKQLK	0	1	1	0	0	HHLLKQLK	Sequence	0.017	41386.64	90.00
59	HLA-A0201	AAVNAAVQ	AAVNAAVQ-	0	8	1	0	0	AAVNAAVQ	Sequence	0.017	41394.25	90.00
20	HLA-A0201	QAEVKHLH	QAEVKHLH-	0	8	1	0	0	QAEVKHLH	Sequence	0.017	41520.74	90.00
36	HLA-A0201	SEKYLKIK	-SEKYLKIK	0	0	1	0	0	SEKYLKIK	Sequence	0.017	41536.04	90.00
11	HLA-A0201	QIALDKPCH	QIALDKPCH	0	0	0	0	0	QIALDKPCH	Sequence	0.017	41590.90	90.00
21	HLA-A0201	AEVKHLHH	A-EVKHLHH	0	1	1	0	0	AEVKHLHH	Sequence	0.016	42054.71	90.00
55	HLA-A0201	SKLQAAVN	SKLQAAVN-	0	8	1	0	0	SKLQAAVN	Sequence	0.016	42233.46	95.00
30	HLA-A0201	LKQLKPSE	-LKQLKPSE	0	0	1	0	0	LKQLKPSE	Sequence	0.015	42491.50	95.00
34	HLA-A0201	KPSEKYLK	KPSEKYLK-	0	8	1	0	0	KPSEKYLK	Sequence	0.015	42517.73	95.00
15	HLA-A0201	DKPCHQAEVK	DKPCHQAEV	0	0	0	0	0	DKPCHQAEV	Sequence	0.015	42584.94	95.00
50	HLA-A0201	ERVDLSKLQ	ERVDLSKLQ	0	0	0	0	0	ERVDLSKLQ	Sequence	0.015	42605.68	95.00
31	HLA-A0201	KQLKPSEK	KQLKPSEK-	0	8	1	0	0	KQLKPSEK	Sequence	0.014	42861.37	95.00
46	HLA-A0201	LLKRERVD	LLKRERVD-	0	8	1	0	0	LLKRERVD	Sequence	0.014	43026.32	95.00
33	HLA-A0201	LKPSEKYLK	LKPSEKYLK	0	0	0	0	0	LKPSEKYLK	Sequence	0.013	43307.02	95.00
17	HLA-A0201	PCHQAEVK	-PCHQAEVK	0	0	1	0	0	PCHQAEVK	Sequence	0.012	43804.68	99.00
15	HLA-A0201	DKPCHQAE	DKPCHQAE-	0	8	1	0	0	DKPCHQAE	Sequence	0.010	44801.20	99.00

Protein Sequence. Allele HLA-A0201. Number of high binders 1. Number of weak binders 4. Number of peptides 177

Link to Allele Frequencies in Worldwide Populations HLA-A0201

Rank Threshold for Strong binding peptides 0.500
Rank Threshold for Weak binding peptides 2.000

---

pos	HLA	peptide	Core	Offset	I_pos	I_len	D_pos	D_len	iCore	Identity	1-log50k(aff)	Affinity(nM)	%Rank	BindLeve
39	HLA-A0301	YLKIKHLLLK	YLKKHLLLK	0	0	0	3	1	YLKIKHLLLK	Sequence	0.700	25.74	0.12	<= SB
8	HLA-A0301	VIFQIALDK	VIFQIALDK	0	0	0	0	0	VIFQIALDK	Sequence	0.632	53.67	0.25	<= SB
25	HLA-A0301	HLHHLLKQLK	HLHHLLQLK	0	0	0	6	1	HLHHLLKQLK	Sequence	0.627	56.48	0.30	<= SB
41	HLA-A0301	KIKHLLLKR	KIKHLLLKR	0	0	0	0	0	KIKHLLLKR	Sequence	0.489	252.67	0.90	<= WB
41	HLA-A0301	KIKHLLLK	KI-KHLLLK	0	2	1	0	0	KIKHLLLK	Sequence	0.486	260.29	0.90	<= WB
29	HLA-A0301	LLKQLKPSEK	LLKLKPSEK	0	0	0	3	1	LLKQLKPSEK	Sequence	0.448	392.73	1.10	<= WB
32	HLA-A0301	QLKPSEKYLK	QLKPSKYLK	0	0	0	5	1	QLKPSEKYLK	Sequence	0.444	410.02	1.20	<= WB
7	HLA-A0301	PVIFQIALDK	VIFQIALDK	1	0	0	0	0	VIFQIALDK	Sequence	0.435	450.78	1.20	<= WB
22	HLA-A0301	EVKHLHHLLK	EVKHHHLLK	0	0	0	4	1	EVKHLHHLLK	Sequence	0.405	626.17	1.50	<= WB
8	HLA-A0301	VIFQIALDKP	VIFQIALDK	0	0	0	0	0	VIFQIALDK	Sequence	0.378	833.69	1.80	<= WB
40	HLA-A0301	LKIKHLLLKR	KIKHLLLKR	1	0	0	0	0	KIKHLLLKR	Sequence	0.287	2248.31	3.50	
24	HLA-A0301	KHLHHLLK	K-HLHHLLK	0	1	1	0	0	KHLHHLLK	Sequence	0.250	3352.00	4.00	
23	HLA-A0301	VKHLHHLLK	VKHLHHLLK	0	0	0	0	0	VKHLHHLLK	Sequence	0.243	3624.51	4.50	
34	HLA-A0301	KPSEKYLKIK	KSEKYLKIK	0	0	0	1	1	KPSEKYLKIK	Sequence	0.239	3779.66	4.50	
40	HLA-A0301	LKIKHLLLK	LKIKHLLLK	0	0	0	0	0	LKIKHLLLK	Sequence	0.237	3862.88	4.50	
41	HLA-A0301	KIKHLLLKRE	KIKHLLLKR	0	0	0	0	0	KIKHLLLKR	Sequence	0.234	3954.48	4.50	
31	HLA-A0301	KQLKPSEKY	KQLKPSEKY	0	0	0	0	0	KQLKPSEKY	Sequence	0.221	4571.52	5.00	
31	HLA-A0301	KQLKPSEK	KQLKP-SEK	0	5	1	0	0	KQLKPSEK	Sequence	0.207	5304.89	5.50	
9	HLA-A0301	IFQIALDK	-IFQIALDK	0	0	1	0	0	IFQIALDK	Sequence	0.205	5437.50	5.50	
38	HLA-A0301	KYLKIKHLLL	KLKIKHLLL	0	0	0	1	1	KYLKIKHLLL	Sequence	0.173	7720.42	7.00	
56	HLA-A0301	KLQAAVNAAV	KLAAVNAAV	0	0	0	2	1	KLQAAVNAAV	Sequence	0.171	7832.41	7.00	
0	HLA-A0301	MVSGIISPV	MVSGIISPV	0	0	0	0	0	MVSGIISPV	Sequence	0.159	8989.62	8.00	
26	HLA-A0301	LHHLLKQLK	LHHLLKQLK	0	0	0	0	0	LHHLLKQLK	Sequence	0.154	9489.96	8.00	
4	HLA-A0301	IISPVIFQI	IISPVIFQI	0	0	0	0	0	IISPVIFQI	Sequence	0.146	10320.60	9.00	
19	HLA-A0301	HQAEVKHLHH	HQAEVHLHH	0	0	0	5	1	HQAEVKHLHH	Sequence	0.131	12090.56	10.00	
3	HLA-A0301	GIISPVIFQ	GIISPVIFQ	0	0	0	0	0	GIISPVIFQ	Sequence	0.131	12131.58	10.00	
3	HLA-A0301	GIISPVIFQI	GIISPVIFI	0	0	0	8	1	GIISPVIFQI	Sequence	0.128	12490.80	11.00	
39	HLA-A0301	YLKIKHLLL	YLKIKHLLL	0	0	0	0	0	YLKIKHLLL	Sequence	0.127	12716.21	11.00	
16	HLA-A0301	KPCHQAEVK	KPCHQAEVK	0	0	0	0	0	KPCHQAEVK	Sequence	0.125	12964.32	11.00	
0	HLA-A0301	MVSGIISPVI	MVSGIISVI	0	0	0	7	1	MVSGIISPVI	Sequence	0.124	13123.52	11.00	
23	HLA-A0301	VKHLHHLLKQ	VKHLHHLLK	0	0	0	0	0	VKHLHHLLK	Sequence	0.116	14253.84	12.00	
56	HLA-A0301	KLQAAVNAA	KLQAAVNAA	0	0	0	0	0	KLQAAVNAA	Sequence	0.116	14282.55	12.00	
47	HLA-A0301	LKRERVDLSK	LKRERVLSK	0	0	0	6	1	LKRERVDLSK	Sequence	0.113	14762.54	13.00	
19	HLA-A0301	HQAEVKHLH	HQAEVKHLH	0	0	0	0	0	HQAEVKHLH	Sequence	0.112	14815.03	13.00	
4	HLA-A0301	IISPVIFQIA	IISPVIFQA	0	0	0	8	1	IISPVIFQIA	Sequence	0.112	14861.10	13.00	
51	HLA-A0301	RVDLSKLQA	RVDLSKLQA	0	0	0	0	0	RVDLSKLQA	Sequence	0.104	16202.31	14.00	
2	HLA-A0301	SGIISPVIFQ	SIISPVIFQ	0	0	0	1	1	SGIISPVIFQ	Sequence	0.103	16367.41	14.00	
30	HLA-A0301	LKQLKPSEKY	KQLKPSEKY	1	0	0	0	0	KQLKPSEKY	Sequence	0.103	16469.73	14.00	
25	HLA-A0301	HLHHLLKQL	HLHHLLKQL	0	0	0	0	0	HLHHLLKQL	Sequence	0.098	17258.01	15.00	
51	HLA-A0301	RVDLSKLQAA	RVLSKLQAA	0	0	0	2	1	RVDLSKLQAA	Sequence	0.095	17984.90	16.00	
52	HLA-A0301	VDLSKLQAAV	VLSKLQAAV	0	0	0	1	1	VDLSKLQAAV	Sequence	0.094	18024.44	16.00	
49	HLA-A0301	RERVDLSK	RER-VDLSK	0	3	1	0	0	RERVDLSK	Sequence	0.093	18298.56	16.00	
27	HLA-A0301	HHLLKQLK	HHLLKQL-K	0	7	1	0	0	HHLLKQLK	Sequence	0.093	18347.92	16.00	
24	HLA-A0301	KHLHHLLKQL	KLHHLLKQL	0	0	0	1	1	KHLHHLLKQL	Sequence	0.089	19033.41	17.00	
34	HLA-A0301	KPSEKYLK	KPSEKYL-K	0	7	1	0	0	KPSEKYLK	Sequence	0.084	20161.57	19.00	
31	HLA-A0301	KQLKPSEKYL	KQLKPSEKY	0	0	0	0	0	KQLKPSEKY	Sequence	0.083	20265.45	19.00	
21	HLA-A0301	AEVKHLHHLL	AVKHLHHLL	0	0	0	1	1	AEVKHLHHLL	Sequence	0.082	20565.42	19.00	
5	HLA-A0301	ISPVIFQIAL	ISVIFQIAL	0	0	0	2	1	ISPVIFQIAL	Sequence	0.082	20604.84	19.00	
44	HLA-A0301	HLLLKRERV	HLLLKRERV	0	0	0	0	0	HLLLKRERV	Sequence	0.081	20806.01	20.00	
48	HLA-A0301	KRERVDLSK	KRERVDLSK	0	0	0	0	0	KRERVDLSK	Sequence	0.080	21098.69	20.00	
57	HLA-A0301	LQAAVNAAV	LQAAVNAAV	0	0	0	0	0	LQAAVNAAV	Sequence	0.077	21633.13	21.00	
43	HLA-A0301	KHLLLKRER	KHLLLKRER	0	0	0	0	0	KHLLLKRER	Sequence	0.074	22522.59	22.00	
44	HLA-A0301	HLLLKRER	HLLLKR-ER	0	6	1	0	0	HLLLKRER	Sequence	0.073	22764.40	23.00	
43	HLA-A0301	KHLLLKRERV	KLLLKRERV	0	0	0	1	1	KHLLLKRERV	Sequence	0.072	22853.24	23.00	
6	HLA-A0301	SPVIFQIALD	SVIFQIALD	0	0	0	1	1	SPVIFQIALD	Sequence	0.072	22940.20	23.00	
42	HLA-A0301	IKHLLLKR	-IKHLLLKR	0	0	1	0	0	IKHLLLKR	Sequence	0.071	23178.47	23.00	
11	HLA-A0301	QIALDKPCH	QIALDKPCH	0	0	0	0	0	QIALDKPCH	Sequence	0.071	23205.31	23.00	
35	HLA-A0301	PSEKYLKIK	PSEKYLKIK	0	0	0	0	0	PSEKYLKIK	Sequence	0.071	23258.35	23.00	
26	HLA-A0301	LHHLLKQLKP	LHHLLKQLK	0	0	0	0	0	LHHLLKQLK	Sequence	0.070	23338.77	24.00	

38	HLA-A0301	KYLKIKHLL	KYLKIKHLL	0	0	0	0	0	KYLKIKHLL	Sequence	0.070	23464.36	24.00
1	HLA-A0301	VSGIISPVIF	VSGIISPVF	0	0	0	8	1	VSGIISPVIF	Sequence	0.069	23722.43	24.00
45	HLA-A0301	LLLKRERVDL	LLLKRRVDL	0	0	0	5	1	LLLKRERVDL	Sequence	0.068	23840.80	24.00
13	HLA-A0301	ALDKPCHQA	ALDKPCHQA	0	0	0	0	0	ALDKPCHQA	Sequence	0.068	23881.07	24.00
3	HLA-A0301	GIISPVIF	GIISPVIF-	0	8	1	0	0	GIISPVIF	Sequence	0.068	23961.05	25.00
15	HLA-A0301	DKPCHQAEVK	KPCHQAEVK	1	0	0	0	0	KPCHQAEVK	Sequence	0.068	24014.00	25.00
1	HLA-A0301	VSGIISPVI	VSGIISPVI	0	0	0	0	0	VSGIISPVI	Sequence	0.065	24792.31	26.00
28	HLA-A0301	HLLKQLKPS	HLLKQLKPS	0	0	0	0	0	HLLKQLKPS	Sequence	0.063	25284.81	27.00
30	HLA-A0301	LKQLKPSEK	LKQLKPSEK	0	0	0	0	0	LKQLKPSEK	Sequence	0.062	25692.96	28.00
34	HLA-A0301	KPSEKYLKI	KPSEKYLKI	0	0	0	0	0	KPSEKYLKI	Sequence	0.061	25712.70	28.00
6	HLA-A0301	SPVIFQIAL	SPVIFQIAL	0	0	0	0	0	SPVIFQIAL	Sequence	0.061	25953.37	28.00
28	HLA-A0301	HLLKQLKPSE	HLLKQLPSE	0	0	0	6	1	HLLKQLKPSE	Sequence	0.061	25957.29	28.00
32	HLA-A0301	QLKPSEKY	QL-KPSEKY	0	2	1	0	0	QLKPSEKY	Sequence	0.060	26189.18	29.00
4	HLA-A0301	IISPVIFQ	IISPVIFQ-	0	8	1	0	0	IISPVIFQ	Sequence	0.059	26443.45	29.00
49	HLA-A0301	RERVDLSKL	RERVDLSKL	0	0	0	0	0	RERVDLSKL	Sequence	0.058	26679.11	30.00
53	HLA-A0301	DLSKLQAAV	DLSKLQAAV	0	0	0	0	0	DLSKLQAAV	Sequence	0.058	26739.50	30.00
22	HLA-A0301	EVKHLHHLL	EVKHLHHLL	0	0	0	0	0	EVKHLHHLL	Sequence	0.057	26918.62	30.00
10	HLA-A0301	FQIALDKPCH	FIALDKPCH	0	0	0	1	1	FQIALDKPCH	Sequence	0.056	27188.19	31.00
16	HLA-A0301	KPCHQAEVKH	KPCHQAEVK	0	0	0	0	0	KPCHQAEVK	Sequence	0.056	27243.26	31.00
32	HLA-A0301	QLKPSEKYL	QLKPSEKYL	0	0	0	0	0	QLKPSEKYL	Sequence	0.056	27320.61	31.00
18	HLA-A0301	CHQAEVKHLH	CQAEVKHLH	0	0	0	1	1	CHQAEVKHLH	Sequence	0.056	27426.92	32.00
24	HLA-A0301	KHLHHLLKQ	KHLHHLLKQ	0	0	0	0	0	KHLHHLLKQ	Sequence	0.054	27759.81	32.00
2	HLA-A0301	SGIISPVIF	SGIISPVIF	0	0	0	0	0	SGIISPVIF	Sequence	0.054	27794.67	33.00
0	HLA-A0301	MVSGIISP	MVSGIISP-	0	8	1	0	0	MVSGIISP	Sequence	0.054	27845.25	33.00
46	HLA-A0301	LLKRERVDL	LLKRERVDL	0	0	0	0	0	LLKRERVDL	Sequence	0.054	27868.15	33.00
39	HLA-A0301	YLKIKHLL	YLKIKHLL-	0	8	1	0	0	YLKIKHLL	Sequence	0.053	28027.50	33.00
20	HLA-A0301	QAEVKHLHH	QAEVKHLHH	0	0	0	0	0	QAEVKHLHH	Sequence	0.053	28198.45	33.00
55	HLA-A0301	SKLQAAVNAA	KLQAAVNAA	1	0	0	0	0	KLQAAVNAA	Sequence	0.053	28301.15	34.00
50	HLA-A0301	ERVDLSKLQA	RVDLSKLQA	1	0	0	0	0	RVDLSKLQA	Sequence	0.053	28306.06	34.00
44	HLA-A0301	HLLLKRERVD	HLLLKRERV	0	0	0	0	0	HLLLKRERV	Sequence	0.052	28555.84	34.00
25	HLA-A0301	HLHHLLKQ	HLH-HLLKQ	0	3	1	0	0	HLHHLLKQ	Sequence	0.051	28725.34	35.00
36	HLA-A0301	SEKYLKIK	SEKYLKI-K	0	7	1	0	0	SEKYLKIK	Sequence	0.051	28858.68	35.00
33	HLA-A0301	LKPSEKYLK	LKPSEKYLK	0	0	0	0	0	LKPSEKYLK	Sequence	0.051	28940.29	35.00
40	HLA-A0301	LKIKHLLL	-LKIKHLLL	0	0	1	0	0	LKIKHLLL	Sequence	0.050	28952.82	35.00
5	HLA-A0301	ISPVIFQIA	ISPVIFQIA	0	0	0	0	0	ISPVIFQIA	Sequence	0.050	29172.94	36.00
21	HLA-A0301	AEVKHLHHL	AEVKHLHHL	0	0	0	0	0	AEVKHLHHL	Sequence	0.049	29514.55	37.00
36	HLA-A0301	SEKYLKIKH	SEKYLKIKH	0	0	0	0	0	SEKYLKIKH	Sequence	0.045	30598.33	40.00
42	HLA-A0301	IKHLLLKRER	IHLLLKRER	0	0	0	1	1	IKHLLLKRER	Sequence	0.044	30986.13	41.00
27	HLA-A0301	HHLLKQLKP	HHLLKQLKP	0	0	0	0	0	HHLLKQLKP	Sequence	0.044	31056.96	41.00
12	HLA-A0301	IALDKPCHQA	ILDKPCHQA	0	0	0	1	1	IALDKPCHQA	Sequence	0.044	31072.08	42.00
56	HLA-A0301	KLQAAVNA	KLQAAVNA-	0	8	1	0	0	KLQAAVNA	Sequence	0.044	31088.56	42.00
13	HLA-A0301	ALDKPCHQAE	ALKPCHQAE	0	0	0	2	1	ALDKPCHQAE	Sequence	0.044	31214.96	42.00
37	HLA-A0301	EKYLKIKHLL	KYLKIKHLL	1	0	0	0	0	KYLKIKHLL	Sequence	0.044	31225.78	42.00
48	HLA-A0301	KRERVDLSKL	KRERVDLSK	0	0	0	0	0	KRERVDLSK	Sequence	0.043	31290.71	42.00
29	HLA-A0301	LLKQLKPSE	LLKQLKPSE	0	0	0	0	0	LLKQLKPSE	Sequence	0.042	31739.09	44.00
57	HLA-A0301	LQAAVNAAVQ	LQAAVNAAV	0	0	0	0	0	LQAAVNAAV	Sequence	0.042	31835.05	44.00
8	HLA-A0301	VIFQIALD	VIFQIALD-	0	8	1	0	0	VIFQIALD	Sequence	0.042	31846.76	44.00
5	HLA-A0301	ISPVIFQI	-ISPVIFQI	0	0	1	0	0	ISPVIFQI	Sequence	0.042	31910.92	44.00
7	HLA-A0301	PVIFQIALD	PVIFQIALD	0	0	0	0	0	PVIFQIALD	Sequence	0.041	32012.58	45.00
36	HLA-A0301	SEKYLKIKHL	SEYLKIKHL	0	0	0	2	1	SEKYLKIKHL	Sequence	0.041	32031.29	45.00
51	HLA-A0301	RVDLSKLQ	RVDLSKLQ-	0	8	1	0	0	RVDLSKLQ	Sequence	0.041	32032.33	45.00
27	HLA-A0301	HHLLKQLKPS	HLLKQLKPS	0	0	0	1	1	HHLLKQLKPS	Sequence	0.040	32519.83	46.00
46	HLA-A0301	LLKRERVDLS	LLKRERVLS	0	0	0	7	1	LLKRERVDLS	Sequence	0.040	32551.87	46.00
11	HLA-A0301	QIALDKPCHQ	QIALKPCHQ	0	0	0	4	1	QIALDKPCHQ	Sequence	0.039	32863.29	47.00
28	HLA-A0301	HLLKQLKP	HLLKQLKP-	0	8	1	0	0	HLLKQLKP	Sequence	0.039	32875.73	48.00
7	HLA-A0301	PVIFQIAL	PVIF-QIAL	0	4	1	0	0	PVIFQIAL	Sequence	0.037	33408.24	49.00
33	HLA-A0301	LKPSEKYLKI	KPSEKYLKI	1	0	0	0	0	KPSEKYLKI	Sequence	0.036	33705.96	55.00
54	HLA-A0301	LSKLQAAVNA	LSLQAAVNA	0	0	0	2	1	LSKLQAAVNA	Sequence	0.036	33707.42	55.00
49	HLA-A0301	RERVDLSKLQ	RERVDLSKL	0	0	0	0	0	RERVDLSKL	Sequence	0.036	33796.16	55.00
54	HLA-A0301	LSKLQAAV	-LSKLQAAV	0	0	1	0	0	LSKLQAAV	Sequence	0.036	34011.11	55.00
1	HLA-A0301	VSGIISPV	-VSGIISPV	0	0	1	0	0	VSGIISPV	Sequence	0.035	34101.40	55.00
38	HLA-A0301	KYLKIKHL	K-YLKIKHL	0	1	1	0	0	KYLKIKHL	Sequence	0.035	34320.91	55.00
20	HLA-A0301	QAEVKHLHHL	QAVKHLHHL	0	0	0	2	1	QAEVKHLHHL	Sequence	0.035	34356.18	55.00
45	HLA-A0301	LLLKRERVD	LLLKRERVD	0	0	0	0	0	LLLKRERVD	Sequence	0.033	34921.73	55.00
23	HLA-A0301	VKHLHHLL	-VKHLHHLL	0	0	1	0	0	VKHLHHLL	Sequence	0.032	35376.94	60.00
9	HLA-A0301	IFQIALDKPC	IFQIALKPC	0	0	0	6	1	IFQIALDKPC	Sequence	0.032	35490.45	60.00
12	HLA-A0301	IALDKPCHQ	IALDKPCHQ	0	0	0	0	0	IALDKPCHQ	Sequence	0.032	35506.95	60.00
54	HLA-A0301	LSKLQAAVN	LSKLQAAVN	0	0	0	0	0	LSKLQAAVN	Sequence	0.032	35547.69	60.00
53	HLA-A0301	DLSKLQAAVN	DLSKLQAAV	0	0	0	0	0	DLSKLQAAV	Sequence	0.031	35603.12	60.00
9	HLA-A0301	IFQIALDKP	IFQIALDKP	0	0	0	0	0	IFQIALDKP	Sequence	0.031	35700.33	60.00
45	HLA-A0301	LLLKRERV	LLLKRERV-	0	8	1	0	0	LLLKRERV	Sequence	0.030	36189.19	65.00
10	HLA-A0301	FQIALDKPC	FQIALDKPC	0	0	0	0	0	FQIALDKPC	Sequence	0.029	36557.94	65.00
58	HLA-A0301	QAAVNAAVQ	QAAVNAAVQ	0	0	0	0	0	QAAVNAAVQ	Sequence	0.029	36648.63	65.00
37	HLA-A0301	EKYLKIKHL	EKYLKIKHL	0	0	0	0	0	EKYLKIKHL	Sequence	0.029	36687.13	65.00
14	HLA-A0301	LDKPCHQAEV	LDKPHQAEV	0	0	0	4	1	LDKPCHQAEV	Sequence	0.028	36973.65	65.00
13	HLA-A0301	ALDKPCHQ	ALDKPCHQ-	0	8	1	0	0	ALDKPCHQ	Sequence	0.028	36985.24	65.00
35	HLA-A0301	PSEKYLKIKH	PSEKYLKIK	0	0	0	0	0	PSEKYLKIK	Sequence	0.028	37045.71	65.00
2	HLA-A0301	SGIISPVI	SGI-ISPVI	0	3	1	0	0	SGIISPVI	Sequence	0.028	37125.57	65.00
52	HLA-A0301	VDLSKLQAA	VDLSKLQAA	0	0	0	0	0	VDLSKLQAA	Sequence	0.027	37288.62	70.00
26	HLA-A0301	LHHLLKQL	-LHHLLKQL	0	0	1	0	0	LHHLLKQL	Sequence	0.026	37617.25	70.00
57	HLA-A0301	LQAAVNAA	LQAAVNAA-	0	8	1	0	0	LQAAVNAA	Sequence	0.025	38070.93	70.00
58	HLA-A0301	QAAVNAAV	-QAAVNAAV	0	0	1	0	0	QAAVNAAV	Sequence	0.025	38247.23	70.00
6	HLA-A0301	SPVIFQIA	SPVIFQIA-	0	8	1	0	0	SPVIFQIA	Sequence	0.025	38248.88	70.00
17	HLA-A0301	PCHQAEVK	-PCHQAEVK	0	0	1	0	0	PCHQAEVK	Sequence	0.024	38563.46	75.00
29	HLA-A0301	LLKQLKPS	LLKQLKPS-	0	8	1	0	0	LLKQLKPS	Sequence	0.024	38741.19	75.00
19	HLA-A0301	HQAEVKHL	HQAEVKHL-	0	8	1	0	0	HQAEVKHL	Sequence	0.023	38807.91	75.00
18	HLA-A0301	CHQAEVKHL	CHQAEVKHL	0	0	0	0	0	CHQAEVKHL	Sequence	0.023	39114.78	75.00
21	HLA-A0301	AEVKHLHH	AE-VKHLHH	0	2	1	0	0	AEVKHLHH	Sequence	0.022	39205.90	75.00
22	HLA-A0301	EVKHLHHL	EV-KHLHHL	0	2	1	0	0	EVKHLHHL	Sequence	0.021	39679.58	80.00
12	HLA-A0301	IALDKPCH	IALDKPC-H	0	7	1	0	0	IALDKPCH	Sequence	0.021	39713.93	80.00

55	HLA-A0301	SKLQAAVNA	SKLQAAVNA	0	0	0	0	0	SKLQAAVNA	Sequence	0.020	40119.48	80.00
16	HLA-A0301	KPCHQAEV	KPC-HQAEV	0	3	1	0	0	KPCHQAEV	Sequence	0.020	40254.71	80.00
14	HLA-A0301	LDKPCHQAE	LDKPCHQAE	0	0	0	0	0	LDKPCHQAE	Sequence	0.020	40449.45	85.00
20	HLA-A0301	QAEVKHLH	-QAEVKHLH	0	0	1	0	0	QAEVKHLH	Sequence	0.019	40528.28	85.00
33	HLA-A0301	LKPSEKYL	-LKPSEKYL	0	0	1	0	0	LKPSEKYL	Sequence	0.019	40586.21	85.00
35	HLA-A0301	PSEKYLKI	-PSEKYLKI	0	0	1	0	0	PSEKYLKI	Sequence	0.019	40589.30	85.00
42	HLA-A0301	IKHLLLKRE	IKHLLLKRE	0	0	0	0	0	IKHLLLKRE	Sequence	0.019	40853.65	85.00
52	HLA-A0301	VDLSKLQA	-VDLSKLQA	0	0	1	0	0	VDLSKLQA	Sequence	0.018	41256.09	85.00
47	HLA-A0301	LKRERVDL	-LKRERVDL	0	0	1	0	0	LKRERVDL	Sequence	0.018	41292.72	85.00
11	HLA-A0301	QIALDKPC	QIALDKPC-	0	8	1	0	0	QIALDKPC	Sequence	0.018	41362.47	85.00
50	HLA-A0301	ERVDLSKL	-ERVDLSKL	0	0	1	0	0	ERVDLSKL	Sequence	0.017	41562.99	90.00
14	HLA-A0301	LDKPCHQA	-LDKPCHQA	0	0	1	0	0	LDKPCHQA	Sequence	0.017	41573.34	90.00
53	HLA-A0301	DLSKLQAA	DLSKLQAA-	0	8	1	0	0	DLSKLQAA	Sequence	0.017	41789.38	90.00
59	HLA-A0301	AAVNAAVQ	AAVNAAVQ-	0	8	1	0	0	AAVNAAVQ	Sequence	0.016	41870.82	90.00
46	HLA-A0301	LLKRERVD	LLKRERVD-	0	8	1	0	0	LLKRERVD	Sequence	0.016	41894.40	90.00
43	HLA-A0301	KHLLLKRE	K-HLLLKRE	0	1	1	0	0	KHLLLKRE	Sequence	0.016	42191.43	90.00
17	HLA-A0301	PCHQAEVKH	PCHQAEVKH	0	0	0	0	0	PCHQAEVKH	Sequence	0.016	42222.49	90.00
17	HLA-A0301	PCHQAEVKHL	PCHQAEVHL	0	0	0	7	1	PCHQAEVKHL	Sequence	0.015	42487.83	90.00
15	HLA-A0301	DKPCHQAEV	DKPCHQAEV	0	0	0	0	0	DKPCHQAEV	Sequence	0.015	42499.34	90.00
10	HLA-A0301	FQIALDKP	FQIALDKP-	0	8	1	0	0	FQIALDKP	Sequence	0.015	42553.61	90.00
47	HLA-A0301	LKRERVDLS	LKRERVDLS	0	0	0	0	0	LKRERVDLS	Sequence	0.014	43143.80	95.00
37	HLA-A0301	EKYLKIKH	-EKYLKIKH	0	0	1	0	0	EKYLKIKH	Sequence	0.013	43294.84	95.00
18	HLA-A0301	CHQAEVKH	C-HQAEVKH	0	1	1	0	0	CHQAEVKH	Sequence	0.012	44093.32	99.00
48	HLA-A0301	KRERVDLS	K-RERVDLS	0	1	1	0	0	KRERVDLS	Sequence	0.011	44230.45	99.00
30	HLA-A0301	LKQLKPSE	-LKQLKPSE	0	0	1	0	0	LKQLKPSE	Sequence	0.011	44277.86	99.00
50	HLA-A0301	ERVDLSKLQ	ERVDLSKLQ	0	0	0	0	0	ERVDLSKLQ	Sequence	0.011	44608.67	99.00
55	HLA-A0301	SKLQAAVN	-SKLQAAVN	0	0	1	0	0	SKLQAAVN	Sequence	0.010	44799.24	99.00
15	HLA-A0301	DKPCHQAE	-DKPCHQAE	0	0	1	0	0	DKPCHQAE	Sequence	0.007	46192.73	99.00

Protein Sequence. Allele HLA-A0301. Number of high binders 3. Number of weak binders 7. Number of peptides 177

Link to Allele Frequencies in Worldwide Populations HLA-A0301

Rank Threshold for Strong binding peptides 0.500
Rank Threshold for Weak binding peptides 2.000
----

pos	HLA	peptide	Core	Offset	I_pos	I_len	D_pos	D_len	iCore	Identity	1-log50k(aff)	Affinity(nM)	%Rank	BindLeve
38	HLA-A2402	KYLKIKHLLL	KYLKIHLLL	0	0	0	5	1	KYLKIKHLLL	Sequence	0.631	54.12	0.10	<= SB
38	HLA-A2402	KYLKIKHLL	KYLKIKHLL	0	0	0	0	0	KYLKIKHLL	Sequence	0.630	54.86	0.10	<= SB
37	HLA-A2402	EKYLKIKHLL	KYLKIKHLL	1	0	0	0	0	KYLKIKHLL	Sequence	0.442	419.07	0.70	<= WB
4	HLA-A2402	IISPVIFQI	IISPVIFQI	0	0	0	0	0	IISPVIFQI	Sequence	0.346	1178.28	1.30	<= WB
39	HLA-A2402	YLKIKHLL	-YLKIKHLL	0	0	1	0	0	YLKIKHLL	Sequence	0.244	3584.53	3.00	
38	HLA-A2402	KYLKIKHL	KYLKIKH-L	0	7	1	0	0	KYLKIKHL	Sequence	0.217	4800.03	3.50	
1	HLA-A2402	VSGIISPVIF	VSGIISPVF	0	0	0	8	1	VSGIISPVIF	Sequence	0.206	5373.17	4.00	
1	HLA-A2402	VSGIISPVI	VSGIISPVI	0	0	0	0	0	VSGIISPVI	Sequence	0.197	5952.98	4.00	
39	HLA-A2402	YLKIKHLLL	YLKIKHLLL	0	0	0	0	0	YLKIKHLLL	Sequence	0.170	7959.27	5.00	
5	HLA-A2402	ISPVIFQIAL	ISPVIFQIL	0	0	0	8	1	ISPVIFQIAL	Sequence	0.168	8083.99	5.00	
3	HLA-A2402	GIISPVIFQI	IISPVIFQI	1	0	0	0	0	IISPVIFQI	Sequence	0.163	8555.47	5.50	
4	HLA-A2402	IISPVIFQIA	IISPVIFQI	0	0	0	0	0	IISPVIFQI	Sequence	0.153	9529.99	6.00	
2	HLA-A2402	SGIISPVIF	SGIISPVIF	0	0	0	0	0	SGIISPVIF	Sequence	0.152	9688.02	6.00	
5	HLA-A2402	ISPVIFQI	I-SPVIFQI	0	1	1	0	0	ISPVIFQI	Sequence	0.141	10903.04	6.50	
34	HLA-A2402	KPSEKYLKI	KPSEKYLKI	0	0	0	0	0	KPSEKYLKI	Sequence	0.131	12097.23	7.50	
33	HLA-A2402	LKPSEKYLKI	LKPSKYLKI	0	0	0	4	1	LKPSEKYLKI	Sequence	0.129	12442.64	7.50	
0	HLA-A2402	MVSGIISPVI	MVSGISPVI	0	0	0	4	1	MVSGIISPVI	Sequence	0.115	14393.16	8.50	
24	HLA-A2402	KHLHHLLKQL	KHLHHLLQL	0	0	0	7	1	KHLHHLLKQL	Sequence	0.094	18106.73	11.00	
9	HLA-A2402	IFQIALDKP	IFQIALDKP	0	0	0	0	0	IFQIALDKP	Sequence	0.091	18705.12	12.00	
5	HLA-A2402	ISPVIFQIA	ISPVIFQIA	0	0	0	0	0	ISPVIFQIA	Sequence	0.086	19638.60	13.00	
31	HLA-A2402	KQLKPSEKYL	KQLKPSEYL	0	0	0	7	1	KQLKPSEKYL	Sequence	0.078	21442.28	14.00	
22	HLA-A2402	EVKHLHHLL	EVKHLHHLL	0	0	0	0	0	EVKHLHHLL	Sequence	0.077	21790.05	14.00	
39	HLA-A2402	YLKIKHLLLK	YLKIKHLLL	0	0	0	0	0	YLKIKHLLL	Sequence	0.070	23547.77	16.00	
18	HLA-A2402	CHQAEVKHL	CHQAEVKHL	0	0	0	0	0	CHQAEVKHL	Sequence	0.068	23884.17	16.00	
57	HLA-A2402	LQAAVNAAV	LQAAVNAAV	0	0	0	0	0	LQAAVNAAV	Sequence	0.068	23974.79	16.00	
23	HLA-A2402	VKHLHHLL	V-KHLHHLL	0	1	1	0	0	VKHLHHLL	Sequence	0.062	25449.49	18.00	
0	HLA-A2402	MVSGIISPV	MVSGIISPV	0	0	0	0	0	MVSGIISPV	Sequence	0.055	27670.74	20.00	
21	HLA-A2402	AEVKHLHHLL	AVKHLHHLL	0	0	0	1	1	AEVKHLHHLL	Sequence	0.054	27933.36	21.00	
9	HLA-A2402	IFQIALDKPC	IFQIALDPC	0	0	0	7	1	IFQIALDKPC	Sequence	0.053	28252.20	21.00	
2	HLA-A2402	SGIISPVIFQ	SGIISPVIF	0	0	0	0	0	SGIISPVIF	Sequence	0.052	28534.23	21.00	
31	HLA-A2402	KQLKPSEKY	KQLKPSEKY	0	0	0	0	0	KQLKPSEKY	Sequence	0.049	29423.04	22.00	
8	HLA-A2402	VIFQIALDKP	VFQIALDKP	0	0	0	1	1	VIFQIALDKP	Sequence	0.045	30586.41	24.00	
3	HLA-A2402	GIISPVIF	GIIS-PVIF	0	4	1	0	0	GIISPVIF	Sequence	0.045	30716.09	24.00	
34	HLA-A2402	KPSEKYLKIK	KPSEKYLKI	0	0	0	0	0	KPSEKYLKI	Sequence	0.044	31012.64	25.00	
2	HLA-A2402	SGIISPVI	S-GIISPVI	0	1	1	0	0	SGIISPVI	Sequence	0.044	31205.50	25.00	
56	HLA-A2402	KLQAAVNAAV	KQAAVNAAV	0	0	0	1	1	KLQAAVNAAV	Sequence	0.042	31589.72	26.00	
40	HLA-A2402	LKIKHLLL	L-KIKHLLL	0	1	1	0	0	LKIKHLLL	Sequence	0.042	31647.87	26.00	
35	HLA-A2402	PSEKYLKI	P-SEKYLKI	0	1	1	0	0	PSEKYLKI	Sequence	0.042	31837.46	26.00	
21	HLA-A2402	AEVKHLHHL	AEVKHLHHL	0	0	0	0	0	AEVKHLHHL	Sequence	0.041	32103.82	27.00	
46	HLA-A2402	LLKRERVDL	LLKRERVDL	0	0	0	0	0	LLKRERVDL	Sequence	0.040	32579.00	27.00	
25	HLA-A2402	HLHHLLKQL	HLHHLLKQL	0	0	0	0	0	HLHHLLKQL	Sequence	0.037	33468.64	29.00	
18	HLA-A2402	CHQAEVKHLH	CHQAEVKHL	0	0	0	0	0	CHQAEVKHL	Sequence	0.037	33590.54	29.00	
52	HLA-A2402	VDLSKLQAAV	VLSKLQAAV	0	0	0	1	1	VDLSKLQAAV	Sequence	0.036	33982.79	30.00	
57	HLA-A2402	LQAAVNAAVQ	LQAAVNAAV	0	0	0	0	0	LQAAVNAAV	Sequence	0.035	34082.58	30.00	
19	HLA-A2402	HQAEVKHLH	HQAEVKHLH	0	0	0	0	0	HQAEVKHLH	Sequence	0.034	34578.46	31.00	
24	HLA-A2402	KHLHHLLKQ	KHLHHLLKQ	0	0	0	0	0	KHLHHLLKQ	Sequence	0.034	34586.32	31.00	
43	HLA-A2402	KHLLLKRERV	KHLLLKERV	0	0	0	6	1	KHLLLKRERV	Sequence	0.034	34621.89	31.00	
45	HLA-A2402	LLLKRERVDL	LLLKRERVL	0	0	0	8	1	LLLKRERVDL	Sequence	0.033	34851.15	32.00	
56	HLA-A2402	KLQAAVNAA	KLQAAVNAA	0	0	0	0	0	KLQAAVNAA	Sequence	0.032	35486.60	33.00	
4	HLA-A2402	IISPVIFQ	IISPVIFQ-	0	8	1	0	0	IISPVIFQ	Sequence	0.030	36062.17	35.00	
49	HLA-A2402	RERVDLSKL	RERVDLSKL	0	0	0	0	0	RERVDLSKL	Sequence	0.029	36461.17	36.00	

17	HLA-A2402	PCHQAEVKHL	CHQAEVKHL	1	0	0	0	0	CHQAEVKHL	Sequence	0.029	36654.19	36.00
40	HLA-A2402	LKIKHLLLK	LKIKHLLLK	0	0	0	0	0	LKIKHLLLK	Sequence	0.028	36786.50	37.00
27	HLA-A2402	HHLLKQLKP	HHLLKQLKP	0	0	0	0	0	HHLLKQLKP	Sequence	0.027	37364.95	38.00
53	HLA-A2402	DLSKLQAAV	DLSKLQAAV	0	0	0	0	0	DLSKLQAAV	Sequence	0.026	37648.61	39.00
22	HLA-A2402	EVKHLHHLLK	EVKHLHHLL	0	0	0	0	0	EVKHLHHLL	Sequence	0.026	37702.01	39.00
33	HLA-A2402	LKPSEKYLK	LKPSEKYLK	0	0	0	0	0	LKPSEKYLK	Sequence	0.026	37710.16	39.00
26	HLA-A2402	LHHLLKQLKP	LHHLLKQLP	0	0	0	8	1	LHHLLKQLKP	Sequence	0.026	37775.10	39.00
48	HLA-A2402	KRERVDLSKL	KRERVDLSL	0	0	0	8	1	KRERVDLSKL	Sequence	0.025	37986.17	40.00
7	HLA-A2402	PVIFQIALD	PVIFQIALD	0	0	0	0	0	PVIFQIALD	Sequence	0.025	38088.24	40.00
16	HLA-A2402	KPCHQAEV	K-PCHQAEV	0	1	1	0	0	KPCHQAEV	Sequence	0.025	38235.64	41.00
15	HLA-A2402	DKPCHQAEV	DKPCHQAEV	0	0	0	0	0	DKPCHQAEV	Sequence	0.025	38239.78	41.00
8	HLA-A2402	VIFQIALDK	VIFQIALDK	0	0	0	0	0	VIFQIALDK	Sequence	0.024	38441.82	41.00
20	HLA-A2402	QAEVKHLHHL	QAVKHLHHL	0	0	0	2	1	QAEVKHLHHL	Sequence	0.024	38595.61	42.00
23	HLA-A2402	VKHLHHLLK	VKHLHHLLK	0	0	0	0	0	VKHLHHLLK	Sequence	0.024	38621.08	42.00
1	HLA-A2402	VSGIISPV	V-SGIISPV	0	1	1	0	0	VSGIISPV	Sequence	0.024	38765.93	43.00
41	HLA-A2402	KIKHLLLKR	KIKHLLLKR	0	0	0	0	0	KIKHLLLKR	Sequence	0.023	38975.38	43.00
44	HLA-A2402	HLLLKRERV	HLLLKRERV	0	0	0	0	0	HLLLKRERV	Sequence	0.023	39005.75	43.00
6	HLA-A2402	SPVIFQIAL	SPVIFQIAL	0	0	0	0	0	SPVIFQIAL	Sequence	0.023	39057.28	44.00
40	HLA-A2402	LKIKHLLLKR	LKIKHLLLR	0	0	0	8	1	LKIKHLLLKR	Sequence	0.022	39270.43	44.00
23	HLA-A2402	VKHLHHLLKQ	VHLHHLLKQ	0	0	0	1	1	VKHLHHLLKQ	Sequence	0.022	39312.08	45.00
26	HLA-A2402	LHHLLKQLK	LHHLLKQLK	0	0	0	0	0	LHHLLKQLK	Sequence	0.022	39413.45	45.00
19	HLA-A2402	HQAEVKHLHH	HQAEVHLHH	0	0	0	5	1	HQAEVKHLHH	Sequence	0.022	39480.02	45.00
9	HLA-A2402	IFQIALDK	IFQIALDK-	0	8	1	0	0	IFQIALDK	Sequence	0.022	39524.04	45.00
30	HLA-A2402	LKQLKPSEKY	LQLKPSEKY	0	0	0	1	1	LKQLKPSEKY	Sequence	0.022	39616.96	46.00
6	HLA-A2402	SPVIFQIALD	SVIFQIALD	0	0	0	1	1	SPVIFQIALD	Sequence	0.021	39827.97	46.00
32	HLA-A2402	QLKPSEKYL	QLKPSEKYL	0	0	0	0	0	QLKPSEKYL	Sequence	0.021	39864.61	47.00
8	HLA-A2402	VIFQIALD	VIFQI-ALD	0	5	1	0	0	VIFQIALD	Sequence	0.021	39910.81	47.00
26	HLA-A2402	LHHLLKQL	LHHLLKQ-L	0	7	1	0	0	LHHLLKQL	Sequence	0.021	39922.04	47.00
52	HLA-A2402	VDLSKLQAA	VDLSKLQAA	0	0	0	0	0	VDLSKLQAA	Sequence	0.021	40018.03	47.00
33	HLA-A2402	LKPSEKYL	LKPSEKY-L	0	7	1	0	0	LKPSEKYL	Sequence	0.020	40139.02	48.00
14	HLA-A2402	LDKPCHQAEV	LDPCHQAEV	0	0	0	2	1	LDKPCHQAEV	Sequence	0.020	40329.27	48.00
19	HLA-A2402	HQAEVKHL	HQAEVKH-L	0	7	1	0	0	HQAEVKHL	Sequence	0.019	40519.97	49.00
55	HLA-A2402	SKLQAAVNA	SKLQAAVNA	0	0	0	0	0	SKLQAAVNA	Sequence	0.019	40739.34	50.00
6	HLA-A2402	SPVIFQIA	S-PVIFQIA	0	1	1	0	0	SPVIFQIA	Sequence	0.019	40863.82	55.00
10	HLA-A2402	FQIALDKPC	FQIALDKPC	0	0	0	0	0	FQIALDKPC	Sequence	0.018	40941.69	55.00
45	HLA-A2402	LLLKRERV	LLLKR-ERV	0	5	1	0	0	LLLKRERV	Sequence	0.018	40986.47	55.00
51	HLA-A2402	RVDLSKLQAA	RVLSKLQAA	0	0	0	2	1	RVDLSKLQAA	Sequence	0.018	41021.51	55.00
45	HLA-A2402	LLLKRERVD	LLLKRERVD	0	0	0	0	0	LLLKRERVD	Sequence	0.018	41128.18	55.00
46	HLA-A2402	LLKRERVDLS	LLKRERVDL	0	0	0	0	0	LLKRERVDL	Sequence	0.018	41304.79	55.00
36	HLA-A2402	SEKYLKIKHL	SEKYLIKHL	0	0	0	5	1	SEKYLKIKHL	Sequence	0.017	41467.79	55.00
37	HLA-A2402	EKYLKIKHL	EKYLKIKHL	0	0	0	0	0	EKYLKIKHL	Sequence	0.017	41481.69	55.00
32	HLA-A2402	QLKPSEKYLK	QLPSEKYLK	0	0	0	2	1	QLKPSEKYLK	Sequence	0.017	41560.74	55.00
25	HLA-A2402	HLHHLLKQLK	HLHHLLKQL	0	0	0	0	0	HLHHLLKQL	Sequence	0.016	41869.93	60.00
58	HLA-A2402	QAAVNAAVQ	QAAVNAAVQ	0	0	0	0	0	QAAVNAAVQ	Sequence	0.016	42196.93	60.00
7	HLA-A2402	PVIFQIAL	PVIFQIA-L	0	7	1	0	0	PVIFQIAL	Sequence	0.016	42256.32	60.00
3	HLA-A2402	GIISPVIFQ	GIISPVIFQ	0	0	0	0	0	GIISPVIFQ	Sequence	0.015	42314.87	60.00
22	HLA-A2402	EVKHLHHL	E-VKHLHHL	0	1	1	0	0	EVKHLHHL	Sequence	0.015	42325.41	60.00
54	HLA-A2402	LSKLQAAV	L-SKLQAAV	0	1	1	0	0	LSKLQAAV	Sequence	0.015	42373.97	60.00
41	HLA-A2402	KIKHLLLKRE	KIKHLLLRE	0	0	0	7	1	KIKHLLLKRE	Sequence	0.015	42391.87	60.00
10	HLA-A2402	FQIALDKPCH	FQIALKPCH	0	0	0	5	1	FQIALDKPCH	Sequence	0.015	42429.94	60.00
54	HLA-A2402	LSKLQAAVNA	LSLQAAVNA	0	0	0	2	1	LSKLQAAVNA	Sequence	0.015	42435.47	60.00
55	HLA-A2402	SKLQAAVNAA	SLQAAVNAA	0	0	0	1	1	SKLQAAVNAA	Sequence	0.015	42448.31	60.00
54	HLA-A2402	LSKLQAAVN	LSKLQAAVN	0	0	0	0	0	LSKLQAAVN	Sequence	0.015	42541.18	60.00
29	HLA-A2402	LLKQLKPSE	LLKQLKPSE	0	0	0	0	0	LLKQLKPSE	Sequence	0.015	42572.96	60.00
10	HLA-A2402	FQIALDKP	-FQIALDKP	0	0	1	0	0	FQIALDKP	Sequence	0.014	42801.60	65.00
12	HLA-A2402	IALDKPCHQA	IALKPCHQA	0	0	0	3	1	IALDKPCHQA	Sequence	0.014	42804.83	65.00
53	HLA-A2402	DLSKLQAAVN	DLSKLQAAV	0	0	0	0	0	DLSKLQAAV	Sequence	0.014	42815.02	65.00
24	HLA-A2402	KHLHHLLK	KHLHHLLK-	0	8	1	0	0	KHLHHLLK	Sequence	0.014	42848.38	65.00
49	HLA-A2402	RERVDLSKLQ	RERVDLSKL	0	0	0	0	0	RERVDLSKL	Sequence	0.014	42897.10	65.00
0	HLA-A2402	MVSGIISP	MVSGIISP-	0	8	1	0	0	MVSGIISP	Sequence	0.014	42907.78	65.00
34	HLA-A2402	KPSEKYLK	K-PSEKYLK	0	1	1	0	0	KPSEKYLK	Sequence	0.014	43083.16	65.00
12	HLA-A2402	IALDKPCHQ	IALDKPCHQ	0	0	0	0	0	IALDKPCHQ	Sequence	0.013	43218.55	65.00
41	HLA-A2402	KIKHLLLK	K-IKHLLLK	0	1	1	0	0	KIKHLLLK	Sequence	0.013	43270.47	65.00
58	HLA-A2402	QAAVNAAV	-QAAVNAAV	0	0	1	0	0	QAAVNAAV	Sequence	0.013	43320.15	65.00
14	HLA-A2402	LDKPCHQAE	LDKPCHQAE	0	0	0	0	0	LDKPCHQAE	Sequence	0.013	43368.93	65.00
51	HLA-A2402	RVDLSKLQA	RVDLSKLQA	0	0	0	0	0	RVDLSKLQA	Sequence	0.013	43406.00	65.00
7	HLA-A2402	PVIFQIALDK	VIFQIALDK	1	0	0	0	0	VIFQIALDK	Sequence	0.013	43468.50	65.00
43	HLA-A2402	KHLLLKRER	KHLLLKRER	0	0	0	0	0	KHLLLKRER	Sequence	0.013	43611.73	70.00
15	HLA-A2402	DKPCHQAEVK	DKPCHQAEV	0	0	0	0	0	DKPCHQAEV	Sequence	0.013	43619.27	70.00
42	HLA-A2402	IKHLLLKRE	IKHLLLKRE	0	0	0	0	0	IKHLLLKRE	Sequence	0.012	43689.65	70.00
27	HLA-A2402	HHLLKQLKPS	HHLLKQLKP	0	0	0	0	0	HHLLKQLKP	Sequence	0.012	43725.59	70.00
44	HLA-A2402	HLLLKRERVD	HLLLKRERV	0	0	0	0	0	HLLLKRERV	Sequence	0.012	43935.67	70.00
47	HLA-A2402	LKRERVDL	L-KRERVDL	0	1	1	0	0	LKRERVDL	Sequence	0.011	44162.05	70.00
17	HLA-A2402	PCHQAEVKH	PCHQAEVKH	0	0	0	0	0	PCHQAEVKH	Sequence	0.011	44200.77	75.00
56	HLA-A2402	KLQAAVNA	K-LQAAVNA	0	1	1	0	0	KLQAAVNA	Sequence	0.011	44288.39	75.00
31	HLA-A2402	KQLKPSEK	KQLKPSEK-	0	8	1	0	0	KQLKPSEK	Sequence	0.011	44411.70	75.00
57	HLA-A2402	LQAAVNAA	LQAAVNAA-	0	8	1	0	0	LQAAVNAA	Sequence	0.011	44483.36	75.00
47	HLA-A2402	LKRERVDLS	LKRERVDLS	0	0	0	0	0	LKRERVDLS	Sequence	0.011	44490.59	75.00
11	HLA-A2402	QIALDKPCH	QIALDKPCH	0	0	0	0	0	QIALDKPCH	Sequence	0.011	44497.33	75.00
13	HLA-A2402	ALDKPCHQA	ALDKPCHQA	0	0	0	0	0	ALDKPCHQA	Sequence	0.011	44537.79	75.00
42	HLA-A2402	IKHLLLKR	I-KHLLLKR	0	1	1	0	0	IKHLLLKR	Sequence	0.010	44642.00	75.00
20	HLA-A2402	QAEVKHLH	-QAEVKHLH	0	0	1	0	0	QAEVKHLH	Sequence	0.010	44701.43	75.00
48	HLA-A2402	KRERVDLSK	KRERVDLSK	0	0	0	0	0	KRERVDLSK	Sequence	0.010	44708.21	75.00
28	HLA-A2402	HLLKQLKPS	HLLKQLKPS	0	0	0	0	0	HLLKQLKPS	Sequence	0.010	44899.69	80.00
16	HLA-A2402	KPCHQAEVKH	KCHQAEVKH	0	0	0	1	1	KPCHQAEVKH	Sequence	0.010	44948.30	80.00
28	HLA-A2402	HLLKQLKPSE	HLLKQLPSE	0	0	0	6	1	HLLKQLKPSE	Sequence	0.010	45004.75	80.00
42	HLA-A2402	IKHLLLKRER	IHLLLKRER	0	0	0	1	1	IKHLLLKRER	Sequence	0.010	45098.83	80.00
28	HLA-A2402	HLLKQLKP	HLLK-QLKP	0	4	1	0	0	HLLKQLKP	Sequence	0.009	45186.75	80.00
32	HLA-A2402	QLKPSEKY	Q-LKPSEKY	0	1	1	0	0	QLKPSEKY	Sequence	0.009	45391.08	80.00
13	HLA-A2402	ALDKPCHQAE	ALKPCHQAE	0	0	0	2	1	ALDKPCHQAE	Sequence	0.009	45524.88	85.00

50	HLA-A2402	ERVDLSKLQ	ERVDLSKLQ	0	0	0	0	0	ERVDLSKLQ	Sequence	0.009	45558.39	85.00
30	HLA-A2402	LKQLKPSEK	LKQLKPSEK	0	0	0	0	0	LKQLKPSEK	Sequence	0.009	45592.40	85.00
20	HLA-A2402	QAEVKHLHH	QAEVKHLHH	0	0	0	0	0	QAEVKHLHH	Sequence	0.008	45681.79	85.00
29	HLA-A2402	LLKQLKPSEK	LLKQLKPSK	0	0	0	8	1	LLKQLKPSEK	Sequence	0.008	45706.98	85.00
25	HLA-A2402	HLHHLLKQ	-HLHHLLKQ	0	0	1	0	0	HLHHLLKQ	Sequence	0.008	45780.23	85.00
11	HLA-A2402	QIALDKPCHQ	QIALKPCHQ	0	0	0	4	1	QIALDKPCHQ	Sequence	0.008	45809.47	85.00
52	HLA-A2402	VDLSKLQA	VDLSK-LQA	0	5	1	0	0	VDLSKLQA	Sequence	0.008	45856.58	85.00
16	HLA-A2402	KPCHQAEVK	KPCHQAEVK	0	0	0	0	0	KPCHQAEVK	Sequence	0.008	45899.78	85.00
55	HLA-A2402	SKLQAAVN	S-KLQAAVN	0	1	1	0	0	SKLQAAVN	Sequence	0.008	45909.70	85.00
36	HLA-A2402	SEKYLKIKH	SEKYLKIKH	0	0	0	0	0	SEKYLKIKH	Sequence	0.008	45916.15	85.00
51	HLA-A2402	RVDLSKLQ	R-VDLSKLQ	0	1	1	0	0	RVDLSKLQ	Sequence	0.008	45933.07	85.00
18	HLA-A2402	CHQAEVKH	CHQAEVKH-	0	8	1	0	0	CHQAEVKH	Sequence	0.008	45947.46	85.00
27	HLA-A2402	HHLLKQLK	HHLLKQLK-	0	8	1	0	0	HHLLKQLK	Sequence	0.008	46027.60	90.00
59	HLA-A2402	AAVNAAVQ	A-AVNAAVQ	0	1	1	0	0	AAVNAAVQ	Sequence	0.007	46194.22	90.00
43	HLA-A2402	KHLLLKRE	KHL-LLKRE	0	3	1	0	0	KHLLLKRE	Sequence	0.007	46293.80	90.00
50	HLA-A2402	ERVDLSKL	ERVDLS-KL	0	6	1	0	0	ERVDLSKL	Sequence	0.007	46310.82	90.00
46	HLA-A2402	LLKRERVD	LLKRER-VD	0	6	1	0	0	LLKRERVD	Sequence	0.007	46332.86	90.00
47	HLA-A2402	LKRERVDLSK	LKRERVLSK	0	0	0	6	1	LKRERVDLSK	Sequence	0.007	46349.93	90.00
35	HLA-A2402	PSEKYLKIK	PSEKYLKIK	0	0	0	0	0	PSEKYLKIK	Sequence	0.007	46398.08	90.00
50	HLA-A2402	ERVDLSKLQA	ERVDLSLQA	0	0	0	6	1	ERVDLSKLQA	Sequence	0.007	46407.12	90.00
12	HLA-A2402	IALDKPCH	I-ALDKPCH	0	1	1	0	0	IALDKPCH	Sequence	0.007	46419.18	90.00
29	HLA-A2402	LLKQLKPS	LLKQLKPS-	0	8	1	0	0	LLKQLKPS	Sequence	0.007	46484.01	90.00
48	HLA-A2402	KRERVDLS	K-RERVDLS	0	1	1	0	0	KRERVDLS	Sequence	0.006	46620.52	95.00
53	HLA-A2402	DLSKLQAA	DLSKLQAA-	0	8	1	0	0	DLSKLQAA	Sequence	0.006	46725.56	95.00
14	HLA-A2402	LDKPCHQA	L-DKPCHQA	0	1	1	0	0	LDKPCHQA	Sequence	0.006	46795.38	95.00
35	HLA-A2402	PSEKYLKIKH	PSEKYLIKH	0	0	0	6	1	PSEKYLKIKH	Sequence	0.006	46811.59	95.00
49	HLA-A2402	RERVDLSK	R-ERVDLSK	0	1	1	0	0	RERVDLSK	Sequence	0.006	46841.48	95.00
15	HLA-A2402	DKPCHQAE	DKPCHQAE-	0	8	1	0	0	DKPCHQAE	Sequence	0.006	46960.71	95.00
11	HLA-A2402	QIALDKPC	-QIALDKPC	0	0	1	0	0	QIALDKPC	Sequence	0.006	46983.59	95.00
30	HLA-A2402	LKQLKPSE	L-KQLKPSE	0	1	1	0	0	LKQLKPSE	Sequence	0.006	47065.50	95.00
21	HLA-A2402	AEVKHLHH	AEVKHLHH-	0	8	1	0	0	AEVKHLHH	Sequence	0.005	47120.53	99.00
44	HLA-A2402	HLLLKRER	HLLLKRER-	0	8	1	0	0	HLLLKRER	Sequence	0.005	47414.60	99.00
17	HLA-A2402	PCHQAEVK	PCHQAEVK-	0	8	1	0	0	PCHQAEVK	Sequence	0.005	47441.30	99.00
13	HLA-A2402	ALDKPCHQ	ALDKPCHQ-	0	8	1	0	0	ALDKPCHQ	Sequence	0.005	47481.86	99.00
36	HLA-A2402	SEKYLKIK	S-EKYLKIK	0	1	1	0	0	SEKYLKIK	Sequence	0.004	47701.74	99.00
37	HLA-A2402	EKYLKIKH	E-KYLKIKH	0	1	1	0	0	EKYLKIKH	Sequence	0.004	47984.89	99.00

Protein Sequence. Allele HLA-A2402. Number of high binders 2. Number of weak binders 2. Number of peptides 177

Link to Allele Frequencies in Worldwide Populations HLA-A2402

Rank Threshold for Strong binding peptides 0.500
Rank Threshold for Weak binding peptides 2.000

---

pos	HLA	peptide	Core	Offset	I_pos	I_len	D_pos	D_len	iCore	Identity	1-log50k(aff)	Affinity(nM)	%Rank	BindLeve
0	HLA-A2601	MVSGIISPV	MVSGIISPV	0	0	0	0	0	MVSGIISPV	Sequence	0.455	362.73	0.25	<= SB
22	HLA-A2601	EVKHLHHLL	EVKHLHHLL	0	0	0	0	0	EVKHLHHLL	Sequence	0.231	4086.10	1.30	<= WB
0	HLA-A2601	MVSGIISPVI	MVSGIISPI	0	0	0	8	1	MVSGIISPVI	Sequence	0.219	4691.74	1.50	<= WB
53	HLA-A2601	DLSKLQAAV	DLSKLQAAV	0	0	0	0	0	DLSKLQAAV	Sequence	0.110	15175.35	6.00	
6	HLA-A2601	SPVIFQIALD	SVIFQIALD	0	0	0	1	1	SPVIFQIALD	Sequence	0.108	15478.32	6.00	
57	HLA-A2601	LQAAVNAAV	LQAAVNAAV	0	0	0	0	0	LQAAVNAAV	Sequence	0.099	17060.85	7.00	
7	HLA-A2601	PVIFQIALD	PVIFQIALD	0	0	0	0	0	PVIFQIALD	Sequence	0.097	17480.71	7.50	
3	HLA-A2601	GIISPVIFQ	GIISPVIFQ	0	0	0	0	0	GIISPVIFQ	Sequence	0.095	17893.67	8.00	
39	HLA-A2601	YLKIKHLLL	YLKIKHLLL	0	0	0	0	0	YLKIKHLLL	Sequence	0.093	18185.86	8.00	
39	HLA-A2601	YLKIKHLLLK	YLIKHLLLK	0	0	0	2	1	YLKIKHLLLK	Sequence	0.093	18211.26	8.00	
0	HLA-A2601	MVSGIISP	MVSGIISP-	0	8	1	0	0	MVSGIISP	Sequence	0.092	18477.82	8.50	
25	HLA-A2601	HLHHLLKQL	HLHHLLKQL	0	0	0	0	0	HLHHLLKQL	Sequence	0.091	18620.92	8.50	
10	HLA-A2601	FQIALDKPC	FQIALDKPC	0	0	0	0	0	FQIALDKPC	Sequence	0.090	18829.19	9.00	
22	HLA-A2601	EVKHLHHLLK	EVHLHHLLK	0	0	0	2	1	EVKHLHHLLK	Sequence	0.087	19427.47	9.50	
22	HLA-A2601	EVKHLHHL	EVK-HLHHL	0	3	1	0	0	EVKHLHHL	Sequence	0.082	20564.53	11.00	
3	HLA-A2601	GIISPVIFQI	GIISVIFQI	0	0	0	4	1	GIISPVIFQI	Sequence	0.082	20623.13	11.00	
19	HLA-A2601	HQAEVKHLH	HQAEVKHLH	0	0	0	0	0	HQAEVKHLH	Sequence	0.079	21284.63	12.00	
2	HLA-A2601	SGIISPVIFQ	SIISPVIFQ	0	0	0	1	1	SGIISPVIFQ	Sequence	0.078	21553.93	12.00	
6	HLA-A2601	SPVIFQIAL	SPVIFQIAL	0	0	0	0	0	SPVIFQIAL	Sequence	0.074	22409.32	13.00	
58	HLA-A2601	QAAVNAAVQ	QAAVNAAVQ	0	0	0	0	0	QAAVNAAVQ	Sequence	0.074	22415.13	13.00	
4	HLA-A2601	IISPVIFQI	IISPVIFQI	0	0	0	0	0	IISPVIFQI	Sequence	0.072	22892.60	14.00	
1	HLA-A2601	VSGIISPV	-VSGIISPV	0	0	1	0	0	VSGIISPV	Sequence	0.068	24082.95	16.00	
50	HLA-A2601	ERVDLSKLQ	ERVDLSKLQ	0	0	0	0	0	ERVDLSKLQ	Sequence	0.067	24347.31	17.00	
20	HLA-A2601	QAEVKHLHHL	QAVKHLHHL	0	0	0	2	1	QAEVKHLHHL	Sequence	0.064	24924.91	18.00	
5	HLA-A2601	ISPVIFQIAL	ISPVIFIAL	0	0	0	6	1	ISPVIFQIAL	Sequence	0.064	25050.36	18.00	
21	HLA-A2601	AEVKHLHHLL	EVKHLHHLL	1	0	0	0	0	EVKHLHHLL	Sequence	0.063	25397.23	19.00	
5	HLA-A2601	ISPVIFQIA	ISPVIFQIA	0	0	0	0	0	ISPVIFQIA	Sequence	0.062	25461.62	19.00	
10	HLA-A2601	FQIALDKPCH	FQIALDKPH	0	0	0	8	1	FQIALDKPCH	Sequence	0.061	25783.19	20.00	
40	HLA-A2601	LKIKHLLLK	LKIKHLLLK	0	0	0	0	0	LKIKHLLLK	Sequence	0.060	26242.52	21.00	
37	HLA-A2601	EKYLKIKHL	EKYLKIKHL	0	0	0	0	0	EKYLKIKHL	Sequence	0.059	26296.51	21.00	
57	HLA-A2601	LQAAVNAAVQ	LQAAVNAAQ	0	0	0	8	1	LQAAVNAAVQ	Sequence	0.058	26582.87	22.00	
7	HLA-A2601	PVIFQIALDK	PVIFQIALK	0	0	0	8	1	PVIFQIALDK	Sequence	0.058	26597.25	22.00	
21	HLA-A2601	AEVKHLHHL	AEVKHLHHL	0	0	0	0	0	AEVKHLHHL	Sequence	0.057	26963.49	23.00	
2	HLA-A2601	SGIISPVIF	SGIISPVIF	0	0	0	0	0	SGIISPVIF	Sequence	0.057	27129.42	23.00	
15	HLA-A2601	DKPCHQAEV	DKPCHQAEV	0	0	0	0	0	DKPCHQAEV	Sequence	0.056	27265.08	24.00	
11	HLA-A2601	QIALDKPCH	QIALDKPCH	0	0	0	0	0	QIALDKPCH	Sequence	0.056	27277.78	24.00	
44	HLA-A2601	HLLLKRERV	HLLLKRERV	0	0	0	0	0	HLLLKRERV	Sequence	0.055	27561.70	25.00	
3	HLA-A2601	GIISPVIF	GIISPVI-F	0	7	1	0	0	GIISPVIF	Sequence	0.055	27612.44	25.00	
1	HLA-A2601	VSGIISPVIF	VSIISPVIF	0	0	0	2	1	VSGIISPVIF	Sequence	0.055	27634.85	25.00	
37	HLA-A2601	EKYLKIKHLL	EKYLKIHLL	0	0	0	6	1	EKYLKIKHLL	Sequence	0.055	27677.63	25.00	
1	HLA-A2601	VSGIISPVI	VSGIISPVI	0	0	0	0	0	VSGIISPVI	Sequence	0.054	27984.48	26.00	
28	HLA-A2601	HLLKQLKPS	HLLKQLKPS	0	0	0	0	0	HLLKQLKPS	Sequence	0.053	28073.94	26.00	
8	HLA-A2601	VIFQIALDK	VIFQIALDK	0	0	0	0	0	VIFQIALDK	Sequence	0.053	28098.86	26.00	

32	HLA-A2601	QLKPSEKYL	QLKPSEKYL	0	0	0	0	0	QLKPSEKYL	Sequence	0.053	28312.48	27.00
26	HLA-A2601	LHHLLKQLK	LHHLLKQLK	0	0	0	0	0	LHHLLKQLK	Sequence	0.052	28386.40	27.00
23	HLA-A2601	VKHLHHLLK	VKHLHHLLK	0	0	0	0	0	VKHLHHLLK	Sequence	0.052	28538.85	28.00
49	HLA-A2601	RERVDLSKL	RERVDLSKL	0	0	0	0	0	RERVDLSKL	Sequence	0.052	28585.52	28.00
56	HLA-A2601	KLQAAVNAAV	LQAAVNAAV	1	0	0	0	0	LQAAVNAAV	Sequence	0.051	28779.79	29.00
56	HLA-A2601	KLQAAVNAA	KLQAAVNAA	0	0	0	0	0	KLQAAVNAA	Sequence	0.051	28946.87	29.00
54	HLA-A2601	LSKLQAAVN	LSKLQAAVN	0	0	0	0	0	LSKLQAAVN	Sequence	0.048	29708.38	32.00
25	HLA-A2601	HLHHLLKQLK	HLHHLLQLK	0	0	0	6	1	HLHHLLKQLK	Sequence	0.048	29738.61	32.00
19	HLA-A2601	HQAEVKHLHH	HQAEVKHLH	0	0	0	8	1	HQAEVKHLHH	Sequence	0.047	29921.95	33.00
41	HLA-A2601	KIKHLLLKR	KIKHLLLKR	0	0	0	0	0	KIKHLLLKR	Sequence	0.047	29983.20	33.00
38	HLA-A2601	KYLKIKHLLL	YLKIKHLLL	1	0	0	0	0	YLKIKHLLL	Sequence	0.046	30233.70	34.00
46	HLA-A2601	LLKRERVDL	LLKRERVDL	0	0	0	0	0	LLKRERVDL	Sequence	0.046	30234.69	34.00
4	HLA-A2601	IISPVIFQIA	IIPVIFQIA	0	0	0	2	1	IISPVIFQIA	Sequence	0.046	30237.64	34.00
7	HLA-A2601	PVIFQIAL	PVIFQIAL-	0	8	1	0	0	PVIFQIAL	Sequence	0.046	30271.35	34.00
34	HLA-A2601	KPSEKYLKI	KPSEKYLKI	0	0	0	0	0	KPSEKYLKI	Sequence	0.045	30607.29	36.00
29	HLA-A2601	LLKQLKPSE	LLKQLKPSE	0	0	0	0	0	LLKQLKPSE	Sequence	0.045	30834.63	37.00
31	HLA-A2601	KQLKPSEKY	KQLKPSEKY	0	0	0	0	0	KQLKPSEKY	Sequence	0.045	30873.36	37.00
40	HLA-A2601	LKIKHLLLKR	LIKHLLLKR	0	0	0	1	1	LKIKHLLLKR	Sequence	0.044	31031.43	38.00
30	HLA-A2601	LKQLKPSEKY	LQLKPSEKY	0	0	0	1	1	LKQLKPSEKY	Sequence	0.044	31076.13	38.00
27	HLA-A2601	HHLLKQLKP	HHLLKQLKP	0	0	0	0	0	HHLLKQLKP	Sequence	0.044	31138.38	38.00
9	HLA-A2601	IFQIALDKPC	FQIALDKPC	1	0	0	0	0	FQIALDKPC	Sequence	0.044	31208.88	38.00
47	HLA-A2601	LKRERVDLS	LKRERVDLS	0	0	0	0	0	LKRERVDLS	Sequence	0.043	31311.36	39.00
18	HLA-A2601	CHQAEVKHL	CHQAEVKHL	0	0	0	0	0	CHQAEVKHL	Sequence	0.043	31476.45	40.00
36	HLA-A2601	SEKYLKIKH	SEKYLKIKH	0	0	0	0	0	SEKYLKIKH	Sequence	0.042	31648.22	40.00
55	HLA-A2601	SKLQAAVNAA	SLQAAVNAA	0	0	0	1	1	SKLQAAVNAA	Sequence	0.042	31761.76	41.00
33	HLA-A2601	LKPSEKYLKI	LPSEKYLKI	0	0	0	1	1	LKPSEKYLKI	Sequence	0.042	31807.49	41.00
9	HLA-A2601	IFQIALDKP	IFQIALDKP	0	0	0	0	0	IFQIALDKP	Sequence	0.042	31904.36	42.00
4	HLA-A2601	IISPVIFQ	-IISPVIFQ	0	0	1	0	0	IISPVIFQ	Sequence	0.041	31920.25	42.00
53	HLA-A2601	DLSKLQAAVN	DLSKLQAAV	0	0	0	0	0	DLSKLQAAV	Sequence	0.041	31938.20	42.00
20	HLA-A2601	QAEVKHLHH	QAEVKHLHH	0	0	0	0	0	QAEVKHLHH	Sequence	0.041	32159.79	43.00
30	HLA-A2601	LKQLKPSEK	LKQLKPSEK	0	0	0	0	0	LKQLKPSEK	Sequence	0.041	32257.71	43.00
12	HLA-A2601	IALDKPCHQ	IALDKPCHQ	0	0	0	0	0	IALDKPCHQ	Sequence	0.040	32422.16	44.00
54	HLA-A2601	LSKLQAAVNA	LSKLAAVNA	0	0	0	4	1	LSKLQAAVNA	Sequence	0.040	32512.80	45.00
57	HLA-A2601	LQAAVNAA	LQAAVNAA-	0	8	1	0	0	LQAAVNAA	Sequence	0.040	32533.92	45.00
51	HLA-A2601	RVDLSKLQA	RVDLSKLQA	0	0	0	0	0	RVDLSKLQA	Sequence	0.040	32578.64	45.00
42	HLA-A2601	IKHLLLKRE	IKHLLLKRE	0	0	0	0	0	IKHLLLKRE	Sequence	0.040	32587.81	45.00
18	HLA-A2601	CHQAEVKHLH	CQAEVKHLH	0	0	0	1	1	CHQAEVKHLH	Sequence	0.039	32712.52	46.00
10	HLA-A2601	FQIALDKP	FQIALDKP-	0	8	1	0	0	FQIALDKP	Sequence	0.039	32768.14	46.00
50	HLA-A2601	ERVDLSKLQA	ERVDLSKLA	0	0	0	8	1	ERVDLSKLQA	Sequence	0.039	32908.48	47.00
8	HLA-A2601	VIFQIALDKP	VIFQIALDK	0	0	0	0	0	VIFQIALDK	Sequence	0.039	32920.58	47.00
52	HLA-A2601	VDLSKLQAAV	DLSKLQAAV	1	0	0	0	0	DLSKLQAAV	Sequence	0.038	33024.05	47.00
8	HLA-A2601	VIFQIALD	-VIFQIALD	0	0	1	0	0	VIFQIALD	Sequence	0.038	33116.00	48.00
24	HLA-A2601	KHLHHLLKQ	KHLHHLLKQ	0	0	0	0	0	KHLHHLLKQ	Sequence	0.038	33229.42	49.00
55	HLA-A2601	SKLQAAVNA	SKLQAAVNA	0	0	0	0	0	SKLQAAVNA	Sequence	0.038	33275.49	49.00
52	HLA-A2601	VDLSKLQAA	VDLSKLQAA	0	0	0	0	0	VDLSKLQAA	Sequence	0.037	33357.30	49.00
33	HLA-A2601	LKPSEKYLK	LKPSEKYLK	0	0	0	0	0	LKPSEKYLK	Sequence	0.037	33561.13	50.00
46	HLA-A2601	LLKRERVDLS	LLRERVDLS	0	0	0	2	1	LLKRERVDLS	Sequence	0.037	33636.36	55.00
11	HLA-A2601	QIALDKPCHQ	QIALKPCHQ	0	0	0	4	1	QIALDKPCHQ	Sequence	0.036	33722.00	55.00
38	HLA-A2601	KYLKIKHLL	KYLKIKHLL	0	0	0	0	0	KYLKIKHLL	Sequence	0.036	33736.60	55.00
39	HLA-A2601	YLKIKHLL	YLKIKHLL-	0	8	1	0	0	YLKIKHLL	Sequence	0.036	33766.55	55.00
40	HLA-A2601	LKIKHLLL	-LKIKHLLL	0	0	1	0	0	LKIKHLLL	Sequence	0.036	33847.39	55.00
23	HLA-A2601	VKHLHHLLKQ	VKHLHHLLQ	0	0	0	8	1	VKHLHHLLKQ	Sequence	0.035	34074.10	55.00
27	HLA-A2601	HHLLKQLKPS	HHLLKQLPS	0	0	0	7	1	HHLLKQLKPS	Sequence	0.035	34144.60	55.00
58	HLA-A2601	QAAVNAAV	QAA-VNAAV	0	3	1	0	0	QAAVNAAV	Sequence	0.035	34356.94	55.00
47	HLA-A2601	LKRERVDLSK	LKRERVLSK	0	0	0	6	1	LKRERVDLSK	Sequence	0.035	34383.34	55.00
43	HLA-A2601	KHLLLKRER	KHLLLKRER	0	0	0	0	0	KHLLLKRER	Sequence	0.034	34557.51	60.00
24	HLA-A2601	KHLHHLLKQL	HLHHLLKQL	1	0	0	0	0	HLHHLLKQL	Sequence	0.034	34570.59	60.00
45	HLA-A2601	LLLKRERVDL	LLLKRRVDL	0	0	0	5	1	LLLKRERVDL	Sequence	0.034	34625.62	60.00
26	HLA-A2601	LHHLLKQLKP	LHHLLQLKP	0	0	0	5	1	LHHLLKQLKP	Sequence	0.034	34667.25	60.00
32	HLA-A2601	QLKPSEKY	QLKPS-EKY	0	5	1	0	0	QLKPSEKY	Sequence	0.033	35104.33	60.00
5	HLA-A2601	ISPVIFQI	ISPVIFQI-	0	8	1	0	0	ISPVIFQI	Sequence	0.033	35118.76	60.00
51	HLA-A2601	RVDLSKLQAA	RVLSKLQAA	0	0	0	2	1	RVDLSKLQAA	Sequence	0.032	35295.52	65.00
23	HLA-A2601	VKHLHHLL	-VKHLHHLL	0	0	1	0	0	VKHLHHLL	Sequence	0.032	35352.47	65.00
14	HLA-A2601	LDKPCHQAEV	LDPCHQAEV	0	0	0	2	1	LDKPCHQAEV	Sequence	0.032	35377.72	65.00
49	HLA-A2601	RERVDLSKLQ	RERVDLSLQ	0	0	0	7	1	RERVDLSKLQ	Sequence	0.032	35393.03	65.00
28	HLA-A2601	HLLKQLKPSE	HLLKQLPSE	0	0	0	6	1	HLLKQLKPSE	Sequence	0.032	35437.86	65.00
29	HLA-A2601	LLKQLKPSEK	LLQLKPSEK	0	0	0	2	1	LLKQLKPSEK	Sequence	0.031	35563.86	65.00
36	HLA-A2601	SEKYLKIKHL	SEYLKIKHL	0	0	0	2	1	SEKYLKIKHL	Sequence	0.031	35574.64	65.00
50	HLA-A2601	ERVDLSKL	ERVD-LSKL	0	4	1	0	0	ERVDLSKL	Sequence	0.031	35686.04	65.00
6	HLA-A2601	SPVIFQIA	SPVIFQIA-	0	8	1	0	0	SPVIFQIA	Sequence	0.031	35787.35	65.00
32	HLA-A2601	QLKPSEKYLK	QLKPSEKYL	0	0	0	0	0	QLKPSEKYL	Sequence	0.030	36046.17	65.00
14	HLA-A2601	LDKPCHQAE	LDKPCHQAE	0	0	0	0	0	LDKPCHQAE	Sequence	0.030	36067.24	70.00
25	HLA-A2601	HLHHLLKQ	HLH-HLLKQ	0	3	1	0	0	HLHHLLKQ	Sequence	0.030	36166.88	70.00
54	HLA-A2601	LSKLQAAV	LSKL-QAAV	0	4	1	0	0	LSKLQAAV	Sequence	0.030	36327.29	70.00
41	HLA-A2601	KIKHLLLKRE	KIHLLLKRE	0	0	0	2	1	KIKHLLLKRE	Sequence	0.030	36333.97	70.00
45	HLA-A2601	LLLKRERVD	LLLKRERVD	0	0	0	0	0	LLLKRERVD	Sequence	0.029	36345.36	70.00
42	HLA-A2601	IKHLLLKRER	IKHLLLKRR	0	0	0	8	1	IKHLLLKRER	Sequence	0.029	36411.49	70.00
13	HLA-A2601	ALDKPCHQA	ALDKPCHQA	0	0	0	0	0	ALDKPCHQA	Sequence	0.029	36464.72	70.00
15	HLA-A2601	DKPCHQAEVK	DKPCHQAVK	0	0	0	7	1	DKPCHQAEVK	Sequence	0.029	36497.09	70.00
35	HLA-A2601	PSEKYLKIK	PSEKYLKIK	0	0	0	0	0	PSEKYLKIK	Sequence	0.029	36509.33	70.00
48	HLA-A2601	KRERVDLSK	KRERVDLSK	0	0	0	0	0	KRERVDLSK	Sequence	0.029	36633.98	70.00
31	HLA-A2601	KQLKPSEKYL	QLKPSEKYL	1	0	0	0	0	QLKPSEKYL	Sequence	0.028	36899.71	75.00
42	HLA-A2601	IKHLLLKR	-IKHLLLKR	0	0	1	0	0	IKHLLLKR	Sequence	0.028	36949.24	75.00
44	HLA-A2601	HLLLKRERVD	HLLLKRERV	0	0	0	0	0	HLLLKRERV	Sequence	0.028	36959.25	75.00
43	HLA-A2601	KHLLLKRERV	HLLLKRERV	1	0	0	0	0	HLLLKRERV	Sequence	0.027	37166.56	75.00
26	HLA-A2601	LHHLLKQL	-LHHLLKQL	0	0	1	0	0	LHHLLKQL	Sequence	0.027	37382.74	75.00
53	HLA-A2601	DLSKLQAA	DLSKLQAA-	0	8	1	0	0	DLSKLQAA	Sequence	0.027	37488.05	75.00
19	HLA-A2601	HQAEVKHL	HQAEVKHL-	0	8	1	0	0	HQAEVKHL	Sequence	0.026	37549.75	75.00
41	HLA-A2601	KIKHLLLK	-KIKHLLLK	0	0	1	0	0	KIKHLLLK	Sequence	0.026	37820.48	80.00
9	HLA-A2601	IFQIALDK	-IFQIALDK	0	0	1	0	0	IFQIALDK	Sequence	0.026	37850.79	80.00

48	HLA-A2601	KRERVDLSKL	KERVDLSKL	0	0	0	1	1	KRERVDLSKL	Sequence	0.025	37960.29	80.00
59	HLA-A2601	AAVNAAVQ	AAVNA-AVQ	0	5	1	0	0	AAVNAAVQ	Sequence	0.025	38054.04	80.00
34	HLA-A2601	KPSEKYLKIK	KPSEKYLIK	0	0	0	7	1	KPSEKYLKIK	Sequence	0.025	38172.41	80.00
11	HLA-A2601	QIALDKPC	-QIALDKPC	0	0	1	0	0	QIALDKPC	Sequence	0.025	38181.50	80.00
37	HLA-A2601	EKYLKIKH	EKYLK-IKH	0	5	1	0	0	EKYLKIKH	Sequence	0.025	38191.82	80.00
24	HLA-A2601	KHLHHLLK	-KHLHHLLK	0	0	1	0	0	KHLHHLLK	Sequence	0.024	38548.84	85.00
12	HLA-A2601	IALDKPCHQA	IALDKPCHQ	0	0	0	0	0	IALDKPCHQ	Sequence	0.024	38559.29	85.00
35	HLA-A2601	PSEKYLKIKH	PSEKYLIKH	0	0	0	6	1	PSEKYLKIKH	Sequence	0.024	38657.04	85.00
28	HLA-A2601	HLLKQLKP	HLLKQLKP-	0	8	1	0	0	HLLKQLKP	Sequence	0.023	38905.44	85.00
20	HLA-A2601	QAEVKHLH	-QAEVKHLH	0	0	1	0	0	QAEVKHLH	Sequence	0.023	39040.38	85.00
27	HLA-A2601	HHLLKQLK	HHLLKQ-LK	0	6	1	0	0	HHLLKQLK	Sequence	0.023	39073.33	85.00
44	HLA-A2601	HLLLKRER	HLLLKR-ER	0	6	1	0	0	HLLLKRER	Sequence	0.023	39166.45	85.00
35	HLA-A2601	PSEKYLKI	-PSEKYLKI	0	0	1	0	0	PSEKYLKI	Sequence	0.022	39298.47	90.00
16	HLA-A2601	KPCHQAEVK	KPCHQAEVK	0	0	0	0	0	KPCHQAEVK	Sequence	0.022	39313.78	90.00
45	HLA-A2601	LLLKRERV	L-LLKRERV	0	1	1	0	0	LLLKRERV	Sequence	0.022	39390.43	90.00
47	HLA-A2601	LKRERVDL	LKRERVD-L	0	7	1	0	0	LKRERVDL	Sequence	0.022	39613.10	90.00
21	HLA-A2601	AEVKHLHH	AEV-KHLHH	0	3	1	0	0	AEVKHLHH	Sequence	0.021	39659.83	90.00
51	HLA-A2601	RVDLSKLQ	RV-DLSKLQ	0	2	1	0	0	RVDLSKLQ	Sequence	0.021	39670.57	90.00
29	HLA-A2601	LLKQLKPS	LLKQLKPS-	0	8	1	0	0	LLKQLKPS	Sequence	0.021	39670.57	90.00
33	HLA-A2601	LKPSEKYL	-LKPSEKYL	0	0	1	0	0	LKPSEKYL	Sequence	0.021	39872.82	90.00
13	HLA-A2601	ALDKPCHQAE	ALKPCHQAE	0	0	0	2	1	ALDKPCHQAE	Sequence	0.021	39906.93	90.00
49	HLA-A2601	RERVDLSK	RER-VDLSK	0	3	1	0	0	RERVDLSK	Sequence	0.021	39929.38	90.00
2	HLA-A2601	SGIISPVI	-SGIISPVI	0	0	1	0	0	SGIISPVI	Sequence	0.021	39962.66	90.00
56	HLA-A2601	KLQAAVNA	KLQAAVNA-	0	8	1	0	0	KLQAAVNA	Sequence	0.021	40034.94	90.00
17	HLA-A2601	PCHQAEVKHL	CHQAEVKHL	1	0	0	0	0	CHQAEVKHL	Sequence	0.020	40114.72	95.00
17	HLA-A2601	PCHQAEVKH	PCHQAEVKH	0	0	0	0	0	PCHQAEVKH	Sequence	0.020	40232.94	95.00
15	HLA-A2601	DKPCHQAE	DKPCHQAE-	0	8	1	0	0	DKPCHQAE	Sequence	0.020	40244.25	95.00
36	HLA-A2601	SEKYLKIK	-SEKYLKIK	0	0	1	0	0	SEKYLKIK	Sequence	0.019	40694.83	95.00
52	HLA-A2601	VDLSKLQA	-VDLSKLQA	0	0	1	0	0	VDLSKLQA	Sequence	0.019	40727.86	95.00
12	HLA-A2601	IALDKPCH	-IALDKPCH	0	0	1	0	0	IALDKPCH	Sequence	0.019	40893.46	95.00
38	HLA-A2601	KYLKIKHL	-KYLKIKHL	0	0	1	0	0	KYLKIKHL	Sequence	0.019	40903.19	95.00
30	HLA-A2601	LKQLKPSE	-LKQLKPSE	0	0	1	0	0	LKQLKPSE	Sequence	0.018	40994.45	95.00
43	HLA-A2601	KHLLLKRE	-KHLLLKRE	0	0	1	0	0	KHLLLKRE	Sequence	0.018	41013.52	95.00
16	HLA-A2601	KPCHQAEV	-KPCHQAEV	0	0	1	0	0	KPCHQAEV	Sequence	0.018	41252.53	99.00
18	HLA-A2601	CHQAEVKH	CHQAE-VKH	0	5	1	0	0	CHQAEVKH	Sequence	0.018	41262.35	99.00
34	HLA-A2601	KPSEKYLK	KPSEKY-LK	0	6	1	0	0	KPSEKYLK	Sequence	0.017	41392.01	99.00
16	HLA-A2601	KPCHQAEVKH	KPHQAEVKH	0	0	0	2	1	KPCHQAEVKH	Sequence	0.017	41426.97	99.00
46	HLA-A2601	LLKRERVD	LLKRERVD-	0	8	1	0	0	LLKRERVD	Sequence	0.017	41623.32	99.00
13	HLA-A2601	ALDKPCHQ	AL-DKPCHQ	0	2	1	0	0	ALDKPCHQ	Sequence	0.017	41634.11	99.00
55	HLA-A2601	SKLQAAVN	SKLQAAVN-	0	8	1	0	0	SKLQAAVN	Sequence	0.017	41638.62	99.00
31	HLA-A2601	KQLKPSEK	KQ-LKPSEK	0	2	1	0	0	KQLKPSEK	Sequence	0.016	42164.07	99.00
48	HLA-A2601	KRERVDLS	-KRERVDLS	0	0	1	0	0	KRERVDLS	Sequence	0.015	42526.48	99.00
14	HLA-A2601	LDKPCHQA	LDKPCHQA-	0	8	1	0	0	LDKPCHQA	Sequence	0.015	42673.49	99.00
17	HLA-A2601	PCHQAEVK	-PCHQAEVK	0	0	1	0	0	PCHQAEVK	Sequence	0.012	44066.60	99.00

Protein Sequence. Allele HLA-A2601. Number of high binders 1. Number of weak binders 2. Number of peptides 177

Link to Allele Frequencies in Worldwide Populations HLA-A2601

\# Rank Threshold for Strong binding peptides 0.500
\# Rank Threshold for Weak binding peptides 2.000

pos	HLA	peptide	Core	Offset	I_pos	I_len	D_pos	D_len	iCore	Identity	1-log50k(aff)	Affinity(nM)	%Rank	BindLeve
6	HLA-B0702	SPVIFQIAL	SPVIFQIAL	0	0	0	0	0	SPVIFQIAL	Sequence	0.626	57.24	0.30	<= SB
34	HLA-B0702	KPSEKYLKI	KPSEKYLKI	0	0	0	0	0	KPSEKYLKI	Sequence	0.389	739.83	1.40	<= WB
5	HLA-B0702	ISPVIFQIAL	IPVIFQIAL	0	0	0	1	1	ISPVIFQIAL	Sequence	0.319	1585.66	2.50	
6	HLA-B0702	SPVIFQIALD	SPVIFQIAL	0	0	0	0	0	SPVIFQIAL	Sequence	0.296	2042.05	2.50	
0	HLA-B0702	MVSGIISPV	MVSGIISPV	0	0	0	0	0	MVSGIISPV	Sequence	0.257	3109.48	3.50	
16	HLA-B0702	KPCHQAEV	KP-CHQAEV	0	2	1	0	0	KPCHQAEV	Sequence	0.248	3431.33	3.50	
49	HLA-B0702	RERVDLSKL	RERVDLSKL	0	0	0	0	0	RERVDLSKL	Sequence	0.189	6468.85	5.00	
56	HLA-B0702	KLQAAVNAAV	KLAAVNAAV	0	0	0	2	1	KLQAAVNAAV	Sequence	0.186	6680.82	5.50	
33	HLA-B0702	LKPSEKYLKI	LPSEKYLKI	0	0	0	1	1	LKPSEKYLKI	Sequence	0.166	8295.68	6.00	
6	HLA-B0702	SPVIFQIA	SPV-IFQIA	0	3	1	0	0	SPVIFQIA	Sequence	0.158	9066.79	6.50	
7	HLA-B0702	PVIFQIAL	-PVIFQIAL	0	0	1	0	0	PVIFQIAL	Sequence	0.151	9717.20	7.00	
51	HLA-B0702	RVDLSKLQAA	RVLSKLQAA	0	0	0	2	1	RVDLSKLQAA	Sequence	0.148	10075.98	7.00	
34	HLA-B0702	KPSEKYLKIK	KPSEKYLKI	0	0	0	0	0	KPSEKYLKI	Sequence	0.145	10386.36	7.50	
56	HLA-B0702	KLQAAVNAA	KLQAAVNAA	0	0	0	0	0	KLQAAVNAA	Sequence	0.143	10606.10	7.50	
25	HLA-B0702	HLHHLLKQL	HLHHLLKQL	0	0	0	0	0	HLHHLLKQL	Sequence	0.142	10814.22	7.50	
57	HLA-B0702	LQAAVNAAV	LQAAVNAAV	0	0	0	0	0	LQAAVNAAV	Sequence	0.141	10918.03	7.50	
51	HLA-B0702	RVDLSKLQA	RVDLSKLQA	0	0	0	0	0	RVDLSKLQA	Sequence	0.139	11123.00	8.00	
0	HLA-B0702	MVSGIISPVI	MVSGIISPI	0	0	0	8	1	MVSGIISPVI	Sequence	0.131	12095.79	8.50	
39	HLA-B0702	YLKIKHLLL	YLKIKHLLL	0	0	0	0	0	YLKIKHLLL	Sequence	0.126	12834.81	9.00	
21	HLA-B0702	AEVKHLHHLL	AVKHLHHLL	0	0	0	1	1	AEVKHLHHLL	Sequence	0.113	14674.64	10.00	
46	HLA-B0702	LLKRERVDL	LLKRERVDL	0	0	0	0	0	LLKRERVDL	Sequence	0.102	16503.62	12.00	
16	HLA-B0702	KPCHQAEVKH	KPHQAEVKH	0	0	0	2	1	KPCHQAEVKH	Sequence	0.098	17302.13	12.00	
45	HLA-B0702	LLLKRERVDL	LLLKRRVDL	0	0	0	5	1	LLLKRERVDL	Sequence	0.097	17416.89	13.00	
38	HLA-B0702	KYLKIKHLL	KYLKIKHLL	0	0	0	0	0	KYLKIKHLL	Sequence	0.091	18623.74	14.00	
22	HLA-B0702	EVKHLHHLL	EVKHLHHLL	0	0	0	0	0	EVKHLHHLL	Sequence	0.090	18788.49	14.00	
58	HLA-B0702	QAAVNAAVQ	QAAVNAAVQ	0	0	0	0	0	QAAVNAAVQ	Sequence	0.089	19006.65	14.00	
4	HLA-B0702	IISPVIFQI	IISPVIFQI	0	0	0	0	0	IISPVIFQI	Sequence	0.085	19893.95	15.00	
58	HLA-B0702	QAAVNAAV	QAA-VNAAV	0	3	1	0	0	QAAVNAAV	Sequence	0.081	20778.58	16.00	
21	HLA-B0702	AEVKHLHHL	AEVKHLHHL	0	0	0	0	0	AEVKHLHHL	Sequence	0.080	21010.75	16.00	
16	HLA-B0702	KPCHQAEVK	KPCHQAEVK	0	0	0	0	0	KPCHQAEVK	Sequence	0.077	21802.79	17.00	
2	HLA-B0702	SGIISPVIF	SGIISPVIF	0	0	0	0	0	SGIISPVIF	Sequence	0.076	21895.69	17.00	
44	HLA-B0702	HLLLKRERV	HLLLKRERV	0	0	0	0	0	HLLLKRERV	Sequence	0.075	22294.45	18.00	
48	HLA-B0702	KRERVDLSKL	RERVDLSKL	1	0	0	0	0	RERVDLSKL	Sequence	0.072	22856.71	19.00	
49	HLA-B0702	RERVDLSKLQ	RERVDLSKL	0	0	0	0	0	RERVDLSKL	Sequence	0.071	23099.35	19.00	
24	HLA-B0702	KHLHHLLKQL	KLHHLLKQL	0	0	0	1	1	KHLHHLLKQL	Sequence	0.068	23875.39	20.00	

19	HLA-B0702	HQAEVKHLH	HQAEVKHLH	0	0	0	0	0	HQAEVKHLH	Sequence	0.067	24127.55	21.00
1	HLA-B0702	VSGIISPVI	VSGIISPVI	0	0	0	0	0	VSGIISPVI	Sequence	0.067	24303.35	21.00
32	HLA-B0702	QLKPSEKYL	QLKPSEKYL	0	0	0	0	0	QLKPSEKYL	Sequence	0.066	24482.29	21.00
57	HLA-B0702	LQAAVNAAVQ	LQAAVNAAV	0	0	0	0	0	LQAAVNAAV	Sequence	0.065	24865.65	22.00
47	HLA-B0702	LKRERVDL	LKRE-RVDL	0	4	1	0	0	LKRERVDL	Sequence	0.065	24876.68	22.00
38	HLA-B0702	KYLKIKHLLL	KLKIKHLLL	0	0	0	1	1	KYLKIKHLLL	Sequence	0.064	25094.57	22.00
55	HLA-B0702	SKLQAAVNAA	SLQAAVNAA	0	0	0	1	1	SKLQAAVNAA	Sequence	0.062	25429.12	23.00
35	HLA-B0702	PSEKYLKI	-PSEKYLKI	0	0	1	0	0	PSEKYLKI	Sequence	0.062	25518.43	23.00
24	HLA-B0702	KHLHHLLKQ	KHLHHLLKQ	0	0	0	0	0	KHLHHLLKQ	Sequence	0.060	26063.40	24.00
23	HLA-B0702	VKHLHHLLK	VKHLHHLLK	0	0	0	0	0	VKHLHHLLK	Sequence	0.060	26149.55	24.00
28	HLA-B0702	HLLKQLKPS	HLLKQLKPS	0	0	0	0	0	HLLKQLKPS	Sequence	0.060	26178.13	24.00
40	HLA-B0702	LKIKHLLLK	LKIKHLLLK	0	0	0	0	0	LKIKHLLLK	Sequence	0.059	26364.60	25.00
20	HLA-B0702	QAEVKHLHHL	QAVKHLHHL	0	0	0	2	1	QAEVKHLHHL	Sequence	0.057	27049.12	26.00
43	HLA-B0702	KHLLLKRER	KHLLLKRER	0	0	0	0	0	KHLLLKRER	Sequence	0.056	27205.55	26.00
55	HLA-B0702	SKLQAAVNA	SKLQAAVNA	0	0	0	0	0	SKLQAAVNA	Sequence	0.056	27348.11	27.00
8	HLA-B0702	VIFQIALDK	VIFQIALDK	0	0	0	0	0	VIFQIALDK	Sequence	0.056	27410.62	27.00
34	HLA-B0702	KPSEKYLK	KPSEKYLK-	0	8	1	0	0	KPSEKYLK	Sequence	0.055	27499.13	27.00
26	HLA-B0702	LHHLLKQLK	LHHLLKQLK	0	0	0	0	0	LHHLLKQLK	Sequence	0.055	27537.25	27.00
13	HLA-B0702	ALDKPCHQA	ALDKPCHQA	0	0	0	0	0	ALDKPCHQA	Sequence	0.055	27708.80	28.00
3	HLA-B0702	GIISPVIFQ	GIISPVIFQ	0	0	0	0	0	GIISPVIFQ	Sequence	0.053	28049.05	28.00
48	HLA-B0702	KRERVDLSK	KRERVDLSK	0	0	0	0	0	KRERVDLSK	Sequence	0.053	28175.89	29.00
5	HLA-B0702	ISPVIFQIA	ISPVIFQIA	0	0	0	0	0	ISPVIFQIA	Sequence	0.052	28487.63	30.00
52	HLA-B0702	VDLSKLQAA	VDLSKLQAA	0	0	0	0	0	VDLSKLQAA	Sequence	0.051	28657.97	30.00
31	HLA-B0702	KQLKPSEKY	KQLKPSEKY	0	0	0	0	0	KQLKPSEKY	Sequence	0.051	28877.73	30.00
10	HLA-B0702	FQIALDKPC	FQIALDKPC	0	0	0	0	0	FQIALDKPC	Sequence	0.051	28928.07	31.00
41	HLA-B0702	KIKHLLLKR	KIKHLLLKR	0	0	0	0	0	KIKHLLLKR	Sequence	0.050	29086.90	31.00
11	HLA-B0702	QIALDKPCH	QIALDKPCH	0	0	0	0	0	QIALDKPCH	Sequence	0.050	29125.00	31.00
31	HLA-B0702	KQLKPSEKYL	KLKPSEKYL	0	0	0	1	1	KQLKPSEKYL	Sequence	0.049	29512.95	32.00
50	HLA-B0702	ERVDLSKLQ	ERVDLSKLQ	0	0	0	0	0	ERVDLSKLQ	Sequence	0.049	29570.49	32.00
29	HLA-B0702	LLKQLKPSE	LLKQLKPSE	0	0	0	0	0	LLKQLKPSE	Sequence	0.048	29849.19	33.00
52	HLA-B0702	VDLSKLQAAV	VLSKLQAAV	0	0	0	1	1	VDLSKLQAAV	Sequence	0.048	29870.83	33.00
47	HLA-B0702	LKRERVDLS	LKRERVDLS	0	0	0	0	0	LKRERVDLS	Sequence	0.047	30207.54	34.00
45	HLA-B0702	LLLKRERVD	LLLKRERVD	0	0	0	0	0	LLLKRERVD	Sequence	0.046	30340.21	35.00
53	HLA-B0702	DLSKLQAAV	DLSKLQAAV	0	0	0	0	0	DLSKLQAAV	Sequence	0.046	30441.48	35.00
1	HLA-B0702	VSGIISPV	-VSGIISPV	0	0	1	0	0	VSGIISPV	Sequence	0.045	30667.28	36.00
39	HLA-B0702	YLKIKHLL	YLK-IKHLL	0	3	1	0	0	YLKIKHLL	Sequence	0.044	30904.45	36.00
37	HLA-B0702	EKYLKIKHL	EKYLKIKHL	0	0	0	0	0	EKYLKIKHL	Sequence	0.043	31285.62	38.00
36	HLA-B0702	SEKYLKIKH	SEKYLKIKH	0	0	0	0	0	SEKYLKIKH	Sequence	0.043	31324.59	38.00
57	HLA-B0702	LQAAVNAA	LQAAVNAA-	0	8	1	0	0	LQAAVNAA	Sequence	0.043	31397.86	38.00
20	HLA-B0702	QAEVKHLHH	QAEVKHLHH	0	0	0	0	0	QAEVKHLHH	Sequence	0.043	31566.13	38.00
19	HLA-B0702	HQAEVKHL	HQAEVKH-L	0	7	1	0	0	HQAEVKHL	Sequence	0.042	31687.96	39.00
42	HLA-B0702	IKHLLLKRE	IKHLLLKRE	0	0	0	0	0	IKHLLLKRE	Sequence	0.042	31806.81	39.00
14	HLA-B0702	LDKPCHQAE	LDKPCHQAE	0	0	0	0	0	LDKPCHQAE	Sequence	0.042	31854.34	39.00
50	HLA-B0702	ERVDLSKLQA	RVDLSKLQA	1	0	0	0	0	RVDLSKLQA	Sequence	0.041	32231.90	41.00
43	HLA-B0702	KHLLLKRERV	KLLLKRERV	0	0	0	1	1	KHLLLKRERV	Sequence	0.040	32493.10	42.00
25	HLA-B0702	HLHHLLKQLK	HLHHLLKQL	0	0	0	0	0	HLHHLLKQL	Sequence	0.040	32518.09	42.00
12	HLA-B0702	IALDKPCHQ	IALDKPCHQ	0	0	0	0	0	IALDKPCHQ	Sequence	0.039	32746.51	43.00
37	HLA-B0702	EKYLKIKHLL	KYLKIKHLL	1	0	0	0	0	KYLKIKHLL	Sequence	0.039	32939.82	43.00
30	HLA-B0702	LKQLKPSEK	LKQLKPSEK	0	0	0	0	0	LKQLKPSEK	Sequence	0.039	32940.18	43.00
39	HLA-B0702	YLKIKHLLLK	YLKIKHLLL	0	0	0	0	0	YLKIKHLLL	Sequence	0.038	33100.96	44.00
1	HLA-B0702	VSGIISPVIF	VSGIISPVF	0	0	0	8	1	VSGIISPVIF	Sequence	0.038	33105.62	44.00
40	HLA-B0702	LKIKHLLL	LKIKHLL-L	0	7	1	0	0	LKIKHLLL	Sequence	0.038	33117.45	44.00
23	HLA-B0702	VKHLHHLL	-VKHLHHLL	0	0	1	0	0	VKHLHHLL	Sequence	0.038	33127.83	44.00
4	HLA-B0702	IISPVIFQIA	IISPVFQIA	0	0	0	5	1	IISPVIFQIA	Sequence	0.038	33190.61	44.00
54	HLA-B0702	LSKLQAAVN	LSKLQAAVN	0	0	0	0	0	LSKLQAAVN	Sequence	0.038	33269.71	45.00
0	HLA-B0702	MVSGIISP	MVSGIISP-	0	8	1	0	0	MVSGIISP	Sequence	0.038	33286.65	45.00
18	HLA-B0702	CHQAEVKHL	CHQAEVKHL	0	0	0	0	0	CHQAEVKHL	Sequence	0.037	33399.20	45.00
51	HLA-B0702	RVDLSKLQ	R-VDLSKLQ	0	1	1	0	0	RVDLSKLQ	Sequence	0.037	33517.20	46.00
3	HLA-B0702	GIISPVIFQI	GIIPVIFQI	0	0	0	3	1	GIISPVIFQI	Sequence	0.037	33542.98	46.00
47	HLA-B0702	LKRERVDLSK	LKRERVLSK	0	0	0	6	1	LKRERVDLSK	Sequence	0.037	33550.23	46.00
13	HLA-B0702	ALDKPCHQAE	ALKPCHQAE	0	0	0	2	1	ALDKPCHQAE	Sequence	0.037	33606.90	46.00
59	HLA-B0702	AAVNAAVQ	AAVNA-AVQ	0	5	1	0	0	AAVNAAVQ	Sequence	0.035	34120.21	48.00
2	HLA-B0702	SGIISPVIFQ	SIISPVIFQ	0	0	0	1	1	SGIISPVIFQ	Sequence	0.034	34442.90	49.00
19	HLA-B0702	HQAEVKHLHH	HQAEVKHLH	0	0	0	8	1	HQAEVKHLHH	Sequence	0.034	34480.95	50.00
49	HLA-B0702	RERVDLSK	RER-VDLSK	0	3	1	0	0	RERVDLSK	Sequence	0.034	34514.55	50.00
7	HLA-B0702	PVIFQIALD	PVIFQIALD	0	0	0	0	0	PVIFQIALD	Sequence	0.034	34645.50	50.00
2	HLA-B0702	SGIISPVI	SGI-ISPVI	0	3	1	0	0	SGIISPVI	Sequence	0.034	34780.71	55.00
23	HLA-B0702	VKHLHHLLKQ	VKHLHHLLQ	0	0	0	8	1	VKHLHHLLKQ	Sequence	0.033	35099.78	55.00
35	HLA-B0702	PSEKYLKIK	PSEKYLKIK	0	0	0	0	0	PSEKYLKIK	Sequence	0.032	35485.07	55.00
18	HLA-B0702	CHQAEVKHLH	HQAEVKHLH	1	0	0	0	0	HQAEVKHLH	Sequence	0.032	35524.63	55.00
10	HLA-B0702	FQIALDKPCH	FQIALDKPH	0	0	0	8	1	FQIALDKPCH	Sequence	0.031	35620.46	55.00
38	HLA-B0702	KYLKIKHL	KYLKIKH-L	0	7	1	0	0	KYLKIKHL	Sequence	0.031	35670.60	55.00
36	HLA-B0702	SEKYLKIKHL	SKYLKIKHL	0	0	0	1	1	SEKYLKIKHL	Sequence	0.031	35719.65	55.00
26	HLA-B0702	LHHLLKQL	L-HHLLKQL	0	1	1	0	0	LHHLLKQL	Sequence	0.031	35797.41	55.00
54	HLA-B0702	LSKLQAAV	LSK-LQAAV	0	3	1	0	0	LSKLQAAV	Sequence	0.030	35980.30	60.00
15	HLA-B0702	DKPCHQAEVK	KPCHQAEVK	1	0	0	0	0	KPCHQAEVK	Sequence	0.030	36151.61	60.00
22	HLA-B0702	EVKHLHHLLK	EVKHLHHLL	0	0	0	0	0	EVKHLHHLL	Sequence	0.030	36186.46	60.00
14	HLA-B0702	LDKPCHQAEV	LDKPCHQAV	0	0	0	8	1	LDKPCHQAEV	Sequence	0.030	36273.87	60.00
3	HLA-B0702	GIISPVIF	GIISP-VIF	0	5	1	0	0	GIISPVIF	Sequence	0.030	36313.91	60.00
46	HLA-B0702	LLKRERVDLS	LLKRERVDL	0	0	0	0	0	LLKRERVDL	Sequence	0.029	36593.56	60.00
41	HLA-B0702	KIKHLLLK	KIK-HLLLK	0	3	1	0	0	KIKHLLLK	Sequence	0.029	36644.29	60.00
44	HLA-B0702	HLLLKRERVD	HLLLKRRVD	0	0	0	6	1	HLLLKRERVD	Sequence	0.029	36665.70	60.00
54	HLA-B0702	LSKLQAAVNA	LSKLQAAVA	0	0	0	8	1	LSKLQAAVNA	Sequence	0.029	36678.40	60.00
33	HLA-B0702	LKPSEKYLK	LKPSEKYLK	0	0	0	0	0	LKPSEKYLK	Sequence	0.029	36727.23	60.00
40	HLA-B0702	LKIKHLLLKR	LKIKHLLLK	0	0	0	0	0	LKIKHLLLK	Sequence	0.028	36964.83	65.00
56	HLA-B0702	KLQAAVNA	KLQAAVNA-	0	8	1	0	0	KLQAAVNA	Sequence	0.028	37107.50	65.00
50	HLA-B0702	ERVDLSKL	E-RVDLSKL	0	1	1	0	0	ERVDLSKL	Sequence	0.028	37107.90	65.00
45	HLA-B0702	LLLKRERV	LLLKRERV-	0	8	1	0	0	LLLKRERV	Sequence	0.027	37376.66	65.00
24	HLA-B0702	KHLHHLLK	K-HLHHLLK	0	1	1	0	0	KHLHHLLK	Sequence	0.027	37457.64	65.00
27	HLA-B0702	HHLLKQLKP	HHLLKQLKP	0	0	0	0	0	HHLLKQLKP	Sequence	0.027	37483.19	65.00

41	HLA-B0702	KIKHLLLKRE	KKHLLLKRE	0	0	0	1	1	KIKHLLLKRE	Sequence	0.026	37544.47	65.00
43	HLA-B0702	KHLLLKRE	KHLLLKRE-	0	8	1	0	0	KHLLLKRE	Sequence	0.026	37642.90	65.00
28	HLA-B0702	HLLKQLKPSE	HLLKQLKPE	0	0	0	8	1	HLLKQLKPSE	Sequence	0.026	37708.53	65.00
42	HLA-B0702	IKHLLLKRER	KHLLLKRER	1	0	0	0	0	KHLLLKRER	Sequence	0.026	37731.81	65.00
22	HLA-B0702	EVKHLHHL	EVKHLHH-L	0	7	1	0	0	EVKHLHHL	Sequence	0.026	37788.98	70.00
12	HLA-B0702	IALDKPCHQA	IALKPCHQA	0	0	0	3	1	IALDKPCHQA	Sequence	0.026	37842.59	70.00
8	HLA-B0702	VIFQIALD	VIFQIALD-	0	8	1	0	0	VIFQIALD	Sequence	0.025	38008.37	70.00
20	HLA-B0702	QAEVKHLH	Q-AEVKHLH	0	1	1	0	0	QAEVKHLH	Sequence	0.024	38386.52	70.00
4	HLA-B0702	IISPVIFQ	-IISPVIFQ	0	0	1	0	0	IISPVIFQ	Sequence	0.024	38438.89	70.00
11	HLA-B0702	QIALDKPCHQ	QIALDKCHQ	0	0	0	6	1	QIALDKPCHQ	Sequence	0.024	38564.70	70.00
27	HLA-B0702	HHLLKQLKPS	HLLKQLKPS	0	0	0	1	1	HHLLKQLKPS	Sequence	0.024	38624.42	75.00
7	HLA-B0702	PVIFQIALDK	VIFQIALDK	1	0	0	0	0	VIFQIALDK	Sequence	0.024	38644.07	75.00
5	HLA-B0702	ISPVIFQI	-ISPVIFQI	0	0	1	0	0	ISPVIFQI	Sequence	0.024	38755.44	75.00
15	HLA-B0702	DKPCHQAEV	DKPCHQAEV	0	0	0	0	0	DKPCHQAEV	Sequence	0.023	38880.18	75.00
8	HLA-B0702	VIFQIALDKP	VIFQIALDK	0	0	0	0	0	VIFQIALDK	Sequence	0.023	38970.32	75.00
48	HLA-B0702	KRERVDLS	KRERVDLS-	0	8	1	0	0	KRERVDLS	Sequence	0.023	39098.29	75.00
10	HLA-B0702	FQIALDKP	FQIALDKP-	0	8	1	0	0	FQIALDKP	Sequence	0.023	39146.12	75.00
25	HLA-B0702	HLHHLLKQ	HLH-HLLKQ	0	3	1	0	0	HLHHLLKQ	Sequence	0.023	39148.24	75.00
26	HLA-B0702	LHHLLKQLKP	LHHLLKQLK	0	0	0	0	0	LHHLLKQLK	Sequence	0.022	39228.38	75.00
31	HLA-B0702	KQLKPSEK	KQLKP-SEK	0	5	1	0	0	KQLKPSEK	Sequence	0.022	39255.13	75.00
33	HLA-B0702	LKPSEKYL	L-KPSEKYL	0	1	1	0	0	LKPSEKYL	Sequence	0.022	39460.37	80.00
29	HLA-B0702	LLKQLKPSEK	LLKQLKSEK	0	0	0	6	1	LLKQLKPSEK	Sequence	0.022	39465.51	80.00
32	HLA-B0702	QLKPSEKYLK	QLKPSEKYL	0	0	0	0	0	QLKPSEKYL	Sequence	0.022	39533.45	80.00
27	HLA-B0702	HHLLKQLK	H-HLLKQLK	0	1	1	0	0	HHLLKQLK	Sequence	0.021	39673.57	80.00
30	HLA-B0702	LKQLKPSEKY	KQLKPSEKY	1	0	0	0	0	KQLKPSEKY	Sequence	0.021	39756.93	80.00
17	HLA-B0702	PCHQAEVK	-PCHQAEVK	0	0	1	0	0	PCHQAEVK	Sequence	0.021	39962.22	80.00
44	HLA-B0702	HLLLKRER	HLLLKR-ER	0	6	1	0	0	HLLLKRER	Sequence	0.021	39969.57	80.00
28	HLA-B0702	HLLKQLKP	HLLKQLKP-	0	8	1	0	0	HLLKQLKP	Sequence	0.020	40177.27	80.00
55	HLA-B0702	SKLQAAVN	S-KLQAAVN	0	1	1	0	0	SKLQAAVN	Sequence	0.020	40297.86	85.00
9	HLA-B0702	IFQIALDKPC	IQIALDKPC	0	0	0	1	1	IFQIALDKPC	Sequence	0.020	40358.51	85.00
17	HLA-B0702	PCHQAEVKH	PCHQAEVKH	0	0	0	0	0	PCHQAEVKH	Sequence	0.020	40385.14	85.00
11	HLA-B0702	QIALDKPC	QIA-LDKPC	0	3	1	0	0	QIALDKPC	Sequence	0.020	40419.25	85.00
9	HLA-B0702	IFQIALDK	-IFQIALDK	0	0	1	0	0	IFQIALDK	Sequence	0.020	40440.68	85.00
9	HLA-B0702	IFQIALDKP	IFQIALDKP	0	0	0	0	0	IFQIALDKP	Sequence	0.019	40508.13	85.00
36	HLA-B0702	SEKYLKIK	SE-KYLKIK	0	2	1	0	0	SEKYLKIK	Sequence	0.019	40603.34	85.00
29	HLA-B0702	LLKQLKPS	LLKQLKPS-	0	8	1	0	0	LLKQLKPS	Sequence	0.019	40631.02	85.00
37	HLA-B0702	EKYLKIKH	EKY-LKIKH	0	3	1	0	0	EKYLKIKH	Sequence	0.019	40635.87	85.00
12	HLA-B0702	IALDKPCH	-IALDKPCH	0	0	1	0	0	IALDKPCH	Sequence	0.019	40671.05	85.00
21	HLA-B0702	AEVKHLHH	AEVKHLH-H	0	7	1	0	0	AEVKHLHH	Sequence	0.019	40679.87	85.00
42	HLA-B0702	IKHLLLKR	-IKHLLLKR	0	0	1	0	0	IKHLLLKR	Sequence	0.019	40685.14	85.00
13	HLA-B0702	ALDKPCHQ	A-LDKPCHQ	0	1	1	0	0	ALDKPCHQ	Sequence	0.018	41149.54	90.00
52	HLA-B0702	VDLSKLQA	-VDLSKLQA	0	0	1	0	0	VDLSKLQA	Sequence	0.018	41309.69	90.00
35	HLA-B0702	PSEKYLKIKH	SEKYLKIKH	1	0	0	0	0	SEKYLKIKH	Sequence	0.017	41443.56	90.00
30	HLA-B0702	LKQLKPSE	LK-QLKPSE	0	2	1	0	0	LKQLKPSE	Sequence	0.017	41492.90	90.00
14	HLA-B0702	LDKPCHQA	LDKPCHQA-	0	8	1	0	0	LDKPCHQA	Sequence	0.017	41554.47	90.00
32	HLA-B0702	QLKPSEKY	QLKP-SEKY	0	4	1	0	0	QLKPSEKY	Sequence	0.017	41560.74	90.00
46	HLA-B0702	LLKRERVD	L-LKRERVD	0	1	1	0	0	LLKRERVD	Sequence	0.016	41969.25	90.00
15	HLA-B0702	DKPCHQAE	-DKPCHQAE	0	0	1	0	0	DKPCHQAE	Sequence	0.016	41995.60	95.00
53	HLA-B0702	DLSKLQAAVN	DLSKLQAAV	0	0	0	0	0	DLSKLQAAV	Sequence	0.015	42646.28	95.00
53	HLA-B0702	DLSKLQAA	-DLSKLQAA	0	0	1	0	0	DLSKLQAA	Sequence	0.014	42970.95	95.00
18	HLA-B0702	CHQAEVKH	CH-QAEVKH	0	2	1	0	0	CHQAEVKH	Sequence	0.013	43226.49	99.00
17	HLA-B0702	PCHQAEVKHL	CHQAEVKHL	1	0	0	0	0	CHQAEVKHL	Sequence	0.012	43913.35	99.00

Protein Sequence. Allele HLA-B0702. Number of high binders 1. Number of weak binders 1. Number of peptides 177

Link to Allele Frequencies in Worldwide Populations HLA-B0702

Rank Threshold for Strong binding peptides 0.500
Rank Threshold for Weak binding peptides 2.000

----

pos	HLA	peptide	Core	Offset	I_pos	I_len	D_pos	D_len	iCore	Identity	1-log50k(aff)	Affinity(nM)	%Rank	BindLeve
39	HLA-B0801	YLKIKHLLL	YLKIKHLLL	0	0	0	0	0	YLKIKHLLL	Sequence	0.866	4.25	0.01	<= SB
39	HLA-B0801	YLKIKHLL	YLKIKHL-L	0	7	1	0	0	YLKIKHLL	Sequence	0.848	5.18	0.01	<= SB
38	HLA-B0801	KYLKIKHLLL	YLKIKHLLL	1	0	0	0	0	YLKIKHLLL	Sequence	0.802	8.49	0.01	<= SB
39	HLA-B0801	YLKIKHLLLK	YLKIKHLLL	0	0	0	0	0	YLKIKHLLL	Sequence	0.703	24.97	0.07	<= SB
40	HLA-B0801	LKIKHLLL	-LKIKHLLL	0	0	1	0	0	LKIKHLLL	Sequence	0.652	43.17	0.12	<= SB
45	HLA-B0801	LLLKRERVDL	LLLKRRVDL	0	0	0	5	1	LLLKRERVDL	Sequence	0.639	49.91	0.15	<= SB
46	HLA-B0801	LLKRERVDL	LLKRERVDL	0	0	0	0	0	LLKRERVDL	Sequence	0.559	118.72	0.30	<= SB
22	HLA-B0801	EVKHLHHL	EVK-HLHHL	0	3	1	0	0	EVKHLHHL	Sequence	0.470	307.83	0.70	<= WB
44	HLA-B0801	HLLLKRERV	HLLLKRERV	0	0	0	0	0	HLLLKRERV	Sequence	0.419	536.98	0.90	<= WB
47	HLA-B0801	LKRERVDL	LKRERV-DL	0	6	1	0	0	LKRERVDL	Sequence	0.412	580.85	1.00	<= WB
45	HLA-B0801	LLLKRERV	LLLKRE-RV	0	6	1	0	0	LLLKRERV	Sequence	0.409	598.30	1.00	<= WB
37	HLA-B0801	EKYLKIKHL	EKYLKIKHL	0	0	0	0	0	EKYLKIKHL	Sequence	0.355	1074.33	1.50	<= WB
20	HLA-B0801	QAEVKHLHHL	QAEVKHHHL	0	0	0	6	1	QAEVKHLHHL	Sequence	0.328	1431.36	1.80	<= WB
22	HLA-B0801	EVKHLHHLL	EVKHLHHLL	0	0	0	0	0	EVKHLHHLL	Sequence	0.323	1515.74	1.90	<= WB
25	HLA-B0801	HLHHLLKQL	HLHHLLKQL	0	0	0	0	0	HLHHLLKQL	Sequence	0.314	1676.02	2.00	<= WB
46	HLA-B0801	LLKRERVDLS	LLKRERVDL	0	0	0	0	0	LLKRERVDL	Sequence	0.304	1873.78	2.50	
36	HLA-B0801	SEKYLKIKHL	EKYLKIKHL	1	0	0	0	0	EKYLKIKHL	Sequence	0.266	2800.86	3.00	
23	HLA-B0801	VKHLHHLL	VKHLHHL-L	0	7	1	0	0	VKHLHHLL	Sequence	0.258	3060.55	3.50	
21	HLA-B0801	AEVKHLHHLL	EVKHLHHLL	1	0	0	0	0	EVKHLHHLL	Sequence	0.253	3219.97	3.50	
6	HLA-B0801	SPVIFQIAL	SPVIFQIAL	0	0	0	0	0	SPVIFQIAL	Sequence	0.249	3390.33	3.50	
29	HLA-B0801	LLKQLKPSE	LLKQLKPSE	0	0	0	0	0	LLKQLKPSE	Sequence	0.247	3456.60	3.50	
43	HLA-B0801	KHLLLKRERV	HLLLKRERV	1	0	0	0	0	HLLLKRERV	Sequence	0.240	3720.22	4.00	
37	HLA-B0801	EKYLKIKHLL	EKYLKIKHL	0	0	0	8	1	EKYLKIKHLL	Sequence	0.238	3793.80	4.00	
54	HLA-B0801	LSKLQAAV	LSKLQ-AAV	0	5	1	0	0	LSKLQAAV	Sequence	0.231	4110.44	4.50	
34	HLA-B0801	KPSEKYLKI	KPSEKYLKI	0	0	0	0	0	KPSEKYLKI	Sequence	0.229	4175.30	4.50	
28	HLA-B0801	HLLKQLKPS	HLLKQLKPS	0	0	0	0	0	HLLKQLKPS	Sequence	0.220	4633.12	4.50	
24	HLA-B0801	KHLHHLLKQL	HLHHLLKQL	1	0	0	0	0	HLHHLLKQL	Sequence	0.219	4654.27	4.50	

32	HLA-B0801	QLKPSEKYL	QLKPSEKYL	0	0	0	0	0	QLKPSEKYL	Sequence	0.216	4846.10	5.00
33	HLA-B0801	LKPSEKYLKI	LPSEKYLKI	0	0	0	1	1	LKPSEKYLKI	Sequence	0.210	5164.94	5.00
0	HLA-B0801	MVSGIISPV	MVSGIISPV	0	0	0	0	0	MVSGIISPV	Sequence	0.186	6663.71	6.50
38	HLA-B0801	KYLKIKHL	-KYLKIKHL	0	0	1	0	0	KYLKIKHL	Sequence	0.186	6664.08	6.50
22	HLA-B0801	EVKHLHHLLK	EVKHLHHLL	0	0	0	0	0	EVKHLHHLL	Sequence	0.177	7395.02	7.00
29	HLA-B0801	LLKQLKPS	LLK-QLKPS	0	3	1	0	0	LLKQLKPS	Sequence	0.175	7515.20	7.50
45	HLA-B0801	LLLKRERVD	LLLKRERVD	0	0	0	0	0	LLLKRERVD	Sequence	0.175	7517.97	7.50
5	HLA-B0801	ISPVIFQIAL	SPVIFQIAL	1	0	0	0	0	SPVIFQIAL	Sequence	0.172	7779.54	7.50
21	HLA-B0801	AEVKHLHHL	AEVKHLHHL	0	0	0	0	0	AEVKHLHHL	Sequence	0.163	8598.16	8.50
28	HLA-B0801	HLLKQLKPSE	LLKQLKPSE	1	0	0	0	0	LLKQLKPSE	Sequence	0.159	8938.80	8.50
38	HLA-B0801	KYLKIKHLL	KYLKIKHLL	0	0	0	0	0	KYLKIKHLL	Sequence	0.154	9460.54	9.00
53	HLA-B0801	DLSKLQAA	DL-SKLQAA	0	2	1	0	0	DLSKLQAA	Sequence	0.152	9606.81	9.50
44	HLA-B0801	HLLLKRERVD	HLLLKRERV	0	0	0	0	0	HLLLKRERV	Sequence	0.152	9612.53	9.50
31	HLA-B0801	KQLKPSEKYL	QLKPSEKYL	1	0	0	0	0	QLKPSEKYL	Sequence	0.152	9688.54	9.50
53	HLA-B0801	DLSKLQAAV	DLSKLQAAV	0	0	0	0	0	DLSKLQAAV	Sequence	0.151	9740.57	9.50
0	HLA-B0801	MVSGIISPVI	MVGIISPVI	0	0	0	2	1	MVSGIISPVI	Sequence	0.147	10139.96	10.00
35	HLA-B0801	PSEKYLKI	-PSEKYLKI	0	0	1	0	0	PSEKYLKI	Sequence	0.141	10828.04	11.00
25	HLA-B0801	HLHHLLKQLK	HLHHLLKQL	0	0	0	0	0	HLHHLLKQL	Sequence	0.138	11174.75	11.00
6	HLA-B0801	SPVIFQIA	SP-VIFQIA	0	2	1	0	0	SPVIFQIA	Sequence	0.136	11465.50	12.00
57	HLA-B0801	LQAAVNAAV	LQAAVNAAV	0	0	0	0	0	LQAAVNAAV	Sequence	0.135	11579.58	12.00
26	HLA-B0801	LHHLLKQL	-LHHLLKQL	0	0	1	0	0	LHHLLKQL	Sequence	0.131	12129.47	12.00
51	HLA-B0801	RVDLSKLQAA	RVLSKLQAA	0	0	0	2	1	RVDLSKLQAA	Sequence	0.130	12261.82	13.00
27	HLA-B0801	HHLLKQLKPS	HLLKQLKPS	1	0	0	0	0	HLLKQLKPS	Sequence	0.129	12329.27	13.00
49	HLA-B0801	RERVDLSKL	RERVDLSKL	0	0	0	0	0	RERVDLSKL	Sequence	0.125	12982.86	13.00
54	HLA-B0801	LSKLQAAVNA	LSKLQAAVA	0	0	0	8	1	LSKLQAAVNA	Sequence	0.124	13086.66	14.00
48	HLA-B0801	KRERVDLSKL	KRERVDLSL	0	0	0	8	1	KRERVDLSKL	Sequence	0.124	13131.34	14.00
33	HLA-B0801	LKPSEKYL	-LKPSEKYL	0	0	1	0	0	LKPSEKYL	Sequence	0.122	13314.75	14.00
28	HLA-B0801	HLLKQLKP	HLLKQLKP-	0	8	1	0	0	HLLKQLKP	Sequence	0.118	13926.08	15.00
1	HLA-B0801	VSGIISPVI	VSGIISPVI	0	0	0	0	0	VSGIISPVI	Sequence	0.117	14083.83	15.00
46	HLA-B0801	LLKRERVD	LLK-RERVD	0	3	1	0	0	LLKRERVD	Sequence	0.115	14364.54	15.00
19	HLA-B0801	HQAEVKHL	HQAEVKH-L	0	7	1	0	0	HQAEVKHL	Sequence	0.113	14723.94	16.00
32	HLA-B0801	QLKPSEKYLK	QLKPSEKYL	0	0	0	0	0	QLKPSEKYL	Sequence	0.104	16231.96	18.00
47	HLA-B0801	LKRERVDLS	LKRERVDLS	0	0	0	0	0	LKRERVDLS	Sequence	0.102	16504.33	18.00
7	HLA-B0801	PVIFQIAL	-PVIFQIAL	0	0	1	0	0	PVIFQIAL	Sequence	0.100	16951.00	19.00
6	HLA-B0801	SPVIFQIALD	SPVIFQIAL	0	0	0	0	0	SPVIFQIAL	Sequence	0.099	17104.09	19.00
56	HLA-B0801	KLQAAVNAA	KLQAAVNAA	0	0	0	0	0	KLQAAVNAA	Sequence	0.097	17541.15	20.00
52	HLA-B0801	VDLSKLQAAV	VLSKLQAAV	0	0	0	1	1	VDLSKLQAAV	Sequence	0.096	17683.31	20.00
58	HLA-B0801	QAAVNAAV	QAAVN-AAV	0	5	1	0	0	QAAVNAAV	Sequence	0.094	18057.04	21.00
34	HLA-B0801	KPSEKYLKIK	KPSEKYLKI	0	0	0	0	0	KPSEKYLKI	Sequence	0.092	18402.20	21.00
56	HLA-B0801	KLQAAVNAAV	LQAAVNAAV	1	0	0	0	0	LQAAVNAAV	Sequence	0.091	18710.59	22.00
2	HLA-B0801	SGIISPVIF	SGIISPVIF	0	0	0	0	0	SGIISPVIF	Sequence	0.091	18778.93	22.00
54	HLA-B0801	LSKLQAAVN	LSKLQAAVN	0	0	0	0	0	LSKLQAAVN	Sequence	0.090	18832.65	22.00
29	HLA-B0801	LLKQLKPSEK	LLKQLKPSE	0	0	0	0	0	LLKQLKPSE	Sequence	0.090	18984.04	22.00
30	HLA-B0801	LKQLKPSE	LKQLKP-SE	0	6	1	0	0	LKQLKPSE	Sequence	0.088	19370.59	23.00
18	HLA-B0801	CHQAEVKHL	CHQAEVKHL	0	0	0	0	0	CHQAEVKHL	Sequence	0.087	19557.39	23.00
5	HLA-B0801	ISPVIFQIA	ISPVIFQIA	0	0	0	0	0	ISPVIFQIA	Sequence	0.086	19793.47	24.00
14	HLA-B0801	LDKPCHQAE	LDKPCHQAE	0	0	0	0	0	LDKPCHQAE	Sequence	0.085	19843.44	24.00
10	HLA-B0801	FQIALDKPC	FQIALDKPC	0	0	0	0	0	FQIALDKPC	Sequence	0.085	19980.68	24.00
12	HLA-B0801	IALDKPCHQA	IALDKCHQA	0	0	0	5	1	IALDKPCHQA	Sequence	0.083	20293.98	25.00
57	HLA-B0801	LQAAVNAAVQ	LQAAVNAAV	0	0	0	0	0	LQAAVNAAV	Sequence	0.083	20465.76	25.00
4	HLA-B0801	IISPVIFQIA	IISVIFQIA	0	0	0	3	1	IISPVIFQIA	Sequence	0.082	20487.24	25.00
20	HLA-B0801	QAEVKHLHH	QAEVKHLHH	0	0	0	0	0	QAEVKHLHH	Sequence	0.080	20935.86	26.00
40	HLA-B0801	LKIKHLLLK	LKIKHLLLK	0	0	0	0	0	LKIKHLLLK	Sequence	0.079	21272.44	26.00
58	HLA-B0801	QAAVNAAVQ	QAAVNAAVQ	0	0	0	0	0	QAAVNAAVQ	Sequence	0.078	21573.52	27.00
47	HLA-B0801	LKRERVDLSK	LKRERVLSK	0	0	0	6	1	LKRERVDLSK	Sequence	0.078	21576.33	27.00
52	HLA-B0801	VDLSKLQAA	VDLSKLQAA	0	0	0	0	0	VDLSKLQAA	Sequence	0.077	21640.85	27.00
13	HLA-B0801	ALDKPCHQAE	ALKPCHQAE	0	0	0	2	1	ALDKPCHQAE	Sequence	0.077	21809.16	27.00
4	HLA-B0801	IISPVIFQI	IISPVIFQI	0	0	0	0	0	IISPVIFQI	Sequence	0.076	21864.92	28.00
50	HLA-B0801	ERVDLSKL	E-RVDLSKL	0	1	1	0	0	ERVDLSKL	Sequence	0.076	21920.59	28.00
1	HLA-B0801	VSGIISPVIF	VSGIISPVF	0	0	0	8	1	VSGIISPVIF	Sequence	0.076	21953.58	28.00
44	HLA-B0801	HLLLKRER	HLLLKRER-	0	8	1	0	0	HLLLKRER	Sequence	0.076	22055.71	28.00
14	HLA-B0801	LDKPCHQAEV	LDKPCHQAV	0	0	0	8	1	LDKPCHQAEV	Sequence	0.075	22302.40	28.00
1	HLA-B0801	VSGIISPV	VSGII-SPV	0	5	1	0	0	VSGIISPV	Sequence	0.072	23035.71	30.00
53	HLA-B0801	DLSKLQAAVN	DLSKLQAAV	0	0	0	0	0	DLSKLQAAV	Sequence	0.069	23651.96	31.00
2	HLA-B0801	SGIISPVI	S-GIISPVI	0	1	1	0	0	SGIISPVI	Sequence	0.067	24138.78	33.00
19	HLA-B0801	HQAEVKHLH	HQAEVKHLH	0	0	0	0	0	HQAEVKHLH	Sequence	0.066	24540.91	34.00
30	HLA-B0801	LKQLKPSEKY	LKQLKPSKY	0	0	0	7	1	LKQLKPSEKY	Sequence	0.065	24796.87	34.00
49	HLA-B0801	RERVDLSKLQ	RERVDLSKL	0	0	0	0	0	RERVDLSKL	Sequence	0.064	24891.49	34.00
30	HLA-B0801	LKQLKPSEK	LKQLKPSEK	0	0	0	0	0	LKQLKPSEK	Sequence	0.064	25042.77	35.00
12	HLA-B0801	IALDKPCHQ	IALDKPCHQ	0	0	0	0	0	IALDKPCHQ	Sequence	0.063	25243.54	35.00
55	HLA-B0801	SKLQAAVNAA	SLQAAVNAA	0	0	0	1	1	SKLQAAVNAA	Sequence	0.062	25576.75	36.00
19	HLA-B0801	HQAEVKHLHH	HQAVKHLHH	0	0	0	3	1	HQAEVKHLHH	Sequence	0.062	25661.30	36.00
23	HLA-B0801	VKHLHHLLK	VKHLHHLLK	0	0	0	0	0	VKHLHHLLK	Sequence	0.060	26015.22	37.00
57	HLA-B0801	LQAAVNAA	LQAAV-NAA	0	5	1	0	0	LQAAVNAA	Sequence	0.059	26446.60	39.00
16	HLA-B0801	KPCHQAEV	KP-CHQAEV	0	2	1	0	0	KPCHQAEV	Sequence	0.059	26473.22	39.00
13	HLA-B0801	ALDKPCHQA	ALDKPCHQA	0	0	0	0	0	ALDKPCHQA	Sequence	0.058	26811.06	40.00
17	HLA-B0801	PCHQAEVKHL	CHQAEVKHL	1	0	0	0	0	CHQAEVKHL	Sequence	0.057	26879.32	40.00
9	HLA-B0801	IFQIALDKPC	FQIALDKPC	1	0	0	0	0	FQIALDKPC	Sequence	0.057	27062.59	40.00
42	HLA-B0801	IKHLLLKRE	IKHLLLKRE	0	0	0	0	0	IKHLLLKRE	Sequence	0.055	27679.13	42.00
20	HLA-B0801	QAEVKHLH	QAEVKHLH-	0	8	1	0	0	QAEVKHLH	Sequence	0.053	28301.77	44.00
0	HLA-B0801	MVSGIISP	MVSGIISP-	0	8	1	0	0	MVSGIISP	Sequence	0.051	28675.98	45.00
55	HLA-B0801	SKLQAAVNA	SKLQAAVNA	0	0	0	0	0	SKLQAAVNA	Sequence	0.050	28985.72	46.00
18	HLA-B0801	CHQAEVKHLH	CHQAEVKHL	0	0	0	0	0	CHQAEVKHL	Sequence	0.050	29116.18	46.00
25	HLA-B0801	HLHHLLKQ	HLH-HLLKQ	0	3	1	0	0	HLHHLLKQ	Sequence	0.050	29208.63	47.00
50	HLA-B0801	ERVDLSKLQ	ERVDLSKLQ	0	0	0	0	0	ERVDLSKLQ	Sequence	0.050	29218.73	47.00
15	HLA-B0801	DKPCHQAEV	DKPCHQAEV	0	0	0	0	0	DKPCHQAEV	Sequence	0.049	29323.25	47.00
31	HLA-B0801	KQLKPSEKY	KQLKPSEKY	0	0	0	0	0	KQLKPSEKY	Sequence	0.049	29382.33	47.00
40	HLA-B0801	LKIKHLLLKR	LKIKHLLLR	0	0	0	8	1	LKIKHLLLKR	Sequence	0.049	29480.73	48.00
51	HLA-B0801	RVDLSKLQA	RVDLSKLQA	0	0	0	0	0	RVDLSKLQA	Sequence	0.048	29614.34	48.00
26	HLA-B0801	LHHLLKQLK	LHHLLKQLK	0	0	0	0	0	LHHLLKQLK	Sequence	0.048	29829.18	49.00

27	HLA-B0801	HHLLKQLKP	HHLLKQLKP	0	0	0	0	0	HHLLKQLKP	Sequence	0.047	30097.93	50.00
14	HLA-B0801	LDKPCHQA	LDKP-CHQA	0	4	1	0	0	LDKPCHQA	Sequence	0.046	30528.55	55.00
3	HLA-B0801	GIISPVIFQI	IISPVIFQI	1	0	0	0	0	IISPVIFQI	Sequence	0.045	30882.71	55.00
10	HLA-B0801	FQIALDKPCH	FQIALDKPH	0	0	0	8	1	FQIALDKPCH	Sequence	0.044	31006.92	55.00
23	HLA-B0801	VKHLHHLLKQ	VKHLHHLLQ	0	0	0	8	1	VKHLHHLLKQ	Sequence	0.043	31418.26	55.00
16	HLA-B0801	KPCHQAEVK	KPCHQAEVK	0	0	0	0	0	KPCHQAEVK	Sequence	0.042	31636.57	55.00
11	HLA-B0801	QIALDKPCHQ	QIALKPCHQ	0	0	0	4	1	QIALDKPCHQ	Sequence	0.042	31747.32	60.00
41	HLA-B0801	KIKHLLLKRE	KIKLLLKRE	0	0	0	3	1	KIKHLLLKRE	Sequence	0.042	31781.35	60.00
48	HLA-B0801	KRERVDLSK	KRERVDLSK	0	0	0	0	0	KRERVDLSK	Sequence	0.042	31844.01	60.00
41	HLA-B0801	KIKHLLLKR	KIKHLLLKR	0	0	0	0	0	KIKHLLLKR	Sequence	0.042	31907.12	60.00
50	HLA-B0801	ERVDLSKLQA	ERVDLSKLA	0	0	0	8	1	ERVDLSKLQA	Sequence	0.041	32075.34	60.00
11	HLA-B0801	QIALDKPCH	QIALDKPCH	0	0	0	0	0	QIALDKPCH	Sequence	0.041	32241.65	60.00
15	HLA-B0801	DKPCHQAE	D-KPCHQAE	0	1	1	0	0	DKPCHQAE	Sequence	0.041	32259.81	60.00
3	HLA-B0801	GIISPVIF	GI-ISPVIF	0	2	1	0	0	GIISPVIF	Sequence	0.040	32361.89	60.00
35	HLA-B0801	PSEKYLKIK	PSEKYLKIK	0	0	0	0	0	PSEKYLKIK	Sequence	0.039	32662.65	60.00
37	HLA-B0801	EKYLKIKH	EKYLKIKH-	0	8	1	0	0	EKYLKIKH	Sequence	0.039	32801.84	60.00
34	HLA-B0801	KPSEKYLK	KPSEKYLK-	0	8	1	0	0	KPSEKYLK	Sequence	0.039	32827.05	60.00
33	HLA-B0801	LKPSEKYLK	LKPSEKYLK	0	0	0	0	0	LKPSEKYLK	Sequence	0.038	33050.13	65.00
5	HLA-B0801	ISPVIFQI	ISPVIFQ-I	0	7	1	0	0	ISPVIFQI	Sequence	0.038	33247.41	65.00
8	HLA-B0801	VIFQIALDK	VIFQIALDK	0	0	0	0	0	VIFQIALDK	Sequence	0.038	33258.93	65.00
11	HLA-B0801	QIALDKPC	QIA-LDKPC	0	3	1	0	0	QIALDKPC	Sequence	0.037	33510.68	65.00
32	HLA-B0801	QLKPSEKY	QLKPSEK-Y	0	7	1	0	0	QLKPSEKY	Sequence	0.037	33559.31	65.00
26	HLA-B0801	LHHLLKQLKP	LHHLLKQLP	0	0	0	8	1	LHHLLKQLKP	Sequence	0.036	33792.13	65.00
10	HLA-B0801	FQIALDKP	FQIALDKP-	0	8	1	0	0	FQIALDKP	Sequence	0.036	34001.92	65.00
48	HLA-B0801	KRERVDLS	-KRERVDLS	0	0	1	0	0	KRERVDLS	Sequence	0.035	34108.77	65.00
2	HLA-B0801	SGIISPVIFQ	SGIISPVIF	0	0	0	0	0	SGIISPVIF	Sequence	0.035	34267.10	70.00
12	HLA-B0801	IALDKPCH	IALDKPCH-	0	8	1	0	0	IALDKPCH	Sequence	0.034	34488.02	70.00
36	HLA-B0801	SEKYLKIKH	SEKYLKIKH	0	0	0	0	0	SEKYLKIKH	Sequence	0.034	34617.38	70.00
41	HLA-B0801	KIKHLLLK	KIK-HLLLK	0	3	1	0	0	KIKHLLLK	Sequence	0.034	34634.24	70.00
59	HLA-B0801	AAVNAAVQ	-AAVNAAVQ	0	0	1	0	0	AAVNAAVQ	Sequence	0.033	35048.55	70.00
36	HLA-B0801	SEKYLKIK	SEK-YLKIK	0	3	1	0	0	SEKYLKIK	Sequence	0.031	35585.03	75.00
7	HLA-B0801	PVIFQIALD	PVIFQIALD	0	0	0	0	0	PVIFQIALD	Sequence	0.031	35602.36	75.00
55	HLA-B0801	SKLQAAVN	S-KLQAAVN	0	1	1	0	0	SKLQAAVN	Sequence	0.031	35642.06	75.00
21	HLA-B0801	AEVKHLHH	-AEVKHLHH	0	0	1	0	0	AEVKHLHH	Sequence	0.031	35904.09	75.00
24	HLA-B0801	KHLHHLLKQ	KHLHHLLKQ	0	0	0	0	0	KHLHHLLKQ	Sequence	0.031	35937.52	75.00
8	HLA-B0801	VIFQIALDKP	VIFQIALKP	0	0	0	7	1	VIFQIALDKP	Sequence	0.030	36107.83	75.00
15	HLA-B0801	DKPCHQAEVK	DKPCHQAEV	0	0	0	0	0	DKPCHQAEV	Sequence	0.030	36239.36	75.00
24	HLA-B0801	KHLHHLLK	-KHLHHLLK	0	0	1	0	0	KHLHHLLK	Sequence	0.029	36445.79	80.00
56	HLA-B0801	KLQAAVNA	KLQAAVN-A	0	7	1	0	0	KLQAAVNA	Sequence	0.028	37095.05	80.00
13	HLA-B0801	ALDKPCHQ	-ALDKPCHQ	0	0	1	0	0	ALDKPCHQ	Sequence	0.027	37286.59	80.00
16	HLA-B0801	KPCHQAEVKH	KPCHQAEVH	0	0	0	8	1	KPCHQAEVKH	Sequence	0.026	37733.85	85.00
3	HLA-B0801	GIISPVIFQ	GIISPVIFQ	0	0	0	0	0	GIISPVIFQ	Sequence	0.026	37911.45	85.00
35	HLA-B0801	PSEKYLKIKH	PSEKYLKIH	0	0	0	8	1	PSEKYLKIKH	Sequence	0.026	37941.41	85.00
9	HLA-B0801	IFQIALDKP	IFQIALDKP	0	0	0	0	0	IFQIALDKP	Sequence	0.025	38000.97	85.00
4	HLA-B0801	IISPVIFQ	IISPVIFQ-	0	8	1	0	0	IISPVIFQ	Sequence	0.025	38207.54	85.00
8	HLA-B0801	VIFQIALD	VIFQIALD-	0	8	1	0	0	VIFQIALD	Sequence	0.025	38224.89	85.00
52	HLA-B0801	VDLSKLQA	VDLSK-LQA	0	5	1	0	0	VDLSKLQA	Sequence	0.025	38277.04	85.00
42	HLA-B0801	IKHLLLKRER	IKHLLLKRE	0	0	0	0	0	IKHLLLKRE	Sequence	0.024	38537.61	85.00
51	HLA-B0801	RVDLSKLQ	-RVDLSKLQ	0	0	1	0	0	RVDLSKLQ	Sequence	0.024	38733.22	90.00
7	HLA-B0801	PVIFQIALDK	VIFQIALDK	1	0	0	0	0	VIFQIALDK	Sequence	0.023	38786.49	90.00
27	HLA-B0801	HHLLKQLK	HHLLKQLK-	0	8	1	0	0	HHLLKQLK	Sequence	0.023	38848.22	90.00
42	HLA-B0801	IKHLLLKR	IKHLLLKR-	0	8	1	0	0	IKHLLLKR	Sequence	0.023	38898.69	90.00
31	HLA-B0801	KQLKPSEK	-KQLKPSEK	0	0	1	0	0	KQLKPSEK	Sequence	0.023	39008.29	90.00
43	HLA-B0801	KHLLLKRE	-KHLLLKRE	0	0	1	0	0	KHLLLKRE	Sequence	0.022	39219.05	90.00
49	HLA-B0801	RERVDLSK	RERVDLSK-	0	8	1	0	0	RERVDLSK	Sequence	0.021	39804.29	90.00
43	HLA-B0801	KHLLLKRER	KHLLLKRER	0	0	0	0	0	KHLLLKRER	Sequence	0.021	39929.38	95.00
17	HLA-B0801	PCHQAEVKH	PCHQAEVKH	0	0	0	0	0	PCHQAEVKH	Sequence	0.020	40334.05	95.00
9	HLA-B0801	IFQIALDK	-IFQIALDK	0	0	1	0	0	IFQIALDK	Sequence	0.017	41407.68	99.00
17	HLA-B0801	PCHQAEVK	-PCHQAEVK	0	0	1	0	0	PCHQAEVK	Sequence	0.016	41872.66	99.00
18	HLA-B0801	CHQAEVKH	C-HQAEVKH	0	1	1	0	0	CHQAEVKH	Sequence	0.015	42655.50	99.00

Protein Sequence. Allele HLA-B0801. Number of high binders 7. Number of weak binders 8. Number of peptides 177

Link to Allele Frequencies in Worldwide Populations HLA-B0801

Rank Threshold for Strong binding peptides 0.500
Rank Threshold for Weak binding peptides 2.000

pos	HLA	peptide	Core	Offset	I_pos	I_len	D_pos	D_len	iCore	Identity	1-log50k(aff)	Affinity(nM)	%Rank	BindLeve
48	HLA-B2705	KRERVDLSKL	KRRVDLSKL	0	0	0	2	1	KRERVDLSKL	Sequence	0.413	575.38	1.50	<= WB
49	HLA-B2705	RERVDLSKLQ	RRVDLSKLQ	0	0	0	1	1	RERVDLSKLQ	Sequence	0.347	1165.61	2.50	
48	HLA-B2705	KRERVDLSK	KRERVDLSK	0	0	0	0	0	KRERVDLSK	Sequence	0.346	1181.82	2.50	
40	HLA-B2705	LKIKHLLLK	LKIKHLLLK	0	0	0	0	0	LKIKHLLLK	Sequence	0.311	1737.47	3.00	
23	HLA-B2705	VKHLHHLLK	VKHLHHLLK	0	0	0	0	0	VKHLHHLLK	Sequence	0.275	2564.17	4.00	
39	HLA-B2705	YLKIKHLLLK	YKIKHLLLK	0	0	0	1	1	YLKIKHLLLK	Sequence	0.258	3054.00	4.50	
40	HLA-B2705	LKIKHLLLKR	LKIHLLLKR	0	0	0	3	1	LKIKHLLLKR	Sequence	0.255	3171.18	4.50	
47	HLA-B2705	LKRERVDLSK	LRERVDLSK	0	0	0	1	1	LKRERVDLSK	Sequence	0.250	3339.00	4.50	
31	HLA-B2705	KQLKPSEKY	KQLKPSEKY	0	0	0	0	0	KQLKPSEKY	Sequence	0.228	4247.39	5.00	
19	HLA-B2705	HQAEVKHLH	HQAEVKHLH	0	0	0	0	0	HQAEVKHLH	Sequence	0.213	4996.35	5.50	
42	HLA-B2705	IKHLLLKRER	IKHLLLKRR	0	0	0	8	1	IKHLLLKRER	Sequence	0.211	5111.35	6.00	
36	HLA-B2705	SEKYLKIKHL	SKYLKIKHL	0	0	0	1	1	SEKYLKIKHL	Sequence	0.204	5499.09	6.00	
37	HLA-B2705	EKYLKIKHL	EKYLKIKHL	0	0	0	0	0	EKYLKIKHL	Sequence	0.199	5780.40	6.00	
57	HLA-B2705	LQAAVNAAV	LQAAVNAAV	0	0	0	0	0	LQAAVNAAV	Sequence	0.196	6024.12	6.50	
50	HLA-B2705	ERVDLSKL	ERVDL-SKL	0	5	1	0	0	ERVDLSKL	Sequence	0.195	6064.28	6.50	
38	HLA-B2705	KYLKIKHLL	KYLKIKHLL	0	0	0	0	0	KYLKIKHLL	Sequence	0.190	6421.22	6.50	
10	HLA-B2705	FQIALDKPC	FQIALDKPC	0	0	0	0	0	FQIALDKPC	Sequence	0.173	7669.97	7.50	
25	HLA-B2705	HLHHLLKQL	HLHHLLKQL	0	0	0	0	0	HLHHLLKQL	Sequence	0.169	8024.56	7.50	
31	HLA-B2705	KQLKPSEKYL	KQLKPSEKL	0	0	0	8	1	KQLKPSEKYL	Sequence	0.166	8270.32	8.00	

23	HLA-B2705	VKHLHHLLKQ	VKHLHHLLK	0	0	0	0	0	VKHLHHLLK	Sequence	0.162	8709.39	8.00
50	HLA-B2705	ERVDLSKLQ	ERVDLSKLQ	0	0	0	0	0	ERVDLSKLQ	Sequence	0.161	8768.01	8.50
26	HLA-B2705	LHHLLKQLK	LHHLLKQLK	0	0	0	0	0	LHHLLKQLK	Sequence	0.160	8893.94	8.50
25	HLA-B2705	HLHHLLKQLK	HLHHLLKLK	0	0	0	7	1	HLHHLLKQLK	Sequence	0.159	8930.68	8.50
42	HLA-B2705	IKHLLLKR	IKHLLL-KR	0	6	1	0	0	IKHLLLKR	Sequence	0.155	9358.63	8.50
37	HLA-B2705	EKYLKIKHLL	EKYLKIHLL	0	0	0	6	1	EKYLKIKHLL	Sequence	0.154	9399.93	8.50
50	HLA-B2705	ERVDLSKLQA	ERVDLSKLA	0	0	0	8	1	ERVDLSKLQA	Sequence	0.148	10113.34	9.00
10	HLA-B2705	FQIALDKPCH	FQIALDKPH	0	0	0	8	1	FQIALDKPCH	Sequence	0.147	10206.12	9.50
56	HLA-B2705	KLQAAVNAAV	KQAAVNAAV	0	0	0	1	1	KLQAAVNAAV	Sequence	0.144	10472.80	9.50
30	HLA-B2705	LKQLKPSEK	LKQLKPSEK	0	0	0	0	0	LKQLKPSEK	Sequence	0.144	10546.26	9.50
48	HLA-B2705	KRERVDLS	KRERVDLS-	0	8	1	0	0	KRERVDLS	Sequence	0.143	10603.69	9.50
41	HLA-B2705	KIKHLLLKR	KIKHLLLKR	0	0	0	0	0	KIKHLLLKR	Sequence	0.132	11940.15	11.00
24	HLA-B2705	KHLHHLLKQL	KLHHLLKQL	0	0	0	1	1	KHLHHLLKQL	Sequence	0.129	12345.42	11.00
49	HLA-B2705	RERVDLSK	-RERVDLSK	0	0	1	0	0	RERVDLSK	Sequence	0.129	12348.63	11.00
38	HLA-B2705	KYLKIKHLLL	KYLKIKLLL	0	0	0	6	1	KYLKIKHLLL	Sequence	0.126	12736.58	11.00
22	HLA-B2705	EVKHLHHLLK	EKHLHHLLK	0	0	0	1	1	EVKHLHHLLK	Sequence	0.125	12963.35	12.00
18	HLA-B2705	CHQAEVKHLH	CQAEVKHLH	0	0	0	1	1	CHQAEVKHLH	Sequence	0.124	13096.58	12.00
49	HLA-B2705	RERVDLSKL	RERVDLSKL	0	0	0	0	0	RERVDLSKL	Sequence	0.122	13356.88	12.00
19	HLA-B2705	HQAEVKHLHH	HQAEVKHLH	0	0	0	8	1	HQAEVKHLHH	Sequence	0.122	13419.03	12.00
39	HLA-B2705	YLKIKHLLL	YLKIKHLLL	0	0	0	0	0	YLKIKHLLL	Sequence	0.117	14136.65	13.00
19	HLA-B2705	HQAEVKHL	HQAEVK-HL	0	6	1	0	0	HQAEVKHL	Sequence	0.114	14556.04	13.00
43	HLA-B2705	KHLLLKRER	KHLLLKRER	0	0	0	0	0	KHLLLKRER	Sequence	0.112	14816.47	13.00
55	HLA-B2705	SKLQAAVNA	SKLQAAVNA	0	0	0	0	0	SKLQAAVNA	Sequence	0.110	15142.06	14.00
30	HLA-B2705	LKQLKPSEKY	LQLKPSEKY	0	0	0	1	1	LKQLKPSEKY	Sequence	0.109	15305.45	14.00
0	HLA-B2705	MVSGIISPV	MVSGIISPV	0	0	0	0	0	MVSGIISPV	Sequence	0.102	16575.38	15.00
23	HLA-B2705	VKHLHHLL	VKHLH-HLL	0	5	1	0	0	VKHLHHLL	Sequence	0.101	16688.93	15.00
24	HLA-B2705	KHLHHLLK	-KHLHHLLK	0	0	1	0	0	KHLHHLLK	Sequence	0.101	16738.12	15.00
57	HLA-B2705	LQAAVNAAVQ	LQAAVNAAV	0	0	0	0	0	LQAAVNAAV	Sequence	0.101	16783.10	15.00
42	HLA-B2705	IKHLLLKRE	IKHLLLKRE	0	0	0	0	0	IKHLLLKRE	Sequence	0.098	17348.25	16.00
33	HLA-B2705	LKPSEKYLKI	LKSEKYLKI	0	0	0	2	1	LKPSEKYLKI	Sequence	0.098	17391.10	16.00
40	HLA-B2705	LKIKHLLL	LKIKHL-LL	0	6	1	0	0	LKIKHLLL	Sequence	0.097	17469.74	16.00
44	HLA-B2705	HLLLKRERV	HLLLKRERV	0	0	0	0	0	HLLLKRERV	Sequence	0.096	17772.12	16.00
33	HLA-B2705	LKPSEKYLK	LKPSEKYLK	0	0	0	0	0	LKPSEKYLK	Sequence	0.093	18307.87	17.00
31	HLA-B2705	KQLKPSEK	KQLK-PSEK	0	4	1	0	0	KQLKPSEK	Sequence	0.093	18364.80	17.00
41	HLA-B2705	KIKHLLLK	-KIKHLLLK	0	0	1	0	0	KIKHLLLK	Sequence	0.092	18407.57	17.00
24	HLA-B2705	KHLHHLLKQ	KHLHHLLKQ	0	0	0	0	0	KHLHHLLKQ	Sequence	0.090	18796.42	17.00
56	HLA-B2705	KLQAAVNAA	KLQAAVNAA	0	0	0	0	0	KLQAAVNAA	Sequence	0.090	18800.89	17.00
38	HLA-B2705	KYLKIKHL	-KYLKIKHL	0	0	1	0	0	KYLKIKHL	Sequence	0.090	18923.75	18.00
18	HLA-B2705	CHQAEVKHL	CHQAEVKHL	0	0	0	0	0	CHQAEVKHL	Sequence	0.087	19451.66	18.00
41	HLA-B2705	KIKHLLLKRE	KKHLLLKRE	0	0	0	1	1	KIKHLLLKRE	Sequence	0.086	19731.89	19.00
51	HLA-B2705	RVDLSKLQA	RVDLSKLQA	0	0	0	0	0	RVDLSKLQA	Sequence	0.085	19834.20	19.00
10	HLA-B2705	FQIALDKP	FQIALDKP-	0	8	1	0	0	FQIALDKP	Sequence	0.085	19914.41	19.00
22	HLA-B2705	EVKHLHHLL	EVKHLHHLL	0	0	0	0	0	EVKHLHHLL	Sequence	0.083	20340.58	19.00
34	HLA-B2705	KPSEKYLKI	KPSEKYLKI	0	0	0	0	0	KPSEKYLKI	Sequence	0.081	20865.08	20.00
28	HLA-B2705	HLLKQLKPS	HLLKQLKPS	0	0	0	0	0	HLLKQLKPS	Sequence	0.080	20941.98	20.00
47	HLA-B2705	LKRERVDLS	LKRERVDLS	0	0	0	0	0	LKRERVDLS	Sequence	0.080	21016.43	20.00
51	HLA-B2705	RVDLSKLQ	-RVDLSKLQ	0	0	1	0	0	RVDLSKLQ	Sequence	0.078	21563.03	21.00
55	HLA-B2705	SKLQAAVNAA	SKLQAAVAA	0	0	0	7	1	SKLQAAVNAA	Sequence	0.077	21740.37	21.00
8	HLA-B2705	VIFQIALDK	VIFQIALDK	0	0	0	0	0	VIFQIALDK	Sequence	0.077	21775.91	21.00
29	HLA-B2705	LLKQLKPSEK	LKQLKPSEK	0	0	0	1	1	LLKQLKPSEK	Sequence	0.074	22467.82	23.00
26	HLA-B2705	LHHLLKQLKP	LHHLLKQLK	0	0	0	0	0	LHHLLKQLK	Sequence	0.074	22511.38	23.00
46	HLA-B2705	LLKRERVDL	LLKRERVDL	0	0	0	0	0	LLKRERVDL	Sequence	0.073	22635.21	23.00
51	HLA-B2705	RVDLSKLQAA	RVLSKLQAA	0	0	0	2	1	RVDLSKLQAA	Sequence	0.072	22959.56	23.00
21	HLA-B2705	AEVKHLHHL	AEVKHLHHL	0	0	0	0	0	AEVKHLHHL	Sequence	0.071	23165.93	24.00
0	HLA-B2705	MVSGIISPVI	MVSGIISPI	0	0	0	8	1	MVSGIISPVI	Sequence	0.070	23362.52	24.00
43	HLA-B2705	KHLLLKRERV	KHLLLRERV	0	0	0	5	1	KHLLLKRERV	Sequence	0.067	24174.59	25.00
4	HLA-B2705	IISPVIFQI	IISPVIFQI	0	0	0	0	0	IISPVIFQI	Sequence	0.067	24326.51	26.00
9	HLA-B2705	IFQIALDKPC	IQIALDKPC	0	0	0	1	1	IFQIALDKPC	Sequence	0.065	24655.62	26.00
27	HLA-B2705	HHLLKQLK	H-HLLKQLK	0	1	1	0	0	HHLLKQLK	Sequence	0.065	24716.78	26.00
3	HLA-B2705	GIISPVIFQI	GIISVIFQI	0	0	0	4	1	GIISPVIFQI	Sequence	0.064	24989.45	27.00
39	HLA-B2705	YLKIKHLL	Y-LKIKHLL	0	1	1	0	0	YLKIKHLL	Sequence	0.061	25804.40	29.00
21	HLA-B2705	AEVKHLHHLL	AVKHLHHLL	0	0	0	1	1	AEVKHLHHLL	Sequence	0.059	26386.87	30.00
54	HLA-B2705	LSKLQAAVNA	LKLQAAVNA	0	0	0	1	1	LSKLQAAVNA	Sequence	0.057	26864.79	31.00
2	HLA-B2705	SGIISPVIF	SGIISPVIF	0	0	0	0	0	SGIISPVIF	Sequence	0.056	27146.16	32.00
36	HLA-B2705	SEKYLKIKH	SEKYLKIKH	0	0	0	0	0	SEKYLKIKH	Sequence	0.056	27273.34	32.00
57	HLA-B2705	LQAAVNAA	LQAAVNAA-	0	8	1	0	0	LQAAVNAA	Sequence	0.055	27539.63	33.00
27	HLA-B2705	HHLLKQLKP	HHLLKQLKP	0	0	0	0	0	HHLLKQLKP	Sequence	0.055	27647.11	33.00
1	HLA-B2705	VSGIISPVI	VSGIISPVI	0	0	0	0	0	VSGIISPVI	Sequence	0.054	27834.10	33.00
32	HLA-B2705	QLKPSEKYL	QLKPSEKYL	0	0	0	0	0	QLKPSEKYL	Sequence	0.053	28144.80	34.00
6	HLA-B2705	SPVIFQIAL	SPVIFQIAL	0	0	0	0	0	SPVIFQIAL	Sequence	0.053	28196.63	34.00
53	HLA-B2705	DLSKLQAAV	DLSKLQAAV	0	0	0	0	0	DLSKLQAAV	Sequence	0.053	28206.69	34.00
26	HLA-B2705	LHHLLKQL	L-HHLLKQL	0	1	1	0	0	LHHLLKQL	Sequence	0.053	28235.10	34.00
32	HLA-B2705	QLKPSEKYLK	QLKPSKYLK	0	0	0	5	1	QLKPSEKYLK	Sequence	0.049	29283.30	37.00
47	HLA-B2705	LKRERVDL	LKRE-RVDL	0	4	1	0	0	LKRERVDL	Sequence	0.049	29435.78	38.00
3	HLA-B2705	GIISPVIFQ	GIISPVIFQ	0	0	0	0	0	GIISPVIFQ	Sequence	0.049	29504.33	38.00
16	HLA-B2705	KPCHQAEVK	KPCHQAEVK	0	0	0	0	0	KPCHQAEVK	Sequence	0.048	29713.21	38.00
58	HLA-B2705	QAAVNAAV	-QAAVNAAV	0	0	1	0	0	QAAVNAAV	Sequence	0.047	30057.89	39.00
20	HLA-B2705	QAEVKHLHHL	QAVKHLHHL	0	0	0	2	1	QAEVKHLHHL	Sequence	0.047	30149.10	40.00
15	HLA-B2705	DKPCHQAEV	DKPCHQAEV	0	0	0	0	0	DKPCHQAEV	Sequence	0.047	30228.81	40.00
34	HLA-B2705	KPSEKYLKIK	KPSEKYLKI	0	0	0	0	0	KPSEKYLKI	Sequence	0.046	30287.41	40.00
27	HLA-B2705	HHLLKQLKPS	HLLKQLKPS	0	0	0	1	1	HHLLKQLKPS	Sequence	0.045	30582.45	41.00
25	HLA-B2705	HLHHLLKQ	HLHHLLKQ-	0	8	1	0	0	HLHHLLKQ	Sequence	0.045	30789.29	42.00
5	HLA-B2705	ISPVIFQIAL	ISVIFQIAL	0	0	0	2	1	ISPVIFQIAL	Sequence	0.045	30845.99	42.00
32	HLA-B2705	QLKPSEKY	-QLKPSEKY	0	0	1	0	0	QLKPSEKY	Sequence	0.044	30953.64	42.00
45	HLA-B2705	LLLKRERVDL	LLKRERVDL	0	0	0	1	1	LLLKRERVDL	Sequence	0.044	31046.54	42.00
46	HLA-B2705	LLKRERVDLS	LKRERVDLS	0	0	0	1	1	LLKRERVDLS	Sequence	0.044	31229.14	43.00
44	HLA-B2705	HLLLKRERVD	HLLLKRERV	0	0	0	0	0	HLLLKRERV	Sequence	0.043	31425.07	44.00
16	HLA-B2705	KPCHQAEVKH	KCHQAEVKH	0	0	0	1	1	KPCHQAEVKH	Sequence	0.043	31427.44	44.00
29	HLA-B2705	LLKQLKPSE	LLKQLKPSE	0	0	0	0	0	LLKQLKPSE	Sequence	0.043	31542.24	44.00
20	HLA-B2705	QAEVKHLHH	QAEVKHLHH	0	0	0	0	0	QAEVKHLHH	Sequence	0.042	31718.14	45.00

37	HLA-B2705	EKYLKIKH	EKYLKI-KH	0	6	1	0	0	EKYLKIKH	Sequence	0.039	32660.18	48.00	
28	HLA-B2705	HLLKQLKPSE	HLLKQLKPS	0	0	0	0	0	HLLKQLKPS	Sequence	0.038	33153.65	50.00	
56	HLA-B2705	KLQAAVNA	KLQAAVNA-	0	8	1	0	0	KLQAAVNA	Sequence	0.038	33215.75	50.00	
1	HLA-B2705	VSGIISPV	VS-GIISPV	0	2	1	0	0	VSGIISPV	Sequence	0.037	33441.87	55.00	
17	HLA-B2705	PCHQAEVKH	PCHQAEVKH	0	0	0	0	0	PCHQAEVKH	Sequence	0.037	33624.37	55.00	
55	HLA-B2705	SKLQAAVN	SKLQAAVN-	0	8	1	0	0	SKLQAAVN	Sequence	0.036	33748.66	55.00	
11	HLA-B2705	QIALDKPCH	QIALDKPCH	0	0	0	0	0	QIALDKPCH	Sequence	0.036	33836.77	55.00	
20	HLA-B2705	QAEVKHLH	-QAEVKHLH	0	0	1	0	0	QAEVKHLH	Sequence	0.036	33861.68	55.00	
28	HLA-B2705	HLLKQLKP	HLLKQLKP-	0	8	1	0	0	HLLKQLKP	Sequence	0.036	33936.48	55.00	
44	HLA-B2705	HLLLKRER	HLLLKR-ER	0	6	1	0	0	HLLLKRER	Sequence	0.036	33951.55	55.00	
1	HLA-B2705	VSGIISPVIF	VSIISPVIF	0	0	0	2	1	VSGIISPVIF	Sequence	0.035	34069.31	55.00	
17	HLA-B2705	PCHQAEVKHL	CHQAEVKHL	1	0	0	0	0	CHQAEVKHL	Sequence	0.035	34200.42	55.00	
13	HLA-B2705	ALDKPCHQA	ALDKPCHQA	0	0	0	0	0	ALDKPCHQA	Sequence	0.035	34342.45	55.00	
54	HLA-B2705	LSKLQAAVN	LSKLQAAVN	0	0	0	0	0	LSKLQAAVN	Sequence	0.034	34569.10	60.00	
52	HLA-B2705	VDLSKLQAA	VDLSKLQAA	0	0	0	0	0	VDLSKLQAA	Sequence	0.034	34706.28	60.00	
35	HLA-B2705	PSEKYLKIK	PSEKYLKIK	0	0	0	0	0	PSEKYLKIK	Sequence	0.033	34855.29	60.00	
52	HLA-B2705	VDLSKLQAAV	VLSKLQAAV	0	0	0	1	1	VDLSKLQAAV	Sequence	0.032	35293.22	60.00	
11	HLA-B2705	QIALDKPC	-QIALDKPC	0	0	1	0	0	QIALDKPC	Sequence	0.032	35319.97	60.00	
58	HLA-B2705	QAAVNAAVQ	QAAVNAAVQ	0	0	0	0	0	QAAVNAAVQ	Sequence	0.032	35390.36	60.00	
3	HLA-B2705	GIISPVIF	GIISPVI-F	0	7	1	0	0	GIISPVIF	Sequence	0.032	35447.08	60.00	
14	HLA-B2705	LDKPCHQAEV	LKPCHQAEV	0	0	0	1	1	LDKPCHQAEV	Sequence	0.032	35449.75	60.00	
18	HLA-B2705	CHQAEVKH	C-HQAEVKH	0	1	1	0	0	CHQAEVKH	Sequence	0.032	35479.69	60.00	
22	HLA-B2705	EVKHLHHL	EVKHLH-HL	0	6	1	0	0	EVKHLHHL	Sequence	0.031	35582.71	60.00	
5	HLA-B2705	ISPVIFQIA	ISPVIFQIA	0	0	0	0	0	ISPVIFQIA	Sequence	0.031	35591.57	60.00	
12	HLA-B2705	IALDKPCHQ	IALDKPCHQ	0	0	0	0	0	IALDKPCHQ	Sequence	0.031	35710.00	65.00	
9	HLA-B2705	IFQIALDKP	IFQIALDKP	0	0	0	0	0	IFQIALDKP	Sequence	0.030	36023.56	65.00	
0	HLA-B2705	MVSGIISP	MVSGIISP-	0	8	1	0	0	MVSGIISP	Sequence	0.030	36120.73	65.00	
8	HLA-B2705	VIFQIALDKP	VIFQIALDK	0	0	0	0	0	VIFQIALDK	Sequence	0.029	36404.80	65.00	
4	HLA-B2705	IISPVIFQIA	IISPVIFQI	0	0	0	0	0	IISPVIFQI	Sequence	0.029	36453.67	65.00	
34	HLA-B2705	KPSEKYLK	KPSEK-YLK	0	5	1	0	0	KPSEKYLK	Sequence	0.029	36560.32	65.00	
15	HLA-B2705	DKPCHQAEVK	DKCHQAEVK	0	0	0	2	1	DKPCHQAEVK	Sequence	0.028	37063.75	70.00	
2	HLA-B2705	SGIISPVIFQ	SGIISPVIF	0	0	0	0	0	SGIISPVIF	Sequence	0.027	37164.95	70.00	
43	HLA-B2705	KHLLLKRE	-KHLLLKRE	0	0	1	0	0	KHLLLKRE	Sequence	0.027	37400.94	70.00	
9	HLA-B2705	IFQIALDK	I-FQIALDK	0	1	1	0	0	IFQIALDK	Sequence	0.026	37608.30	70.00	
7	HLA-B2705	PVIFQIALDK	PVIFQIALK	0	0	0	8	1	PVIFQIALDK	Sequence	0.026	37669.39	70.00	
30	HLA-B2705	LKQLKPSE	LKQLKPSE-	0	8	1	0	0	LKQLKPSE	Sequence	0.026	37677.54	70.00	
29	HLA-B2705	LLKQLKPS	LLKQLKPS-	0	8	1	0	0	LLKQLKPS	Sequence	0.026	37733.44	70.00	
5	HLA-B2705	ISPVIFQI	IS-PVIFQI	0	2	1	0	0	ISPVIFQI	Sequence	0.026	37736.29	70.00	
54	HLA-B2705	LSKLQAAV	LS-KLQAAV	0	2	1	0	0	LSKLQAAV	Sequence	0.025	38070.93	75.00	
45	HLA-B2705	LLLKRERV	L-LLKRERV	0	1	1	0	0	LLLKRERV	Sequence	0.025	38126.18	75.00	
6	HLA-B2705	SPVIFQIALD	SPVIFQIAL	0	0	0	0	0	SPVIFQIAL	Sequence	0.024	38382.79	75.00	
36	HLA-B2705	SEKYLKIK	SEKYLKI-K	0	7	1	0	0	SEKYLKIK	Sequence	0.024	38592.67	75.00	
33	HLA-B2705	LKPSEKYL	LK-PSEKYL	0	2	1	0	0	LKPSEKYL	Sequence	0.024	38729.45	75.00	
53	HLA-B2705	DLSKLQAAVN	DLSKLQAAV	0	0	0	0	0	DLSKLQAAV	Sequence	0.023	38934.91	80.00	
2	HLA-B2705	SGIISPVI	S-GIISPVI	0	1	1	0	0	SGIISPVI	Sequence	0.023	39146.12	80.00	
45	HLA-B2705	LLLKRERVD	LLLKRERVD	0	0	0	0	0	LLLKRERVD	Sequence	0.022	39275.52	80.00	
7	HLA-B2705	PVIFQIALD	PVIFQIALD	0	0	0	0	0	PVIFQIALD	Sequence	0.022	39500.52	80.00	
14	HLA-B2705	LDKPCHQAE	LDKPCHQAE	0	0	0	0	0	LDKPCHQAE	Sequence	0.022	39510.79	80.00	
35	HLA-B2705	PSEKYLKIKH	SEKYLKIKH	1	0	0	0	0	SEKYLKIKH	Sequence	0.021	39674.87	80.00	
16	HLA-B2705	KPCHQAEV	-KPCHQAEV	0	0	1	0	0	KPCHQAEV	Sequence	0.021	39888.35	85.00	
35	HLA-B2705	PSEKYLKI	-PSEKYLKI	0	0	1	0	0	PSEKYLKI	Sequence	0.018	41152.67	90.00	
46	HLA-B2705	LLKRERVD	LLKRERVD-	0	8	1	0	0	LLKRERVD	Sequence	0.018	41174.49	90.00	
7	HLA-B2705	PVIFQIAL	PVIF-QIAL	0	4	1	0	0	PVIFQIAL	Sequence	0.018	41192.32	90.00	
59	HLA-B2705	AAVNAAVQ	AAVNAAVQ-	0	8	1	0	0	AAVNAAVQ	Sequence	0.018	41278.88	90.00	
12	HLA-B2705	IALDKPCHQA	IALDKPCHA	0	0	0	8	1	IALDKPCHQA	Sequence	0.018	41360.68	90.00	
13	HLA-B2705	ALDKPCHQAE	ALDKPCHQA	0	0	0	0	0	ALDKPCHQA	Sequence	0.017	41540.54	90.00	
11	HLA-B2705	QIALDKPCHQ	QALDKPCHQ	0	0	0	1	1	QIALDKPCHQ	Sequence	0.017	41729.27	90.00	
53	HLA-B2705	DLSKLQAA	DLSKLQAA-	0	8	1	0	0	DLSKLQAA	Sequence	0.016	41927.95	95.00	
17	HLA-B2705	PCHQAEVK	PCHQAEV-K	0	7	1	0	0	PCHQAEVK	Sequence	0.016	42052.00	95.00	
52	HLA-B2705	VDLSKLQA	-VDLSKLQA	0	0	1	0	0	VDLSKLQA	Sequence	0.015	42520.48	95.00	
21	HLA-B2705	AEVKHLHH	AEVKHLHH-	0	8	1	0	0	AEVKHLHH	Sequence	0.015	42597.85	95.00	
4	HLA-B2705	IISPVIFQ	I-ISPVIFQ	0	1	1	0	0	IISPVIFQ	Sequence	0.014	42847.47	95.00	
6	HLA-B2705	SPVIFQIA	SPVIFQIA-	0	8	1	0	0	SPVIFQIA	Sequence	0.014	42984.91	95.00	
8	HLA-B2705	VIFQIALD	VIFQIALD-	0	8	1	0	0	VIFQIALD	Sequence	0.013	43229.78	99.00	
13	HLA-B2705	ALDKPCHQ	ALDKPCHQ-	0	8	1	0	0	ALDKPCHQ	Sequence	0.013	43429.49	99.00	
15	HLA-B2705	DKPCHQAE	DKPCHQAE-	0	8	1	0	0	DKPCHQAE	Sequence	0.013	43436.55	99.00	
12	HLA-B2705	IALDKPCH	IALDKPCH-	0	8	1	0	0	IALDKPCH	Sequence	0.012	43897.66	99.00	
14	HLA-B2705	LDKPCHQA	LDKPCHQA-	0	8	1	0	0	LDKPCHQA	Sequence	0.012	43992.29	99.00	

Protein Sequence. Allele HLA-B2705. Number of high binders 0. Number of weak binders 1. Number of peptides 177

Link to Allele Frequencies in Worldwide Populations HLA-B2705

\# Rank Threshold for Strong binding peptides 0.500
\# Rank Threshold for Weak binding peptides 2.000

pos	HLA	peptide	Core	Offset	I_pos	I_len	D_pos	D_len	iCore	Identity	1-log50k(aff)	Affinity(nM)	%Rank	BindLevel
57	HLA-B3901	LQAAVNAAV	LQAAVNAAV	0	0	0	0	0	LQAAVNAAV	Sequence	0.590	84.09	0.17	<= SB
6	HLA-B3901	SPVIFQIAL	SPVIFQIAL	0	0	0	0	0	SPVIFQIAL	Sequence	0.520	180.67	0.30	<= SB
19	HLA-B3901	HQAEVKHL	HQAEVKH-L	0	7	1	0	0	HQAEVKHL	Sequence	0.291	2140.90	1.20	<= WB
0	HLA-B3901	MVSGIISPV	MVSGIISPV	0	0	0	0	0	MVSGIISPV	Sequence	0.281	2389.44	1.30	<= WB
10	HLA-B3901	FQIALDKPC	FQIALDKPC	0	0	0	0	0	FQIALDKPC	Sequence	0.275	2553.63	1.40	<= WB
19	HLA-B3901	HQAEVKHLH	HQAEVKHLH	0	0	0	0	0	HQAEVKHLH	Sequence	0.219	4664.40	2.50	
18	HLA-B3901	CHQAEVKHL	CHQAEVKHL	0	0	0	0	0	CHQAEVKHL	Sequence	0.198	5843.66	2.50	
25	HLA-B3901	HLHHLLKQL	HLHHLLKQL	0	0	0	0	0	HLHHLLKQL	Sequence	0.182	6965.37	3.00	
21	HLA-B3901	AEVKHLHHL	AEVKHLHHL	0	0	0	0	0	AEVKHLHHL	Sequence	0.163	8547.33	3.50	
58	HLA-B3901	QAAVNAAV	-QAAVNAAV	0	0	1	0	0	QAAVNAAV	Sequence	0.159	8962.72	4.00	
37	HLA-B3901	EKYLKIKHL	EKYLKIKHL	0	0	0	0	0	EKYLKIKHL	Sequence	0.134	11743.10	5.00	

38	HLA-B3901	KYLKIKHLL	KYLKIKHLL	0	0	0	0	0	KYLKIKHLL	Sequence	0.125	12928.61	5.50
22	HLA-B3901	EVKHLHHLL	EVKHLHHLL	0	0	0	0	0	EVKHLHHLL	Sequence	0.124	13026.19	5.50
57	HLA-B3901	LQAAVNAAVQ	LQAAVNAAV	0	0	0	0	0	LQAAVNAAV	Sequence	0.110	15184.06	7.00
55	HLA-B3901	SKLQAAVNA	SKLQAAVNA	0	0	0	0	0	SKLQAAVNA	Sequence	0.109	15379.99	7.00
56	HLA-B3901	KLQAAVNAAV	LQAAVNAAV	1	0	0	0	0	LQAAVNAAV	Sequence	0.106	15892.74	7.00
37	HLA-B3901	EKYLKIKHLL	EKYKIKHLL	0	0	0	3	1	EKYLKIKHLL	Sequence	0.105	16076.76	7.50
50	HLA-B3901	ERVDLSKL	ERVDLS-KL	0	6	1	0	0	ERVDLSKL	Sequence	0.104	16154.17	7.50
2	HLA-B3901	SGIISPVIF	SGIISPVIF	0	0	0	0	0	SGIISPVIF	Sequence	0.102	16597.10	7.50
6	HLA-B3901	SPVIFQIALD	SPVIFQIAL	0	0	0	0	0	SPVIFQIAL	Sequence	0.102	16645.47	7.50
50	HLA-B3901	ERVDLSKLQ	ERVDLSKLQ	0	0	0	0	0	ERVDLSKLQ	Sequence	0.098	17239.35	8.00
5	HLA-B3901	ISPVIFQIAL	SPVIFQIAL	1	0	0	0	0	SPVIFQIAL	Sequence	0.098	17302.70	8.00
23	HLA-B3901	VKHLHHLL	VKH-LHHLL	0	3	1	0	0	VKHLHHLL	Sequence	0.097	17430.29	8.00
26	HLA-B3901	LHHLLKQL	LHHLLKQ-L	0	7	1	0	0	LHHLLKQL	Sequence	0.096	17764.81	8.50
58	HLA-B3901	QAAVNAAVQ	QAAVNAAVQ	0	0	0	0	0	QAAVNAAVQ	Sequence	0.095	17799.05	8.50
7	HLA-B3901	PVIFQIAL	-PVIFQIAL	0	0	1	0	0	PVIFQIAL	Sequence	0.095	17864.07	8.50
53	HLA-B3901	DLSKLQAAV	DLSKLQAAV	0	0	0	0	0	DLSKLQAAV	Sequence	0.095	17975.37	8.50
56	HLA-B3901	KLQAAVNAA	KLQAAVNAA	0	0	0	0	0	KLQAAVNAA	Sequence	0.088	19270.46	9.50
26	HLA-B3901	LHHLLKQLK	LHHLLKQLK	0	0	0	0	0	LHHLLKQLK	Sequence	0.079	21382.73	12.00
39	HLA-B3901	YLKIKHLL	Y-LKIKHLL	0	1	1	0	0	YLKIKHLL	Sequence	0.075	22163.85	12.00
4	HLA-B3901	IISPVIFQI	IISPVIFQI	0	0	0	0	0	IISPVIFQI	Sequence	0.075	22216.65	12.00
34	HLA-B3901	KPSEKYLKI	KPSEKYLKI	0	0	0	0	0	KPSEKYLKI	Sequence	0.075	22235.66	12.00
39	HLA-B3901	YLKIKHLLL	YLKIKHLLL	0	0	0	0	0	YLKIKHLLL	Sequence	0.074	22532.09	13.00
27	HLA-B3901	HHLLKQLKP	HHLLKQLKP	0	0	0	0	0	HHLLKQLKP	Sequence	0.071	23221.39	13.00
15	HLA-B3901	DKPCHQAEV	DKPCHQAEV	0	0	0	0	0	DKPCHQAEV	Sequence	0.066	24589.27	15.00
24	HLA-B3901	KHLHHLLKQ	KHLHHLLKQ	0	0	0	0	0	KHLHHLLKQ	Sequence	0.065	24648.68	15.00
44	HLA-B3901	HLLLKRERV	HLLLKRERV	0	0	0	0	0	HLLLKRERV	Sequence	0.060	26147.85	17.00
1	HLA-B3901	VSGIISPVI	VSGIISPVI	0	0	0	0	0	VSGIISPVI	Sequence	0.057	27068.43	19.00
40	HLA-B3901	LKIKHLLL	LKIK-HLLL	0	4	1	0	0	LKIKHLLL	Sequence	0.056	27360.25	19.00
49	HLA-B3901	RERVDLSKL	RERVDLSKL	0	0	0	0	0	RERVDLSKL	Sequence	0.056	27363.79	19.00
23	HLA-B3901	VKHLHHLLK	VKHLHHLLK	0	0	0	0	0	VKHLHHLLK	Sequence	0.055	27430.19	19.00
16	HLA-B3901	KPCHQAEVK	KPCHQAEVK	0	0	0	0	0	KPCHQAEVK	Sequence	0.054	27923.08	20.00
28	HLA-B3901	HLLKQLKPS	HLLKQLKPS	0	0	0	0	0	HLLKQLKPS	Sequence	0.053	28126.55	20.00
0	HLA-B3901	MVSGIISPV	MVSGIISPV	0	0	0	0	0	MVSGIISPV	Sequence	0.053	28184.72	20.00
24	HLA-B3901	KHLHHLLKQL	KHLHHLLQL	0	0	0	7	1	KHLHHLLKQL	Sequence	0.052	28400.85	21.00
51	HLA-B3901	RVDLSKLQA	RVDLSKLQA	0	0	0	0	0	RVDLSKLQA	Sequence	0.051	28672.87	21.00
48	HLA-B3901	KRERVDLSK	KRERVDLSK	0	0	0	0	0	KRERVDLSK	Sequence	0.050	28992.94	22.00
40	HLA-B3901	LKIKHLLLK	LKIKHLLLK	0	0	0	0	0	LKIKHLLLK	Sequence	0.049	29547.78	23.00
55	HLA-B3901	SKLQAAVNAA	SKLQAAVAA	0	0	0	7	1	SKLQAAVNAA	Sequence	0.048	29749.56	24.00
11	HLA-B3901	QIALDKPCH	QIALDKPCH	0	0	0	0	0	QIALDKPCH	Sequence	0.048	29849.19	24.00
21	HLA-B3901	AEVKHLHHLL	AEVHLHHLL	0	0	0	3	1	AEVKHLHHLL	Sequence	0.046	30265.46	25.00
13	HLA-B3901	ALDKPCHQA	ALDKPCHQA	0	0	0	0	0	ALDKPCHQA	Sequence	0.046	30350.05	25.00
25	HLA-B3901	HLHHLLKQLK	HLHHLLKQL	0	0	0	0	0	HLHHLLKQL	Sequence	0.045	30647.70	26.00
57	HLA-B3901	LQAAVNAA	LQAAVN-AA	0	6	1	0	0	LQAAVNAA	Sequence	0.045	30731.38	26.00
22	HLA-B3901	EVKHLHHL	E-VKHLHHL	0	1	1	0	0	EVKHLHHL	Sequence	0.044	30969.37	27.00
43	HLA-B3901	KHLLLKRER	KHLLLKRER	0	0	0	0	0	KHLLLKRER	Sequence	0.044	30993.51	27.00
3	HLA-B3901	GIISPVIFQ	GIISPVIFQ	0	0	0	0	0	GIISPVIFQ	Sequence	0.044	31021.71	27.00
46	HLA-B3901	LLKRERVDL	LLKRERVDL	0	0	0	0	0	LLKRERVDL	Sequence	0.044	31031.09	27.00
31	HLA-B3901	KQLKPSEKY	KQLKPSEKY	0	0	0	0	0	KQLKPSEKY	Sequence	0.043	31263.29	27.00
19	HLA-B3901	HQAEVKHLHH	HQAEVKHLH	0	0	0	0	0	HQAEVKHLH	Sequence	0.043	31372.74	28.00
10	HLA-B3901	FQIALDKPCH	FQIALDKPC	0	0	0	0	0	FQIALDKPC	Sequence	0.043	31505.06	28.00
18	HLA-B3901	CHQAEVKHLH	HQAEVKHLH	1	0	0	0	0	HQAEVKHLH	Sequence	0.042	31570.92	28.00
32	HLA-B3901	QLKPSEKYL	QLKPSEKYL	0	0	0	0	0	QLKPSEKYL	Sequence	0.042	31754.90	29.00
20	HLA-B3901	QAEVKHLHH	QAEVKHLHH	0	0	0	0	0	QAEVKHLHH	Sequence	0.042	31898.49	29.00
48	HLA-B3901	KRERVDLSKL	KRERVDLSL	0	0	0	8	1	KRERVDLSKL	Sequence	0.040	32430.94	31.00
20	HLA-B3901	QAEVKHLHHL	QEVKHLHHL	0	0	0	1	1	QAEVKHLHHL	Sequence	0.040	32431.30	31.00
30	HLA-B3901	LKQLKPSEK	LKQLKPSEK	0	0	0	0	0	LKQLKPSEK	Sequence	0.040	32432.34	31.00
9	HLA-B3901	IFQIALDKPC	FQIALDKPC	1	0	0	0	0	FQIALDKPC	Sequence	0.039	32728.10	32.00
1	HLA-B3901	VSGIISPV	-VSGIISPV	0	0	1	0	0	VSGIISPV	Sequence	0.036	33835.68	35.00
22	HLA-B3901	EVKHLHHLLK	EVKHLHHLL	0	0	0	0	0	EVKHLHHLL	Sequence	0.034	34573.96	38.00
42	HLA-B3901	IKHLLLKRE	IKHLLLKRE	0	0	0	0	0	IKHLLLKRE	Sequence	0.032	35209.31	41.00
8	HLA-B3901	VIFQIALDK	VIFQIALDK	0	0	0	0	0	VIFQIALDK	Sequence	0.032	35320.36	41.00
0	HLA-B3901	MVSGIISP	MVSGIISP-	0	8	1	0	0	MVSGIISP	Sequence	0.032	35328.77	41.00
12	HLA-B3901	IALDKPCHQ	IALDKPCHQ	0	0	0	0	0	IALDKPCHQ	Sequence	0.032	35439.39	42.00
36	HLA-B3901	SEKYLKIKHL	EKYLKIKHL	1	0	0	0	0	EKYLKIKHL	Sequence	0.032	35459.72	42.00
6	HLA-B3901	SPVIFQIA	SPV-IFQIA	0	3	1	0	0	SPVIFQIA	Sequence	0.032	35502.35	42.00
10	HLA-B3901	FQIALDKP	FQIALDKP-	0	8	1	0	0	FQIALDKP	Sequence	0.032	35518.11	42.00
41	HLA-B3901	KIKHLLLKR	KIKHLLLKR	0	0	0	0	0	KIKHLLLKR	Sequence	0.032	35532.31	42.00
52	HLA-B3901	VDLSKLQAA	VDLSKLQAA	0	0	0	0	0	VDLSKLQAA	Sequence	0.031	35568.86	42.00
5	HLA-B3901	ISPVIFQIA	ISPVIFQIA	0	0	0	0	0	ISPVIFQIA	Sequence	0.031	35585.03	42.00
17	HLA-B3901	PCHQAEVKHL	CHQAEVKHL	1	0	0	0	0	CHQAEVKHL	Sequence	0.031	35645.92	43.00
36	HLA-B3901	SEKYLKIKH	SEKYLKIKH	0	0	0	0	0	SEKYLKIKH	Sequence	0.031	35706.13	43.00
33	HLA-B3901	LKPSEKYLK	LKPSEKYLK	0	0	0	0	0	LKPSEKYLK	Sequence	0.030	35986.55	44.00
27	HLA-B3901	HHLLKQLKPS	HHLLKQLPS	0	0	0	7	1	HHLLKQLKPS	Sequence	0.030	36134.80	45.00
7	HLA-B3901	PVIFQIALD	PVIFQIALD	0	0	0	0	0	PVIFQIALD	Sequence	0.030	36327.29	46.00
25	HLA-B3901	HLHHLLKQ	-HLHHLLKQ	0	0	1	0	0	HLHHLLKQ	Sequence	0.030	36332.00	46.00
27	HLA-B3901	HHLLKQLK	HHL-LKQLK	0	3	1	0	0	HHLLKQLK	Sequence	0.029	36350.49	46.00
50	HLA-B3901	ERVDLSKLQA	ERVDLSLQA	0	0	0	6	1	ERVDLSKLQA	Sequence	0.029	36424.50	47.00
38	HLA-B3901	KYLKIKHL	-KYLKIKHL	0	0	1	0	0	KYLKIKHL	Sequence	0.029	36539.77	47.00
2	HLA-B3901	SGIISPVI	SGI-ISPVI	0	3	1	0	0	SGIISPVI	Sequence	0.028	36781.32	49.00
38	HLA-B3901	KYLKIKHLLL	KYLKIKHLL	0	0	0	0	0	KYLKIKHLL	Sequence	0.028	36843.45	49.00
3	HLA-B3901	GIISPVIF	GI-ISPVIF	0	2	1	0	0	GIISPVIF	Sequence	0.027	37161.33	55.00
39	HLA-B3901	YLKIKHLLLK	YLKIKHLLL	0	0	0	0	0	YLKIKHLLL	Sequence	0.027	37192.71	55.00
11	HLA-B3901	QIALDKPC	-QIALDKPC	0	0	1	0	0	QIALDKPC	Sequence	0.027	37206.80	55.00
20	HLA-B3901	QAEVKHLH	-QAEVKHLH	0	0	1	0	0	QAEVKHLH	Sequence	0.027	37218.08	55.00
16	HLA-B3901	KPCHQAEV	KP-CHQAEV	0	2	1	0	0	KPCHQAEV	Sequence	0.027	37242.64	55.00
33	HLA-B3901	LKPSEKYL	LKPSEKY-L	0	7	1	0	0	LKPSEKYL	Sequence	0.027	37386.38	55.00
5	HLA-B3901	ISPVIFQI	-ISPVIFQI	0	0	1	0	0	ISPVIFQI	Sequence	0.027	37491.29	55.00
47	HLA-B3901	LKRERVDL	LK-RERVDL	0	2	1	0	0	LKRERVDL	Sequence	0.027	37508.75	55.00
54	HLA-B3901	LSKLQAAV	-LSKLQAAV	0	0	1	0	0	LSKLQAAV	Sequence	0.026	37605.46	55.00
47	HLA-B3901	LKRERVDLS	LKRERVDLS	0	0	0	0	0	LKRERVDLS	Sequence	0.026	37647.39	55.00

31	HLA-B3901	KQLKPSEKYL	KQLKPSEYL	0	0	0	7	1	KQLKPSEKYL	Sequence	0.026	37726.09	55.00
51	HLA-B3901	RVDLSKLQAA	RVDLSLQAA	0	0	0	5	1	RVDLSKLQAA	Sequence	0.026	37858.58	55.00
29	HLA-B3901	LLKQLKPSE	LLKQLKPSE	0	0	0	0	0	LLKQLKPSE	Sequence	0.025	37974.27	60.00
17	HLA-B3901	PCHQAEVKH	PCHQAEVKH	0	0	0	0	0	PCHQAEVKH	Sequence	0.025	38040.89	60.00
2	HLA-B3901	SGIISPVIFQ	SGIISPVIF	0	0	0	0	0	SGIISPVIF	Sequence	0.025	38063.12	60.00
56	HLA-B3901	KLQAAVNA	KLQAAVNA-	0	8	1	0	0	KLQAAVNA	Sequence	0.025	38125.77	60.00
54	HLA-B3901	LSKLQAAVN	LSKLQAAVN	0	0	0	0	0	LSKLQAAVN	Sequence	0.025	38143.92	60.00
55	HLA-B3901	SKLQAAVN	SKLQAA-VN	0	6	1	0	0	SKLQAAVN	Sequence	0.025	38198.84	60.00
37	HLA-B3901	EKYLKIKH	EKY-LKIKH	0	3	1	0	0	EKYLKIKH	Sequence	0.025	38232.75	60.00
35	HLA-B3901	PSEKYLKI	-PSEKYLKI	0	0	1	0	0	PSEKYLKI	Sequence	0.024	38514.25	60.00
49	HLA-B3901	RERVDLSKLQ	ERVDLSKLQ	1	0	0	0	0	ERVDLSKLQ	Sequence	0.023	38818.40	65.00
51	HLA-B3901	RVDLSKLQ	-RVDLSKLQ	0	0	1	0	0	RVDLSKLQ	Sequence	0.023	38827.21	65.00
43	HLA-B3901	KHLLLKRERV	KHLLLRERV	0	0	0	5	1	KHLLLKRERV	Sequence	0.023	38862.53	65.00
3	HLA-B3901	GIISPVIFQI	GIISVIFQI	0	0	0	4	1	GIISPVIFQI	Sequence	0.023	38868.82	65.00
11	HLA-B3901	QIALDKPCHQ	QIALDPCHQ	0	0	0	5	1	QIALDKPCHQ	Sequence	0.023	38917.63	65.00
33	HLA-B3901	LKPSEKYLKI	KPSEKYLKI	1	0	0	0	0	KPSEKYLKI	Sequence	0.023	39116.89	65.00
24	HLA-B3901	KHLHHLLK	-KHLHHLLK	0	0	1	0	0	KHLHHLLK	Sequence	0.022	39263.61	65.00
1	HLA-B3901	VSGIISPVIF	SGIISPVIF	1	0	0	0	0	SGIISPVIF	Sequence	0.022	39267.87	65.00
34	HLA-B3901	KPSEKYLKIK	KPSEKYLKI	0	0	0	0	0	KPSEKYLKI	Sequence	0.022	39327.83	65.00
44	HLA-B3901	HLLLKRER	-HLLLKRER	0	0	1	0	0	HLLLKRER	Sequence	0.022	39382.32	70.00
4	HLA-B3901	IISPVIFQIA	IISPVIFQI	0	0	0	0	0	IISPVIFQI	Sequence	0.022	39580.11	70.00
28	HLA-B3901	HLLKQLKP	HLLKQLKP-	0	8	1	0	0	HLLKQLKP	Sequence	0.021	39685.59	70.00
53	HLA-B3901	DLSKLQAAVN	DLSKLQAAV	0	0	0	0	0	DLSKLQAAV	Sequence	0.021	39778.89	70.00
23	HLA-B3901	VKHLHHLLKQ	VKHLHHLLQ	0	0	0	8	1	VKHLHHLLKQ	Sequence	0.021	39822.37	70.00
45	HLA-B3901	LLLKRERVD	LLLKRERVD	0	0	0	0	0	LLLKRERVD	Sequence	0.021	39919.88	70.00
52	HLA-B3901	VDLSKLQAAV	DLSKLQAAV	1	0	0	0	0	DLSKLQAAV	Sequence	0.021	39959.62	75.00
28	HLA-B3901	HLLKQLKPSE	HLLQLKPSE	0	0	0	3	1	HLLKQLKPSE	Sequence	0.020	40105.18	75.00
54	HLA-B3901	LSKLQAAVNA	SKLQAAVNA	1	0	0	0	0	SKLQAAVNA	Sequence	0.020	40121.21	75.00
26	HLA-B3901	LHHLLKQLKP	LHHLLKQLK	0	0	0	0	0	LHHLLKQLK	Sequence	0.020	40122.09	75.00
9	HLA-B3901	IFQIALDKP	IFQIALDKP	0	0	0	0	0	IFQIALDKP	Sequence	0.020	40139.46	75.00
59	HLA-B3901	AAVNAAVQ	-AAVNAAVQ	0	0	1	0	0	AAVNAAVQ	Sequence	0.020	40238.16	75.00
45	HLA-B3901	LLLKRERVDL	LLLKRRVDL	0	0	0	5	1	LLLKRERVDL	Sequence	0.020	40353.70	75.00
41	HLA-B3901	KIKHLLLK	-KIKHLLLK	0	0	1	0	0	KIKHLLLK	Sequence	0.020	40379.91	75.00
34	HLA-B3901	KPSEKYLK	KPSE-KYLK	0	4	1	0	0	KPSEKYLK	Sequence	0.020	40380.35	75.00
18	HLA-B3901	CHQAEVKH	CHQAE-VKH	0	5	1	0	0	CHQAEVKH	Sequence	0.020	40479.20	75.00
16	HLA-B3901	KPCHQAEVKH	KPCHQAEVK	0	0	0	0	0	KPCHQAEVK	Sequence	0.020	40480.97	75.00
14	HLA-B3901	LDKPCHQAE	LDKPCHQAE	0	0	0	0	0	LDKPCHQAE	Sequence	0.019	40499.80	75.00
44	HLA-B3901	HLLLKRERVD	HLLLKRERV	0	0	0	0	0	HLLLKRERV	Sequence	0.019	40520.39	75.00
4	HLA-B3901	IISPVIFQ	-IISPVIFQ	0	0	1	0	0	IISPVIFQ	Sequence	0.019	40592.81	80.00
40	HLA-B3901	LKIKHLLLKR	LKIKHLLLK	0	0	0	0	0	LKIKHLLLK	Sequence	0.019	40595.01	80.00
21	HLA-B3901	AEVKHLHH	AEVKHLH-H	0	7	1	0	0	AEVKHLHH	Sequence	0.019	40776.82	80.00
32	HLA-B3901	QLKPSEKY	-QLKPSEKY	0	0	1	0	0	QLKPSEKY	Sequence	0.019	40778.13	80.00
35	HLA-B3901	PSEKYLKIK	PSEKYLKIK	0	0	0	0	0	PSEKYLKIK	Sequence	0.019	40804.62	80.00
12	HLA-B3901	IALDKPCH	-IALDKPCH	0	0	1	0	0	IALDKPCH	Sequence	0.019	40908.94	80.00
30	HLA-B3901	LKQLKPSEKY	LKQLKPSEY	0	0	0	8	1	LKQLKPSEKY	Sequence	0.018	40943.49	80.00
13	HLA-B3901	ALDKPCHQ	ALDK-PCHQ	0	4	1	0	0	ALDKPCHQ	Sequence	0.018	41116.17	80.00
53	HLA-B3901	DLSKLQAA	DLSKL-QAA	0	5	1	0	0	DLSKLQAA	Sequence	0.018	41188.75	85.00
41	HLA-B3901	KIKHLLLKRE	IKHLLLKRE	1	0	0	0	0	IKHLLLKRE	Sequence	0.018	41211.47	85.00
15	HLA-B3901	DKPCHQAEVK	DKPCHQAEV	0	0	0	0	0	DKPCHQAEV	Sequence	0.017	41408.59	85.00
13	HLA-B3901	ALDKPCHQAE	ALDPCHQAE	0	0	0	3	1	ALDKPCHQAE	Sequence	0.017	41462.38	85.00
42	HLA-B3901	IKHLLLKR	IKH-LLLKR	0	3	1	0	0	IKHLLLKR	Sequence	0.017	41514.02	85.00
48	HLA-B3901	KRERVDLS	KRERVDLS-	0	8	1	0	0	KRERVDLS	Sequence	0.017	41522.54	85.00
49	HLA-B3901	RERVDLSK	-RERVDLSK	0	0	1	0	0	RERVDLSK	Sequence	0.017	41610.25	85.00
43	HLA-B3901	KHLLLKRE	KHLLLKRE-	0	8	1	0	0	KHLLLKRE	Sequence	0.017	41692.25	85.00
8	HLA-B3901	VIFQIALD	-VIFQIALD	0	0	1	0	0	VIFQIALD	Sequence	0.017	41724.75	90.00
15	HLA-B3901	DKPCHQAE	DK-PCHQAE	0	2	1	0	0	DKPCHQAE	Sequence	0.017	41777.60	90.00
32	HLA-B3901	QLKPSEKYLK	QLKPSEKYL	0	0	0	0	0	QLKPSEKYL	Sequence	0.017	41779.87	90.00
31	HLA-B3901	KQLKPSEK	KQLK-PSEK	0	4	1	0	0	KQLKPSEK	Sequence	0.017	41788.00	90.00
14	HLA-B3901	LDKPCHQAEV	DKPCHQAEV	1	0	0	0	0	DKPCHQAEV	Sequence	0.016	41851.36	90.00
52	HLA-B3901	VDLSKLQA	-VDLSKLQA	0	0	1	0	0	VDLSKLQA	Sequence	0.016	41969.25	90.00
17	HLA-B3901	PCHQAEVK	-PCHQAEVK	0	0	1	0	0	PCHQAEVK	Sequence	0.016	42073.83	90.00
47	HLA-B3901	LKRERVDLSK	KRERVDLSK	1	0	0	0	0	KRERVDLSK	Sequence	0.016	42116.18	90.00
46	HLA-B3901	LLKRERVDLS	LLKRERVDL	0	0	0	0	0	LLKRERVDL	Sequence	0.016	42147.65	90.00
30	HLA-B3901	LKQLKPSE	LKQLKPSE-	0	8	1	0	0	LKQLKPSE	Sequence	0.016	42240.33	90.00
14	HLA-B3901	LDKPCHQA	-LDKPCHQA	0	0	1	0	0	LDKPCHQA	Sequence	0.015	42328.16	90.00
42	HLA-B3901	IKHLLLKRER	KHLLLKRER	1	0	0	0	0	KHLLLKRER	Sequence	0.015	42350.61	90.00
12	HLA-B3901	IALDKPCHQA	ALDKPCHQA	1	0	0	0	0	ALDKPCHQA	Sequence	0.015	42483.23	95.00
29	HLA-B3901	LLKQLKPSEK	LKQLKPSEK	1	0	0	0	0	LKQLKPSEK	Sequence	0.015	42531.53	95.00
45	HLA-B3901	LLLKRERV	LLLK-RERV	0	4	1	0	0	LLLKRERV	Sequence	0.015	42735.43	95.00
9	HLA-B3901	IFQIALDK	-IFQIALDK	0	0	1	0	0	IFQIALDK	Sequence	0.014	42992.82	95.00
7	HLA-B3901	PVIFQIALDK	PVIFQIALK	0	0	0	8	1	PVIFQIALDK	Sequence	0.014	43022.61	95.00
8	HLA-B3901	VIFQIALDKP	VIFQIALDK	0	0	0	0	0	VIFQIALDK	Sequence	0.013	43337.50	99.00
36	HLA-B3901	SEKYLKIK	S-EKYLKIK	0	1	1	0	0	SEKYLKIK	Sequence	0.013	43446.89	99.00
29	HLA-B3901	LLKQLKPS	-LLKQLKPS	0	0	1	0	0	LLKQLKPS	Sequence	0.013	43520.28	99.00
35	HLA-B3901	PSEKYLKIKH	SEKYLKIKH	1	0	0	0	0	SEKYLKIKH	Sequence	0.013	43666.50	99.00
46	HLA-B3901	LLKRERVD	LLKRERVD-	0	8	1	0	0	LLKRERVD	Sequence	0.011	44177.34	99.00

Protein Sequence. Allele HLA-B3901. Number of high binders 2. Number of weak binders 3. Number of peptides 177

Link to Allele Frequencies in Worldwide Populations HLA-B3901

Rank Threshold for Strong binding peptides   0.500
Rank Threshold for Weak binding peptides   2.000

----

pos	HLA	peptide	Core	Offset	I_pos	I_len	D_pos	D_len	iCore	Identity	1-log50k(aff)	Affinity(nM)	%Rank	BindLeve
21	HLA-B4001	AEVKHLHHL	AEVKHLHHL	0	0	0	0	0	AEVKHLHHL	Sequence	0.710	23.17	0.12	<= SB
21	HLA-B4001	AEVKHLHHLL	AEVHLHHLL	0	0	0	3	1	AEVKHLHHLL	Sequence	0.650	44.11	0.20	<= SB
49	HLA-B4001	RERVDLSKL	RERVDLSKL	0	0	0	0	0	RERVDLSKL	Sequence	0.482	272.28	0.60	<= WB

20	HLA-B4001	QAEVKHLHHL	AEVKHLHHL	1	0	0	0	0	AEVKHLHHL	Sequence	0.430	479.30	0.80	<= WB
57	HLA-B4001	LQAAVNAAV	LQAAVNAAV	0	0	0	0	0	LQAAVNAAV	Sequence	0.296	2024.13	1.70	<= WB
48	HLA-B4001	KRERVDLSKL	RERVDLSKL	1	0	0	0	0	RERVDLSKL	Sequence	0.246	3509.47	2.50	
36	HLA-B4001	SEKYLKIKHL	SEKYLKIKL	0	0	0	8	1	SEKYLKIKHL	Sequence	0.228	4227.95	3.00	
22	HLA-B4001	EVKHLHHL	-EVKHLHHL	0	0	1	0	0	EVKHLHHL	Sequence	0.212	5064.39	3.50	
49	HLA-B4001	RERVDLSKLQ	RERVDLSKL	0	0	0	0	0	RERVDLSKL	Sequence	0.184	6822.76	4.00	
56	HLA-B4001	KLQAAVNAAV	KQAAVNAAV	0	0	0	1	1	KLQAAVNAAV	Sequence	0.150	9830.14	5.50	
10	HLA-B4001	FQIALDKPC	FQIALDKPC	0	0	0	0	0	FQIALDKPC	Sequence	0.149	9952.14	5.50	
36	HLA-B4001	SEKYLKIKH	SEKYLKIKH	0	0	0	0	0	SEKYLKIKH	Sequence	0.142	10716.73	5.50	
19	HLA-B4001	HQAEVKHLH	HQAEVKHLH	0	0	0	0	0	HQAEVKHLH	Sequence	0.133	11912.92	6.50	
19	HLA-B4001	HQAEVKHL	HQAEVKH-L	0	7	1	0	0	HQAEVKHL	Sequence	0.131	12120.16	6.50	
31	HLA-B4001	KQLKPSEKYL	KQLKPSEYL	0	0	0	7	1	KQLKPSEKYL	Sequence	0.112	14803.17	8.00	
50	HLA-B4001	ERVDLSKL	-ERVDLSKL	0	0	1	0	0	ERVDLSKL	Sequence	0.103	16355.02	9.00	
21	HLA-B4001	AEVKHLHH	AEVKHLH-H	0	7	1	0	0	AEVKHLHH	Sequence	0.102	16647.28	9.50	
0	HLA-B4001	MVSGIISPV	MVSGIISPV	0	0	0	0	0	MVSGIISPV	Sequence	0.100	17003.16	9.50	
25	HLA-B4001	HLHHLLKQL	HLHHLLKQL	0	0	0	0	0	HLHHLLKQL	Sequence	0.096	17703.60	11.00	
6	HLA-B4001	SPVIFQIAL	SPVIFQIAL	0	0	0	0	0	SPVIFQIAL	Sequence	0.095	17908.01	11.00	
31	HLA-B4001	KQLKPSEKY	KQLKPSEKY	0	0	0	0	0	KQLKPSEKY	Sequence	0.093	18328.68	11.00	
49	HLA-B4001	RERVDLSK	RE-RVDLSK	0	2	1	0	0	RERVDLSK	Sequence	0.089	19011.38	12.00	
19	HLA-B4001	HQAEVKHLHH	HQAEVHLHH	0	0	0	5	1	HQAEVKHLHH	Sequence	0.086	19739.80	13.00	
2	HLA-B4001	SGIISPVIF	SGIISPVIF	0	0	0	0	0	SGIISPVIF	Sequence	0.084	20157.42	13.00	
57	HLA-B4001	LQAAVNAAVQ	LQAAVNAAV	0	0	0	0	0	LQAAVNAAV	Sequence	0.083	20424.38	13.00	
38	HLA-B4001	KYLKIKHLL	KYLKIKHLL	0	0	0	0	0	KYLKIKHLL	Sequence	0.082	20587.90	14.00	
5	HLA-B4001	ISPVIFQIAL	ISPVIFIAL	0	0	0	6	1	ISPVIFQIAL	Sequence	0.075	22195.52	16.00	
39	HLA-B4001	YLKIKHLLL	YLKIKHLLL	0	0	0	0	0	YLKIKHLLL	Sequence	0.075	22207.53	16.00	
58	HLA-B4001	QAAVNAAVQ	QAAVNAAVQ	0	0	0	0	0	QAAVNAAVQ	Sequence	0.073	22574.55	16.00	
4	HLA-B4001	IISPVIFQI	IISPVIFQI	0	0	0	0	0	IISPVIFQI	Sequence	0.073	22777.70	17.00	
22	HLA-B4001	EVKHLHHLL	EVKHLHHLL	0	0	0	0	0	EVKHLHHLL	Sequence	0.073	22798.41	17.00	
1	HLA-B4001	VSGIISPVI	VSGIISPVI	0	0	0	0	0	VSGIISPVI	Sequence	0.071	23205.31	17.00	
20	HLA-B4001	QAEVKHLHH	QAEVKHLHH	0	0	0	0	0	QAEVKHLHH	Sequence	0.070	23464.11	18.00	
58	HLA-B4001	QAAVNAAV	-QAAVNAAV	0	0	1	0	0	QAAVNAAV	Sequence	0.070	23488.74	18.00	
9	HLA-B4001	IFQIALDKP	IFQIALDKP	0	0	0	0	0	IFQIALDKP	Sequence	0.068	24023.87	19.00	
10	HLA-B4001	FQIALDKPCH	FQIALDKPH	0	0	0	8	1	FQIALDKPCH	Sequence	0.067	24095.72	19.00	
51	HLA-B4001	RVDLSKLQA	RVDLSKLQA	0	0	0	0	0	RVDLSKLQA	Sequence	0.067	24151.84	19.00	
35	HLA-B4001	PSEKYLKIKH	SEKYLKIKH	1	0	0	0	0	SEKYLKIKH	Sequence	0.065	24744.61	21.00	
9	HLA-B4001	IFQIALDKPC	FQIALDKPC	1	0	0	0	0	FQIALDKPC	Sequence	0.064	25010.28	21.00	
8	HLA-B4001	VIFQIALDK	VIFQIALDK	0	0	0	0	0	VIFQIALDK	Sequence	0.063	25238.07	22.00	
40	HLA-B4001	LKIKHLLLK	LKIKHLLLK	0	0	0	0	0	LKIKHLLLK	Sequence	0.063	25269.49	22.00	
5	HLA-B4001	ISPVIFQIA	ISPVIFQIA	0	0	0	0	0	ISPVIFQIA	Sequence	0.063	25273.87	22.00	
34	HLA-B4001	KPSEKYLKI	KPSEKYLKI	0	0	0	0	0	KPSEKYLKI	Sequence	0.063	25303.96	22.00	
3	HLA-B4001	GIISPVIFQ	GIISPVIFQ	0	0	0	0	0	GIISPVIFQ	Sequence	0.063	25342.05	22.00	
36	HLA-B4001	SEKYLKIK	SE-KYLKIK	0	2	1	0	0	SEKYLKIK	Sequence	0.063	25411.24	22.00	
23	HLA-B4001	VKHLHHLLK	VKHLHHLLK	0	0	0	0	0	VKHLHHLLK	Sequence	0.062	25459.12	22.00	
10	HLA-B4001	FQIALDKP	FQI-ALDKP	0	3	1	0	0	FQIALDKP	Sequence	0.061	25768.41	23.00	
24	HLA-B4001	KHLHHLLKQ	KHLHHLLKQ	0	0	0	0	0	KHLHHLLKQ	Sequence	0.061	25904.54	23.00	
13	HLA-B4001	ALDKPCHQA	ALDKPCHQA	0	0	0	0	0	ALDKPCHQA	Sequence	0.059	26463.49	25.00	
46	HLA-B4001	LLKRERVDL	LLKRERVDL	0	0	0	0	0	LLKRERVDL	Sequence	0.059	26480.10	25.00	
56	HLA-B4001	KLQAAVNAA	KLQAAVNAA	0	0	0	0	0	KLQAAVNAA	Sequence	0.058	26672.18	25.00	
44	HLA-B4001	HLLLKRERV	HLLLKRERV	0	0	0	0	0	HLLLKRERV	Sequence	0.056	27189.96	27.00	
41	HLA-B4001	KIKHLLLKR	KIKHLLLKR	0	0	0	0	0	KIKHLLLKR	Sequence	0.056	27282.19	27.00	
48	HLA-B4001	KRERVDLSK	KRERVDLSK	0	0	0	0	0	KRERVDLSK	Sequence	0.056	27421.29	28.00	
14	HLA-B4001	LDKPCHQAE	LDKPCHQAE	0	0	0	0	0	LDKPCHQAE	Sequence	0.055	27487.83	28.00	
18	HLA-B4001	CHQAEVKHLH	HQAEVKHLH	1	0	0	0	0	HQAEVKHLH	Sequence	0.055	27505.97	28.00	
1	HLA-B4001	VSGIISPVIF	VSIISPVIF	0	0	0	2	1	VSGIISPVIF	Sequence	0.055	27666.86	28.00	
52	HLA-B4001	VDLSKLQAA	VDLSKLQAA	0	0	0	0	0	VDLSKLQAA	Sequence	0.053	28085.18	30.00	
55	HLA-B4001	SKLQAAVNA	SKLQAAVNA	0	0	0	0	0	SKLQAAVNA	Sequence	0.053	28233.26	30.00	
0	HLA-B4001	MVSGIISPVI	MVSGIISPI	0	0	0	8	1	MVSGIISPVI	Sequence	0.053	28290.44	30.00	
32	HLA-B4001	QLKPSEKYL	QLKPSEKYL	0	0	0	0	0	QLKPSEKYL	Sequence	0.052	28462.99	31.00	
24	HLA-B4001	KHLHHLLKQL	KHLHHLLQL	0	0	0	7	1	KHLHHLLKQL	Sequence	0.052	28463.30	31.00	
37	HLA-B4001	EKYLKIKHL	EKYLKIKHL	0	0	0	0	0	EKYLKIKHL	Sequence	0.052	28572.53	31.00	
42	HLA-B4001	IKHLLLKRE	IKHLLLKRE	0	0	0	0	0	IKHLLLKRE	Sequence	0.051	28781.98	32.00	
3	HLA-B4001	GIISPVIFQI	GIISVIFQI	0	0	0	4	1	GIISPVIFQI	Sequence	0.050	28990.12	33.00	
28	HLA-B4001	HLLKQLKPS	HLLKQLKPS	0	0	0	0	0	HLLKQLKPS	Sequence	0.050	29002.98	33.00	
38	HLA-B4001	KYLKIKHLLL	KYLKIHLLL	0	0	0	5	1	KYLKIKHLLL	Sequence	0.050	29201.36	33.00	
3	HLA-B4001	GIISPVIF	G-IISPVIF	0	1	1	0	0	GIISPVIF	Sequence	0.049	29295.35	34.00	
29	HLA-B4001	LLKQLKPSE	LLKQLKPSE	0	0	0	0	0	LLKQLKPSE	Sequence	0.047	29962.77	36.00	
16	HLA-B4001	KPCHQAEVK	KPCHQAEVK	0	0	0	0	0	KPCHQAEVK	Sequence	0.047	30047.81	36.00	
40	HLA-B4001	LKIKHLLL	LKIKHLL-L	0	7	1	0	0	LKIKHLLL	Sequence	0.047	30098.26	37.00	
18	HLA-B4001	CHQAEVKHL	CHQAEVKHL	0	0	0	0	0	CHQAEVKHL	Sequence	0.047	30098.59	37.00	
17	HLA-B4001	PCHQAEVKH	PCHQAEVKH	0	0	0	0	0	PCHQAEVKH	Sequence	0.047	30141.93	37.00	
30	HLA-B4001	LKQLKPSEKY	LQLKPSEKY	0	0	0	1	1	LKQLKPSEKY	Sequence	0.046	30436.22	38.00	
11	HLA-B4001	QIALDKPCH	QIALDKPCH	0	0	0	0	0	QIALDKPCH	Sequence	0.046	30449.39	38.00	
8	HLA-B4001	VIFQIALDKP	VIFQILDKP	0	0	0	5	1	VIFQIALDKP	Sequence	0.046	30521.96	38.00	
57	HLA-B4001	LQAAVNAA	LQAAVNAA-	0	8	1	0	0	LQAAVNAA	Sequence	0.045	30684.21	39.00	
30	HLA-B4001	LKQLKPSEK	LKQLKPSEK	0	0	0	0	0	LKQLKPSEK	Sequence	0.045	30829.96	39.00	
23	HLA-B4001	VKHLHHLL	VKHLHHL-L	0	7	1	0	0	VKHLHHLL	Sequence	0.044	30945.93	40.00	
26	HLA-B4001	LHHLLKQLK	LHHLLKQLK	0	0	0	0	0	LHHLLKQLK	Sequence	0.044	31048.22	40.00	
51	HLA-B4001	RVDLSKLQAA	RVDLSLQAA	0	0	0	5	1	RVDLSKLQAA	Sequence	0.044	31134.34	41.00	
35	HLA-B4001	PSEKYLKIK	PSEKYLKIK	0	0	0	0	0	PSEKYLKIK	Sequence	0.043	31445.81	42.00	
37	HLA-B4001	EKYLKIKH	-EKYLKIKH	0	0	1	0	0	EKYLKIKH	Sequence	0.041	32029.21	44.00	
43	HLA-B4001	KHLLLKRER	KHLLLKRER	0	0	0	0	0	KHLLLKRER	Sequence	0.041	32085.40	44.00	
39	HLA-B4001	YLKIKHLLLK	YLIKHLLLK	0	0	0	2	1	YLKIKHLLLK	Sequence	0.041	32212.37	45.00	
33	HLA-B4001	LKPSEKYLK	LKPSEKYLK	0	0	0	0	0	LKPSEKYLK	Sequence	0.040	32526.16	46.00	
47	HLA-B4001	LKRERVDLS	LKRERVDLS	0	0	0	0	0	LKRERVDLS	Sequence	0.040	32582.89	46.00	
25	HLA-B4001	HLHHLLKQLK	HLHHLLKQL	0	0	0	0	0	HLHHLLKQL	Sequence	0.039	32620.27	47.00	
4	HLA-B4001	IISPVIFQIA	ISPVIFQIA	0	0	0	1	1	IISPVIFQIA	Sequence	0.039	32650.29	47.00	
12	HLA-B4001	IALDKPCHQ	IALDKPCHQ	0	0	0	0	0	IALDKPCHQ	Sequence	0.039	32651.00	47.00	
54	HLA-B4001	LSKLQAAVN	LSKLQAAVN	0	0	0	0	0	LSKLQAAVN	Sequence	0.039	32733.42	47.00	
27	HLA-B4001	HHLLKQLKP	HHLLKQLKP	0	0	0	0	0	HHLLKQLKP	Sequence	0.039	32859.03	48.00	
23	HLA-B4001	VKHLHHLLKQ	VKHHHLLKQ	0	0	0	3	1	VKHLHHLLKQ	Sequence	0.039	32933.41	48.00	

13	HLA-B4001	ALDKPCHQAE	ALDKCHQAE	0	0	0	4	1	ALDKPCHQAE	Sequence	0.039	32949.81	48.00
16	HLA-B4001	KPCHQAEVKH	KCHQAEVKH	0	0	0	1	1	KPCHQAEVKH	Sequence	0.038	33127.83	49.00
45	HLA-B4001	LLLKRERVD	LLLKRERVD	0	0	0	0	0	LLLKRERVD	Sequence	0.038	33133.21	49.00
15	HLA-B4001	DKPCHQAEV	DKPCHQAEV	0	0	0	0	0	DKPCHQAEV	Sequence	0.038	33151.85	49.00
37	HLA-B4001	EKYLKIKHLL	EKYLIKHLL	0	0	0	4	1	EKYLKIKHLL	Sequence	0.038	33295.64	50.00
50	HLA-B4001	ERVDLSKLQ	ERVDLSKLQ	0	0	0	0	0	ERVDLSKLQ	Sequence	0.037	33565.11	55.00
39	HLA-B4001	YLKIKHLL	Y-LKIKHLL	0	1	1	0	0	YLKIKHLL	Sequence	0.037	33636.02	55.00
26	HLA-B4001	LHHLLKQL	L-HHLLKQL	0	1	1	0	0	LHHLLKQL	Sequence	0.036	33876.33	55.00
5	HLA-B4001	ISPVIFQI	I-SPVIFQI	0	1	1	0	0	ISPVIFQI	Sequence	0.036	33895.76	55.00
2	HLA-B4001	SGIISPVIFQ	GIISPVIFQ	1	0	0	0	0	GIISPVIFQ	Sequence	0.036	33899.80	55.00
25	HLA-B4001	HLHHLLKQ	HLHH-LLKQ	0	4	1	0	0	HLHHLLKQ	Sequence	0.036	34018.48	55.00
1	HLA-B4001	VSGIISPV	V-SGIISPV	0	1	1	0	0	VSGIISPV	Sequence	0.035	34100.65	55.00
40	HLA-B4001	LKIKHLLLKR	LKIHLLLKR	0	0	0	3	1	LKIKHLLLKR	Sequence	0.035	34201.15	55.00
33	HLA-B4001	LKPSEKYLKI	LKSEKYLKI	0	0	0	2	1	LKPSEKYLKI	Sequence	0.035	34201.90	55.00
2	HLA-B4001	SGIISPVI	S-GIISPVI	0	1	1	0	0	SGIISPVI	Sequence	0.035	34344.66	55.00
52	HLA-B4001	VDLSKLQAAV	VDLSKLQAV	0	0	0	7	1	VDLSKLQAAV	Sequence	0.034	34506.34	60.00
20	HLA-B4001	QAEVKHLH	-QAEVKHLH	0	0	1	0	0	QAEVKHLH	Sequence	0.034	34529.11	60.00
53	HLA-B4001	DLSKLQAAV	DLSKLQAAV	0	0	0	0	0	DLSKLQAAV	Sequence	0.034	34548.53	60.00
14	HLA-B4001	LDKPCHQAEV	LDKPCHQAV	0	0	0	8	1	LDKPCHQAEV	Sequence	0.034	34585.20	60.00
11	HLA-B4001	QIALDKPC	-QIALDKPC	0	0	1	0	0	QIALDKPC	Sequence	0.034	34615.15	60.00
22	HLA-B4001	EVKHLHHLLK	EVHLHHLLK	0	0	0	2	1	EVKHLHHLLK	Sequence	0.034	34762.26	60.00
34	HLA-B4001	KPSEKYLKIK	KSEKYLKIK	0	0	0	1	1	KPSEKYLKIK	Sequence	0.033	34981.87	60.00
7	HLA-B4001	PVIFQIALD	PVIFQIALD	0	0	0	0	0	PVIFQIALD	Sequence	0.033	35117.62	60.00
55	HLA-B4001	SKLQAAVNAA	SLQAAVNAA	0	0	0	1	1	SKLQAAVNAA	Sequence	0.032	35183.05	60.00
45	HLA-B4001	LLLKRERVDL	LLLKERVDL	0	0	0	4	1	LLLKRERVDL	Sequence	0.032	35202.84	60.00
32	HLA-B4001	QLKPSEKY	-QLKPSEKY	0	0	1	0	0	QLKPSEKY	Sequence	0.032	35224.18	60.00
28	HLA-B4001	HLLKQLKPSE	HLLKQLKPE	0	0	0	8	1	HLLKQLKPSE	Sequence	0.032	35294.38	65.00
50	HLA-B4001	ERVDLSKLQA	RVDLSKLQA	1	0	0	0	0	RVDLSKLQA	Sequence	0.032	35349.78	65.00
31	HLA-B4001	KQLKPSEK	KQLK-PSEK	0	4	1	0	0	KQLKPSEK	Sequence	0.031	35733.96	65.00
7	HLA-B4001	PVIFQIALDK	VIFQIALDK	1	0	0	0	0	VIFQIALDK	Sequence	0.031	35944.12	65.00
9	HLA-B4001	IFQIALDK	I-FQIALDK	0	1	1	0	0	IFQIALDK	Sequence	0.030	35953.07	65.00
41	HLA-B4001	KIKHLLLKRE	KIKHLLLKE	0	0	0	8	1	KIKHLLLKRE	Sequence	0.030	36003.68	65.00
42	HLA-B4001	IKHLLLKRER	IKHLLLRER	0	0	0	6	1	IKHLLLKRER	Sequence	0.030	36164.93	70.00
0	HLA-B4001	MVSGIISP	M-VSGIISP	0	1	1	0	0	MVSGIISP	Sequence	0.030	36209.94	70.00
12	HLA-B4001	IALDKPCHQA	ALDKPCHQA	1	0	0	0	0	ALDKPCHQA	Sequence	0.030	36273.87	70.00
17	HLA-B4001	PCHQAEVKHL	PCHQAEVKL	0	0	0	8	1	PCHQAEVKHL	Sequence	0.030	36304.11	70.00
47	HLA-B4001	LKRERVDLSK	LKERVDLSK	0	0	0	2	1	LKRERVDLSK	Sequence	0.029	36353.63	70.00
59	HLA-B4001	AAVNAAVQ	A-AVNAAVQ	0	1	1	0	0	AAVNAAVQ	Sequence	0.029	36381.96	70.00
6	HLA-B4001	SPVIFQIALD	SPVIFQIAL	0	0	0	0	0	SPVIFQIAL	Sequence	0.029	36532.65	70.00
41	HLA-B4001	KIKHLLLK	K-IKHLLLK	0	1	1	0	0	KIKHLLLK	Sequence	0.029	36554.78	70.00
24	HLA-B4001	KHLHHLLK	K-HLHHLLK	0	1	1	0	0	KHLHHLLK	Sequence	0.029	36640.31	70.00
51	HLA-B4001	RVDLSKLQ	RVDLSKL-Q	0	7	1	0	0	RVDLSKLQ	Sequence	0.029	36668.87	70.00
43	HLA-B4001	KHLLLKRERV	HLLLKRERV	1	0	0	0	0	HLLLKRERV	Sequence	0.029	36693.07	70.00
38	HLA-B4001	KYLKIKHL	KYLKIKH-L	0	7	1	0	0	KYLKIKHL	Sequence	0.028	36892.12	75.00
29	HLA-B4001	LLKQLKPSEK	LLKQLKPSK	0	0	0	8	1	LLKQLKPSEK	Sequence	0.028	37017.67	75.00
6	HLA-B4001	SPVIFQIA	S-PVIFQIA	0	1	1	0	0	SPVIFQIA	Sequence	0.028	37050.94	75.00
47	HLA-B4001	LKRERVDL	LKRERVD-L	0	7	1	0	0	LKRERVDL	Sequence	0.027	37459.67	80.00
42	HLA-B4001	IKHLLLKR	IKHL-LLKR	0	4	1	0	0	IKHLLLKR	Sequence	0.027	37488.46	80.00
13	HLA-B4001	ALDKPCHQ	ALDKPCH-Q	0	7	1	0	0	ALDKPCHQ	Sequence	0.026	37720.37	80.00
54	HLA-B4001	LSKLQAAVNA	LKLQAAVNA	0	0	0	1	1	LSKLQAAVNA	Sequence	0.026	37928.67	80.00
27	HLA-B4001	HHLLKQLKPS	HLLKQLKPS	0	0	0	1	1	HHLLKQLKPS	Sequence	0.025	37973.43	80.00
18	HLA-B4001	CHQAEVKH	-CHQAEVKH	0	0	1	0	0	CHQAEVKH	Sequence	0.025	37985.78	80.00
46	HLA-B4001	LLKRERVDLS	LLKRERVDL	0	0	0	0	0	LLKRERVDL	Sequence	0.025	38015.36	85.00
4	HLA-B4001	IISPVIFQ	I-ISPVIFQ	0	1	1	0	0	IISPVIFQ	Sequence	0.025	38019.89	85.00
32	HLA-B4001	QLKPSEKYLK	QLPSEKYLK	0	0	0	2	1	QLKPSEKYLK	Sequence	0.025	38047.04	85.00
56	HLA-B4001	KLQAAVNA	KLQAAVNA-	0	8	1	0	0	KLQAAVNA	Sequence	0.025	38100.20	85.00
8	HLA-B4001	VIFQIALD	VIFQIAL-D	0	7	1	0	0	VIFQIALD	Sequence	0.025	38197.19	85.00
7	HLA-B4001	PVIFQIAL	-PVIFQIAL	0	0	1	0	0	PVIFQIAL	Sequence	0.025	38202.56	85.00
16	HLA-B4001	KPCHQAEV	K-PCHQAEV	0	1	1	0	0	KPCHQAEV	Sequence	0.025	38296.94	85.00
43	HLA-B4001	KHLLLKRE	K-HLLLKRE	0	1	1	0	0	KHLLLKRE	Sequence	0.025	38302.71	85.00
28	HLA-B4001	HLLKQLKP	HLLKQLKP-	0	8	1	0	0	HLLKQLKP	Sequence	0.024	38386.52	85.00
11	HLA-B4001	QIALDKPCHQ	QIALDKPHQ	0	0	0	7	1	QIALDKPCHQ	Sequence	0.024	38451.79	85.00
14	HLA-B4001	LDKPCHQA	L-DKPCHQA	0	1	1	0	0	LDKPCHQA	Sequence	0.024	38635.28	90.00
44	HLA-B4001	HLLLKRERVD	HLLLKRERV	0	0	0	0	0	HLLLKRERV	Sequence	0.024	38667.48	90.00
52	HLA-B4001	VDLSKLQA	V-DLSKLQA	0	1	1	0	0	VDLSKLQA	Sequence	0.024	38671.67	90.00
44	HLA-B4001	HLLLKRER	HL-LLKRER	0	2	1	0	0	HLLLKRER	Sequence	0.024	38724.01	90.00
33	HLA-B4001	LKPSEKYL	LKPSEKY-L	0	7	1	0	0	LKPSEKYL	Sequence	0.024	38753.76	90.00
26	HLA-B4001	LHHLLKQLKP	LHHLLKLKP	0	0	0	6	1	LHHLLKQLKP	Sequence	0.023	38851.58	90.00
45	HLA-B4001	LLLKRERV	LLLKR-ERV	0	5	1	0	0	LLLKRERV	Sequence	0.023	39122.82	90.00
29	HLA-B4001	LLKQLKPS	LLKQ-LKPS	0	4	1	0	0	LLKQLKPS	Sequence	0.023	39171.55	90.00
54	HLA-B4001	LSKLQAAV	L-SKLQAAV	0	1	1	0	0	LSKLQAAV	Sequence	0.022	39389.56	95.00
35	HLA-B4001	PSEKYLKI	-PSEKYLKI	0	0	1	0	0	PSEKYLKI	Sequence	0.022	39450.95	95.00
15	HLA-B4001	DKPCHQAEVK	KPCHQAEVK	1	0	0	0	0	KPCHQAEVK	Sequence	0.022	39519.76	95.00
27	HLA-B4001	HHLLKQLK	H-HLLKQLK	0	1	1	0	0	HHLLKQLK	Sequence	0.021	39710.50	95.00
48	HLA-B4001	KRERVDLS	KRERVDLS-	0	8	1	0	0	KRERVDLS	Sequence	0.021	39745.32	95.00
17	HLA-B4001	PCHQAEVK	PCHQA-EVK	0	5	1	0	0	PCHQAEVK	Sequence	0.020	40055.73	95.00
15	HLA-B4001	DKPCHQAE	-DKPCHQAE	0	0	1	0	0	DKPCHQAE	Sequence	0.020	40302.23	99.00
34	HLA-B4001	KPSEKYLK	K-PSEKYLK	0	1	1	0	0	KPSEKYLK	Sequence	0.020	40308.34	99.00
30	HLA-B4001	LKQLKPSE	LK-QLKPSE	0	2	1	0	0	LKQLKPSE	Sequence	0.020	40399.58	99.00
53	HLA-B4001	DLSKLQAA	-DLSKLQAA	0	0	1	0	0	DLSKLQAA	Sequence	0.020	40426.68	99.00
12	HLA-B4001	IALDKPCH	I-ALDKPCH	0	1	1	0	0	IALDKPCH	Sequence	0.019	40510.33	99.00
55	HLA-B4001	SKLQAAVN	SKLQAAVN-	0	8	1	0	0	SKLQAAVN	Sequence	0.019	40546.71	99.00
46	HLA-B4001	LLKRERVD	LLKRERVD-	0	8	1	0	0	LLKRERVD	Sequence	0.018	41071.70	99.00
53	HLA-B4001	DLSKLQAAVN	LSKLQAAVN	1	0	0	0	0	LSKLQAAVN	Sequence	0.018	41339.21	99.00

Protein Sequence. Allele HLA-B4001. Number of high binders 2. Number of weak binders 3. Number of peptides 177

Link to Allele Frequencies in Worldwide Populations HLA-B4001

Rank Threshold for Strong binding peptides 0.500
Rank Threshold for Weak binding peptides 2.000

pos	HLA	peptide	Core	Offset	I_pos	I_len	D_pos	D_len	iCore	Identity	1-log50k(aff)	Affinity(nM)	%Rank	BindLeve
4	HLA-B5801	IISPVIFQI	IISPVIFQI	0	0	0	0	0	IISPVIFQI	Sequence	0.610	68.26	0.30	<= SB
5	HLA-B5801	ISPVIFQI	IS-PVIFQI	0	2	1	0	0	ISPVIFQI	Sequence	0.458	353.12	0.80	<= WB
1	HLA-B5801	VSGIISPVI	VSGIISPVI	0	0	0	0	0	VSGIISPVI	Sequence	0.369	925.84	1.40	<= WB
0	HLA-B5801	MVSGIISPVI	MVSGIIPVI	0	0	0	6	1	MVSGIISPVI	Sequence	0.340	1257.54	1.70	<= WB
3	HLA-B5801	GIISPVIFQI	GISPVIFQI	0	0	0	1	1	GIISPVIFQI	Sequence	0.332	1380.97	1.80	<= WB
1	HLA-B5801	VSGIISPVIF	VSIISPVIF	0	0	0	2	1	VSGIISPVIF	Sequence	0.322	1540.37	1.90	<= WB
4	HLA-B5801	IISPVIFQIA	IISPVIFQI	0	0	0	0	0	IISPVIFQI	Sequence	0.295	2057.02	2.50	
0	HLA-B5801	MVSGIISPV	MVSGIISPV	0	0	0	0	0	MVSGIISPV	Sequence	0.213	5007.40	4.50	
31	HLA-B5801	KQLKPSEKY	KQLKPSEKY	0	0	0	0	0	KQLKPSEKY	Sequence	0.195	6059.29	5.50	
2	HLA-B5801	SGIISPVIF	SGIISPVIF	0	0	0	0	0	SGIISPVIF	Sequence	0.186	6692.69	6.00	
20	HLA-B5801	QAEVKHLHHL	QAVKHLHHL	0	0	0	2	1	QAEVKHLHHL	Sequence	0.178	7267.55	6.50	
5	HLA-B5801	ISPVIFQIAL	ISVIFQIAL	0	0	0	2	1	ISPVIFQIAL	Sequence	0.170	7967.29	7.00	
38	HLA-B5801	KYLKIKHLL	KYLKIKHLL	0	0	0	0	0	KYLKIKHLL	Sequence	0.150	9903.48	9.00	
58	HLA-B5801	QAAVNAAVQ	QAAVNAAVQ	0	0	0	0	0	QAAVNAAVQ	Sequence	0.149	10010.13	9.00	
34	HLA-B5801	KPSEKYLKI	KPSEKYLKI	0	0	0	0	0	KPSEKYLKI	Sequence	0.140	10998.89	10.00	
19	HLA-B5801	HQAEVKHLH	HQAEVKHLH	0	0	0	0	0	HQAEVKHLH	Sequence	0.118	13895.23	13.00	
57	HLA-B5801	LQAAVNAAVQ	LAAVNAAVQ	0	0	0	1	1	LQAAVNAAVQ	Sequence	0.117	14109.45	14.00	
4	HLA-B5801	IISPVIFQ	IISPVIFQ-	0	8	1	0	0	IISPVIFQ	Sequence	0.116	14292.76	14.00	
12	HLA-B5801	IALDKPCHQ	IALDKPCHQ	0	0	0	0	0	IALDKPCHQ	Sequence	0.111	15067.37	15.00	
54	HLA-B5801	LSKLQAAVN	LSKLQAAVN	0	0	0	0	0	LSKLQAAVN	Sequence	0.101	16763.86	17.00	
19	HLA-B5801	HQAEVKHLHH	HQAEVKLHH	0	0	0	6	1	HQAEVKHLHH	Sequence	0.100	16916.73	18.00	
33	HLA-B5801	LKPSEKYLKI	LKSEKYLKI	0	0	0	2	1	LKPSEKYLKI	Sequence	0.099	17047.74	18.00	
52	HLA-B5801	VDLSKLQAAV	VLSKLQAAV	0	0	0	1	1	VDLSKLQAAV	Sequence	0.097	17536.22	19.00	
57	HLA-B5801	LQAAVNAAV	LQAAVNAAV	0	0	0	0	0	LQAAVNAAV	Sequence	0.094	18130.85	20.00	
54	HLA-B5801	LSKLQAAVNA	LSLQAAVNA	0	0	0	2	1	LSKLQAAVNA	Sequence	0.092	18534.49	20.00	
51	HLA-B5801	RVDLSKLQA	RVDLSKLQA	0	0	0	0	0	RVDLSKLQA	Sequence	0.091	18609.84	20.00	
31	HLA-B5801	KQLKPSEKYL	KQLPSEKYL	0	0	0	3	1	KQLKPSEKYL	Sequence	0.091	18726.58	21.00	
56	HLA-B5801	KLQAAVNAAV	KLAAVNAAV	0	0	0	2	1	KLQAAVNAAV	Sequence	0.090	18975.62	21.00	
32	HLA-B5801	QLKPSEKYL	QLKPSEKYL	0	0	0	0	0	QLKPSEKYL	Sequence	0.089	19004.59	21.00	
34	HLA-B5801	KPSEKYLKIK	KPSEKYLKI	0	0	0	0	0	KPSEKYLKI	Sequence	0.085	19994.51	23.00	
56	HLA-B5801	KLQAAVNAA	KLQAAVNAA	0	0	0	0	0	KLQAAVNAA	Sequence	0.083	20334.21	24.00	
38	HLA-B5801	KYLKIKHLLL	KYLKIKHLL	0	0	0	7	1	KYLKIKHLLL	Sequence	0.083	20463.54	24.00	
25	HLA-B5801	HLHHLLKQL	HLHHLLKQL	0	0	0	0	0	HLHHLLKQL	Sequence	0.082	20489.90	24.00	
20	HLA-B5801	QAEVKHLHH	QAEVKHLHH	0	0	0	0	0	QAEVKHLHH	Sequence	0.081	20783.52	25.00	
22	HLA-B5801	EVKHLHHLL	EVKHLHHLL	0	0	0	0	0	EVKHLHHLL	Sequence	0.081	20842.07	25.00	
12	HLA-B5801	IALDKPCHQA	IALDKPCHA	0	0	0	8	1	IALDKPCHQA	Sequence	0.078	21509.89	26.00	
21	HLA-B5801	AEVKHLHHL	AEVKHLHHL	0	0	0	0	0	AEVKHLHHL	Sequence	0.077	21723.44	27.00	
18	HLA-B5801	CHQAEVKHLH	CQAEVKHLH	0	0	0	1	1	CHQAEVKHLH	Sequence	0.076	21905.41	27.00	
5	HLA-B5801	ISPVIFQIA	ISPVIFQIA	0	0	0	0	0	ISPVIFQIA	Sequence	0.074	22351.44	28.00	
53	HLA-B5801	DLSKLQAAV	DLSKLQAAV	0	0	0	0	0	DLSKLQAAV	Sequence	0.074	22407.38	28.00	
3	HLA-B5801	GIISPVIFQ	GIISPVIFQ	0	0	0	0	0	GIISPVIFQ	Sequence	0.073	22744.21	29.00	
9	HLA-B5801	IFQIALDKPC	IFQIALDPC	0	0	0	7	1	IFQIALDKPC	Sequence	0.072	23004.83	30.00	
2	HLA-B5801	SGIISPVI	-SGIISPVI	0	0	1	0	0	SGIISPVI	Sequence	0.071	23209.83	30.00	
53	HLA-B5801	DLSKLQAAVN	DLSKQAAVN	0	0	0	6	1	DLSKLQAAVN	Sequence	0.071	23293.87	31.00	
2	HLA-B5801	SGIISPVIFQ	SGIISPVIF	0	0	0	0	0	SGIISPVIF	Sequence	0.068	23836.42	32.00	
46	HLA-B5801	LLKRERVDL	LLKRERVDL	0	0	0	0	0	LLKRERVDL	Sequence	0.068	23968.31	32.00	
1	HLA-B5801	VSGIISPV	VS-GIISPV	0	2	1	0	0	VSGIISPV	Sequence	0.068	23974.79	32.00	
21	HLA-B5801	AEVKHLHHLL	AVKHLHHLL	0	0	0	1	1	AEVKHLHHLL	Sequence	0.068	24036.09	33.00	
39	HLA-B5801	YLKIKHLLL	YLKIKHLLL	0	0	0	0	0	YLKIKHLLL	Sequence	0.067	24131.72	33.00	
6	HLA-B5801	SPVIFQIAL	SPVIFQIAL	0	0	0	0	0	SPVIFQIAL	Sequence	0.067	24170.40	33.00	
20	HLA-B5801	QAEVKHLH	QA-EVKHLH	0	2	1	0	0	QAEVKHLH	Sequence	0.067	24287.57	33.00	
10	HLA-B5801	FQIALDKPC	FQIALDKPC	0	0	0	0	0	FQIALDKPC	Sequence	0.066	24484.68	34.00	
0	HLA-B5801	MVSGIISP	MVSGIISP-	0	8	1	0	0	MVSGIISP	Sequence	0.066	24501.37	34.00	
9	HLA-B5801	IFQIALDKP	IFQIALDKP	0	0	0	0	0	IFQIALDKP	Sequence	0.063	25176.98	36.00	
11	HLA-B5801	QIALDKPCHQ	QALDKPCHQ	0	0	0	1	1	QIALDKPCHQ	Sequence	0.063	25324.78	36.00	
54	HLA-B5801	LSKLQAAV	LS-KLQAAV	0	2	1	0	0	LSKLQAAV	Sequence	0.062	25470.70	37.00	
24	HLA-B5801	KHLHHLLKQL	KLHHLLKQL	0	0	0	1	1	KHLHHLLKQL	Sequence	0.062	25607.77	37.00	
41	HLA-B5801	KIKHLLLKR	KIKHLLLKR	0	0	0	0	0	KIKHLLLKR	Sequence	0.062	25687.41	38.00	
13	HLA-B5801	ALDKPCHQA	ALDKPCHQA	0	0	0	0	0	ALDKPCHQA	Sequence	0.061	25794.07	38.00	
37	HLA-B5801	EKYLKIKHLL	EKYKIKHLL	0	0	0	3	1	EKYLKIKHLL	Sequence	0.061	25839.60	38.00	
58	HLA-B5801	QAAVNAAV	QAAVNAAV-	0	8	1	0	0	QAAVNAAV	Sequence	0.061	25889.69	38.00	
48	HLA-B5801	KRERVDLSKL	KRERVDLSL	0	0	0	8	1	KRERVDLSKL	Sequence	0.060	26008.75	39.00	
16	HLA-B5801	KPCHQAEVKH	KCHQAEVKH	0	0	0	1	1	KPCHQAEVKH	Sequence	0.060	26172.48	39.00	
24	HLA-B5801	KHLHHLLKQ	KHLHHLLKQ	0	0	0	0	0	KHLHHLLKQ	Sequence	0.059	26491.56	40.00	
8	HLA-B5801	VIFQIALDKP	VIFQILDKP	0	0	0	5	1	VIFQIALDKP	Sequence	0.058	26578.27	41.00	
49	HLA-B5801	RERVDLSKL	RERVDLSKL	0	0	0	0	0	RERVDLSKL	Sequence	0.057	26926.19	42.00	
8	HLA-B5801	VIFQIALDK	VIFQIALDK	0	0	0	0	0	VIFQIALDK	Sequence	0.057	26983.93	42.00	
32	HLA-B5801	QLKPSEKYLK	QLKPSEKYL	0	0	0	0	0	QLKPSEKYL	Sequence	0.057	27080.74	42.00	
3	HLA-B5801	GIISPVIF	GIIS-PVIF	0	4	1	0	0	GIISPVIF	Sequence	0.056	27219.10	43.00	
35	HLA-B5801	PSEKYLKIKH	PSKYLKIKH	0	0	0	2	1	PSEKYLKIKH	Sequence	0.056	27360.53	43.00	
18	HLA-B5801	CHQAEVKHL	CHQAEVKHL	0	0	0	0	0	CHQAEVKHL	Sequence	0.055	27661.16	44.00	
59	HLA-B5801	AAVNAAVQ	AAVNAAVQ-	0	8	1	0	0	AAVNAAVQ	Sequence	0.054	27932.15	45.00	
44	HLA-B5801	HLLLKRERV	HLLLKRERV	0	0	0	0	0	HLLLKRERV	Sequence	0.054	27939.70	45.00	
48	HLA-B5801	KRERVDLSK	KRERVDLSK	0	0	0	0	0	KRERVDLSK	Sequence	0.053	28180.45	46.00	
16	HLA-B5801	KPCHQAEVK	KPCHQAEVK	0	0	0	0	0	KPCHQAEVK	Sequence	0.053	28313.71	47.00	
6	HLA-B5801	SPVIFQIALD	SVIFQIALD	0	0	0	1	1	SPVIFQIALD	Sequence	0.052	28491.64	48.00	
40	HLA-B5801	LKIKHLLLK	LKIKHLLLK	0	0	0	0	0	LKIKHLLLK	Sequence	0.051	28869.93	49.00	
11	HLA-B5801	QIALDKPCH	QIALDKPCH	0	0	0	0	0	QIALDKPCH	Sequence	0.051	28871.79	49.00	
7	HLA-B5801	PVIFQIALD	PVIFQIALD	0	0	0	0	0	PVIFQIALD	Sequence	0.051	28874.30	49.00	
47	HLA-B5801	LKRERVDLS	LKRERVDLS	0	0	0	0	0	LKRERVDLS	Sequence	0.051	28875.23	49.00	
45	HLA-B5801	LLLKRERVD	LLLKRERVD	0	0	0	0	0	LLLKRERVD	Sequence	0.051	28883.35	49.00	
45	HLA-B5801	LLLKRERVDL	LLKRERVDL	0	0	0	1	1	LLLKRERVDL	Sequence	0.050	28998.27	50.00	
19	HLA-B5801	HQAEVKHL	HQAEVK-HL	0	6	1	0	0	HQAEVKHL	Sequence	0.050	29139.19	50.00	
51	HLA-B5801	RVDLSKLQAA	RVDLSKLQA	0	0	0	8	1	RVDLSKLQAA	Sequence	0.050	29197.26	50.00	
17	HLA-B5801	PCHQAEVKH	PCHQAEVKH	0	0	0	0	0	PCHQAEVKH	Sequence	0.049	29500.18	55.00	

41	HLA-B5801	KIKHLLLKRE	KIKHLLLRE	0	0	0	7	1	KIKHLLLKRE	Sequence	0.049	29549.38	55.00
46	HLA-B5801	LLKRERVDLS	LLKRERVDL	0	0	0	0	0	LLKRERVDL	Sequence	0.048	29699.70	55.00
39	HLA-B5801	YLKIKHLLLK	YLKIKHLLLK	0	0	0	2	1	YLKIKHLLLK	Sequence	0.047	29962.44	55.00
28	HLA-B5801	HLLKQLKPS	HLLKQLKPS	0	0	0	0	0	HLLKQLKPS	Sequence	0.047	29972.16	55.00
34	HLA-B5801	KPSEKYLK	KPSE-KYLK	0	4	1	0	0	KPSEKYLK	Sequence	0.047	30020.85	55.00
35	HLA-B5801	PSEKYLKIK	PSEKYLKIK	0	0	0	0	0	PSEKYLKIK	Sequence	0.047	30051.07	55.00
29	HLA-B5801	LLKQLKPSE	LLKQLKPSE	0	0	0	0	0	LLKQLKPSE	Sequence	0.046	30315.92	55.00
36	HLA-B5801	SEKYLKIKH	SEKYLKIKH	0	0	0	0	0	SEKYLKIKH	Sequence	0.046	30380.29	55.00
39	HLA-B5801	YLKIKHLL	YL-KIKHLL	0	2	1	0	0	YLKIKHLL	Sequence	0.046	30490.60	60.00
25	HLA-B5801	HLHHLLKQLK	HLHHLLKQL	0	0	0	0	0	HLHHLLKQL	Sequence	0.046	30525.60	60.00
30	HLA-B5801	LKQLKPSEKY	LQLKPSEKY	0	0	0	1	1	LKQLKPSEKY	Sequence	0.044	30934.22	60.00
43	HLA-B5801	KHLLLKRERV	KLLLKRERV	0	0	0	1	1	KHLLLKRERV	Sequence	0.044	31146.13	60.00
10	HLA-B5801	FQIALDKPCH	FQIALDKPH	0	0	0	8	1	FQIALDKPCH	Sequence	0.044	31209.89	60.00
17	HLA-B5801	PCHQAEVKHL	PCHQAEVHL	0	0	0	7	1	PCHQAEVKHL	Sequence	0.043	31253.82	60.00
55	HLA-B5801	SKLQAAVNAA	SLQAAVNAA	0	0	0	1	1	SKLQAAVNAA	Sequence	0.043	31255.17	60.00
55	HLA-B5801	SKLQAAVNA	SKLQAAVNA	0	0	0	0	0	SKLQAAVNA	Sequence	0.043	31303.56	60.00
22	HLA-B5801	EVKHLHHLLK	EVKHLHHLL	0	0	0	0	0	EVKHLHHLL	Sequence	0.043	31542.59	65.00
37	HLA-B5801	EKYLKIKHL	EKYLKIKHL	0	0	0	0	0	EKYLKIKHL	Sequence	0.042	31835.05	65.00
35	HLA-B5801	PSEKYLKI	-PSEKYLKI	0	0	1	0	0	PSEKYLKI	Sequence	0.041	31919.90	65.00
26	HLA-B5801	LHHLLKQLK	LHHLLKQLK	0	0	0	0	0	LHHLLKQLK	Sequence	0.041	31939.93	65.00
36	HLA-B5801	SEKYLKIKHL	SEKYLKIHL	0	0	0	7	1	SEKYLKIKHL	Sequence	0.041	32077.43	65.00
32	HLA-B5801	QLKPSEKY	QLKPSE-KY	0	6	1	0	0	QLKPSEKY	Sequence	0.041	32112.14	65.00
33	HLA-B5801	LKPSEKYL	-LKPSEKYL	0	0	1	0	0	LKPSEKYL	Sequence	0.041	32255.61	65.00
50	HLA-B5801	ERVDLSKLQ	ERVDLSKLQ	0	0	0	0	0	ERVDLSKLQ	Sequence	0.040	32343.32	65.00
13	HLA-B5801	ALDKPCHQAE	ALDKPCHQE	0	0	0	8	1	ALDKPCHQAE	Sequence	0.040	32441.11	65.00
33	HLA-B5801	LKPSEKYLK	LKPSEKYLK	0	0	0	0	0	LKPSEKYLK	Sequence	0.040	32483.61	65.00
27	HLA-B5801	HHLLKQLKP	HHLLKQLKP	0	0	0	0	0	HHLLKQLKP	Sequence	0.039	32621.68	70.00
47	HLA-B5801	LKRERVDLSK	LKRERVDLK	0	0	0	8	1	LKRERVDLSK	Sequence	0.039	32683.51	70.00
50	HLA-B5801	ERVDLSKLQA	RVDLSKLQA	1	0	0	0	0	RVDLSKLQA	Sequence	0.039	32763.87	70.00
12	HLA-B5801	IALDKPCH	IALDKPCH-	0	8	1	0	0	IALDKPCH	Sequence	0.039	32865.43	70.00
38	HLA-B5801	KYLKIKHL	KYLKIK-HL	0	6	1	0	0	KYLKIKHL	Sequence	0.038	33175.89	70.00
49	HLA-B5801	RERVDLSKLQ	RRVDLSKLQ	0	0	0	1	1	RERVDLSKLQ	Sequence	0.038	33307.18	70.00
40	HLA-B5801	LKIKHLLLKR	KIKHLLLKR	1	0	0	0	0	KIKHLLLKR	Sequence	0.037	33381.86	70.00
22	HLA-B5801	EVKHLHHL	EV-KHLHHL	0	2	1	0	0	EVKHLHHL	Sequence	0.037	33462.86	70.00
30	HLA-B5801	LKQLKPSEK	LKQLKPSEK	0	0	0	0	0	LKQLKPSEK	Sequence	0.037	33571.65	70.00
44	HLA-B5801	HLLLKRERVD	HLLKRERVD	0	0	0	1	1	HLLLKRERVD	Sequence	0.037	33641.09	70.00
23	HLA-B5801	VKHLHHLLK	VKHLHHLLK	0	0	0	0	0	VKHLHHLLK	Sequence	0.036	33863.14	75.00
14	HLA-B5801	LDKPCHQAEV	LDKPCQAEV	0	0	0	5	1	LDKPCHQAEV	Sequence	0.036	33991.62	75.00
28	HLA-B5801	HLLKQLKPSE	HLKQLKPSE	0	0	0	1	1	HLLKQLKPSE	Sequence	0.036	34018.86	75.00
42	HLA-B5801	IKHLLLKRE	IKHLLLKRE	0	0	0	0	0	IKHLLLKRE	Sequence	0.035	34265.97	75.00
13	HLA-B5801	ALDKPCHQ	-ALDKPCHQ	0	0	1	0	0	ALDKPCHQ	Sequence	0.035	34298.25	75.00
7	HLA-B5801	PVIFQIALDK	PVIFIALDK	0	0	0	4	1	PVIFQIALDK	Sequence	0.034	34634.24	75.00
27	HLA-B5801	HHLLKQLKPS	HLLKQLKPS	0	0	0	1	1	HHLLKQLKPS	Sequence	0.034	34759.64	80.00
23	HLA-B5801	VKHLHHLL	-VKHLHHLL	0	0	1	0	0	VKHLHHLL	Sequence	0.034	34772.80	80.00
41	HLA-B5801	KIKHLLLK	KIKHLLLK-	0	8	1	0	0	KIKHLLLK	Sequence	0.034	34780.33	80.00
29	HLA-B5801	LLKQLKPSEK	LLKQKPSEK	0	0	0	4	1	LLKQLKPSEK	Sequence	0.033	34800.28	80.00
14	HLA-B5801	LDKPCHQAE	LDKPCHQAE	0	0	0	0	0	LDKPCHQAE	Sequence	0.033	34803.29	80.00
26	HLA-B5801	LHHLLKQLKP	LHHLLQLKP	0	0	0	5	1	LHHLLKQLKP	Sequence	0.033	34870.37	80.00
8	HLA-B5801	VIFQIALD	VIFQIA-LD	0	6	1	0	0	VIFQIALD	Sequence	0.033	34920.22	80.00
51	HLA-B5801	RVDLSKLQ	RVDLSKLQ-	0	8	1	0	0	RVDLSKLQ	Sequence	0.033	34956.88	80.00
56	HLA-B5801	KLQAAVNA	KL-QAAVNA	0	2	1	0	0	KLQAAVNA	Sequence	0.033	34978.07	80.00
23	HLA-B5801	VKHLHHLLKQ	VKHHHLLKQ	0	0	0	3	1	VKHLHHLLKQ	Sequence	0.033	35085.34	80.00
15	HLA-B5801	DKPCHQAEV	DKPCHQAEV	0	0	0	0	0	DKPCHQAEV	Sequence	0.033	35092.56	80.00
48	HLA-B5801	KRERVDLS	KR-ERVDLS	0	2	1	0	0	KRERVDLS	Sequence	0.033	35154.49	80.00
43	HLA-B5801	KHLLLKRER	KHLLLKRER	0	0	0	0	0	KHLLLKRER	Sequence	0.032	35263.46	80.00
53	HLA-B5801	DLSKLQAA	DLSKLQ-AA	0	6	1	0	0	DLSKLQAA	Sequence	0.031	35765.29	85.00
25	HLA-B5801	HLHHLLKQ	HLHHLL-KQ	0	6	1	0	0	HLHHLLKQ	Sequence	0.030	35972.14	85.00
40	HLA-B5801	LKIKHLLL	LKIKHL-LL	0	6	1	0	0	LKIKHLLL	Sequence	0.030	36015.36	85.00
47	HLA-B5801	LKRERVDL	-LKRERVDL	0	0	1	0	0	LKRERVDL	Sequence	0.030	36120.73	85.00
16	HLA-B5801	KPCHQAEV	KPCHQAEV-	0	8	1	0	0	KPCHQAEV	Sequence	0.030	36152.40	85.00
11	HLA-B5801	QIALDKPC	QIALDK-PC	0	6	1	0	0	QIALDKPC	Sequence	0.030	36218.56	85.00
15	HLA-B5801	DKPCHQAEVK	KPCHQAEVK	1	0	0	0	0	KPCHQAEVK	Sequence	0.030	36295.46	85.00
55	HLA-B5801	SKLQAAVN	-SKLQAAVN	0	0	1	0	0	SKLQAAVN	Sequence	0.029	36597.12	85.00
9	HLA-B5801	IFQIALDK	IFQIALDK-	0	8	1	0	0	IFQIALDK	Sequence	0.028	37096.25	90.00
46	HLA-B5801	LLKRERVD	LLKR-ERVD	0	4	1	0	0	LLKRERVD	Sequence	0.027	37154.91	90.00
57	HLA-B5801	LQAAVNAA	LQAAV-NAA	0	5	1	0	0	LQAAVNAA	Sequence	0.027	37431.71	90.00
14	HLA-B5801	LDKPCHQA	-LDKPCHQA	0	0	1	0	0	LDKPCHQA	Sequence	0.026	37544.05	90.00
18	HLA-B5801	CHQAEVKH	C-HQAEVKH	0	1	1	0	0	CHQAEVKH	Sequence	0.026	37568.04	90.00
21	HLA-B5801	AEVKHLHH	-AEVKHLHH	0	0	1	0	0	AEVKHLHH	Sequence	0.026	37668.16	95.00
31	HLA-B5801	KQLKPSEK	KQLKPSEK-	0	8	1	0	0	KQLKPSEK	Sequence	0.026	37700.80	95.00
7	HLA-B5801	PVIFQIAL	PV-IFQIAL	0	2	1	0	0	PVIFQIAL	Sequence	0.026	37800.44	95.00
10	HLA-B5801	FQIALDKP	FQI-ALDKP	0	3	1	0	0	FQIALDKP	Sequence	0.025	37947.97	95.00
26	HLA-B5801	LHHLLKQL	L-HHLLKQL	0	1	1	0	0	LHHLLKQL	Sequence	0.025	37975.07	95.00
24	HLA-B5801	KHLHHLLK	KHLHHLLK-	0	8	1	0	0	KHLHHLLK	Sequence	0.024	38388.18	95.00
6	HLA-B5801	SPVIFQIA	-SPVIFQIA	0	0	1	0	0	SPVIFQIA	Sequence	0.024	38423.52	95.00
42	HLA-B5801	IKHLLLKRER	IKHLLLRER	0	0	0	6	1	IKHLLLKRER	Sequence	0.024	38457.61	95.00
49	HLA-B5801	RERVDLSK	RE-RVDLSK	0	2	1	0	0	RERVDLSK	Sequence	0.024	38498.01	95.00
36	HLA-B5801	SEKYLKIK	-SEKYLKIK	0	0	1	0	0	SEKYLKIK	Sequence	0.024	38606.04	95.00
29	HLA-B5801	LLKQLKPS	LLKQLK-PS	0	6	1	0	0	LLKQLKPS	Sequence	0.024	38707.25	95.00
17	HLA-B5801	PCHQAEVK	PCHQAEVK-	0	8	1	0	0	PCHQAEVK	Sequence	0.023	38821.32	95.00
52	HLA-B5801	VDLSKLQAA	VDLSKLQAA	0	0	0	0	0	VDLSKLQAA	Sequence	0.023	39089.41	99.00
45	HLA-B5801	LLLKRERV	LL-LKRERV	0	2	1	0	0	LLLKRERV	Sequence	0.023	39091.10	99.00
50	HLA-B5801	ERVDLSKL	E-RVDLSKL	0	1	1	0	0	ERVDLSKL	Sequence	0.023	39119.88	99.00
28	HLA-B5801	HLLKQLKP	HL-LKQLKP	0	2	1	0	0	HLLKQLKP	Sequence	0.022	39353.79	99.00
52	HLA-B5801	VDLSKLQA	-VDLSKLQA	0	0	1	0	0	VDLSKLQA	Sequence	0.021	39848.24	99.00
15	HLA-B5801	DKPCHQAE	D-KPCHQAE	0	1	1	0	0	DKPCHQAE	Sequence	0.021	39978.67	99.00
30	HLA-B5801	LKQLKPSE	-LKQLKPSE	0	0	1	0	0	LKQLKPSE	Sequence	0.020	40221.63	99.00
27	HLA-B5801	HHLLKQLK	H-HLLKQLK	0	1	1	0	0	HHLLKQLK	Sequence	0.020	40270.40	99.00
37	HLA-B5801	EKYLKIKH	E-KYLKIKH	0	1	1	0	0	EKYLKIKH	Sequence	0.020	40445.07	99.00
42	HLA-B5801	IKHLLLKR	I-KHLLLKR	0	1	1	0	0	IKHLLLKR	Sequence	0.019	40541.90	99.00

43	HLA-B5801	KHLLLKRE	K-HLLLKRE	0	1	1	0	0	KHLLLKRE	Sequence	0.019	40761.36	99.00	
44	HLA-B5801	HLLLKRER	HL-LLKRER	0	2	1	0	0	HLLLKRER	Sequence	0.017	41555.35	99.00	

Protein Sequence. Allele HLA-B5801. Number of high binders 1. Number of weak binders 5. Number of peptides 177

Link to Allele Frequencies in Worldwide Populations HLA-B5801

Rank Threshold for Strong binding peptides 0.500
Rank Threshold for Weak binding peptides 2.000

----

pos	HLA	peptide	Core	Offset	I_pos	I_len	D_pos	D_len	iCore	Identity	1-log50k(aff)	Affinity(nM)	%Rank	BindLeve
57	HLA-B1501	LQAAVNAAV	LQAAVNAAV	0	0	0	0	0	LQAAVNAAV	Sequence	0.583	91.38	0.80	<= WB
57	HLA-B1501	LQAAVNAAVQ	LQAAVNAVQ	0	0	0	6	1	LQAAVNAAVQ	Sequence	0.544	138.81	1.00	<= WB
30	HLA-B1501	LKQLKPSEKY	LQLKPSEKY	0	0	0	1	1	LKQLKPSEKY	Sequence	0.521	177.64	1.20	<= WB
56	HLA-B1501	KLQAAVNAAV	KQAAVNAAV	0	0	0	1	1	KLQAAVNAAV	Sequence	0.474	297.63	1.60	<= WB
31	HLA-B1501	KQLKPSEKY	KQLKPSEKY	0	0	0	0	0	KQLKPSEKY	Sequence	0.471	307.42	1.60	<= WB
10	HLA-B1501	FQIALDKPCH	FQIALDKPH	0	0	0	8	1	FQIALDKPCH	Sequence	0.454	369.15	1.80	<= WB
1	HLA-B1501	VSGIISPVIF	VSGIISPVF	0	0	0	8	1	VSGIISPVIF	Sequence	0.406	617.20	2.50	
19	HLA-B1501	HQAEVKHLHH	HQAEVHLHH	0	0	0	5	1	HQAEVKHLHH	Sequence	0.388	748.38	3.00	
10	HLA-B1501	FQIALDKPC	FQIALDKPC	0	0	0	0	0	FQIALDKPC	Sequence	0.345	1201.55	4.00	
56	HLA-B1501	KLQAAVNAA	KLQAAVNAA	0	0	0	0	0	KLQAAVNAA	Sequence	0.338	1291.85	4.00	
31	HLA-B1501	KQLKPSEKYL	KQLKPSEYL	0	0	0	7	1	KQLKPSEKYL	Sequence	0.322	1536.84	4.50	
0	HLA-B1501	MVSGIISPV	MVSGIISPV	0	0	0	0	0	MVSGIISPV	Sequence	0.296	2031.61	5.00	
0	HLA-B1501	MVSGIISPVI	MVSGIISPI	0	0	0	8	1	MVSGIISPVI	Sequence	0.282	2356.02	5.50	
2	HLA-B1501	SGIISPVIF	SGIISPVIF	0	0	0	0	0	SGIISPVIF	Sequence	0.271	2672.52	6.00	
39	HLA-B1501	YLKIKHLLL	YLKIKHLLL	0	0	0	0	0	YLKIKHLLL	Sequence	0.265	2838.35	6.00	
9	HLA-B1501	IFQIALDKPC	IQIALDKPC	0	0	0	1	1	IFQIALDKPC	Sequence	0.262	2926.57	6.00	
57	HLA-B1501	LQAAVNAA	LQAAVNAA-	0	8	1	0	0	LQAAVNAA	Sequence	0.256	3119.59	6.50	
19	HLA-B1501	HQAEVKHLH	HQAEVKHLH	0	0	0	0	0	HQAEVKHLH	Sequence	0.239	3779.13	7.00	
55	HLA-B1501	SKLQAAVNAA	SLQAAVNAA	0	0	0	1	1	SKLQAAVNAA	Sequence	0.233	4018.67	7.50	
46	HLA-B1501	LLKRERVDL	LLKRERVDL	0	0	0	0	0	LLKRERVDL	Sequence	0.203	5552.18	9.00	
3	HLA-B1501	GIISPVIF	GIISPVI-F	0	7	1	0	0	GIISPVIF	Sequence	0.194	6125.14	10.00	
21	HLA-B1501	AEVKHLHHL	AEVKHLHHL	0	0	0	0	0	AEVKHLHHL	Sequence	0.192	6233.65	10.00	
32	HLA-B1501	QLKPSEKY	QLKP-SEKY	0	4	1	0	0	QLKPSEKY	Sequence	0.187	6630.13	11.00	
32	HLA-B1501	QLKPSEKYL	QLKPSEKYL	0	0	0	0	0	QLKPSEKYL	Sequence	0.182	7004.90	11.00	
54	HLA-B1501	LSKLQAAVNA	LSKLQAAVA	0	0	0	8	1	LSKLQAAVNA	Sequence	0.178	7317.26	11.00	
1	HLA-B1501	VSGIISPVI	VSGIISPVI	0	0	0	0	0	VSGIISPVI	Sequence	0.174	7570.95	12.00	
10	HLA-B1501	FQIALDKP	FQIALDKP-	0	8	1	0	0	FQIALDKP	Sequence	0.172	7769.28	12.00	
4	HLA-B1501	IISPVIFQI	IISPVIFQI	0	0	0	0	0	IISPVIFQI	Sequence	0.170	7957.55	12.00	
19	HLA-B1501	HQAEVKHL	HQAE-VKHL	0	4	1	0	0	HQAEVKHL	Sequence	0.168	8122.83	12.00	
5	HLA-B1501	ISPVIFQIAL	ISPVIFQAL	0	0	0	7	1	ISPVIFQIAL	Sequence	0.167	8201.61	12.00	
58	HLA-B1501	QAAVNAAV	-QAAVNAAV	0	0	1	0	0	QAAVNAAV	Sequence	0.163	8556.67	13.00	
3	HLA-B1501	GIISPVIFQI	GIISPVIFI	0	0	0	8	1	GIISPVIFQI	Sequence	0.157	9122.39	13.00	
25	HLA-B1501	HLHHLLKQL	HLHHLLKQL	0	0	0	0	0	HLHHLLKQL	Sequence	0.155	9349.22	14.00	
52	HLA-B1501	VDLSKLQAAV	VLSKLQAAV	0	0	0	1	1	VDLSKLQAAV	Sequence	0.154	9458.49	14.00	
4	HLA-B1501	IISPVIFQIA	IISPVFQIA	0	0	0	5	1	IISPVIFQIA	Sequence	0.152	9636.58	14.00	
45	HLA-B1501	LLLKRERVDL	LLLKRERVL	0	0	0	8	1	LLLKRERVDL	Sequence	0.152	9675.45	14.00	
36	HLA-B1501	SEKYLKIKHL	SEKYLKIHL	0	0	0	5	1	SEKYLKIKHL	Sequence	0.141	10861.24	15.00	
29	HLA-B1501	LLKQLKPSE	LLKQLKPSE	0	0	0	0	0	LLKQLKPSE	Sequence	0.136	11467.99	16.00	
21	HLA-B1501	AEVKHLHHLL	AEVKHLHHL	0	0	0	8	1	AEVKHLHHLL	Sequence	0.135	11567.05	16.00	
2	HLA-B1501	SGIISPVIFQ	SIISPVIFQ	0	0	0	1	1	SGIISPVIFQ	Sequence	0.127	12712.91	17.00	
38	HLA-B1501	KYLKIKHLLL	KLKIKHLLL	0	0	0	1	1	KYLKIKHLLL	Sequence	0.126	12724.33	17.00	
20	HLA-B1501	QAEVKHLHHL	QAVKHLHHL	0	0	0	2	1	QAEVKHLHHL	Sequence	0.122	13328.44	18.00	
49	HLA-B1501	RERVDLSKL	RERVDLSKL	0	0	0	0	0	RERVDLSKL	Sequence	0.119	13841.66	19.00	
3	HLA-B1501	GIISPVIFQ	GIISPVIFQ	0	0	0	0	0	GIISPVIFQ	Sequence	0.116	14232.26	19.00	
58	HLA-B1501	QAAVNAAVQ	QAAVNAAVQ	0	0	0	0	0	QAAVNAAVQ	Sequence	0.116	14287.96	19.00	
22	HLA-B1501	EVKHLHHLL	EVKHLHHLL	0	0	0	0	0	EVKHLHHLL	Sequence	0.114	14597.67	20.00	
54	HLA-B1501	LSKLQAAVN	LSKLQAAVN	0	0	0	0	0	LSKLQAAVN	Sequence	0.113	14731.91	20.00	
18	HLA-B1501	CHQAEVKHLH	CQAEVKHLH	0	0	0	1	1	CHQAEVKHLH	Sequence	0.111	15123.72	20.00	
24	HLA-B1501	KHLHHLLKQL	KLHHLLKQL	0	0	0	1	1	KHLHHLLKQL	Sequence	0.107	15694.68	21.00	
13	HLA-B1501	ALDKPCHQAE	ALKPCHQAE	0	0	0	2	1	ALDKPCHQAE	Sequence	0.106	15938.02	21.00	
5	HLA-B1501	ISPVIFQIA	ISPVIFQIA	0	0	0	0	0	ISPVIFQIA	Sequence	0.104	16275.93	22.00	
51	HLA-B1501	RVDLSKLQAA	RVLSKLQAA	0	0	0	2	1	RVDLSKLQAA	Sequence	0.100	16857.71	23.00	
56	HLA-B1501	KLQAAVNA	KLQAAVNA-	0	8	1	0	0	KLQAAVNA	Sequence	0.098	17244.76	23.00	
39	HLA-B1501	YLKIKHLLLK	YLKIKHLLL	0	0	0	0	0	YLKIKHLLL	Sequence	0.090	18784.21	25.00	
39	HLA-B1501	YLKIKHLL	YLKIKHL-L	0	7	1	0	0	YLKIKHLL	Sequence	0.089	19089.92	26.00	
0	HLA-B1501	MVSGIISP	MVSGIISP-	0	8	1	0	0	MVSGIISP	Sequence	0.085	19918.51	27.00	
54	HLA-B1501	LSKLQAAV	LSK-LQAAV	0	3	1	0	0	LSKLQAAV	Sequence	0.085	20017.02	27.00	
14	HLA-B1501	LDKPCHQAEV	LDKPCHQAV	0	0	0	8	1	LDKPCHQAEV	Sequence	0.081	20895.81	29.00	
12	HLA-B1501	IALDKPCHQ	IALDKPCHQ	0	0	0	0	0	IALDKPCHQ	Sequence	0.079	21369.32	29.00	
11	HLA-B1501	QIALDKPCH	QIALDKPCH	0	0	0	0	0	QIALDKPCH	Sequence	0.077	21719.44	30.00	
29	HLA-B1501	LLKQLKPSEK	LLKQLKPSK	0	0	0	8	1	LLKQLKPSEK	Sequence	0.075	22090.81	31.00	
53	HLA-B1501	DLSKLQAAV	DLSKLQAAV	0	0	0	0	0	DLSKLQAAV	Sequence	0.075	22149.70	31.00	
29	HLA-B1501	LLKQLKPS	LLKQLKPS-	0	8	1	0	0	LLKQLKPS	Sequence	0.074	22386.05	31.00	
28	HLA-B1501	HLLKQLKPS	HLLKQLKPS	0	0	0	0	0	HLLKQLKPS	Sequence	0.073	22736.83	32.00	
14	HLA-B1501	LDKPCHQAE	LDKPCHQAE	0	0	0	0	0	LDKPCHQAE	Sequence	0.071	23315.80	33.00	
6	HLA-B1501	SPVIFQIAL	SPVIFQIAL	0	0	0	0	0	SPVIFQIAL	Sequence	0.070	23536.82	33.00	
11	HLA-B1501	QIALDKPCHQ	QIADKPCHQ	0	0	0	3	1	QIALDKPCHQ	Sequence	0.069	23639.93	33.00	
49	HLA-B1501	RERVDLSKLQ	RERDLSKLQ	0	0	0	3	1	RERVDLSKLQ	Sequence	0.069	23660.66	33.00	
11	HLA-B1501	QIALDKPC	-QIALDKPC	0	0	1	0	0	QIALDKPC	Sequence	0.066	24413.79	35.00	
28	HLA-B1501	HLLKQLKPSE	HLKQLKPSE	0	0	0	1	1	HLLKQLKPSE	Sequence	0.065	24626.82	35.00	
44	HLA-B1501	HLLLKRERV	HLLLKRERV	0	0	0	0	0	HLLLKRERV	Sequence	0.063	25392.29	37.00	
1	HLA-B1501	VSGIISPV	-VSGIISPV	0	0	1	0	0	VSGIISPV	Sequence	0.061	25804.12	38.00	
36	HLA-B1501	SEKYLKIKH	SEKYLKIKH	0	0	0	0	0	SEKYLKIKH	Sequence	0.061	25822.56	38.00	
46	HLA-B1501	LLKRERVDLS	LLKRERVDL	0	0	0	0	0	LLKRERVDL	Sequence	0.061	25958.14	38.00	
2	HLA-B1501	SGIISPVI	SGI-ISPVI	0	3	1	0	0	SGIISPVI	Sequence	0.060	26013.81	38.00	
51	HLA-B1501	RVDLSKLQA	RVDLSKLQA	0	0	0	0	0	RVDLSKLQA	Sequence	0.058	26584.89	39.00	
37	HLA-B1501	EKYLKIKHL	EKYLKIKHL	0	0	0	0	0	EKYLKIKHL	Sequence	0.058	26594.66	39.00	

8	HLA-B1501	VIFQIALDK	VIFQIALDK	0	0	0	0	0	VIFQIALDK	Sequence	0.058	26744.42	40.00
32	HLA-B1501	QLKPSEKYLK	QLKPSEKYL	0	0	0	0	0	QLKPSEKYL	Sequence	0.056	27348.41	41.00
38	HLA-B1501	KYLKIKHLL	KYLKIKHLL	0	0	0	0	0	KYLKIKHLL	Sequence	0.055	27662.36	42.00
48	HLA-B1501	KRERVDLSKL	KERVDLSKL	0	0	0	1	1	KRERVDLSKL	Sequence	0.054	27818.45	42.00
18	HLA-B1501	CHQAEVKHL	CHQAEVKHL	0	0	0	0	0	CHQAEVKHL	Sequence	0.050	29000.47	45.00
5	HLA-B1501	ISPVIFQI	IS-PVIFQI	0	2	1	0	0	ISPVIFQI	Sequence	0.050	29009.88	45.00
8	HLA-B1501	VIFQIALDKP	VIFQIALDP	0	0	0	8	1	VIFQIALDKP	Sequence	0.049	29296.92	46.00
22	HLA-B1501	EVKHLHHL	EVK-HLHHL	0	3	1	0	0	EVKHLHHL	Sequence	0.049	29495.39	47.00
12	HLA-B1501	IALDKPCHQA	IALDKPCHA	0	0	0	8	1	IALDKPCHQA	Sequence	0.048	29594.18	47.00
25	HLA-B1501	HLHHLLKQLK	HLHHLLKQL	0	0	0	0	0	HLHHLLKQL	Sequence	0.048	29665.02	47.00
6	HLA-B1501	SPVIFQIALD	SVIFQIALD	0	0	0	1	1	SPVIFQIALD	Sequence	0.048	29704.86	47.00
52	HLA-B1501	VDLSKLQAA	VDLSKLQAA	0	0	0	0	0	VDLSKLQAA	Sequence	0.048	29887.96	48.00
20	HLA-B1501	QAEVKHLH	-QAEVKHLH	0	0	1	0	0	QAEVKHLH	Sequence	0.047	30122.36	48.00
38	HLA-B1501	KYLKIKHL	K-YLKIKHL	0	1	1	0	0	KYLKIKHL	Sequence	0.046	30387.52	49.00
20	HLA-B1501	QAEVKHLHH	QAEVKHLHH	0	0	0	0	0	QAEVKHLHH	Sequence	0.045	30668.26	50.00
31	HLA-B1501	KQLKPSEK	KQLKPSEK-	0	8	1	0	0	KQLKPSEK	Sequence	0.044	30909.12	55.00
55	HLA-B1501	SKLQAAVNA	SKLQAAVNA	0	0	0	0	0	SKLQAAVNA	Sequence	0.044	30911.81	55.00
13	HLA-B1501	ALDKPCHQA	ALDKPCHQA	0	0	0	0	0	ALDKPCHQA	Sequence	0.044	30925.51	55.00
45	HLA-B1501	LLLKRERVD	LLLKRERVD	0	0	0	0	0	LLLKRERVD	Sequence	0.043	31357.13	55.00
4	HLA-B1501	IISPVIFQ	IISPVIFQ-	0	8	1	0	0	IISPVIFQ	Sequence	0.043	31357.13	55.00
40	HLA-B1501	LKIKHLLL	-LKIKHLLL	0	0	1	0	0	LKIKHLLL	Sequence	0.043	31377.16	55.00
23	HLA-B1501	VKHLHHLL	-VKHLHHLL	0	0	1	0	0	VKHLHHLL	Sequence	0.043	31404.67	55.00
27	HLA-B1501	HHLLKQLKPS	HLLKQLKPS	0	0	0	1	1	HHLLKQLKPS	Sequence	0.042	31908.84	55.00
41	HLA-B1501	KIKHLLLKR	KIKHLLLKR	0	0	0	0	0	KIKHLLLKR	Sequence	0.041	31956.87	55.00
59	HLA-B1501	AAVNAAVQ	AAVNA-AVQ	0	5	1	0	0	AAVNAAVQ	Sequence	0.041	32199.47	55.00
7	HLA-B1501	PVIFQIAL	PVIFQI-AL	0	6	1	0	0	PVIFQIAL	Sequence	0.040	32365.40	55.00
33	HLA-B1501	LKPSEKYL	-LKPSEKYL	0	0	1	0	0	LKPSEKYL	Sequence	0.039	32611.79	60.00
53	HLA-B1501	DLSKLQAAVN	LSKLQAAVN	1	0	0	0	0	LSKLQAAVN	Sequence	0.039	32637.57	60.00
14	HLA-B1501	LDKPCHQA	LDKPCHQA-	0	8	1	0	0	LDKPCHQA	Sequence	0.039	32738.38	60.00
37	HLA-B1501	EKYLKIKHLL	EKYLKIKHL	0	0	0	8	1	EKYLKIKHLL	Sequence	0.038	33160.83	60.00
8	HLA-B1501	VIFQIALD	VIFQIALD-	0	8	1	0	0	VIFQIALD	Sequence	0.037	33503.80	60.00
7	HLA-B1501	PVIFQIALD	PVIFQIALD	0	0	0	0	0	PVIFQIALD	Sequence	0.037	33505.25	60.00
43	HLA-B1501	KHLLLKRERV	KLLLKRERV	0	0	0	1	1	KHLLLKRERV	Sequence	0.037	33542.24	60.00
22	HLA-B1501	EVKHLHHLLK	EVKHLHHLL	0	0	0	0	0	EVKHLHHLL	Sequence	0.036	33947.14	60.00
34	HLA-B1501	KPSEKYLKI	KPSEKYLKI	0	0	0	0	0	KPSEKYLKI	Sequence	0.035	34252.27	65.00
47	HLA-B1501	LKRERVDLS	LKRERVDLS	0	0	0	0	0	LKRERVDLS	Sequence	0.035	34296.39	65.00
41	HLA-B1501	KIKHLLLKRE	KIKHLLLRE	0	0	0	7	1	KIKHLLLKRE	Sequence	0.034	34453.72	65.00
40	HLA-B1501	LKIKHLLLK	LKIKHLLLK	0	0	0	0	0	LKIKHLLLK	Sequence	0.033	34997.75	65.00
23	HLA-B1501	VKHLHHLLK	VKHLHHLLK	0	0	0	0	0	VKHLHHLLK	Sequence	0.033	35033.39	65.00
47	HLA-B1501	LKRERVDLSK	LKRERVLSK	0	0	0	6	1	LKRERVDLSK	Sequence	0.033	35147.64	65.00
47	HLA-B1501	LKRERVDL	-LKRERVDL	0	0	1	0	0	LKRERVDL	Sequence	0.033	35158.30	65.00
49	HLA-B1501	RERVDLSK	RER-VDLSK	0	3	1	0	0	RERVDLSK	Sequence	0.032	35452.06	70.00
13	HLA-B1501	ALDKPCHQ	AL-DKPCHQ	0	2	1	0	0	ALDKPCHQ	Sequence	0.031	35690.68	70.00
9	HLA-B1501	IFQIALDKP	IFQIALDKP	0	0	0	0	0	IFQIALDKP	Sequence	0.031	35710.37	70.00
26	HLA-B1501	LHHLLKQL	-LHHLLKQL	0	0	1	0	0	LHHLLKQL	Sequence	0.031	35755.24	70.00
28	HLA-B1501	HLLKQLKP	HLLKQLKP-	0	8	1	0	0	HLLKQLKP	Sequence	0.031	35900.59	70.00
45	HLA-B1501	LLLKRERV	LLLKRERV-	0	8	1	0	0	LLLKRERV	Sequence	0.030	35956.57	70.00
26	HLA-B1501	LHHLLKQLK	LHHLLKQLK	0	0	0	0	0	LHHLLKQLK	Sequence	0.030	36038.36	70.00
21	HLA-B1501	AEVKHLHH	AEVKH-LHH	0	5	1	0	0	AEVKHLHH	Sequence	0.030	36069.57	70.00
46	HLA-B1501	LLKRERVD	LLKRERVD-	0	8	1	0	0	LLKRERVD	Sequence	0.030	36265.23	70.00
6	HLA-B1501	SPVIFQIA	-SPVIFQIA	0	0	1	0	0	SPVIFQIA	Sequence	0.029	36474.57	70.00
7	HLA-B1501	PVIFQIALDK	PVIFQIALK	0	0	0	8	1	PVIFQIALDK	Sequence	0.029	36568.62	70.00
40	HLA-B1501	LKIKHLLLKR	LIKHLLLKR	0	0	0	1	1	LKIKHLLLKR	Sequence	0.029	36590.39	75.00
44	HLA-B1501	HLLLKRERVD	HLLLKRERV	0	0	0	0	0	HLLLKRERV	Sequence	0.028	36806.41	75.00
51	HLA-B1501	RVDLSKLQ	RV-DLSKLQ	0	2	1	0	0	RVDLSKLQ	Sequence	0.028	37047.34	75.00
23	HLA-B1501	VKHLHHLLKQ	VKHLHHLLQ	0	0	0	8	1	VKHLHHLLKQ	Sequence	0.027	37168.98	75.00
25	HLA-B1501	HLHHLLKQ	HLHHLLKQ-	0	8	1	0	0	HLHHLLKQ	Sequence	0.027	37270.87	75.00
16	HLA-B1501	KPCHQAEVK	KPCHQAEVK	0	0	0	0	0	KPCHQAEVK	Sequence	0.027	37330.99	75.00
41	HLA-B1501	KIKHLLLK	KIKHLLLK-	0	8	1	0	0	KIKHLLLK	Sequence	0.027	37377.07	75.00
30	HLA-B1501	LKQLKPSEK	LKQLKPSEK	0	0	0	0	0	LKQLKPSEK	Sequence	0.026	37702.43	75.00
16	HLA-B1501	KPCHQAEVKH	KPCHQAEVH	0	0	0	8	1	KPCHQAEVKH	Sequence	0.025	38035.54	80.00
42	HLA-B1501	IKHLLLKRE	IKHLLLKRE	0	0	0	0	0	IKHLLLKRE	Sequence	0.025	38282.02	80.00
33	HLA-B1501	LKPSEKYLKI	LKSEKYLKI	0	0	0	2	1	LKPSEKYLKI	Sequence	0.025	38297.33	80.00
17	HLA-B1501	PCHQAEVKHL	PCHQAEVHL	0	0	0	7	1	PCHQAEVKHL	Sequence	0.024	38544.27	80.00
35	HLA-B1501	PSEKYLKIKH	SEKYLKIKH	1	0	0	0	0	SEKYLKIKH	Sequence	0.024	38634.88	80.00
12	HLA-B1501	IALDKPCH	IALDKPCH-	0	8	1	0	0	IALDKPCH	Sequence	0.024	38737.84	80.00
30	HLA-B1501	LKQLKPSE	-LKQLKPSE	0	0	1	0	0	LKQLKPSE	Sequence	0.023	38937.87	80.00
16	HLA-B1501	KPCHQAEV	K-PCHQAEV	0	1	1	0	0	KPCHQAEV	Sequence	0.023	39054.73	85.00
24	HLA-B1501	KHLHHLLKQ	KHLHHLLKQ	0	0	0	0	0	KHLHHLLKQ	Sequence	0.023	39064.89	85.00
50	HLA-B1501	ERVDLSKLQ	ERVDLSKLQ	0	0	0	0	0	ERVDLSKLQ	Sequence	0.023	39154.18	85.00
43	HLA-B1501	KHLLLKRER	KHLLLKRER	0	0	0	0	0	KHLLLKRER	Sequence	0.023	39179.18	85.00
48	HLA-B1501	KRERVDLSK	KRERVDLSK	0	0	0	0	0	KRERVDLSK	Sequence	0.022	39245.36	85.00
50	HLA-B1501	ERVDLSKLQA	RVDLSKLQA	1	0	0	0	0	RVDLSKLQA	Sequence	0.022	39321.44	85.00
55	HLA-B1501	SKLQAAVN	-SKLQAAVN	0	0	1	0	0	SKLQAAVN	Sequence	0.022	39333.77	85.00
50	HLA-B1501	ERVDLSKL	-ERVDLSKL	0	0	1	0	0	ERVDLSKL	Sequence	0.022	39435.18	85.00
15	HLA-B1501	DKPCHQAEV	DKPCHQAEV	0	0	0	0	0	DKPCHQAEV	Sequence	0.022	39442.46	85.00
26	HLA-B1501	LHHLLKQLKP	LHHLLKQLP	0	0	0	8	1	LHHLLKQLKP	Sequence	0.021	39670.14	85.00
27	HLA-B1501	HHLLKQLKP	HHLLKQLKP	0	0	0	0	0	HHLLKQLKP	Sequence	0.020	40249.91	90.00
53	HLA-B1501	DLSKLQAA	DLSKLQAA-	0	8	1	0	0	DLSKLQAA	Sequence	0.020	40409.19	90.00
9	HLA-B1501	IFQIALDK	-IFQIALDK	0	0	1	0	0	IFQIALDK	Sequence	0.020	40441.99	90.00
36	HLA-B1501	SEKYLKIK	SEKYLKIK-	0	8	1	0	0	SEKYLKIK	Sequence	0.020	40470.90	90.00
24	HLA-B1501	KHLHHLLK	K-HLHHLLK	0	1	1	0	0	KHLHHLLK	Sequence	0.019	40505.94	90.00
44	HLA-B1501	HLLLKRER	HLLLKRER-	0	8	1	0	0	HLLLKRER	Sequence	0.019	40800.20	90.00
42	HLA-B1501	IKHLLLKRER	IKHLLLRER	0	0	0	6	1	IKHLLLKRER	Sequence	0.018	41094.38	90.00
37	HLA-B1501	EKYLKIKH	-EKYLKIKH	0	0	1	0	0	EKYLKIKH	Sequence	0.017	41417.56	95.00
17	HLA-B1501	PCHQAEVKH	PCHQAEVKH	0	0	0	0	0	PCHQAEVKH	Sequence	0.017	41616.10	95.00
34	HLA-B1501	KPSEKYLKIK	KSEKYLKIK	0	0	0	1	1	KPSEKYLKIK	Sequence	0.017	41707.15	95.00
15	HLA-B1501	DKPCHQAE	-DKPCHQAE	0	0	1	0	0	DKPCHQAE	Sequence	0.017	41729.27	95.00
33	HLA-B1501	LKPSEKYLK	LKPSEKYLK	0	0	0	0	0	LKPSEKYLK	Sequence	0.016	41842.29	95.00
48	HLA-B1501	KRERVDLS	K-RERVDLS	0	1	1	0	0	KRERVDLS	Sequence	0.016	41846.82	95.00

42	HLA-B1501	IKHLLLKR	IKHLLLKR-	0	8	1	0	0	IKHLLLKR	Sequence	0.016	42007.87	95.00
52	HLA-B1501	VDLSKLQA	VDLSKLQA-	0	8	1	0	0	VDLSKLQA	Sequence	0.016	42128.49	95.00
27	HLA-B1501	HHLLKQLK	H-HLLKQLK	0	1	1	0	0	HHLLKQLK	Sequence	0.016	42211.54	95.00
43	HLA-B1501	KHLLLKRE	KHLLLKRE-	0	8	1	0	0	KHLLLKRE	Sequence	0.015	42555.02	95.00
18	HLA-B1501	CHQAEVKH	C-HQAEVKH	0	1	1	0	0	CHQAEVKH	Sequence	0.014	43154.99	99.00
35	HLA-B1501	PSEKYLKIK	PSEKYLKIK	0	0	0	0	0	PSEKYLKIK	Sequence	0.013	43361.40	99.00
15	HLA-B1501	DKPCHQAEVK	KPCHQAEVK	1	0	0	0	0	KPCHQAEVK	Sequence	0.012	43866.81	99.00
34	HLA-B1501	KPSEKYLK	KPSEKYLK-	0	8	1	0	0	KPSEKYLK	Sequence	0.012	44012.27	99.00
35	HLA-B1501	PSEKYLKI	-PSEKYLKI	0	0	1	0	0	PSEKYLKI	Sequence	0.011	44403.05	99.00
17	HLA-B1501	PCHQAEVK	-PCHQAEVK	0	0	1	0	0	PCHQAEVK	Sequence	0.008	45625.46	99.00

Protein Sequence. Allele HLA-B1501. Number of high binders 0. Number of weak binders 6. Number of peptides 177

Link to Allele Frequencies in Worldwide Populations HLA-B1501

Go **back**.
