# Supplementary material for: Identification, design, and in vivo proof of concept of a shared APC neoantigen delivered via a self-amplifying RNA containing virus-like nanoparticle for cancer vaccination
Source: Front Immunol. 2026 Jun 4;17:1810178. doi: 10.3389/fimmu.2026.1810178 (PMC13275407; doi:10.3389/fimmu.2026.1810178)
Supplement: Supplementary file 2 [file DataSheet2.pdf]

jobid=6679A0A6001D2614CB83D847&wait=20 Server Output - DTU Health Tech

---

```
# NetMHC version 4.0

# Input is in FSA format

# Peptide length 8,9,10
# Rank Threshold for Strong binding peptides   0.500
# Rank Threshold for Weak binding peptides   2.000
```

pos	HLA	peptide	Core	Offset	I_pos	I_len	D_pos	D_len	iCore	Identity	1-log50k(aff)	Affinity(nM)	%Rank	BindLeve
17	HLA-A0101	QMDFLVHPA	QMDFLVHPA	0	0	0	0	0	QMDFLVHPA	Sequence	0.360	1017.10	0.60	<= WB
16	HLA-A0101	LQMDFLVHPA	LMDFLVHPA	0	0	0	1	1	LQMDFLVHPA	Sequence	0.340	1265.63	0.70	<= WB
17	HLA-A0101	QMDFLVHPAA	QMDFLVHPA	0	0	0	8	1	QMDFLVHPAA	Sequence	0.221	4598.66	1.50	<= WB
1	HLA-A0101	VLPDADIYY	VLPDADIYY	0	0	0	0	0	VLPDADIYY	Sequence	0.218	4731.29	1.50	<= WB
0	HLA-A0101	QVLPDADIYY	VLPDADIYY	1	0	0	0	0	VLPDADIYY	Sequence	0.162	8627.61	2.50	
18	HLA-A0101	MDFLVHPA	-MDFLVHPA	0	0	1	0	0	MDFLVHPA	Sequence	0.134	11711.63	3.50	
2	HLA-A0101	LPDADIYY	LPDA-DIYY	0	4	1	0	0	LPDADIYY	Sequence	0.106	15903.06	6.00	
0	HLA-A0101	QVLPDADIY	QVLPDADIY	0	0	0	0	0	QVLPDADIY	Sequence	0.105	16114.20	6.00	
1	HLA-A0101	VLPDADIYYI	VLDADIYYI	0	0	0	2	1	VLPDADIYYI	Sequence	0.092	18386.28	8.00	
14	HLA-A0101	KVLQMDFLV	KVLQMDFLV	0	0	0	0	0	KVLQMDFLV	Sequence	0.090	18934.61	8.50	
25	HLA-A0101	AALSLDEPF	AALSLDEPF	0	0	0	0	0	AALSLDEPF	Sequence	0.088	19388.20	9.00	
21	HLA-A0101	LVHPAALSL	LVHPAALSL	0	0	0	0	0	LVHPAALSL	Sequence	0.085	19981.54	9.50	
4	HLA-A0101	DADIYYILP	DADIYYILP	0	0	0	0	0	DADIYYILP	Sequence	0.083	20436.99	10.00	
17	HLA-A0101	QMDFLVHP	QMDFLVHP-	0	8	1	0	0	QMDFLVHP	Sequence	0.082	20603.95	11.00	
2	HLA-A0101	LPDADIYYI	LPDADIYYI	0	0	0	0	0	LPDADIYYI	Sequence	0.080	21010.29	11.00	
15	HLA-A0101	VLQMDFLVH	VLQMDFLVH	0	0	0	0	0	VLQMDFLVH	Sequence	0.079	21355.68	12.00	
3	HLA-A0101	PDADIYYIL	PDADIYYIL	0	0	0	0	0	PDADIYYIL	Sequence	0.078	21592.44	12.00	
12	HLA-A0101	PRKVLQMDF	PRKVLQMDF	0	0	0	0	0	PRKVLQMDF	Sequence	0.070	23538.86	16.00	
1	HLA-A0101	VLPDADIY	VL-PDADIY	0	2	1	0	0	VLPDADIY	Sequence	0.069	23652.21	16.00	
19	HLA-A0101	DFLVHPAAL	DFLVHPAAL	0	0	0	0	0	DFLVHPAAL	Sequence	0.068	23867.38	16.00	
4	HLA-A0101	DADIYYILPR	DADIYYLPR	0	0	0	6	1	DADIYYILPR	Sequence	0.068	23977.38	17.00	
18	HLA-A0101	MDFLVHPAA	MDFLVHPAA	0	0	0	0	0	MDFLVHPAA	Sequence	0.067	24190.01	17.00	
6	HLA-A0101	DIYYILPRK	DIYYILPRK	0	0	0	0	0	DIYYILPRK	Sequence	0.065	24754.78	18.00	
27	HLA-A0101	LSLDEPFIQ	LSLDEPFIQ	0	0	0	0	0	LSLDEPFIQ	Sequence	0.065	24782.93	19.00	
24	HLA-A0101	PAALSLDEPF	PAASLDEPF	0	0	0	3	1	PAALSLDEPF	Sequence	0.065	24820.50	19.00	
13	HLA-A0101	RKVLQMDFL	RKVLQMDFL	0	0	0	0	0	RKVLQMDFL	Sequence	0.064	25151.92	19.00	
20	HLA-A0101	FLVHPAALSL	LVHPAALSL	1	0	0	0	0	LVHPAALSL	Sequence	0.063	25319.85	20.00	
9	HLA-A0101	YILPRKVLQ	YILPRKVLQ	0	0	0	0	0	YILPRKVLQ	Sequence	0.063	25387.60	20.00	
8	HLA-A0101	YYILPRKVL	YYILPRKVL	0	0	0	0	0	YYILPRKVL	Sequence	0.062	25694.07	21.00	
7	HLA-A0101	IYYILPRKV	IYYILPRKV	0	0	0	0	0	IYYILPRKV	Sequence	0.061	25729.12	21.00	
4	HLA-A0101	DADIYYIL	DADIYYIL-	0	8	1	0	0	DADIYYIL	Sequence	0.061	25840.72	21.00	
5	HLA-A0101	ADIYYILPR	ADIYYILPR	0	0	0	0	0	ADIYYILPR	Sequence	0.061	25967.97	22.00	
10	HLA-A0101	ILPRKVLQM	ILPRKVLQM	0	0	0	0	0	ILPRKVLQM	Sequence	0.060	26203.93	22.00	
2	HLA-A0101	LPDADIYYIL	LPDAIYYIL	0	0	0	4	1	LPDADIYYIL	Sequence	0.058	26556.43	23.00	
26	HLA-A0101	ALSLDEPFI	ALSLDEPFI	0	0	0	0	0	ALSLDEPFI	Sequence	0.057	26906.09	25.00	
15	HLA-A0101	VLQMDFLV	VL-QMDFLV	0	2	1	0	0	VLQMDFLV	Sequence	0.057	26951.53	25.00	
9	HLA-A0101	YILPRKVLQM	YILPRVLQM	0	0	0	5	1	YILPRKVLQM	Sequence	0.056	27168.79	25.00	
13	HLA-A0101	RKVLQMDFLV	KVLQMDFLV	1	0	0	0	0	KVLQMDFLV	Sequence	0.056	27306.11	26.00	
26	HLA-A0101	ALSLDEPF	AL-SLDEPF	0	2	1	0	0	ALSLDEPF	Sequence	0.056	27331.54	26.00	
20	HLA-A0101	FLVHPAALS	FLVHPAALS	0	0	0	0	0	FLVHPAALS	Sequence	0.055	27589.13	27.00	
16	HLA-A0101	LQMDFLVHP	LQMDFLVHP	0	0	0	0	0	LQMDFLVHP	Sequence	0.053	28113.45	29.00	
18	HLA-A0101	MDFLVHPAAL	DFLVHPAAL	1	0	0	0	0	DFLVHPAAL	Sequence	0.052	28529.59	30.00	
20	HLA-A0101	FLVHPAAL	FL-VHPAAL	0	2	1	0	0	FLVHPAAL	Sequence	0.052	28570.36	30.00	
11	HLA-A0101	LPRKVLQMDF	LRKVLQMDF	0	0	0	1	1	LPRKVLQMDF	Sequence	0.049	29369.92	34.00	
23	HLA-A0101	HPAALSLDE	HPAALSLDE	0	0	0	0	0	HPAALSLDE	Sequence	0.046	30303.13	38.00	
7	HLA-A0101	IYYILPRKVL	YYILPRKVL	1	0	0	0	0	YYILPRKVL	Sequence	0.045	30615.90	39.00	
12	HLA-A0101	PRKVLQMDFL	PRKVLQMDF	0	0	0	0	0	PRKVLQMDF	Sequence	0.044	30996.87	41.00	
6	HLA-A0101	DIYYILPRKV	DIYYILPRV	0	0	0	8	1	DIYYILPRKV	Sequence	0.044	31071.42	41.00	
14	HLA-A0101	KVLQMDFLVH	KVLQMDFLV	0	0	0	0	0	KVLQMDFLV	Sequence	0.044	31073.42	41.00	
25	HLA-A0101	AALSLDEPFI	AALSLDEPF	0	0	0	0	0	AALSLDEPF	Sequence	0.044	31124.91	41.00	
14	HLA-A0101	KVLQMDFL	KV-LQMDFL	0	2	1	0	0	KVLQMDFL	Sequence	0.043	31405.34	43.00	
9	HLA-A0101	YILPRKVL	YILPRK-VL	0	6	1	0	0	YILPRKVL	Sequence	0.042	31642.73	44.00	
6	HLA-A0101	DIYYILPR	DIY-YILPR	0	3	1	0	0	DIYYILPR	Sequence	0.041	31966.56	46.00	
13	HLA-A0101	RKVLQMDF	-RKVLQMDF	0	0	1	0	0	RKVLQMDF	Sequence	0.041	32028.17	46.00	
3	HLA-A0101	PDADIYYILP	PDADIYYIL	0	0	0	0	0	PDADIYYIL	Sequence	0.041	32127.10	46.00	
24	HLA-A0101	PAALSLDEP	PAALSLDEP	0	0	0	0	0	PAALSLDEP	Sequence	0.041	32143.78	46.00	
21	HLA-A0101	LVHPAALSLD	LVHPAALSL	0	0	0	0	0	LVHPAALSL	Sequence	0.040	32477.28	48.00	
19	HLA-A0101	DFLVHPAALS	DFLVHPAAL	0	0	0	0	0	DFLVHPAAL	Sequence	0.039	32705.44	49.00	
8	HLA-A0101	YYILPRKV	Y-YILPRKV	0	1	1	0	0	YYILPRKV	Sequence	0.039	32711.47	49.00	
11	HLA-A0101	LPRKVLQMD	LPRKVLQMD	0	0	0	0	0	LPRKVLQMD	Sequence	0.038	33008.32	55.00	
27	HLA-A0101	LSLDEPFI	LSLDEP-FI	0	6	1	0	0	LSLDEPFI	Sequence	0.038	33047.64	55.00	
22	HLA-A0101	VHPAALSLD	VHPAALSLD	0	0	0	0	0	VHPAALSLD	Sequence	0.037	33561.85	55.00	
5	HLA-A0101	ADIYYILPRK	DIYYILPRK	1	0	0	0	0	DIYYILPRK	Sequence	0.036	33727.84	55.00	
3	HLA-A0101	PDADIYYI	PDADI-YYI	0	5	1	0	0	PDADIYYI	Sequence	0.036	33736.60	55.00	
22	HLA-A0101	VHPAALSL	-VHPAALSL	0	0	1	0	0	VHPAALSL	Sequence	0.035	34144.95	60.00	
16	HLA-A0101	LQMDFLVH	LQMD-FLVH	0	4	1	0	0	LQMDFLVH	Sequence	0.035	34192.66	60.00	
8	HLA-A0101	YYILPRKVLQ	YILPRKVLQ	1	0	0	0	0	YILPRKVLQ	Sequence	0.035	34213.75	60.00	
19	HLA-A0101	DFLVHPAA	DFLVHPA-A	0	7	1	0	0	DFLVHPAA	Sequence	0.033	35031.11	65.00	
26	HLA-A0101	ALSLDEPFIQ	LSLDEPFIQ	1	0	0	0	0	LSLDEPFIQ	Sequence	0.033	35120.27	65.00	
11	HLA-A0101	LPRKVLQM	LPR-KVLQM	0	3	1	0	0	LPRKVLQM	Sequence	0.032	35414.11	65.00	
0	HLA-A0101	QVLPDADI	QVLP-DADI	0	4	1	0	0	QVLPDADI	Sequence	0.030	35959.68	70.00	
5	HLA-A0101	ADIYYILP	ADIYYILP-	0	8	1	0	0	ADIYYILP	Sequence	0.030	36031.74	70.00	
7	HLA-A0101	IYYILPRK	-IYYILPRK	0	0	1	0	0	IYYILPRK	Sequence	0.029	36342.21	70.00	
15	HLA-A0101	VLQMDFLVHP	LQMDFLVHP	1	0	0	0	0	LQMDFLVHP	Sequence	0.029	36590.80	75.00	
28	HLA-A0101	SLDEPFIQ	SLDE-PFIQ	0	4	1	0	0	SLDEPFIQ	Sequence	0.028	37028.08	75.00	

21	HLA-A0101	LVHPAALS	LVHPAALS-	0	8	1	0	0	LVHPAALS	Sequence	0.027	37349.58	80.00
23	HLA-A0101	HPAALSLD	HPAALSLD-	0	8	1	0	0	HPAALSLD	Sequence	0.024	38359.12	85.00
25	HLA-A0101	AALSLDEP	AALSLDEP-	0	8	1	0	0	AALSLDEP	Sequence	0.024	38754.61	85.00
23	HLA-A0101	HPAALSLDEP	PAALSLDEP	1	0	0	0	0	PAALSLDEP	Sequence	0.022	39622.09	90.00
22	HLA-A0101	VHPAALSLDE	HPAALSLDE	1	0	0	0	0	HPAALSLDE	Sequence	0.021	39635.82	90.00
10	HLA-A0101	ILPRKVLQMD	ILPRKVLQM	0	0	0	0	0	ILPRKVLQM	Sequence	0.021	39725.54	90.00
24	HLA-A0101	PAALSLDE	PAALSLDE-	0	8	1	0	0	PAALSLDE	Sequence	0.021	39940.61	90.00
10	HLA-A0101	ILPRKVLQ	ILPRKVLQ-	0	8	1	0	0	ILPRKVLQ	Sequence	0.021	39962.66	90.00
12	HLA-A0101	PRKVLQMD	PRKVLQMD-	0	8	1	0	0	PRKVLQMD	Sequence	0.019	40583.57	95.00

Protein Sequence. Allele HLA-A0101. Number of high binders 0. Number of weak binders 4. Number of peptides 84

Link to Allele Frequencies in Worldwide Populations HLA-A0101

Rank Threshold for Strong binding peptides 0.500
Rank Threshold for Weak binding peptides 2.000

---

pos	HLA	peptide	Core	Offset	I_pos	I_len	D_pos	D_len	iCore	Identity	1-log50k(aff)	Affinity(nM)	%Rank	BindLeve
1	HLA-A0201	VLPDADIYYI	VLDADIYYI	0	0	0	2	1	VLPDADIYYI	Sequence	0.729	18.80	0.25	<= SB
14	HLA-A0201	KVLQMDFLV	KVLQMDFLV	0	0	0	0	0	KVLQMDFLV	Sequence	0.723	20.07	0.30	<= SB
16	HLA-A0201	LQMDFLVHPA	LQMDFLVPA	0	0	0	7	1	LQMDFLVHPA	Sequence	0.686	29.93	0.40	<= SB
20	HLA-A0201	FLVHPAALSL	FLVPAALSL	0	0	0	3	1	FLVHPAALSL	Sequence	0.628	55.91	0.70	<= WB
17	HLA-A0201	QMDFLVHPA	QMDFLVHPA	0	0	0	0	0	QMDFLVHPA	Sequence	0.622	59.97	0.70	<= WB
26	HLA-A0201	ALSLDEPFI	ALSLDEPFI	0	0	0	0	0	ALSLDEPFI	Sequence	0.598	77.75	0.90	<= WB
13	HLA-A0201	RKVLQMDFLV	KVLQMDFLV	1	0	0	0	0	KVLQMDFLV	Sequence	0.494	238.37	1.80	<= WB
15	HLA-A0201	VLQMDFLV	VL-QMDFLV	0	2	1	0	0	VLQMDFLV	Sequence	0.470	310.40	2.50	
17	HLA-A0201	QMDFLVHPAA	QMDFLVHPA	0	0	0	8	1	QMDFLVHPAA	Sequence	0.400	660.00	3.50	
20	HLA-A0201	FLVHPAAL	FL-VHPAAL	0	2	1	0	0	FLVHPAAL	Sequence	0.397	680.74	3.50	
14	HLA-A0201	KVLQMDFLVH	KVLQMDFLV	0	0	0	0	0	KVLQMDFLV	Sequence	0.378	837.06	4.00	
9	HLA-A0201	YILPRKVLQM	YILPKVLQM	0	0	0	4	1	YILPRKVLQM	Sequence	0.372	888.42	4.00	
25	HLA-A0201	AALSLDEPFI	ALSLDEPFI	0	0	0	1	1	AALSLDEPFI	Sequence	0.369	921.86	4.00	
15	HLA-A0201	VLQMDFLVHP	VLMDFLVHP	0	0	0	2	1	VLQMDFLVHP	Sequence	0.366	952.80	4.00	
16	HLA-A0201	LQMDFLVHP	LQMDFLVHP	0	0	0	0	0	LQMDFLVHP	Sequence	0.302	1899.67	5.50	
10	HLA-A0201	ILPRKVLQM	ILPRKVLQM	0	0	0	0	0	ILPRKVLQM	Sequence	0.296	2026.12	6.00	
20	HLA-A0201	FLVHPAALS	FLVHPAALS	0	0	0	0	0	FLVHPAALS	Sequence	0.276	2523.63	6.50	
18	HLA-A0201	MDFLVHPA	-MDFLVHPA	0	0	1	0	0	MDFLVHPA	Sequence	0.276	2535.04	6.50	
21	HLA-A0201	LVHPAALSL	LVHPAALSL	0	0	0	0	0	LVHPAALSL	Sequence	0.260	3010.76	7.50	
26	HLA-A0201	ALSLDEPFIQ	ALSLDEPFI	0	0	0	0	0	ALSLDEPFI	Sequence	0.241	3680.22	8.00	
6	HLA-A0201	DIYYILPRKV	DIYYILPRV	0	0	0	8	1	DIYYILPRKV	Sequence	0.181	7057.62	12.00	
19	HLA-A0201	DFLVHPAALS	FLVHPAALS	1	0	0	0	0	FLVHPAALS	Sequence	0.177	7371.53	12.00	
2	HLA-A0201	LPDADIYYI	LPDADIYYI	0	0	0	0	0	LPDADIYYI	Sequence	0.173	7698.07	13.00	
1	HLA-A0201	VLPDADIYY	VLPDADIYY	0	0	0	0	0	VLPDADIYY	Sequence	0.172	7812.61	13.00	
18	HLA-A0201	MDFLVHPAAL	MDFLVPAAL	0	0	0	5	1	MDFLVHPAAL	Sequence	0.163	8578.27	14.00	
27	HLA-A0201	LSLDEPFI	-LSLDEPFI	0	0	1	0	0	LSLDEPFI	Sequence	0.161	8777.88	14.00	
14	HLA-A0201	KVLQMDFL	KVLQMDF-L	0	7	1	0	0	KVLQMDFL	Sequence	0.153	9565.22	15.00	
10	HLA-A0201	ILPRKVLQMD	ILPRKVLQM	0	0	0	0	0	ILPRKVLQM	Sequence	0.128	12480.94	18.00	
18	HLA-A0201	MDFLVHPAA	MDFLVHPAA	0	0	0	0	0	MDFLVHPAA	Sequence	0.125	12957.18	19.00	
16	HLA-A0201	LQMDFLVH	LQMDFLVH-	0	8	1	0	0	LQMDFLVH	Sequence	0.125	12978.36	19.00	
0	HLA-A0201	QVLPDADIYY	VLPDADIYY	1	0	0	0	0	VLPDADIYY	Sequence	0.111	14995.00	21.00	
9	HLA-A0201	YILPRKVL	YILPRKV-L	0	7	1	0	0	YILPRKVL	Sequence	0.110	15200.99	21.00	
15	HLA-A0201	VLQMDFLVH	VLQMDFLVH	0	0	0	0	0	VLQMDFLVH	Sequence	0.109	15446.20	21.00	
2	HLA-A0201	LPDADIYYIL	LPDADIYYL	0	0	0	8	1	LPDADIYYIL	Sequence	0.101	16807.81	23.00	
7	HLA-A0201	IYYILPRKV	IYYILPRKV	0	0	0	0	0	IYYILPRKV	Sequence	0.099	17063.43	23.00	
21	HLA-A0201	LVHPAALSLD	LVHPAALSL	0	0	0	0	0	LVHPAALSL	Sequence	0.095	17887.09	24.00	
17	HLA-A0201	QMDFLVHP	QM-DFLVHP	0	2	1	0	0	QMDFLVHP	Sequence	0.094	18170.32	25.00	
26	HLA-A0201	ALSLDEPF	AL-SLDEPF	0	2	1	0	0	ALSLDEPF	Sequence	0.092	18486.21	25.00	
13	HLA-A0201	RKVLQMDFL	RKVLQMDFL	0	0	0	0	0	RKVLQMDFL	Sequence	0.092	18546.52	25.00	
25	HLA-A0201	AALSLDEPF	AALSLDEPF	0	0	0	0	0	AALSLDEPF	Sequence	0.089	18990.82	26.00	
9	HLA-A0201	YILPRKVLQ	YILPRKVLQ	0	0	0	0	0	YILPRKVLQ	Sequence	0.086	19713.76	27.00	
28	HLA-A0201	SLDEPFIQ	SLDEPFIQ-	0	8	1	0	0	SLDEPFIQ	Sequence	0.085	19880.61	27.00	
7	HLA-A0201	IYYILPRKVL	IYYILPRVL	0	0	0	7	1	IYYILPRKVL	Sequence	0.082	20567.20	28.00	
1	HLA-A0201	VLPDADIY	VLPDADIY-	0	8	1	0	0	VLPDADIY	Sequence	0.078	21571.20	30.00	
5	HLA-A0201	ADIYYILPR	ADIYYILPR	0	0	0	0	0	ADIYYILPR	Sequence	0.077	21794.06	30.00	
11	HLA-A0201	LPRKVLQM	-LPRKVLQM	0	0	1	0	0	LPRKVLQM	Sequence	0.075	22319.31	31.00	
5	HLA-A0201	ADIYYILPRK	AIYYILPRK	0	0	0	1	1	ADIYYILPRK	Sequence	0.074	22506.52	31.00	
8	HLA-A0201	YYILPRKV	Y-YILPRKV	0	1	1	0	0	YYILPRKV	Sequence	0.069	23822.74	33.00	
24	HLA-A0201	PAALSLDEPF	PAALSLEPF	0	0	0	6	1	PAALSLDEPF	Sequence	0.061	25795.75	37.00	
19	HLA-A0201	DFLVHPAAL	DFLVHPAAL	0	0	0	0	0	DFLVHPAAL	Sequence	0.061	25976.96	38.00	
22	HLA-A0201	VHPAALSL	-VHPAALSL	0	0	1	0	0	VHPAALSL	Sequence	0.060	26177.58	38.00	
6	HLA-A0201	DIYYILPRK	DIYYILPRK	0	0	0	0	0	DIYYILPRK	Sequence	0.059	26521.96	39.00	
4	HLA-A0201	DADIYYILP	DADIYYILP	0	0	0	0	0	DADIYYILP	Sequence	0.057	26990.93	40.00	
4	HLA-A0201	DADIYYILPR	ADIYYILPR	1	0	0	0	0	ADIYYILPR	Sequence	0.056	27354.32	40.00	
2	HLA-A0201	LPDADIYY	-LPDADIYY	0	0	1	0	0	LPDADIYY	Sequence	0.056	27413.29	41.00	
8	HLA-A0201	YYILPRKVL	YYILPRKVL	0	0	0	0	0	YYILPRKVL	Sequence	0.054	27747.80	41.00	
12	HLA-A0201	PRKVLQMDFL	RKVLQMDFL	1	0	0	0	0	RKVLQMDFL	Sequence	0.053	28105.24	42.00	
6	HLA-A0201	DIYYILPR	DIY-YILPR	0	3	1	0	0	DIYYILPR	Sequence	0.050	29003.92	44.00	
11	HLA-A0201	LPRKVLQMDF	LPRKVLMDF	0	0	0	6	1	LPRKVLQMDF	Sequence	0.050	29225.70	45.00	
5	HLA-A0201	ADIYYILP	ADIYYILP-	0	8	1	0	0	ADIYYILP	Sequence	0.048	29599.62	46.00	
27	HLA-A0201	LSLDEPFIQ	LSLDEPFIQ	0	0	0	0	0	LSLDEPFIQ	Sequence	0.048	29768.56	46.00	
21	HLA-A0201	LVHPAALS	-LVHPAALS	0	0	1	0	0	LVHPAALS	Sequence	0.047	29924.53	46.00	
10	HLA-A0201	ILPRKVLQ	ILPRKVLQ-	0	8	1	0	0	ILPRKVLQ	Sequence	0.047	30001.36	47.00	
8	HLA-A0201	YYILPRKVLQ	YILPRKVLQ	0	0	0	1	1	YYILPRKVLQ	Sequence	0.044	30960.01	49.00	
4	HLA-A0201	DADIYYIL	DADIYYI-L	0	7	1	0	0	DADIYYIL	Sequence	0.044	31092.93	50.00	
3	HLA-A0201	PDADIYYIL	PDADIYYIL	0	0	0	0	0	PDADIYYIL	Sequence	0.042	31666.02	55.00	
3	HLA-A0201	PDADIYYI	-PDADIYYI	0	0	1	0	0	PDADIYYI	Sequence	0.042	31842.28	55.00	
0	HLA-A0201	QVLPDADIY	QVLPDADIY	0	0	0	0	0	QVLPDADIY	Sequence	0.041	32151.79	55.00	
11	HLA-A0201	LPRKVLQMD	LPRKVLQMD	0	0	0	0	0	LPRKVLQMD	Sequence	0.038	32980.12	55.00	
7	HLA-A0201	IYYILPRK	-IYYILPRK	0	0	1	0	0	IYYILPRK	Sequence	0.038	33206.41	60.00	

0	HLA-A0201	QVLPDADI	QVLP-DADI	0	4	1	0	0	QVLPDADI	Sequence	0.037	33653.11	60.00	
3	HLA-A0201	PDADIYYILP	DADIYYILP	1	0	0	0	0	DADIYYILP	Sequence	0.036	33704.13	60.00	
13	HLA-A0201	RKVLQMDF	R-KVLQMDF	0	1	1	0	0	RKVLQMDF	Sequence	0.030	36057.87	70.00	
19	HLA-A0201	DFLVHPAA	-DFLVHPAA	0	0	1	0	0	DFLVHPAA	Sequence	0.030	36153.57	70.00	
12	HLA-A0201	PRKVLQMDF	PRKVLQMDF	0	0	0	0	0	PRKVLQMDF	Sequence	0.028	36983.64	70.00	
23	HLA-A0201	HPAALSLDE	HPAALSLDE	0	0	0	0	0	HPAALSLDE	Sequence	0.026	37785.72	75.00	
23	HLA-A0201	HPAALSLDEP	HAALSLDEP	0	0	0	1	1	HPAALSLDEP	Sequence	0.026	37829.49	75.00	
25	HLA-A0201	AALSLDEP	AALSLDEP-	0	8	1	0	0	AALSLDEP	Sequence	0.024	38602.70	80.00	
24	HLA-A0201	PAALSLDEP	PAALSLDEP	0	0	0	0	0	PAALSLDEP	Sequence	0.024	38717.71	80.00	
22	HLA-A0201	VHPAALSLDE	VHPAALLDE	0	0	0	6	1	VHPAALSLDE	Sequence	0.022	39196.54	80.00	
22	HLA-A0201	VHPAALSLD	VHPAALSLD	0	0	0	0	0	VHPAALSLD	Sequence	0.022	39240.68	80.00	
12	HLA-A0201	PRKVLQMD	-PRKVLQMD	0	0	1	0	0	PRKVLQMD	Sequence	0.018	40973.60	90.00	
23	HLA-A0201	HPAALSLD	HPAALSLD-	0	8	1	0	0	HPAALSLD	Sequence	0.017	41621.95	90.00	
24	HLA-A0201	PAALSLDE	-PAALSLDE	0	0	1	0	0	PAALSLDE	Sequence	0.015	42556.85	95.00	

Protein Sequence. Allele HLA-A0201. Number of high binders 3. Number of weak binders 4. Number of peptides 84

Link to Allele Frequencies in Worldwide Populations HLA-A0201

Rank Threshold for Strong binding peptides   0.500
Rank Threshold for Weak binding peptides   2.000

---

pos	HLA	peptide	Core	Offset	I_pos	I_len	D_pos	D_len	iCore	Identity	1-log50k(aff)	Affinity(nM)	%Rank	BindLevel
5	HLA-A0301	ADIYYILPRK	AIYYILPRK	0	0	0	1	1	ADIYYILPRK	Sequence	0.719	21.00	0.08	<= SB
6	HLA-A0301	DIYYILPRK	DIYYILPRK	0	0	0	0	0	DIYYILPRK	Sequence	0.419	537.40	1.40	<= WB
7	HLA-A0301	IYYILPRK	-IYYILPRK	0	0	1	0	0	IYYILPRK	Sequence	0.361	1006.62	2.00	<= WB
14	HLA-A0301	KVLQMDFLVH	KVLQMFLVH	0	0	0	5	1	KVLQMDFLVH	Sequence	0.283	2328.27	3.50	
0	HLA-A0301	QVLPDADIYY	QVLPDAIYY	0	0	0	6	1	QVLPDADIYY	Sequence	0.257	3095.18	4.00	
6	HLA-A0301	DIYYILPRKV	DIYYILPRK	0	0	0	0	0	DIYYILPRK	Sequence	0.217	4755.05	5.00	
1	HLA-A0301	VLPDADIYY	VLPDADIYY	0	0	0	0	0	VLPDADIYY	Sequence	0.188	6510.70	6.00	
15	HLA-A0301	VLQMDFLVH	VLQMDFLVH	0	0	0	0	0	VLQMDFLVH	Sequence	0.159	8964.47	8.00	
5	HLA-A0301	ADIYYILPR	ADIYYILPR	0	0	0	0	0	ADIYYILPR	Sequence	0.153	9518.25	8.00	
21	HLA-A0301	LVHPAALSL	LVHPAALSL	0	0	0	0	0	LVHPAALSL	Sequence	0.120	13642.13	12.00	
14	HLA-A0301	KVLQMDFLV	KVLQMDFLV	0	0	0	0	0	KVLQMDFLV	Sequence	0.114	14602.73	13.00	
17	HLA-A0301	QMDFLVHPAA	QMFLVHPAA	0	0	0	2	1	QMDFLVHPAA	Sequence	0.113	14643.70	13.00	
20	HLA-A0301	FLVHPAALSL	FLHPAALSL	0	0	0	2	1	FLVHPAALSL	Sequence	0.113	14777.89	13.00	
15	HLA-A0301	VLQMDFLVHP	VLMDFLVHP	0	0	0	2	1	VLQMDFLVHP	Sequence	0.104	16144.38	14.00	
10	HLA-A0301	ILPRKVLQM	ILPRKVLQM	0	0	0	0	0	ILPRKVLQM	Sequence	0.100	16937.97	15.00	
0	HLA-A0301	QVLPDADIY	QVLPDADIY	0	0	0	0	0	QVLPDADIY	Sequence	0.094	18068.96	16.00	
26	HLA-A0301	ALSLDEPFI	ALSLDEPFI	0	0	0	0	0	ALSLDEPFI	Sequence	0.094	18072.09	16.00	
13	HLA-A0301	RKVLQMDFLV	RVLQMDFLV	0	0	0	1	1	RKVLQMDFLV	Sequence	0.092	18457.04	17.00	
9	HLA-A0301	YILPRKVLQM	YILPRKVLM	0	0	0	8	1	YILPRKVLQM	Sequence	0.090	18851.41	17.00	
4	HLA-A0301	DADIYYILPR	ADIYYILPR	1	0	0	0	0	ADIYYILPR	Sequence	0.087	19458.61	18.00	
9	HLA-A0301	YILPRKVLQ	YILPRKVLQ	0	0	0	0	0	YILPRKVLQ	Sequence	0.087	19594.66	18.00	
20	HLA-A0301	FLVHPAALS	FLVHPAALS	0	0	0	0	0	FLVHPAALS	Sequence	0.084	20072.54	19.00	
6	HLA-A0301	DIYYILPR	DIY-YILPR	0	3	1	0	0	DIYYILPR	Sequence	0.084	20132.58	19.00	
17	HLA-A0301	QMDFLVHPA	QMDFLVHPA	0	0	0	0	0	QMDFLVHPA	Sequence	0.081	20861.48	20.00	
21	HLA-A0301	LVHPAALSLD	LVHPAALSL	0	0	0	0	0	LVHPAALSL	Sequence	0.080	21071.99	20.00	
1	HLA-A0301	VLPDADIYYI	VLPDADIYY	0	0	0	0	0	VLPDADIYY	Sequence	0.077	21750.71	21.00	
16	HLA-A0301	LQMDFLVHPA	LMDFLVHPA	0	0	0	1	1	LQMDFLVHPA	Sequence	0.076	21933.16	21.00	
18	HLA-A0301	MDFLVHPAA	MDFLVHPAA	0	0	0	0	0	MDFLVHPAA	Sequence	0.071	23315.80	24.00	
18	HLA-A0301	MDFLVHPAAL	MFLVHPAAL	0	0	0	1	1	MDFLVHPAAL	Sequence	0.070	23363.53	24.00	
25	HLA-A0301	AALSLDEPFI	ALSLDEPFI	0	0	0	1	1	AALSLDEPFI	Sequence	0.070	23372.37	24.00	
16	HLA-A0301	LQMDFLVHP	LQMDFLVHP	0	0	0	0	0	LQMDFLVHP	Sequence	0.068	23998.41	25.00	
7	HLA-A0301	IYYILPRKVL	IYYLPRKVL	0	0	0	3	1	IYYILPRKVL	Sequence	0.065	24636.15	26.00	
7	HLA-A0301	IYYILPRKV	IYYILPRKV	0	0	0	0	0	IYYILPRKV	Sequence	0.063	25350.56	27.00	
10	HLA-A0301	ILPRKVLQMD	ILPRKVLQM	0	0	0	0	0	ILPRKVLQM	Sequence	0.062	25603.04	28.00	
8	HLA-A0301	YYILPRKVLQ	YILPRKVLQ	0	0	0	1	1	YYILPRKVLQ	Sequence	0.061	25723.85	28.00	
26	HLA-A0301	ALSLDEPFIQ	ALSLDEPFI	0	0	0	0	0	ALSLDEPFI	Sequence	0.056	27396.67	32.00	
8	HLA-A0301	YYILPRKVL	YYILPRKVL	0	0	0	0	0	YYILPRKVL	Sequence	0.052	28337.31	34.00	
13	HLA-A0301	RKVLQMDFL	RKVLQMDFL	0	0	0	0	0	RKVLQMDFL	Sequence	0.049	29349.91	37.00	
20	HLA-A0301	FLVHPAAL	FLVHPAAL-	0	8	1	0	0	FLVHPAAL	Sequence	0.049	29474.02	37.00	
16	HLA-A0301	LQMDFLVH	LQMD-FLVH	0	4	1	0	0	LQMDFLVH	Sequence	0.048	29595.77	37.00	
11	HLA-A0301	LPRKVLQMDF	LPRKVLMDF	0	0	0	6	1	LPRKVLQMDF	Sequence	0.047	30102.82	39.00	
14	HLA-A0301	KVLQMDFL	KV-LQMDFL	0	2	1	0	0	KVLQMDFL	Sequence	0.045	30768.31	41.00	
9	HLA-A0301	YILPRKVL	YILPRKVL-	0	8	1	0	0	YILPRKVL	Sequence	0.044	31132.66	42.00	
19	HLA-A0301	DFLVHPAALS	FLVHPAALS	1	0	0	0	0	FLVHPAALS	Sequence	0.043	31469.30	43.00	
19	HLA-A0301	DFLVHPAAL	DFLVHPAAL	0	0	0	0	0	DFLVHPAAL	Sequence	0.042	31624.59	43.00	
2	HLA-A0301	LPDADIYYI	LPDADIYYI	0	0	0	0	0	LPDADIYYI	Sequence	0.042	31904.71	44.00	
2	HLA-A0301	LPDADIYY	-LPDADIYY	0	0	1	0	0	LPDADIYY	Sequence	0.041	32213.76	45.00	
21	HLA-A0301	LVHPAALS	LVHPAALS-	0	8	1	0	0	LVHPAALS	Sequence	0.041	32243.40	45.00	
15	HLA-A0301	VLQMDFLV	VLQMDFLV-	0	8	1	0	0	VLQMDFLV	Sequence	0.040	32397.27	46.00	
1	HLA-A0301	VLPDADIY	VLPDADI-Y	0	7	1	0	0	VLPDADIY	Sequence	0.039	32832.02	47.00	
2	HLA-A0301	LPDADIYYIL	LPDAIYYIL	0	0	0	4	1	LPDADIYYIL	Sequence	0.039	32855.47	47.00	
28	HLA-A0301	SLDEPFIQ	SLDEPFIQ-	0	8	1	0	0	SLDEPFIQ	Sequence	0.039	32894.59	48.00	
26	HLA-A0301	ALSLDEPF	ALSLDEPF-	0	8	1	0	0	ALSLDEPF	Sequence	0.038	33118.15	48.00	
18	HLA-A0301	MDFLVHPA	MDFLVHPA-	0	8	1	0	0	MDFLVHPA	Sequence	0.038	33283.41	49.00	
22	HLA-A0301	VHPAALSL	-VHPAALSL	0	0	1	0	0	VHPAALSL	Sequence	0.037	33326.64	49.00	
25	HLA-A0301	AALSLDEPF	AALSLDEPF	0	0	0	0	0	AALSLDEPF	Sequence	0.037	33546.96	50.00	
17	HLA-A0301	QMDFLVHP	QMDFLVHP-	0	8	1	0	0	QMDFLVHP	Sequence	0.037	33564.38	50.00	
23	HLA-A0301	HPAALSLDE	HPAALSLDE	0	0	0	0	0	HPAALSLDE	Sequence	0.036	34023.64	55.00	
10	HLA-A0301	ILPRKVLQ	IL-PRKVLQ	0	2	1	0	0	ILPRKVLQ	Sequence	0.035	34178.22	55.00	
4	HLA-A0301	DADIYYILP	DADIYYILP	0	0	0	0	0	DADIYYILP	Sequence	0.035	34367.35	55.00	
13	HLA-A0301	RKVLQMDF	R-KVLQMDF	0	1	1	0	0	RKVLQMDF	Sequence	0.034	34659.35	55.00	
27	HLA-A0301	LSLDEPFI	-LSLDEPFI	0	0	1	0	0	LSLDEPFI	Sequence	0.031	35649.39	60.00	
12	HLA-A0301	PRKVLQMDFL	RKVLQMDFL	1	0	0	0	0	RKVLQMDFL	Sequence	0.031	35754.45	60.00	
5	HLA-A0301	ADIYYILP	ADIYYILP-	0	8	1	0	0	ADIYYILP	Sequence	0.030	36188.79	65.00	
23	HLA-A0301	HPAALSLDEP	HPAALSLEP	0	0	0	7	1	HPAALSLDEP	Sequence	0.030	36260.54	65.00	

8	HLA-A0301	YYILPRKV	Y-YILPRKV	0	1	1	0	0	YYILPRKV	Sequence	0.028	36780.92	65.00	
3	HLA-A0301	PDADIYYIL	PDADIYYIL	0	0	0	0	0	PDADIYYIL	Sequence	0.028	36919.68	65.00	
24	HLA-A0301	PAALSLDEPF	AALSLDEPF	1	0	0	0	0	AALSLDEPF	Sequence	0.027	37416.31	70.00	
11	HLA-A0301	LPRKVLQM	-LPRKVLQM	0	0	1	0	0	LPRKVLQM	Sequence	0.027	37455.61	70.00	
3	HLA-A0301	PDADIYYILP	PADIYYILP	0	0	0	1	1	PDADIYYILP	Sequence	0.026	37795.96	70.00	
27	HLA-A0301	LSLDEPFIQ	LSLDEPFIQ	0	0	0	0	0	LSLDEPFIQ	Sequence	0.026	37804.95	70.00	
12	HLA-A0301	PRKVLQMDF	PRKVLQMDF	0	0	0	0	0	PRKVLQMDF	Sequence	0.026	37911.87	70.00	
4	HLA-A0301	DADIYYIL	DADIYYIL-	0	8	1	0	0	DADIYYIL	Sequence	0.025	38156.71	70.00	
22	HLA-A0301	VHPAALSLD	VHPAALSLD	0	0	0	0	0	VHPAALSLD	Sequence	0.024	38682.97	75.00	
11	HLA-A0301	LPRKVLQMD	LPRKVLQMD	0	0	0	0	0	LPRKVLQMD	Sequence	0.023	39177.06	75.00	
19	HLA-A0301	DFLVHPAA	-DFLVHPAA	0	0	1	0	0	DFLVHPAA	Sequence	0.022	39554.41	80.00	
0	HLA-A0301	QVLPDADI	QV-LPDADI	0	2	1	0	0	QVLPDADI	Sequence	0.021	39654.25	80.00	
22	HLA-A0301	VHPAALSLDE	HPAALSLDE	1	0	0	0	0	HPAALSLDE	Sequence	0.021	39828.84	80.00	
23	HLA-A0301	HPAALSLD	HPAALSLD-	0	8	1	0	0	HPAALSLD	Sequence	0.019	40623.11	85.00	
3	HLA-A0301	PDADIYYI	-PDADIYYI	0	0	1	0	0	PDADIYYI	Sequence	0.017	41551.30	90.00	
24	HLA-A0301	PAALSLDEP	PAALSLDEP	0	0	0	0	0	PAALSLDEP	Sequence	0.015	42368.94	90.00	
25	HLA-A0301	AALSLDEP	AALS-LDEP	0	4	1	0	0	AALSLDEP	Sequence	0.011	44236.67	99.00	
24	HLA-A0301	PAALSLDE	-PAALSLDE	0	0	1	0	0	PAALSLDE	Sequence	0.011	44317.14	99.00	
12	HLA-A0301	PRKVLQMD	-PRKVLQMD	0	0	1	0	0	PRKVLQMD	Sequence	0.009	45388.62	99.00	

Protein Sequence. Allele HLA-A0301. Number of high binders 1. Number of weak binders 2. Number of peptides 84

Link to Allele Frequencies in Worldwide Populations HLA-A0301

Rank Threshold for Strong binding peptides 0.500
Rank Threshold for Weak binding peptides 2.000

----

pos	HLA	peptide	Core	Offset	I_pos	I_len	D_pos	D_len	iCore	Identity	1-log50k(aff)	Affinity(nM)	%Rank	BindLeve
7	HLA-A2402	IYYILPRKVL	IYYILPRVL	0	0	0	7	1	IYYILPRKVL	Sequence	0.349	1142.78	1.30	<= WB
8	HLA-A2402	YYILPRKVL	YYILPRKVL	0	0	0	0	0	YYILPRKVL	Sequence	0.336	1317.94	1.40	<= WB
7	HLA-A2402	IYYILPRKV	IYYILPRKV	0	0	0	0	0	IYYILPRKV	Sequence	0.308	1790.40	1.70	<= WB
1	HLA-A2402	VLPDADIYYI	VLPDAIYYI	0	0	0	5	1	VLPDADIYYI	Sequence	0.219	4668.24	3.50	
8	HLA-A2402	YYILPRKVLQ	YYILPRKVL	0	0	0	0	0	YYILPRKVL	Sequence	0.217	4789.03	3.50	
14	HLA-A2402	KVLQMDFLV	KVLQMDFLV	0	0	0	0	0	KVLQMDFLV	Sequence	0.178	7295.91	5.00	
10	HLA-A2402	ILPRKVLQM	ILPRKVLQM	0	0	0	0	0	ILPRKVLQM	Sequence	0.169	8034.03	5.00	
6	HLA-A2402	DIYYILPRKV	IYYILPRKV	1	0	0	0	0	IYYILPRKV	Sequence	0.135	11623.26	7.00	
3	HLA-A2402	PDADIYYIL	PDADIYYIL	0	0	0	0	0	PDADIYYIL	Sequence	0.134	11741.32	7.00	
13	HLA-A2402	RKVLQMDFLV	RVLQMDFLV	0	0	0	1	1	RKVLQMDFLV	Sequence	0.132	12005.44	7.50	
21	HLA-A2402	LVHPAALSL	LVHPAALSL	0	0	0	0	0	LVHPAALSL	Sequence	0.129	12353.30	7.50	
18	HLA-A2402	MDFLVHPAAL	MFLVHPAAL	0	0	0	1	1	MDFLVHPAAL	Sequence	0.125	12939.67	8.00	
8	HLA-A2402	YYILPRKV	YYI-LPRKV	0	3	1	0	0	YYILPRKV	Sequence	0.116	14265.56	8.50	
19	HLA-A2402	DFLVHPAAL	DFLVHPAAL	0	0	0	0	0	DFLVHPAAL	Sequence	0.113	14749.93	9.00	
9	HLA-A2402	YILPRKVLQM	YLPRKVLQM	0	0	0	1	1	YILPRKVLQM	Sequence	0.109	15386.15	9.50	
2	HLA-A2402	LPDADIYYIL	LDADIYYIL	0	0	0	1	1	LPDADIYYIL	Sequence	0.101	16710.98	10.00	
25	HLA-A2402	AALSLDEPF	AALSLDEPF	0	0	0	0	0	AALSLDEPF	Sequence	0.100	16864.28	11.00	
22	HLA-A2402	VHPAALSL	VHPAA-LSL	0	5	1	0	0	VHPAALSL	Sequence	0.095	17974.98	11.00	
22	HLA-A2402	VHPAALSLD	VHPAALSLD	0	0	0	0	0	VHPAALSLD	Sequence	0.086	19705.01	13.00	
20	HLA-A2402	FLVHPAALSL	FLHPAALSL	0	0	0	2	1	FLVHPAALSL	Sequence	0.080	20966.02	14.00	
14	HLA-A2402	KVLQMDFLVH	KVLQMDFLV	0	0	0	0	0	KVLQMDFLV	Sequence	0.077	21674.13	14.00	
10	HLA-A2402	ILPRKVLQMD	ILPRKVLQM	0	0	0	0	0	ILPRKVLQM	Sequence	0.073	22609.52	15.00	
11	HLA-A2402	LPRKVLQMDF	LPRKVLQMF	0	0	0	8	1	LPRKVLQMDF	Sequence	0.070	23368.83	16.00	
15	HLA-A2402	VLQMDFLV	V-LQMDFLV	0	1	1	0	0	VLQMDFLV	Sequence	0.070	23422.76	16.00	
16	HLA-A2402	LQMDFLVHP	LQMDFLVHP	0	0	0	0	0	LQMDFLVHP	Sequence	0.068	23900.21	16.00	
13	HLA-A2402	RKVLQMDF	R-KVLQMDF	0	1	1	0	0	RKVLQMDF	Sequence	0.066	24439.95	17.00	
26	HLA-A2402	ALSLDEPFI	ALSLDEPFI	0	0	0	0	0	ALSLDEPFI	Sequence	0.061	25738.32	18.00	
9	HLA-A2402	YILPRKVL	-YILPRKVL	0	0	1	0	0	YILPRKVL	Sequence	0.060	26001.99	18.00	
15	HLA-A2402	VLQMDFLVH	VLQMDFLVH	0	0	0	0	0	VLQMDFLVH	Sequence	0.060	26196.27	19.00	
21	HLA-A2402	LVHPAALSLD	LVHPAALSL	0	0	0	0	0	LVHPAALSL	Sequence	0.059	26383.14	19.00	
24	HLA-A2402	PAALSLDEPF	PAASLDEPF	0	0	0	3	1	PAALSLDEPF	Sequence	0.058	26593.81	19.00	
7	HLA-A2402	IYYILPRK	IYY-ILPRK	0	3	1	0	0	IYYILPRK	Sequence	0.058	26765.26	19.00	
12	HLA-A2402	PRKVLQMDF	PRKVLQMDF	0	0	0	0	0	PRKVLQMDF	Sequence	0.057	27003.80	19.00	
15	HLA-A2402	VLQMDFLVHP	VLQMDFLVP	0	0	0	8	1	VLQMDFLVHP	Sequence	0.057	27104.49	20.00	
4	HLA-A2402	DADIYYIL	D-ADIYYIL	0	1	1	0	0	DADIYYIL	Sequence	0.053	28241.82	21.00	
13	HLA-A2402	RKVLQMDFL	RKVLQMDFL	0	0	0	0	0	RKVLQMDFL	Sequence	0.053	28292.59	21.00	
3	HLA-A2402	PDADIYYILP	PDADIYYIL	0	0	0	0	0	PDADIYYIL	Sequence	0.048	29873.09	23.00	
9	HLA-A2402	YILPRKVLQ	YILPRKVLQ	0	0	0	0	0	YILPRKVLQ	Sequence	0.046	30397.04	24.00	
16	HLA-A2402	LQMDFLVHPA	LQMFLVHPA	0	0	0	3	1	LQMDFLVHPA	Sequence	0.045	30673.59	24.00	
25	HLA-A2402	AALSLDEPFI	AALSLEPFI	0	0	0	5	1	AALSLDEPFI	Sequence	0.044	31099.67	25.00	
2	HLA-A2402	LPDADIYYI	LPDADIYYI	0	0	0	0	0	LPDADIYYI	Sequence	0.043	31384.97	25.00	
19	HLA-A2402	DFLVHPAALS	DFLVHPAAL	0	0	0	0	0	DFLVHPAAL	Sequence	0.042	31800.62	26.00	
20	HLA-A2402	FLVHPAAL	-FLVHPAAL	0	0	1	0	0	FLVHPAAL	Sequence	0.041	32029.21	26.00	
14	HLA-A2402	KVLQMDFL	KVLQMDF-L	0	7	1	0	0	KVLQMDFL	Sequence	0.041	32052.10	27.00	
26	HLA-A2402	ALSLDEPF	A-LSLDEPF	0	1	1	0	0	ALSLDEPF	Sequence	0.040	32585.71	27.00	
22	HLA-A2402	VHPAALSLDE	VHPAALSLE	0	0	0	8	1	VHPAALSLDE	Sequence	0.039	32871.83	28.00	
1	HLA-A2402	VLPDADIYY	VLPDADIYY	0	0	0	0	0	VLPDADIYY	Sequence	0.038	33196.01	29.00	
27	HLA-A2402	LSLDEPFI	LSLDEPF-I	0	7	1	0	0	LSLDEPFI	Sequence	0.036	34040.56	30.00	
3	HLA-A2402	PDADIYYI	PDADIYY-I	0	7	1	0	0	PDADIYYI	Sequence	0.033	34926.64	32.00	
11	HLA-A2402	LPRKVLQM	L-PRKVLQM	0	1	1	0	0	LPRKVLQM	Sequence	0.033	34939.88	32.00	
27	HLA-A2402	LSLDEPFIQ	LSLDEPFIQ	0	0	0	0	0	LSLDEPFIQ	Sequence	0.030	36179.79	35.00	
16	HLA-A2402	LQMDFLVH	LQM-DFLVH	0	3	1	0	0	LQMDFLVH	Sequence	0.027	37143.24	38.00	
20	HLA-A2402	FLVHPAALS	FLVHPAALS	0	0	0	0	0	FLVHPAALS	Sequence	0.024	38485.10	42.00	
26	HLA-A2402	ALSLDEPFIQ	ALSLDEPFI	0	0	0	0	0	ALSLDEPFI	Sequence	0.024	38592.25	42.00	
12	HLA-A2402	PRKVLQMDFL	RKVLQMDFL	1	0	0	0	0	RKVLQMDFL	Sequence	0.024	38711.45	42.00	
10	HLA-A2402	ILPRKVLQ	ILPRKVLQ-	0	8	1	0	0	ILPRKVLQ	Sequence	0.021	39711.81	46.00	
18	HLA-A2402	MDFLVHPAA	MDFLVHPAA	0	0	0	0	0	MDFLVHPAA	Sequence	0.020	40215.54	48.00	
17	HLA-A2402	QMDFLVHPA	QMDFLVHPA	0	0	0	0	0	QMDFLVHPA	Sequence	0.020	40454.25	49.00	
5	HLA-A2402	ADIYYILPR	ADIYYILPR	0	0	0	0	0	ADIYYILPR	Sequence	0.018	40953.66	55.00	
17	HLA-A2402	QMDFLVHPAA	QMFLVHPAA	0	0	0	2	1	QMDFLVHPAA	Sequence	0.017	41488.41	55.00	

17	HLA-A2402	QMDFLVHP	-QMDFLVHP	0	0	1	0	0	QMDFLVHP	Sequence	0.017	41492.02	55.00
5	HLA-A2402	ADIYYILP	ADI-YYILP	0	3	1	0	0	ADIYYILP	Sequence	0.016	42028.79	60.00
0	HLA-A2402	QVLPDADIY	QVLPDADIY	0	0	0	0	0	QVLPDADIY	Sequence	0.016	42045.62	60.00
11	HLA-A2402	LPRKVLQMD	LPRKVLQMD	0	0	0	0	0	LPRKVLQMD	Sequence	0.016	42076.11	60.00
4	HLA-A2402	DADIYYILP	DADIYYILP	0	0	0	0	0	DADIYYILP	Sequence	0.015	42385.45	60.00
23	HLA-A2402	HPAALSLD	-HPAALSLD	0	0	1	0	0	HPAALSLD	Sequence	0.015	42523.25	60.00
0	HLA-A2402	QVLPDADI	QVLPD-ADI	0	5	1	0	0	QVLPDADI	Sequence	0.015	42579.89	60.00
0	HLA-A2402	QVLPDADIYY	VLPDADIYY	1	0	0	0	0	VLPDADIYY	Sequence	0.015	42696.59	60.00
23	HLA-A2402	HPAALSLDE	HPAALSLDE	0	0	0	0	0	HPAALSLDE	Sequence	0.014	43168.54	65.00
6	HLA-A2402	DIYYILPRK	DIYYILPRK	0	0	0	0	0	DIYYILPRK	Sequence	0.014	43181.63	65.00
23	HLA-A2402	HPAALSLDEP	HPAALSLEP	0	0	0	7	1	HPAALSLDEP	Sequence	0.013	43669.31	70.00
1	HLA-A2402	VLPDADIY	VLPDA-DIY	0	5	1	0	0	VLPDADIY	Sequence	0.012	43730.33	70.00
5	HLA-A2402	ADIYYILPRK	AIYYILPRK	0	0	0	1	1	ADIYYILPRK	Sequence	0.012	43765.34	70.00
19	HLA-A2402	DFLVHPAA	DFL-VHPAA	0	3	1	0	0	DFLVHPAA	Sequence	0.012	43844.02	70.00
24	HLA-A2402	PAALSLDEP	PAALSLDEP	0	0	0	0	0	PAALSLDEP	Sequence	0.012	44064.21	70.00
4	HLA-A2402	DADIYYILPR	DADIYYILP	0	0	0	0	0	DADIYYILP	Sequence	0.011	44280.23	75.00
21	HLA-A2402	LVHPAALS	LVHPAALS-	0	8	1	0	0	LVHPAALS	Sequence	0.011	44589.86	75.00
28	HLA-A2402	SLDEPFIQ	S-LDEPFIQ	0	1	1	0	0	SLDEPFIQ	Sequence	0.010	44894.36	80.00
2	HLA-A2402	LPDADIYY	L-PDADIYY	0	1	1	0	0	LPDADIYY	Sequence	0.009	45125.68	80.00
18	HLA-A2402	MDFLVHPA	MDF-LVHPA	0	3	1	0	0	MDFLVHPA	Sequence	0.009	45521.42	85.00
6	HLA-A2402	DIYYILPR	DIYYILPR-	0	8	1	0	0	DIYYILPR	Sequence	0.008	45758.95	85.00
24	HLA-A2402	PAALSLDE	P-AALSLDE	0	1	1	0	0	PAALSLDE	Sequence	0.007	46426.72	90.00
12	HLA-A2402	PRKVLQMD	P-RKVLQMD	0	1	1	0	0	PRKVLQMD	Sequence	0.006	46636.66	95.00
25	HLA-A2402	AALSLDEP	A-ALSLDEP	0	1	1	0	0	AALSLDEP	Sequence	0.006	46866.80	95.00

Protein Sequence. Allele HLA-A2402. Number of high binders 0. Number of weak binders 3. Number of peptides 84

Link to Allele Frequencies in Worldwide Populations HLA-A2402

Rank Threshold for Strong binding peptides 0.500
Rank Threshold for Weak binding peptides 2.000

----

pos	HLA	peptide	Core	Offset	I_pos	I_len	D_pos	D_len	iCore	Identity	1-log50k(aff)	Affinity(nM)	%Rank	BindLeve
0	HLA-A2601	QVLPDADIYY	QVPDADIYY	0	0	0	2	1	QVLPDADIYY	Sequence	0.295	2065.43	0.80	<= WB
9	HLA-A2601	YILPRKVLQM	YIPRKVLQM	0	0	0	2	1	YILPRKVLQM	Sequence	0.172	7788.55	2.50	
6	HLA-A2601	DIYYILPRKV	DIYYILPRV	0	0	0	8	1	DIYYILPRKV	Sequence	0.154	9476.11	3.00	
1	HLA-A2601	VLPDADIYY	VLPDADIYY	0	0	0	0	0	VLPDADIYY	Sequence	0.144	10487.31	3.50	
0	HLA-A2601	QVLPDADIY	QVLPDADIY	0	0	0	0	0	QVLPDADIY	Sequence	0.138	11186.00	4.00	
6	HLA-A2601	DIYYILPRK	DIYYILPRK	0	0	0	0	0	DIYYILPRK	Sequence	0.136	11428.47	4.00	
20	HLA-A2601	FLVHPAALSL	FVHPAALSL	0	0	0	1	1	FLVHPAALSL	Sequence	0.114	14618.85	5.50	
21	HLA-A2601	LVHPAALSL	LVHPAALSL	0	0	0	0	0	LVHPAALSL	Sequence	0.113	14705.62	5.50	
4	HLA-A2601	DADIYYILPR	DAIYYILPR	0	0	0	2	1	DADIYYILPR	Sequence	0.108	15534.19	6.00	
10	HLA-A2601	ILPRKVLQM	ILPRKVLQM	0	0	0	0	0	ILPRKVLQM	Sequence	0.093	18182.91	8.00	
20	HLA-A2601	FLVHPAALS	FLVHPAALS	0	0	0	0	0	FLVHPAALS	Sequence	0.090	18820.84	8.50	
25	HLA-A2601	AALSLDEPF	AALSLDEPF	0	0	0	0	0	AALSLDEPF	Sequence	0.083	20329.80	11.00	
21	HLA-A2601	LVHPAALSLD	LVHPAALSL	0	0	0	0	0	LVHPAALSL	Sequence	0.080	21117.87	12.00	
1	HLA-A2601	VLPDADIYYI	VLPDADIYY	0	0	0	0	0	VLPDADIYY	Sequence	0.071	23314.79	15.00	
8	HLA-A2601	YYILPRKVL	YYILPRKVL	0	0	0	0	0	YYILPRKVL	Sequence	0.069	23626.88	15.00	
19	HLA-A2601	DFLVHPAAL	DFLVHPAAL	0	0	0	0	0	DFLVHPAAL	Sequence	0.068	23844.15	16.00	
5	HLA-A2601	ADIYYILPR	ADIYYILPR	0	0	0	0	0	ADIYYILPR	Sequence	0.067	24257.64	17.00	
18	HLA-A2601	MDFLVHPAA	MDFLVHPAA	0	0	0	0	0	MDFLVHPAA	Sequence	0.067	24338.61	17.00	
11	HLA-A2601	LPRKVLQMDF	LPRKVLQMF	0	0	0	8	1	LPRKVLQMDF	Sequence	0.066	24376.04	17.00	
9	HLA-A2601	YILPRKVLQ	YILPRKVLQ	0	0	0	0	0	YILPRKVLQ	Sequence	0.065	24802.77	18.00	
2	HLA-A2601	LPDADIYY	-LPDADIYY	0	0	1	0	0	LPDADIYY	Sequence	0.064	24926.26	18.00	
6	HLA-A2601	DIYYILPR	DIYYILPR-	0	8	1	0	0	DIYYILPR	Sequence	0.063	25298.22	19.00	
13	HLA-A2601	RKVLQMDFL	RKVLQMDFL	0	0	0	0	0	RKVLQMDFL	Sequence	0.062	25461.88	19.00	
18	HLA-A2601	MDFLVHPAAL	MDFLVHAAL	0	0	0	6	1	MDFLVHPAAL	Sequence	0.061	25975.55	20.00	
4	HLA-A2601	DADIYYILP	DADIYYILP	0	0	0	0	0	DADIYYILP	Sequence	0.060	26040.84	21.00	
16	HLA-A2601	LQMDFLVHPA	LQMDFLHPA	0	0	0	6	1	LQMDFLVHPA	Sequence	0.060	26172.48	21.00	
24	HLA-A2601	PAALSLDEPF	PAALSLDPF	0	0	0	7	1	PAALSLDEPF	Sequence	0.058	26591.22	22.00	
17	HLA-A2601	QMDFLVHPA	QMDFLVHPA	0	0	0	0	0	QMDFLVHPA	Sequence	0.058	26630.95	22.00	
14	HLA-A2601	KVLQMDFLV	KVLQMDFLV	0	0	0	0	0	KVLQMDFLV	Sequence	0.058	26690.95	22.00	
16	HLA-A2601	LQMDFLVHP	LQMDFLVHP	0	0	0	0	0	LQMDFLVHP	Sequence	0.058	26740.95	22.00	
2	HLA-A2601	LPDADIYYI	LPDADIYYI	0	0	0	0	0	LPDADIYYI	Sequence	0.057	27101.55	23.00	
22	HLA-A2601	VHPAALSLD	VHPAALSLD	0	0	0	0	0	VHPAALSLD	Sequence	0.056	27299.62	24.00	
2	HLA-A2601	LPDADIYYIL	LPADIYYIL	0	0	0	2	1	LPDADIYYIL	Sequence	0.056	27416.84	24.00	
23	HLA-A2601	HPAALSLDE	HPAALSLDE	0	0	0	0	0	HPAALSLDE	Sequence	0.054	27933.36	26.00	
26	HLA-A2601	ALSLDEPFI	ALSLDEPFI	0	0	0	0	0	ALSLDEPFI	Sequence	0.054	27961.17	26.00	
5	HLA-A2601	ADIYYILPRK	DIYYILPRK	1	0	0	0	0	DIYYILPRK	Sequence	0.054	27990.54	26.00	
15	HLA-A2601	VLQMDFLVH	VLQMDFLVH	0	0	0	0	0	VLQMDFLVH	Sequence	0.054	28009.63	26.00	
3	HLA-A2601	PDADIYYIL	PDADIYYIL	0	0	0	0	0	PDADIYYIL	Sequence	0.053	28051.79	26.00	
27	HLA-A2601	LSLDEPFIQ	LSLDEPFIQ	0	0	0	0	0	LSLDEPFIQ	Sequence	0.050	29248.78	30.00	
17	HLA-A2601	QMDFLVHPAA	QMFLVHPAA	0	0	0	2	1	QMDFLVHPAA	Sequence	0.049	29444.06	31.00	
8	HLA-A2601	YYILPRKVLQ	YYILPRVLQ	0	0	0	6	1	YYILPRKVLQ	Sequence	0.048	29631.35	32.00	
13	HLA-A2601	RKVLQMDFLV	RVLQMDFLV	0	0	0	1	1	RKVLQMDFLV	Sequence	0.048	29745.38	32.00	
20	HLA-A2601	FLVHPAAL	FLV-HPAAL	0	3	1	0	0	FLVHPAAL	Sequence	0.047	29914.50	33.00	
12	HLA-A2601	PRKVLQMDF	PRKVLQMDF	0	0	0	0	0	PRKVLQMDF	Sequence	0.047	30197.42	34.00	
19	HLA-A2601	DFLVHPAALS	DLVHPAALS	0	0	0	1	1	DFLVHPAALS	Sequence	0.045	30672.26	36.00	
11	HLA-A2601	LPRKVLQMD	LPRKVLQMD	0	0	0	0	0	LPRKVLQMD	Sequence	0.044	31148.16	38.00	
7	HLA-A2601	IYYILPRKV	IYYILPRKV	0	0	0	0	0	IYYILPRKV	Sequence	0.043	31434.58	39.00	
22	HLA-A2601	VHPAALSLDE	VHPAALSLE	0	0	0	8	1	VHPAALSLDE	Sequence	0.042	31881.59	42.00	
10	HLA-A2601	ILPRKVLQMD	ILPRKVLQM	0	0	0	0	0	ILPRKVLQM	Sequence	0.041	32091.65	43.00	
7	HLA-A2601	IYYILPRKVL	IYILPRKVL	0	0	0	1	1	IYYILPRKVL	Sequence	0.040	32468.15	45.00	
12	HLA-A2601	PRKVLQMDFL	PRVLQMDFL	0	0	0	2	1	PRKVLQMDFL	Sequence	0.040	32555.75	45.00	
14	HLA-A2601	KVLQMDFLVH	KVLQMFLVH	0	0	0	5	1	KVLQMDFLVH	Sequence	0.039	32701.89	46.00	
15	HLA-A2601	VLQMDFLVHP	VLMDFLVHP	0	0	0	2	1	VLQMDFLVHP	Sequence	0.039	32726.32	46.00	
25	HLA-A2601	AALSLDEPFI	AALSLDEPF	0	0	0	0	0	AALSLDEPF	Sequence	0.039	32767.78	46.00	
4	HLA-A2601	DADIYYIL	DA-DIYYIL	0	2	1	0	0	DADIYYIL	Sequence	0.038	32968.34	47.00	

22	HLA-A2601	VHPAALSL	-VHPAALSL	0	0	1	0	0	VHPAALSL	Sequence	0.038	33044.79	48.00
23	HLA-A2601	HPAALSLDEP	HPAALSLEP	0	0	0	7	1	HPAALSLDEP	Sequence	0.038	33194.21	48.00
24	HLA-A2601	PAALSLDEP	PAALSLDEP	0	0	0	0	0	PAALSLDEP	Sequence	0.038	33262.53	49.00
16	HLA-A2601	LQMDFLVH	LQMD-FLVH	0	4	1	0	0	LQMDFLVH	Sequence	0.036	33840.43	55.00
11	HLA-A2601	LPRKVLQM	-LPRKVLQM	0	0	1	0	0	LPRKVLQM	Sequence	0.036	34002.30	55.00
1	HLA-A2601	VLPDADIY	VLPDADI-Y	0	7	1	0	0	VLPDADIY	Sequence	0.036	34037.25	55.00
15	HLA-A2601	VLQMDFLV	-VLQMDFLV	0	0	1	0	0	VLQMDFLV	Sequence	0.034	34463.04	60.00
18	HLA-A2601	MDFLVHPA	MDFLVHPA-	0	8	1	0	0	MDFLVHPA	Sequence	0.034	34537.70	60.00
5	HLA-A2601	ADIYYILP	ADIYYILP-	0	8	1	0	0	ADIYYILP	Sequence	0.033	34821.74	60.00
14	HLA-A2601	KVLQMDFL	-KVLQMDFL	0	0	1	0	0	KVLQMDFL	Sequence	0.033	34924.75	60.00
7	HLA-A2601	IYYILPRK	-IYYILPRK	0	0	1	0	0	IYYILPRK	Sequence	0.033	35086.48	60.00
21	HLA-A2601	LVHPAALS	LVHPAALS-	0	8	1	0	0	LVHPAALS	Sequence	0.033	35122.56	60.00
3	HLA-A2601	PDADIYYILP	PDAIYYILP	0	0	0	3	1	PDADIYYILP	Sequence	0.032	35207.79	60.00
26	HLA-A2601	ALSLDEPFIQ	ALSLDEPFQ	0	0	0	8	1	ALSLDEPFIQ	Sequence	0.032	35313.09	65.00
9	HLA-A2601	YILPRKVL	YILPRKVL-	0	8	1	0	0	YILPRKVL	Sequence	0.032	35555.39	65.00
26	HLA-A2601	ALSLDEPF	ALSLD-EPF	0	5	1	0	0	ALSLDEPF	Sequence	0.030	36262.48	70.00
19	HLA-A2601	DFLVHPAA	D-FLVHPAA	0	1	1	0	0	DFLVHPAA	Sequence	0.029	36664.50	70.00
13	HLA-A2601	RKVLQMDF	RKVLQMD-F	0	7	1	0	0	RKVLQMDF	Sequence	0.029	36704.60	70.00
23	HLA-A2601	HPAALSLD	HPAALSLD-	0	8	1	0	0	HPAALSLD	Sequence	0.028	36892.93	75.00
27	HLA-A2601	LSLDEPFI	LSLDE-PFI	0	5	1	0	0	LSLDEPFI	Sequence	0.027	37434.15	75.00
17	HLA-A2601	QMDFLVHP	QMDFLVHP-	0	8	1	0	0	QMDFLVHP	Sequence	0.025	37950.44	80.00
8	HLA-A2601	YYILPRKV	YYILPR-KV	0	6	1	0	0	YYILPRKV	Sequence	0.025	38021.54	80.00
10	HLA-A2601	ILPRKVLQ	ILPRKV-LQ	0	6	1	0	0	ILPRKVLQ	Sequence	0.024	38361.62	80.00
0	HLA-A2601	QVLPDADI	QVLPDAD-I	0	7	1	0	0	QVLPDADI	Sequence	0.021	39691.17	90.00
3	HLA-A2601	PDADIYYI	PDADIY-YI	0	6	1	0	0	PDADIYYI	Sequence	0.020	40090.41	90.00
28	HLA-A2601	SLDEPFIQ	SLDEPFI-Q	0	7	1	0	0	SLDEPFIQ	Sequence	0.020	40234.68	95.00
24	HLA-A2601	PAALSLDE	-PAALSLDE	0	0	1	0	0	PAALSLDE	Sequence	0.020	40362.00	95.00
25	HLA-A2601	AALSLDEP	AALSLDEP-	0	8	1	0	0	AALSLDEP	Sequence	0.019	40878.85	95.00
12	HLA-A2601	PRKVLQMD	-PRKVLQMD	0	0	1	0	0	PRKVLQMD	Sequence	0.017	41378.13	99.00

Protein Sequence. Allele HLA-A2601. Number of high binders 0. Number of weak binders 1. Number of peptides 84

Link to Allele Frequencies in Worldwide Populations HLA-A2601

Rank Threshold for Strong binding peptides 0.500
Rank Threshold for Weak binding peptides 2.000

---

pos	HLA	peptide	Core	Offset	I_pos	I_len	D_pos	D_len	iCore	Identity	1-log50k(aff)	Affinity(nM)	%Rank	BindLeve
11	HLA-B0702	LPRKVLQMDF	LPRKVLMDF	0	0	0	6	1	LPRKVLQMDF	Sequence	0.619	61.51	0.30	<= SB
11	HLA-B0702	LPRKVLQM	LPRKV-LQM	0	5	1	0	0	LPRKVLQM	Sequence	0.613	66.18	0.30	<= SB
21	HLA-B0702	LVHPAALSL	LVHPAALSL	0	0	0	0	0	LVHPAALSL	Sequence	0.541	142.82	0.60	<= WB
2	HLA-B0702	LPDADIYYIL	LPADIYYIL	0	0	0	2	1	LPDADIYYIL	Sequence	0.381	811.59	1.50	<= WB
20	HLA-B0702	FLVHPAALSL	FVHPAALSL	0	0	0	1	1	FLVHPAALSL	Sequence	0.288	2214.41	3.00	
11	HLA-B0702	LPRKVLQMD	LPRKVLQMD	0	0	0	0	0	LPRKVLQMD	Sequence	0.268	2746.30	3.00	
23	HLA-B0702	HPAALSLDE	HPAALSLDE	0	0	0	0	0	HPAALSLDE	Sequence	0.246	3505.98	3.50	
21	HLA-B0702	LVHPAALSLD	LVHPAALSL	0	0	0	0	0	LVHPAALSL	Sequence	0.189	6448.02	5.00	
9	HLA-B0702	YILPRKVL	YIL-PRKVL	0	3	1	0	0	YILPRKVL	Sequence	0.174	7633.79	6.00	
8	HLA-B0702	YYILPRKVL	YYILPRKVL	0	0	0	0	0	YYILPRKVL	Sequence	0.170	7914.79	6.00	
23	HLA-B0702	HPAALSLD	HPAALSLD-	0	8	1	0	0	HPAALSLD	Sequence	0.169	8026.21	6.00	
2	HLA-B0702	LPDADIYYI	LPDADIYYI	0	0	0	0	0	LPDADIYYI	Sequence	0.169	8075.16	6.00	
10	HLA-B0702	ILPRKVLQMD	IPRKVLQMD	0	0	0	1	1	ILPRKVLQMD	Sequence	0.166	8264.50	6.00	
9	HLA-B0702	YILPRKVLQM	YILPRKLQM	0	0	0	6	1	YILPRKVLQM	Sequence	0.164	8502.68	6.00	
23	HLA-B0702	HPAALSLDEP	HPAALSLEP	0	0	0	7	1	HPAALSLDEP	Sequence	0.161	8800.51	6.50	
25	HLA-B0702	AALSLDEPF	AALSLDEPF	0	0	0	0	0	AALSLDEPF	Sequence	0.133	11842.74	8.00	
22	HLA-B0702	VHPAALSLDE	VPAALSLDE	0	0	0	1	1	VHPAALSLDE	Sequence	0.117	14143.69	9.50	
22	HLA-B0702	VHPAALSL	-VHPAALSL	0	0	1	0	0	VHPAALSL	Sequence	0.114	14615.37	10.00	
14	HLA-B0702	KVLQMDFLV	KVLQMDFLV	0	0	0	0	0	KVLQMDFLV	Sequence	0.106	15885.86	11.00	
9	HLA-B0702	YILPRKVLQ	YILPRKVLQ	0	0	0	0	0	YILPRKVLQ	Sequence	0.090	18860.59	14.00	
20	HLA-B0702	FLVHPAAL	FLVHP-AAL	0	5	1	0	0	FLVHPAAL	Sequence	0.089	19163.79	14.00	
1	HLA-B0702	VLPDADIYYI	VPDADIYYI	0	0	0	1	1	VLPDADIYYI	Sequence	0.083	20285.64	15.00	
13	HLA-B0702	RKVLQMDFL	RKVLQMDFL	0	0	0	0	0	RKVLQMDFL	Sequence	0.082	20504.98	16.00	
7	HLA-B0702	IYYILPRKVL	IYYLPRKVL	0	0	0	3	1	IYYILPRKVL	Sequence	0.080	21048.76	16.00	
12	HLA-B0702	PRKVLQMD	-PRKVLQMD	0	0	1	0	0	PRKVLQMD	Sequence	0.075	22156.42	18.00	
10	HLA-B0702	ILPRKVLQM	ILPRKVLQM	0	0	0	0	0	ILPRKVLQM	Sequence	0.072	23005.82	19.00	
18	HLA-B0702	MDFLVHPAA	MDFLVHPAA	0	0	0	0	0	MDFLVHPAA	Sequence	0.067	24101.19	21.00	
8	HLA-B0702	YYILPRKVLQ	YYILPRKVL	0	0	0	0	0	YYILPRKVL	Sequence	0.066	24393.72	21.00	
17	HLA-B0702	QMDFLVHPA	QMDFLVHPA	0	0	0	0	0	QMDFLVHPA	Sequence	0.066	24514.10	21.00	
18	HLA-B0702	MDFLVHPAAL	MFLVHPAAL	0	0	0	1	1	MDFLVHPAAL	Sequence	0.064	24915.20	22.00	
15	HLA-B0702	VLQMDFLVH	VLQMDFLVH	0	0	0	0	0	VLQMDFLVH	Sequence	0.063	25324.52	23.00	
14	HLA-B0702	KVLQMDFLVH	KVLQMFLVH	0	0	0	5	1	KVLQMDFLVH	Sequence	0.061	25848.84	24.00	
13	HLA-B0702	RKVLQMDFLV	RVLQMDFLV	0	0	0	1	1	RKVLQMDFLV	Sequence	0.060	26083.70	24.00	
24	HLA-B0702	PAALSLDE	-PAALSLDE	0	0	1	0	0	PAALSLDE	Sequence	0.058	26667.85	25.00	
16	HLA-B0702	LQMDFLVHPA	LQMFLVHPA	0	0	0	3	1	LQMDFLVHPA	Sequence	0.057	27036.54	26.00	
0	HLA-B0702	QVLPDADIY	QVLPDADIY	0	0	0	0	0	QVLPDADIY	Sequence	0.052	28429.14	29.00	
25	HLA-B0702	AALSLDEPFI	AALSLDEPI	0	0	0	8	1	AALSLDEPFI	Sequence	0.051	28928.07	31.00	
14	HLA-B0702	KVLQMDFL	KVLQMDF-L	0	7	1	0	0	KVLQMDFL	Sequence	0.050	29175.46	31.00	
19	HLA-B0702	DFLVHPAAL	DFLVHPAAL	0	0	0	0	0	DFLVHPAAL	Sequence	0.049	29321.04	32.00	
2	HLA-B0702	LPDADIYY	LP-DADIYY	0	2	1	0	0	LPDADIYY	Sequence	0.049	29462.54	32.00	
24	HLA-B0702	PAALSLDEPF	AALSLDEPF	1	0	0	0	0	AALSLDEPF	Sequence	0.047	30099.89	34.00	
22	HLA-B0702	VHPAALSLD	VHPAALSLD	0	0	0	0	0	VHPAALSLD	Sequence	0.045	30742.36	36.00	
5	HLA-B0702	ADIYYILPR	ADIYYILPR	0	0	0	0	0	ADIYYILPR	Sequence	0.044	31011.96	37.00	
26	HLA-B0702	ALSLDEPFI	ALSLDEPFI	0	0	0	0	0	ALSLDEPFI	Sequence	0.044	31171.75	37.00	
1	HLA-B0702	VLPDADIYY	VLPDADIYY	0	0	0	0	0	VLPDADIYY	Sequence	0.043	31448.87	38.00	
7	HLA-B0702	IYYILPRKV	IYYILPRKV	0	0	0	0	0	IYYILPRKV	Sequence	0.043	31482.58	38.00	
3	HLA-B0702	PDADIYYI	-PDADIYYI	0	0	1	0	0	PDADIYYI	Sequence	0.042	31571.96	38.00	
20	HLA-B0702	FLVHPAALS	FLVHPAALS	0	0	0	0	0	FLVHPAALS	Sequence	0.042	31742.18	39.00	
3	HLA-B0702	PDADIYYIL	PDADIYYIL	0	0	0	0	0	PDADIYYIL	Sequence	0.042	31785.49	39.00	
27	HLA-B0702	LSLDEPFIQ	LSLDEPFIQ	0	0	0	0	0	LSLDEPFIQ	Sequence	0.041	32010.85	40.00	

21	HLA-B0702	LVHPAALS	LVHPAALS-	0	8	1	0	0	LVHPAALS	Sequence	0.040	32323.03	41.00
16	HLA-B0702	LQMDFLVH	LQMD-FLVH	0	4	1	0	0	LQMDFLVH	Sequence	0.040	32341.93	41.00
17	HLA-B0702	QMDFLVHPAA	QMFLVHPAA	0	0	0	2	1	QMDFLVHPAA	Sequence	0.040	32433.05	41.00
16	HLA-B0702	LQMDFLVHP	LQMDFLVHP	0	0	0	0	0	LQMDFLVHP	Sequence	0.040	32451.99	41.00
12	HLA-B0702	PRKVLQMDF	PRKVLQMDF	0	0	0	0	0	PRKVLQMDF	Sequence	0.038	33120.31	44.00
26	HLA-B0702	ALSLDEPF	A-LSLDEPF	0	1	1	0	0	ALSLDEPF	Sequence	0.035	34356.94	49.00
6	HLA-B0702	DIYYILPRK	DIYYILPRK	0	0	0	0	0	DIYYILPRK	Sequence	0.033	35011.03	55.00
13	HLA-B0702	RKVLQMDF	R-KVLQMDF	0	1	1	0	0	RKVLQMDF	Sequence	0.031	35657.88	55.00
8	HLA-B0702	YYILPRKV	YYILPRK-V	0	7	1	0	0	YYILPRKV	Sequence	0.030	36002.52	60.00
12	HLA-B0702	PRKVLQMDFL	RKVLQMDFL	1	0	0	0	0	RKVLQMDFL	Sequence	0.030	36251.88	60.00
24	HLA-B0702	PAALSLDEP	PAALSLDEP	0	0	0	0	0	PAALSLDEP	Sequence	0.028	36735.97	60.00
0	HLA-B0702	QVLPDADIYY	QVLPADIYY	0	0	0	4	1	QVLPDADIYY	Sequence	0.027	37232.58	65.00
18	HLA-B0702	MDFLVHPA	MDFLVH-PA	0	6	1	0	0	MDFLVHPA	Sequence	0.027	37438.59	65.00
10	HLA-B0702	ILPRKVLQ	-ILPRKVLQ	0	0	1	0	0	ILPRKVLQ	Sequence	0.025	38129.06	70.00
5	HLA-B0702	ADIYYILPRK	AIYYILPRK	0	0	0	1	1	ADIYYILPRK	Sequence	0.024	38446.38	70.00
26	HLA-B0702	ALSLDEPFIQ	ALSLDPFIQ	0	0	0	5	1	ALSLDEPFIQ	Sequence	0.024	38471.78	70.00
25	HLA-B0702	AALSLDEP	AALSLDEP-	0	8	1	0	0	AALSLDEP	Sequence	0.024	38525.08	70.00
0	HLA-B0702	QVLPDADI	QVLP-DADI	0	4	1	0	0	QVLPDADI	Sequence	0.024	38629.02	75.00
28	HLA-B0702	SLDEPFIQ	S-LDEPFIQ	0	1	1	0	0	SLDEPFIQ	Sequence	0.023	38915.12	75.00
15	HLA-B0702	VLQMDFLVHP	VLQMDFLVH	0	0	0	0	0	VLQMDFLVH	Sequence	0.022	39321.44	75.00
15	HLA-B0702	VLQMDFLV	V-LQMDFLV	0	1	1	0	0	VLQMDFLV	Sequence	0.022	39325.27	75.00
19	HLA-B0702	DFLVHPAA	-DFLVHPAA	0	0	1	0	0	DFLVHPAA	Sequence	0.021	39917.71	80.00
4	HLA-B0702	DADIYYILP	DADIYYILP	0	0	0	0	0	DADIYYILP	Sequence	0.020	40190.75	80.00
1	HLA-B0702	VLPDADIY	V-LPDADIY	0	1	1	0	0	VLPDADIY	Sequence	0.020	40286.09	85.00
7	HLA-B0702	IYYILPRK	-IYYILPRK	0	0	1	0	0	IYYILPRK	Sequence	0.020	40484.03	85.00
6	HLA-B0702	DIYYILPRKV	DIYYILRKV	0	0	0	6	1	DIYYILPRKV	Sequence	0.019	40673.26	85.00
19	HLA-B0702	DFLVHPAALS	DFLVHPAAL	0	0	0	0	0	DFLVHPAAL	Sequence	0.019	40856.29	85.00
17	HLA-B0702	QMDFLVHP	QMDFLVHP-	0	8	1	0	0	QMDFLVHP	Sequence	0.019	40866.04	85.00
4	HLA-B0702	DADIYYILPR	ADIYYILPR	1	0	0	0	0	ADIYYILPR	Sequence	0.018	41030.40	85.00
4	HLA-B0702	DADIYYIL	-DADIYYIL	0	0	1	0	0	DADIYYIL	Sequence	0.018	41191.86	90.00
6	HLA-B0702	DIYYILPR	DIYYI-LPR	0	5	1	0	0	DIYYILPR	Sequence	0.018	41254.31	90.00
5	HLA-B0702	ADIYYILP	ADIYYILP-	0	8	1	0	0	ADIYYILP	Sequence	0.016	41874.46	90.00
27	HLA-B0702	LSLDEPFI	LSLD-EPFI	0	4	1	0	0	LSLDEPFI	Sequence	0.016	42251.75	95.00
3	HLA-B0702	PDADIYYILP	PDADIYYIL	0	0	0	0	0	PDADIYYIL	Sequence	0.015	42729.39	95.00

Protein Sequence. Allele HLA-B0702. Number of high binders 2. Number of weak binders 2. Number of peptides 84

Link to Allele Frequencies in Worldwide Populations HLA-B0702

Rank Threshold for Strong binding peptides 0.500
Rank Threshold for Weak binding peptides 2.000

----

pos	HLA	peptide	Core	Offset	I_pos	I_len	D_pos	D_len	iCore	Identity	1-log50k(aff)	Affinity(nM)	%Rank	BindLeve
9	HLA-B0801	YILPRKVL	YILPRK-VL	0	6	1	0	0	YILPRKVL	Sequence	0.655	41.88	0.12	<= SB
20	HLA-B0801	FLVHPAAL	FL-VHPAAL	0	2	1	0	0	FLVHPAAL	Sequence	0.642	48.28	0.15	<= SB
11	HLA-B0801	LPRKVLQM	LPR-KVLQM	0	3	1	0	0	LPRKVLQM	Sequence	0.630	54.98	0.17	<= SB
9	HLA-B0801	YILPRKVLQM	YILRKVLQM	0	0	0	3	1	YILPRKVLQM	Sequence	0.482	270.34	0.60	<= WB
18	HLA-B0801	MDFLVHPAAL	MFLVHPAAL	0	0	0	1	1	MDFLVHPAAL	Sequence	0.432	469.11	0.90	<= WB
19	HLA-B0801	DFLVHPAAL	DFLVHPAAL	0	0	0	0	0	DFLVHPAAL	Sequence	0.311	1727.47	2.50	
2	HLA-B0801	LPDADIYYIL	LPADIYYIL	0	0	0	2	1	LPDADIYYIL	Sequence	0.281	2394.72	3.00	
20	HLA-B0801	FLVHPAALSL	FLHPAALSL	0	0	0	2	1	FLVHPAALSL	Sequence	0.280	2418.75	3.00	
10	HLA-B0801	ILPRKVLQM	ILPRKVLQM	0	0	0	0	0	ILPRKVLQM	Sequence	0.279	2443.45	3.00	
11	HLA-B0801	LPRKVLQMDF	LPRKVLQMF	0	0	0	8	1	LPRKVLQMDF	Sequence	0.253	3228.66	3.50	
21	HLA-B0801	LVHPAALSL	LVHPAALSL	0	0	0	0	0	LVHPAALSL	Sequence	0.234	3986.75	4.00	
16	HLA-B0801	LQMDFLVHPA	LQMFLVHPA	0	0	0	3	1	LQMDFLVHPA	Sequence	0.191	6304.53	6.00	
8	HLA-B0801	YYILPRKVL	YYILPRKVL	0	0	0	0	0	YYILPRKVL	Sequence	0.173	7653.39	7.50	
7	HLA-B0801	IYYILPRKVL	YYILPRKVL	1	0	0	0	0	YYILPRKVL	Sequence	0.160	8851.89	8.50	
9	HLA-B0801	YILPRKVLQ	YILPRKVLQ	0	0	0	0	0	YILPRKVLQ	Sequence	0.143	10642.20	11.00	
17	HLA-B0801	QMDFLVHPA	QMDFLVHPA	0	0	0	0	0	QMDFLVHPA	Sequence	0.128	12480.00	13.00	
17	HLA-B0801	QMDFLVHPAA	QMFLVHPAA	0	0	0	2	1	QMDFLVHPAA	Sequence	0.125	12921.90	13.00	
19	HLA-B0801	DFLVHPAALS	DFLVHPAAL	0	0	0	0	0	DFLVHPAAL	Sequence	0.125	12939.80	13.00	
4	HLA-B0801	DADIYYIL	DA-DIYYIL	0	2	1	0	0	DADIYYIL	Sequence	0.123	13240.62	14.00	
15	HLA-B0801	VLQMDFLV	VLQMDFL-V	0	7	1	0	0	VLQMDFLV	Sequence	0.118	13973.17	15.00	
21	HLA-B0801	LVHPAALSLD	LVHPAALSL	0	0	0	0	0	LVHPAALSL	Sequence	0.118	13987.54	15.00	
11	HLA-B0801	LPRKVLQMD	LPRKVLQMD	0	0	0	0	0	LPRKVLQMD	Sequence	0.117	14140.01	15.00	
8	HLA-B0801	YYILPRKVLQ	YYILPRKVL	0	0	0	0	0	YYILPRKVL	Sequence	0.105	16012.52	17.00	
10	HLA-B0801	ILPRKVLQMD	ILPRKVLQM	0	0	0	0	0	ILPRKVLQM	Sequence	0.100	16870.85	19.00	
18	HLA-B0801	MDFLVHPAA	MDFLVHPAA	0	0	0	0	0	MDFLVHPAA	Sequence	0.099	17180.32	19.00	
13	HLA-B0801	RKVLQMDFL	RKVLQMDFL	0	0	0	0	0	RKVLQMDFL	Sequence	0.091	18738.14	22.00	
16	HLA-B0801	LQMDFLVHP	LQMDFLVHP	0	0	0	0	0	LQMDFLVHP	Sequence	0.088	19369.75	23.00	
25	HLA-B0801	AALSLDEPF	AALSLDEPF	0	0	0	0	0	AALSLDEPF	Sequence	0.088	19377.72	23.00	
22	HLA-B0801	VHPAALSL	-VHPAALSL	0	0	1	0	0	VHPAALSL	Sequence	0.084	20139.98	24.00	
12	HLA-B0801	PRKVLQMDFL	PRKVLQMFL	0	0	0	7	1	PRKVLQMDFL	Sequence	0.084	20141.07	24.00	
15	HLA-B0801	VLQMDFLVH	VLQMDFLVH	0	0	0	0	0	VLQMDFLVH	Sequence	0.082	20501.44	25.00	
2	HLA-B0801	LPDADIYYI	LPDADIYYI	0	0	0	0	0	LPDADIYYI	Sequence	0.082	20609.30	25.00	
14	HLA-B0801	KVLQMDFLV	KVLQMDFLV	0	0	0	0	0	KVLQMDFLV	Sequence	0.080	21109.41	26.00	
14	HLA-B0801	KVLQMDFL	KVLQMDF-L	0	7	1	0	0	KVLQMDFL	Sequence	0.079	21203.95	26.00	
19	HLA-B0801	DFLVHPAA	DFLVHP-AA	0	6	1	0	0	DFLVHPAA	Sequence	0.076	22076.00	28.00	
23	HLA-B0801	HPAALSLDE	HPAALSLDE	0	0	0	0	0	HPAALSLDE	Sequence	0.075	22253.71	28.00	
15	HLA-B0801	VLQMDFLVHP	VLQMDFLVP	0	0	0	8	1	VLQMDFLVHP	Sequence	0.072	23039.70	30.00	
12	HLA-B0801	PRKVLQMDF	PRKVLQMDF	0	0	0	0	0	PRKVLQMDF	Sequence	0.071	23199.79	30.00	
3	HLA-B0801	PDADIYYIL	PDADIYYIL	0	0	0	0	0	PDADIYYIL	Sequence	0.070	23378.96	31.00	
18	HLA-B0801	MDFLVHPA	MDFLVH-PA	0	6	1	0	0	MDFLVHPA	Sequence	0.069	23743.49	32.00	
13	HLA-B0801	RKVLQMDFLV	RVLQMDFLV	0	0	0	1	1	RKVLQMDFLV	Sequence	0.068	23898.13	32.00	
13	HLA-B0801	RKVLQMDF	-RKVLQMDF	0	0	1	0	0	RKVLQMDF	Sequence	0.057	26883.40	40.00	
16	HLA-B0801	LQMDFLVH	LQM-DFLVH	0	3	1	0	0	LQMDFLVH	Sequence	0.053	28292.59	44.00	
26	HLA-B0801	ALSLDEPFI	ALSLDEPFI	0	0	0	0	0	ALSLDEPFI	Sequence	0.052	28630.08	45.00	
24	HLA-B0801	PAALSLDEPF	AALSLDEPF	1	0	0	0	0	AALSLDEPF	Sequence	0.051	28700.18	45.00	

14	HLA-B0801	KVLQMDFLVH	KVLQMFLVH	0	0	0	5	1	KVLQMDFLVH	Sequence	0.051	28839.63	46.00
20	HLA-B0801	FLVHPAALS	FLVHPAALS	0	0	0	0	0	FLVHPAALS	Sequence	0.049	29429.10	48.00
26	HLA-B0801	ALSLDEPF	-ALSLDEPF	0	0	1	0	0	ALSLDEPF	Sequence	0.049	29480.08	48.00
1	HLA-B0801	VLPDADIYYI	LPDADIYYI	1	0	0	0	0	LPDADIYYI	Sequence	0.049	29529.57	48.00
0	HLA-B0801	QVLPDADIY	QVLPDADIY	0	0	0	0	0	QVLPDADIY	Sequence	0.045	30650.69	55.00
25	HLA-B0801	AALSLDEPFI	AALSLDEPI	0	0	0	8	1	AALSLDEPFI	Sequence	0.044	31091.24	55.00
22	HLA-B0801	VHPAALSLDE	HPAALSLDE	1	0	0	0	0	HPAALSLDE	Sequence	0.043	31233.21	55.00
10	HLA-B0801	ILPRKVLQ	IL-PRKVLQ	0	2	1	0	0	ILPRKVLQ	Sequence	0.042	31641.71	55.00
27	HLA-B0801	LSLDEPFIQ	LSLDEPFIQ	0	0	0	0	0	LSLDEPFIQ	Sequence	0.041	31927.84	60.00
8	HLA-B0801	YYILPRKV	Y-YILPRKV	0	1	1	0	0	YYILPRKV	Sequence	0.041	32224.92	60.00
17	HLA-B0801	QMDFLVHP	QMDFLVHP-	0	8	1	0	0	QMDFLVHP	Sequence	0.040	32308.36	60.00
4	HLA-B0801	DADIYYILP	DADIYYILP	0	0	0	0	0	DADIYYILP	Sequence	0.040	32441.11	60.00
23	HLA-B0801	HPAALSLDEP	HPAALSLEP	0	0	0	7	1	HPAALSLDEP	Sequence	0.039	32685.28	60.00
6	HLA-B0801	DIYYILPRKV	DIYYILPRV	0	0	0	8	1	DIYYILPRKV	Sequence	0.037	33560.77	65.00
7	HLA-B0801	IYYILPRKV	IYYILPRKV	0	0	0	0	0	IYYILPRKV	Sequence	0.036	33716.89	65.00
3	HLA-B0801	PDADIYYILP	PDADIYYIL	0	0	0	0	0	PDADIYYIL	Sequence	0.035	34055.32	65.00
27	HLA-B0801	LSLDEPFI	LSLDEP-FI	0	6	1	0	0	LSLDEPFI	Sequence	0.035	34085.15	65.00
21	HLA-B0801	LVHPAALS	LVHPAALS-	0	8	1	0	0	LVHPAALS	Sequence	0.034	34560.12	70.00
12	HLA-B0801	PRKVLQMD	-PRKVLQMD	0	0	1	0	0	PRKVLQMD	Sequence	0.034	34778.07	70.00
4	HLA-B0801	DADIYYILPR	DAIYYILPR	0	0	0	2	1	DADIYYILPR	Sequence	0.031	35738.59	75.00
1	HLA-B0801	VLPDADIYY	VLPDADIYY	0	0	0	0	0	VLPDADIYY	Sequence	0.030	36169.21	75.00
5	HLA-B0801	ADIYYILPR	ADIYYILPR	0	0	0	0	0	ADIYYILPR	Sequence	0.030	36208.38	75.00
0	HLA-B0801	QVLPDADI	QVLP-DADI	0	4	1	0	0	QVLPDADI	Sequence	0.029	36456.44	80.00
6	HLA-B0801	DIYYILPRK	DIYYILPRK	0	0	0	0	0	DIYYILPRK	Sequence	0.029	36593.96	80.00
6	HLA-B0801	DIYYILPR	DIY-YILPR	0	3	1	0	0	DIYYILPR	Sequence	0.029	36637.55	80.00
23	HLA-B0801	HPAALSLD	HPAALSLD-	0	8	1	0	0	HPAALSLD	Sequence	0.028	36905.30	80.00
26	HLA-B0801	ALSLDEPFIQ	LSLDEPFIQ	1	0	0	0	0	LSLDEPFIQ	Sequence	0.027	37220.09	80.00
0	HLA-B0801	QVLPDADIYY	QVLPDADIY	0	0	0	8	1	QVLPDADIYY	Sequence	0.027	37518.88	85.00
2	HLA-B0801	LPDADIYY	LP-DADIYY	0	2	1	0	0	LPDADIYY	Sequence	0.027	37532.70	85.00
3	HLA-B0801	PDADIYYI	-PDADIYYI	0	0	1	0	0	PDADIYYI	Sequence	0.024	38500.49	85.00
24	HLA-B0801	PAALSLDEP	PAALSLDEP	0	0	0	0	0	PAALSLDEP	Sequence	0.023	39171.12	90.00
22	HLA-B0801	VHPAALSLD	VHPAALSLD	0	0	0	0	0	VHPAALSLD	Sequence	0.022	39225.39	90.00
24	HLA-B0801	PAALSLDE	-PAALSLDE	0	0	1	0	0	PAALSLDE	Sequence	0.022	39375.08	90.00
25	HLA-B0801	AALSLDEP	AALSLDEP-	0	8	1	0	0	AALSLDEP	Sequence	0.021	39801.27	90.00
5	HLA-B0801	ADIYYILP	ADIYYILP-	0	8	1	0	0	ADIYYILP	Sequence	0.021	39840.48	90.00
1	HLA-B0801	VLPDADIY	-VLPDADIY	0	0	1	0	0	VLPDADIY	Sequence	0.021	39929.38	95.00
28	HLA-B0801	SLDEPFIQ	SLDEPF-IQ	0	6	1	0	0	SLDEPFIQ	Sequence	0.020	40182.48	95.00
5	HLA-B0801	ADIYYILPRK	ADIYYILPK	0	0	0	8	1	ADIYYILPRK	Sequence	0.019	40816.10	95.00
7	HLA-B0801	IYYILPRK	-IYYILPRK	0	0	1	0	0	IYYILPRK	Sequence	0.016	42277.34	99.00

Protein Sequence. Allele HLA-B0801. Number of high binders 3. Number of weak binders 2. Number of peptides 84

Link to Allele Frequencies in Worldwide Populations HLA-B0801

Rank Threshold for Strong binding peptides 0.500
Rank Threshold for Weak binding peptides 2.000

----

pos	HLA	peptide	Core	Offset	I_pos	I_len	D_pos	D_len	iCore	Identity	1-log50k(aff)	Affinity(nM)	%Rank	BindLeve
13	HLA-B2705	RKVLQMDFLV	RKLQMDFLV	0	0	0	2	1	RKVLQMDFLV	Sequence	0.292	2115.23	3.50	
16	HLA-B2705	LQMDFLVHPA	LQMFLVHPA	0	0	0	3	1	LQMDFLVHPA	Sequence	0.279	2440.65	4.00	
13	HLA-B2705	RKVLQMDFL	RKVLQMDFL	0	0	0	0	0	RKVLQMDFL	Sequence	0.277	2494.91	4.00	
11	HLA-B2705	LPRKVLQMDF	LRKVLQMDF	0	0	0	1	1	LPRKVLQMDF	Sequence	0.228	4226.02	5.00	
12	HLA-B2705	PRKVLQMDFL	PRKVLQMFL	0	0	0	7	1	PRKVLQMDFL	Sequence	0.163	8573.44	8.00	
21	HLA-B2705	LVHPAALSL	LVHPAALSL	0	0	0	0	0	LVHPAALSL	Sequence	0.146	10290.83	9.50	
12	HLA-B2705	PRKVLQMDF	PRKVLQMDF	0	0	0	0	0	PRKVLQMDF	Sequence	0.146	10350.13	9.50	
16	HLA-B2705	LQMDFLVHP	LQMDFLVHP	0	0	0	0	0	LQMDFLVHP	Sequence	0.141	10875.82	9.50	
14	HLA-B2705	KVLQMDFLV	KVLQMDFLV	0	0	0	0	0	KVLQMDFLV	Sequence	0.135	11557.43	11.00	
20	HLA-B2705	FLVHPAALSL	FLHPAALSL	0	0	0	2	1	FLVHPAALSL	Sequence	0.118	13951.72	12.00	
13	HLA-B2705	RKVLQMDF	-RKVLQMDF	0	0	1	0	0	RKVLQMDF	Sequence	0.118	13964.56	12.00	
25	HLA-B2705	AALSLDEPF	AALSLDEPF	0	0	0	0	0	AALSLDEPF	Sequence	0.111	15044.41	13.00	
17	HLA-B2705	QMDFLVHPA	QMDFLVHPA	0	0	0	0	0	QMDFLVHPA	Sequence	0.106	15814.86	14.00	
5	HLA-B2705	ADIYYILPR	ADIYYILPR	0	0	0	0	0	ADIYYILPR	Sequence	0.095	17957.28	16.00	
8	HLA-B2705	YYILPRKVL	YYILPRKVL	0	0	0	0	0	YYILPRKVL	Sequence	0.092	18458.43	17.00	
16	HLA-B2705	LQMDFLVH	LQMDFLVH-	0	8	1	0	0	LQMDFLVH	Sequence	0.092	18497.22	17.00	
9	HLA-B2705	YILPRKVLQM	YILRKVLQM	0	0	0	3	1	YILPRKVLQM	Sequence	0.092	18552.73	17.00	
6	HLA-B2705	DIYYILPRK	DIYYILPRK	0	0	0	0	0	DIYYILPRK	Sequence	0.081	20737.92	20.00	
7	HLA-B2705	IYYILPRKV	IYYILPRKV	0	0	0	0	0	IYYILPRKV	Sequence	0.081	20876.15	20.00	
10	HLA-B2705	ILPRKVLQM	ILPRKVLQM	0	0	0	0	0	ILPRKVLQM	Sequence	0.077	21667.33	21.00	
21	HLA-B2705	LVHPAALSLD	LVHPAALSL	0	0	0	0	0	LVHPAALSL	Sequence	0.077	21837.97	22.00	
5	HLA-B2705	ADIYYILPRK	AIYYILPRK	0	0	0	1	1	ADIYYILPRK	Sequence	0.076	22003.75	22.00	
14	HLA-B2705	KVLQMDFLVH	KVLQMDFLV	0	0	0	0	0	KVLQMDFLV	Sequence	0.070	23470.45	24.00	
20	HLA-B2705	FLVHPAALS	FLVHPAALS	0	0	0	0	0	FLVHPAALS	Sequence	0.066	24432.54	26.00	
7	HLA-B2705	IYYILPRKVL	IYYILPRKL	0	0	0	8	1	IYYILPRKVL	Sequence	0.061	25742.22	29.00	
25	HLA-B2705	AALSLDEPFI	AALSLDEPF	0	0	0	0	0	AALSLDEPF	Sequence	0.059	26331.25	30.00	
17	HLA-B2705	QMDFLVHPAA	QMFLVHPAA	0	0	0	2	1	QMDFLVHPAA	Sequence	0.059	26464.63	30.00	
15	HLA-B2705	VLQMDFLVHP	VQMDFLVHP	0	0	0	1	1	VLQMDFLVHP	Sequence	0.058	26773.08	31.00	
9	HLA-B2705	YILPRKVLQ	YILPRKVLQ	0	0	0	0	0	YILPRKVLQ	Sequence	0.057	26943.96	31.00	
7	HLA-B2705	IYYILPRK	I-YYILPRK	0	1	1	0	0	IYYILPRK	Sequence	0.052	28556.15	35.00	
0	HLA-B2705	QVLPDADIY	QVLPDADIY	0	0	0	0	0	QVLPDADIY	Sequence	0.052	28604.07	35.00	
15	HLA-B2705	VLQMDFLVH	VLQMDFLVH	0	0	0	0	0	VLQMDFLVH	Sequence	0.051	28655.50	35.00	
26	HLA-B2705	ALSLDEPFI	ALSLDEPFI	0	0	0	0	0	ALSLDEPFI	Sequence	0.051	28702.66	36.00	
14	HLA-B2705	KVLQMDFL	KVLQMDF-L	0	7	1	0	0	KVLQMDFL	Sequence	0.050	29075.25	37.00	
22	HLA-B2705	VHPAALSL	V-HPAALSL	0	1	1	0	0	VHPAALSL	Sequence	0.047	30230.76	40.00	
2	HLA-B2705	LPDADIYYI	LPDADIYYI	0	0	0	0	0	LPDADIYYI	Sequence	0.046	30493.90	41.00	
4	HLA-B2705	DADIYYILPR	DAIYYILPR	0	0	0	2	1	DADIYYILPR	Sequence	0.046	30521.62	41.00	
1	HLA-B2705	VLPDADIYY	VLPDADIYY	0	0	0	0	0	VLPDADIYY	Sequence	0.046	30558.63	41.00	
8	HLA-B2705	YYILPRKVLQ	YYILPRKVL	0	0	0	0	0	YYILPRKVL	Sequence	0.045	30868.68	42.00	
17	HLA-B2705	QMDFLVHP	QMDFLVHP-	0	8	1	0	0	QMDFLVHP	Sequence	0.042	31615.36	44.00	

26	HLA-B2705	ALSLDEPF	A-LSLDEPF	0	1	1	0	0	ALSLDEPF	Sequence	0.042	31674.25	45.00	
24	HLA-B2705	PAALSLDEPF	AALSLDEPF	1	0	0	0	0	AALSLDEPF	Sequence	0.040	32424.27	47.00	
18	HLA-B2705	MDFLVHPAA	MDFLVHPAA	0	0	0	0	0	MDFLVHPAA	Sequence	0.040	32447.09	47.00	
8	HLA-B2705	YYILPRKV	Y-YILPRKV	0	1	1	0	0	YYILPRKV	Sequence	0.039	32628.39	48.00	
20	HLA-B2705	FLVHPAAL	FLVHPA-AL	0	6	1	0	0	FLVHPAAL	Sequence	0.038	33212.88	50.00	
6	HLA-B2705	DIYYILPRKV	DIYYILPRK	0	0	0	0	0	DIYYILPRK	Sequence	0.038	33284.11	50.00	
19	HLA-B2705	DFLVHPAAL	DFLVHPAAL	0	0	0	0	0	DFLVHPAAL	Sequence	0.037	33525.55	55.00	
0	HLA-B2705	QVLPDADIYY	QVLDADIYY	0	0	0	3	1	QVLPDADIYY	Sequence	0.037	33652.75	55.00	
12	HLA-B2705	PRKVLQMD	PRKVLQMD-	0	8	1	0	0	PRKVLQMD	Sequence	0.036	33847.39	55.00	
6	HLA-B2705	DIYYILPR	D-IYYILPR	0	1	1	0	0	DIYYILPR	Sequence	0.036	33922.18	55.00	
10	HLA-B2705	ILPRKVLQMD	ILPRKVLQM	0	0	0	0	0	ILPRKVLQM	Sequence	0.036	33990.15	55.00	
21	HLA-B2705	LVHPAALS	LVHPAALS-	0	8	1	0	0	LVHPAALS	Sequence	0.034	34655.62	60.00	
11	HLA-B2705	LPRKVLQMD	LPRKVLQMD	0	0	0	0	0	LPRKVLQMD	Sequence	0.034	34749.86	60.00	
27	HLA-B2705	LSLDEPFIQ	LSLDEPFIQ	0	0	0	0	0	LSLDEPFIQ	Sequence	0.033	34973.54	60.00	
23	HLA-B2705	HPAALSLDE	HPAALSLDE	0	0	0	0	0	HPAALSLDE	Sequence	0.031	35818.34	65.00	
9	HLA-B2705	YILPRKVL	Y-ILPRKVL	0	1	1	0	0	YILPRKVL	Sequence	0.031	35828.41	65.00	
18	HLA-B2705	MDFLVHPAAL	MDFLVHPAL	0	0	0	7	1	MDFLVHPAAL	Sequence	0.030	36041.11	65.00	
11	HLA-B2705	LPRKVLQM	LPRKV-LQM	0	5	1	0	0	LPRKVLQM	Sequence	0.030	36046.17	65.00	
15	HLA-B2705	VLQMDFLV	V-LQMDFLV	0	1	1	0	0	VLQMDFLV	Sequence	0.029	36644.29	65.00	
22	HLA-B2705	VHPAALSLD	VHPAALSLD	0	0	0	0	0	VHPAALSLD	Sequence	0.028	37016.48	70.00	
1	HLA-B2705	VLPDADIYYI	VLDADIYYI	0	0	0	2	1	VLPDADIYYI	Sequence	0.026	37561.13	70.00	
3	HLA-B2705	PDADIYYIL	PDADIYYIL	0	0	0	0	0	PDADIYYIL	Sequence	0.026	37836.05	75.00	
18	HLA-B2705	MDFLVHPA	-MDFLVHPA	0	0	1	0	0	MDFLVHPA	Sequence	0.026	37868.39	75.00	
0	HLA-B2705	QVLPDADI	-QVLPDADI	0	0	1	0	0	QVLPDADI	Sequence	0.025	38126.18	75.00	
2	HLA-B2705	LPDADIYYIL	LDADIYYIL	0	0	0	1	1	LPDADIYYIL	Sequence	0.025	38129.06	75.00	
4	HLA-B2705	DADIYYILP	DADIYYILP	0	0	0	0	0	DADIYYILP	Sequence	0.025	38162.07	75.00	
26	HLA-B2705	ALSLDEPFIQ	ALSLDEPFI	0	0	0	0	0	ALSLDEPFI	Sequence	0.024	38539.27	75.00	
27	HLA-B2705	LSLDEPFI	LSLDEPF-I	0	7	1	0	0	LSLDEPFI	Sequence	0.022	39226.25	80.00	
19	HLA-B2705	DFLVHPAALS	FLVHPAALS	1	0	0	0	0	FLVHPAALS	Sequence	0.020	40270.40	85.00	
25	HLA-B2705	AALSLDEP	AALSLDEP-	0	8	1	0	0	AALSLDEP	Sequence	0.020	40347.58	85.00	
28	HLA-B2705	SLDEPFIQ	SLDEPFIQ-	0	8	1	0	0	SLDEPFIQ	Sequence	0.019	40520.83	85.00	
2	HLA-B2705	LPDADIYY	LPDADIY-Y	0	7	1	0	0	LPDADIYY	Sequence	0.019	40744.17	85.00	
5	HLA-B2705	ADIYYILP	ADIYYILP-	0	8	1	0	0	ADIYYILP	Sequence	0.019	40758.28	90.00	
23	HLA-B2705	HPAALSLD	HPAALSLD-	0	8	1	0	0	HPAALSLD	Sequence	0.017	41474.95	90.00	
10	HLA-B2705	ILPRKVLQ	ILPRKVLQ-	0	8	1	0	0	ILPRKVLQ	Sequence	0.017	41483.03	90.00	
4	HLA-B2705	DADIYYIL	D-ADIYYIL	0	1	1	0	0	DADIYYIL	Sequence	0.017	41504.14	90.00	
1	HLA-B2705	VLPDADIY	VLPDADI-Y	0	7	1	0	0	VLPDADIY	Sequence	0.017	41519.86	90.00	
23	HLA-B2705	HPAALSLDEP	HPAALSLEP	0	0	0	7	1	HPAALSLDEP	Sequence	0.017	41563.45	90.00	
24	HLA-B2705	PAALSLDEP	PAALSLDEP	0	0	0	0	0	PAALSLDEP	Sequence	0.016	42065.64	95.00	
22	HLA-B2705	VHPAALSLDE	VHPAALSLD	0	0	0	0	0	VHPAALSLD	Sequence	0.014	42949.11	95.00	
19	HLA-B2705	DFLVHPAA	D-FLVHPAA	0	1	1	0	0	DFLVHPAA	Sequence	0.013	43568.80	99.00	
3	HLA-B2705	PDADIYYI	-PDADIYYI	0	0	1	0	0	PDADIYYI	Sequence	0.012	43960.41	99.00	
3	HLA-B2705	PDADIYYILP	PDADIYYIL	0	0	0	0	0	PDADIYYIL	Sequence	0.011	44416.51	99.00	
24	HLA-B2705	PAALSLDE	PAALSLDE-	0	8	1	0	0	PAALSLDE	Sequence	0.007	46139.76	99.00	

Protein Sequence. Allele HLA-B2705. Number of high binders 0. Number of weak binders 0. Number of peptides 84

Link to Allele Frequencies in Worldwide Populations HLA-B2705

Rank Threshold for Strong binding peptides 0.500
Rank Threshold for Weak binding peptides 2.000

---

pos	HLA	peptide	Core	Offset	I_pos	I_len	D_pos	D_len	iCore	Identity	1-log50k(aff)	Affinity(nM)	%Rank	BindLevel
21	HLA-B3901	LVHPAALSL	LVHPAALSL	0	0	0	0	0	LVHPAALSL	Sequence	0.320	1563.85	1.00	<= WB
8	HLA-B3901	YYILPRKVL	YYILPRKVL	0	0	0	0	0	YYILPRKVL	Sequence	0.268	2755.17	1.40	<= WB
17	HLA-B3901	QMDFLVHPA	QMDFLVHPA	0	0	0	0	0	QMDFLVHPA	Sequence	0.248	3400.91	1.70	<= WB
2	HLA-B3901	LPDADIYYI	LPDADIYYI	0	0	0	0	0	LPDADIYYI	Sequence	0.192	6251.62	3.00	
20	HLA-B3901	FLVHPAAL	F-LVHPAAL	0	1	1	0	0	FLVHPAAL	Sequence	0.181	7029.96	3.00	
19	HLA-B3901	DFLVHPAAL	DFLVHPAAL	0	0	0	0	0	DFLVHPAAL	Sequence	0.167	8194.60	3.50	
22	HLA-B3901	VHPAALSL	VH-PAALSL	0	2	1	0	0	VHPAALSL	Sequence	0.155	9300.99	4.00	
2	HLA-B3901	LPDADIYYIL	LPDAIYYIL	0	0	0	4	1	LPDADIYYIL	Sequence	0.135	11597.26	5.00	
13	HLA-B3901	RKVLQMDFL	RKVLQMDFL	0	0	0	0	0	RKVLQMDFL	Sequence	0.131	12065.60	5.00	
20	HLA-B3901	FLVHPAALSL	FLHPAALSL	0	0	0	2	1	FLVHPAALSL	Sequence	0.127	12635.15	5.50	
23	HLA-B3901	HPAALSLDE	HPAALSLDE	0	0	0	0	0	HPAALSLDE	Sequence	0.126	12815.94	5.50	
3	HLA-B3901	PDADIYYIL	PDADIYYIL	0	0	0	0	0	PDADIYYIL	Sequence	0.104	16311.90	7.50	
18	HLA-B3901	MDFLVHPAA	MDFLVHPAA	0	0	0	0	0	MDFLVHPAA	Sequence	0.100	16898.26	8.00	
18	HLA-B3901	MDFLVHPAAL	MDFLVPAAL	0	0	0	5	1	MDFLVHPAAL	Sequence	0.079	21261.16	11.00	
16	HLA-B3901	LQMDFLVHP	LQMDFLVHP	0	0	0	0	0	LQMDFLVHP	Sequence	0.075	22103.01	12.00	
25	HLA-B3901	AALSLDEPF	AALSLDEPF	0	0	0	0	0	AALSLDEPF	Sequence	0.075	22291.79	12.00	
16	HLA-B3901	LQMDFLVHPA	LQMDLVHPA	0	0	0	4	1	LQMDFLVHPA	Sequence	0.070	23348.87	14.00	
9	HLA-B3901	YILPRKVL	YILPRK-VL	0	6	1	0	0	YILPRKVL	Sequence	0.062	25563.20	16.00	
9	HLA-B3901	YILPRKVLQ	YILPRKVLQ	0	0	0	0	0	YILPRKVLQ	Sequence	0.061	25960.38	17.00	
14	HLA-B3901	KVLQMDFLV	KVLQMDFLV	0	0	0	0	0	KVLQMDFLV	Sequence	0.059	26344.63	17.00	
4	HLA-B3901	DADIYYIL	DAD-IYYIL	0	3	1	0	0	DADIYYIL	Sequence	0.059	26374.59	17.00	
17	HLA-B3901	QMDFLVHPAA	QMDFLVHPA	0	0	0	0	0	QMDFLVHPA	Sequence	0.057	26918.62	18.00	
20	HLA-B3901	FLVHPAALS	FLVHPAALS	0	0	0	0	0	FLVHPAALS	Sequence	0.055	27428.12	19.00	
21	HLA-B3901	LVHPAALSLD	LVHPAALSL	0	0	0	0	0	LVHPAALSL	Sequence	0.055	27601.08	19.00	
0	HLA-B3901	QVLPDADIY	QVLPDADIY	0	0	0	0	0	QVLPDADIY	Sequence	0.055	27653.69	20.00	
18	HLA-B3901	MDFLVHPA	-MDFLVHPA	0	0	1	0	0	MDFLVHPA	Sequence	0.055	27653.99	20.00	
17	HLA-B3901	QMDFLVHP	QMDFLVHP-	0	8	1	0	0	QMDFLVHP	Sequence	0.054	27791.68	20.00	
5	HLA-B3901	ADIYYILPR	ADIYYILPR	0	0	0	0	0	ADIYYILPR	Sequence	0.052	28415.60	21.00	
22	HLA-B3901	VHPAALSLD	VHPAALSLD	0	0	0	0	0	VHPAALSLD	Sequence	0.048	29676.90	24.00	
15	HLA-B3901	VLQMDFLVH	VLQMDFLVH	0	0	0	0	0	VLQMDFLVH	Sequence	0.047	30039.04	24.00	
27	HLA-B3901	LSLDEPFIQ	LSLDEPFIQ	0	0	0	0	0	LSLDEPFIQ	Sequence	0.046	30390.15	25.00	
4	HLA-B3901	DADIYYILP	DADIYYILP	0	0	0	0	0	DADIYYILP	Sequence	0.045	30802.95	26.00	
3	HLA-B3901	PDADIYYI	-PDADIYYI	0	0	1	0	0	PDADIYYI	Sequence	0.045	30892.73	26.00	
8	HLA-B3901	YYILPRKVLQ	YYILPRKVL	0	0	0	0	0	YYILPRKVL	Sequence	0.044	31128.62	27.00	
26	HLA-B3901	ALSLDEPFI	ALSLDEPFI	0	0	0	0	0	ALSLDEPFI	Sequence	0.043	31405.01	28.00	

7	HLA-B3901	IYYILPRKVL	YYILPRKVL	1	0	0	0	0	YYILPRKVL	Sequence	0.042	31596.54	28.00
6	HLA-B3901	DIYYILPRK	DIYYILPRK	0	0	0	0	0	DIYYILPRK	Sequence	0.039	32814.26	32.00
23	HLA-B3901	HPAALSLD	HPAALS-LD	0	6	1	0	0	HPAALSLD	Sequence	0.037	33459.23	34.00
7	HLA-B3901	IYYILPRKV	IYYILPRKV	0	0	0	0	0	IYYILPRKV	Sequence	0.037	33609.45	34.00
10	HLA-B3901	ILPRKVLQM	ILPRKVLQM	0	0	0	0	0	ILPRKVLQM	Sequence	0.037	33618.91	34.00
13	HLA-B3901	RKVLQMDFLV	RKVLQMDFL	0	0	0	0	0	RKVLQMDFL	Sequence	0.036	34022.17	36.00
16	HLA-B3901	LQMDFLVH	LQMD-FLVH	0	4	1	0	0	LQMDFLVH	Sequence	0.035	34232.27	37.00
14	HLA-B3901	KVLQMDFL	-KVLQMDFL	0	0	1	0	0	KVLQMDFL	Sequence	0.034	34444.77	38.00
1	HLA-B3901	VLPDADIYYI	LPDADIYYI	1	0	0	0	0	LPDADIYYI	Sequence	0.034	34614.39	38.00
23	HLA-B3901	HPAALSLDEP	HPAALSLEP	0	0	0	7	1	HPAALSLDEP	Sequence	0.033	34874.15	39.00
12	HLA-B3901	PRKVLQMDFL	RKVLQMDFL	1	0	0	0	0	RKVLQMDFL	Sequence	0.033	35002.67	40.00
9	HLA-B3901	YILPRKVLQM	YILPRVLQM	0	0	0	5	1	YILPRKVLQM	Sequence	0.033	35036.41	40.00
19	HLA-B3901	DFLVHPAALS	DFLVHPAAL	0	0	0	0	0	DFLVHPAAL	Sequence	0.033	35137.02	40.00
11	HLA-B3901	LPRKVLQMD	LPRKVLQMD	0	0	0	0	0	LPRKVLQMD	Sequence	0.032	35184.18	41.00
12	HLA-B3901	PRKVLQMDF	PRKVLQMDF	0	0	0	0	0	PRKVLQMDF	Sequence	0.031	35794.32	43.00
28	HLA-B3901	SLDEPFIQ	SLDE-PFIQ	0	4	1	0	0	SLDEPFIQ	Sequence	0.030	36289.96	46.00
1	HLA-B3901	VLPDADIYY	VLPDADIYY	0	0	0	0	0	VLPDADIYY	Sequence	0.028	36820.75	49.00
13	HLA-B3901	RKVLQMDF	RKV-LQMDF	0	3	1	0	0	RKVLQMDF	Sequence	0.028	36905.30	49.00
6	HLA-B3901	DIYYILPR	D-IYYILPR	0	1	1	0	0	DIYYILPR	Sequence	0.028	37062.96	50.00
14	HLA-B3901	KVLQMDFLVH	KVLQMDFLV	0	0	0	0	0	KVLQMDFLV	Sequence	0.028	37077.81	50.00
2	HLA-B3901	LPDADIYY	LPDADIY-Y	0	7	1	0	0	LPDADIYY	Sequence	0.027	37184.66	55.00
26	HLA-B3901	ALSLDEPF	-ALSLDEPF	0	0	1	0	0	ALSLDEPF	Sequence	0.027	37381.53	55.00
22	HLA-B3901	VHPAALSLDE	HPAALSLDE	1	0	0	0	0	HPAALSLDE	Sequence	0.026	37744.85	55.00
15	HLA-B3901	VLQMDFLVHP	VLQMDFLVH	0	0	0	0	0	VLQMDFLVH	Sequence	0.025	38157.94	60.00
4	HLA-B3901	DADIYYILPR	DAIYYILPR	0	0	0	2	1	DADIYYILPR	Sequence	0.024	38503.42	60.00
24	HLA-B3901	PAALSLDEP	PAALSLDEP	0	0	0	0	0	PAALSLDEP	Sequence	0.024	38697.20	65.00
11	HLA-B3901	LPRKVLQM	LP-RKVLQM	0	2	1	0	0	LPRKVLQM	Sequence	0.023	38860.85	65.00
8	HLA-B3901	YYILPRKV	YYILPR-KV	0	6	1	0	0	YYILPRKV	Sequence	0.023	38934.91	65.00
0	HLA-B3901	QVLPDADIYY	QVLPDADIY	0	0	0	0	0	QVLPDADIY	Sequence	0.023	39012.92	65.00
15	HLA-B3901	VLQMDFLV	-VLQMDFLV	0	0	1	0	0	VLQMDFLV	Sequence	0.022	39265.74	65.00
3	HLA-B3901	PDADIYYILP	PDADIYYIL	0	0	0	0	0	PDADIYYIL	Sequence	0.022	39577.96	70.00
25	HLA-B3901	AALSLDEPFI	AALSLDEPI	0	0	0	8	1	AALSLDEPFI	Sequence	0.021	39730.71	70.00
6	HLA-B3901	DIYYILPRKV	DIYYILPRV	0	0	0	8	1	DIYYILPRKV	Sequence	0.021	39781.45	70.00
24	HLA-B3901	PAALSLDEPF	AALSLDEPF	1	0	0	0	0	AALSLDEPF	Sequence	0.021	39804.29	70.00
0	HLA-B3901	QVLPDADI	-QVLPDADI	0	0	1	0	0	QVLPDADI	Sequence	0.021	39968.71	75.00
5	HLA-B3901	ADIYYILPRK	ADIYYILPR	0	0	0	0	0	ADIYYILPR	Sequence	0.021	39990.76	75.00
21	HLA-B3901	LVHPAALS	LVHPAALS-	0	8	1	0	0	LVHPAALS	Sequence	0.020	40162.92	75.00
27	HLA-B3901	LSLDEPFI	LSLDEPF-I	0	7	1	0	0	LSLDEPFI	Sequence	0.020	40487.53	75.00
11	HLA-B3901	LPRKVLQMDF	LPRKVLQMF	0	0	0	8	1	LPRKVLQMDF	Sequence	0.019	40504.19	75.00
19	HLA-B3901	DFLVHPAA	D-FLVHPAA	0	1	1	0	0	DFLVHPAA	Sequence	0.019	40550.66	75.00
26	HLA-B3901	ALSLDEPFIQ	ALSLEPFIQ	0	0	0	4	1	ALSLDEPFIQ	Sequence	0.019	40703.20	80.00
7	HLA-B3901	IYYILPRK	-IYYILPRK	0	0	1	0	0	IYYILPRK	Sequence	0.019	40739.75	80.00
5	HLA-B3901	ADIYYILP	ADIYYILP-	0	8	1	0	0	ADIYYILP	Sequence	0.019	40881.06	80.00
1	HLA-B3901	VLPDADIY	-VLPDADIY	0	0	1	0	0	VLPDADIY	Sequence	0.018	41373.65	85.00
24	HLA-B3901	PAALSLDE	-PAALSLDE	0	0	1	0	0	PAALSLDE	Sequence	0.017	41590.90	85.00
10	HLA-B3901	ILPRKVLQ	-ILPRKVLQ	0	0	1	0	0	ILPRKVLQ	Sequence	0.016	42102.98	90.00
25	HLA-B3901	AALSLDEP	AALSLDEP-	0	8	1	0	0	AALSLDEP	Sequence	0.015	42642.13	95.00
10	HLA-B3901	ILPRKVLQMD	ILPRKVLQM	0	0	0	0	0	ILPRKVLQM	Sequence	0.015	42715.07	95.00
12	HLA-B3901	PRKVLQMD	-PRKVLQMD	0	0	1	0	0	PRKVLQMD	Sequence	0.013	43525.44	99.00

Protein Sequence. Allele HLA-B3901. Number of high binders 0. Number of weak binders 3. Number of peptides 84

Link to Allele Frequencies in Worldwide Populations HLA-B3901

Rank Threshold for Strong binding peptides 0.500
Rank Threshold for Weak binding peptides 2.000

pos	HLA	peptide	Core	Offset	I_pos	I_len	D_pos	D_len	iCore	Identity	1-log50k(aff)	Affinity(nM)	%Rank	BindLeve
18	HLA-B4001	MDFLVHPAAL	MDFLVHPAL	0	0	0	7	1	MDFLVHPAAL	Sequence	0.176	7477.33	4.50	
16	HLA-B4001	LQMDFLVHP	LQMDFLVHP	0	0	0	0	0	LQMDFLVHP	Sequence	0.151	9791.72	5.50	
13	HLA-B4001	RKVLQMDFL	RKVLQMDFL	0	0	0	0	0	RKVLQMDFL	Sequence	0.150	9880.68	5.50	
21	HLA-B4001	LVHPAALSL	LVHPAALSL	0	0	0	0	0	LVHPAALSL	Sequence	0.130	12270.31	6.50	
18	HLA-B4001	MDFLVHPAA	MDFLVHPAA	0	0	0	0	0	MDFLVHPAA	Sequence	0.110	15257.51	8.50	
25	HLA-B4001	AALSLDEPF	AALSLDEPF	0	0	0	0	0	AALSLDEPF	Sequence	0.106	15897.03	9.00	
5	HLA-B4001	ADIYYILPR	ADIYYILPR	0	0	0	0	0	ADIYYILPR	Sequence	0.099	17086.52	10.00	
16	HLA-B4001	LQMDFLVHPA	LQMDFLVPA	0	0	0	7	1	LQMDFLVHPA	Sequence	0.099	17192.97	10.00	
2	HLA-B4001	LPDADIYYIL	LDADIYYIL	0	0	0	1	1	LPDADIYYIL	Sequence	0.097	17560.90	10.00	
8	HLA-B4001	YYILPRKVL	YYILPRKVL	0	0	0	0	0	YYILPRKVL	Sequence	0.094	18171.70	11.00	
3	HLA-B4001	PDADIYYIL	PDADIYYIL	0	0	0	0	0	PDADIYYIL	Sequence	0.091	18776.90	12.00	
20	HLA-B4001	FLVHPAALSL	FLVHAALSL	0	0	0	4	1	FLVHPAALSL	Sequence	0.082	20574.10	14.00	
17	HLA-B4001	QMDFLVHPA	QMDFLVHPA	0	0	0	0	0	QMDFLVHPA	Sequence	0.078	21459.46	15.00	
15	HLA-B4001	VLQMDFLVH	VLQMDFLVH	0	0	0	0	0	VLQMDFLVH	Sequence	0.072	23012.79	17.00	
15	HLA-B4001	VLQMDFLVHP	VQMDFLVHP	0	0	0	1	1	VLQMDFLVHP	Sequence	0.066	24415.11	20.00	
0	HLA-B4001	QVLPDADIY	QVLPDADIY	0	0	0	0	0	QVLPDADIY	Sequence	0.065	24782.38	21.00	
14	HLA-B4001	KVLQMDFLV	KVLQMDFLV	0	0	0	0	0	KVLQMDFLV	Sequence	0.062	25608.03	23.00	
19	HLA-B4001	DFLVHPAAL	DFLVHPAAL	0	0	0	0	0	DFLVHPAAL	Sequence	0.060	26037.75	24.00	
7	HLA-B4001	IYYILPRKV	IYYILPRKV	0	0	0	0	0	IYYILPRKV	Sequence	0.060	26138.79	24.00	
2	HLA-B4001	LPDADIYYI	LPDADIYYI	0	0	0	0	0	LPDADIYYI	Sequence	0.058	26626.33	25.00	
12	HLA-B4001	PRKVLQMDFL	RKVLQMDFL	1	0	0	0	0	RKVLQMDFL	Sequence	0.058	26735.45	26.00	
10	HLA-B4001	ILPRKVLQM	ILPRKVLQM	0	0	0	0	0	ILPRKVLQM	Sequence	0.057	27084.55	27.00	
26	HLA-B4001	ALSLDEPFI	ALSLDEPFI	0	0	0	0	0	ALSLDEPFI	Sequence	0.057	27119.73	27.00	
16	HLA-B4001	LQMDFLVH	LQMD-FLVH	0	4	1	0	0	LQMDFLVH	Sequence	0.056	27311.43	27.00	
17	HLA-B4001	QMDFLVHPAA	MDFLVHPAA	1	0	0	0	0	MDFLVHPAA	Sequence	0.054	27791.37	29.00	
27	HLA-B4001	LSLDEPFIQ	LSLDEPFIQ	0	0	0	0	0	LSLDEPFIQ	Sequence	0.054	27892.28	29.00	
23	HLA-B4001	HPAALSLDE	HPAALSLDE	0	0	0	0	0	HPAALSLDE	Sequence	0.052	28358.17	31.00	
1	HLA-B4001	VLPDADIYY	VLPDADIYY	0	0	0	0	0	VLPDADIYY	Sequence	0.051	28684.04	32.00	
24	HLA-B4001	PAALSLDEP	PAALSLDEP	0	0	0	0	0	PAALSLDEP	Sequence	0.051	28840.26	32.00	
5	HLA-B4001	ADIYYILPRK	ADIYYILPK	0	0	0	8	1	ADIYYILPRK	Sequence	0.050	29066.12	33.00	

4	HLA-B4001	DADIYYIL	-DADIYYIL	0	0	1	0	0	DADIYYIL	Sequence	0.050	29096.95	33.00
13	HLA-B4001	RKVLQMDFLV	RKVLQMDFL	0	0	0	0	0	RKVLQMDFL	Sequence	0.050	29198.52	33.00
20	HLA-B4001	FLVHPAALS	FLVHPAALS	0	0	0	0	0	FLVHPAALS	Sequence	0.049	29319.13	34.00
24	HLA-B4001	PAALSLDEPF	AALSLDEPF	1	0	0	0	0	AALSLDEPF	Sequence	0.047	29909.64	36.00
20	HLA-B4001	FLVHPAAL	F-LVHPAAL	0	1	1	0	0	FLVHPAAL	Sequence	0.047	29995.19	36.00
22	HLA-B4001	VHPAALSL	V-HPAALSL	0	1	1	0	0	VHPAALSL	Sequence	0.047	30076.78	37.00
4	HLA-B4001	DADIYYILP	DADIYYILP	0	0	0	0	0	DADIYYILP	Sequence	0.046	30464.22	38.00
6	HLA-B4001	DIYYILPRK	DIYYILPRK	0	0	0	0	0	DIYYILPRK	Sequence	0.046	30483.01	38.00
23	HLA-B4001	HPAALSLDEP	HAALSLDEP	0	0	0	1	1	HPAALSLDEP	Sequence	0.046	30496.20	38.00
9	HLA-B4001	YILPRKVLQ	YILPRKVLQ	0	0	0	0	0	YILPRKVLQ	Sequence	0.046	30536.49	38.00
25	HLA-B4001	AALSLDEPFI	AALSLDEPI	0	0	0	8	1	AALSLDEPFI	Sequence	0.045	30589.73	38.00
4	HLA-B4001	DADIYYILPR	ADIYYILPR	1	0	0	0	0	ADIYYILPR	Sequence	0.045	30695.49	39.00
18	HLA-B4001	MDFLVHPA	MDFLVH-PA	0	6	1	0	0	MDFLVHPA	Sequence	0.044	31114.13	41.00
11	HLA-B4001	LPRKVLQMD	LPRKVLQMD	0	0	0	0	0	LPRKVLQMD	Sequence	0.043	31461.12	42.00
14	HLA-B4001	KVLQMDFLVH	KVLQMFLVH	0	0	0	5	1	KVLQMDFLVH	Sequence	0.042	31599.29	42.00
21	HLA-B4001	LVHPAALSLD	LVHPAALSL	0	0	0	0	0	LVHPAALSL	Sequence	0.042	31828.50	43.00
17	HLA-B4001	QMDFLVHP	-QMDFLVHP	0	0	1	0	0	QMDFLVHP	Sequence	0.041	31993.20	44.00
7	HLA-B4001	IYYILPRKVL	YYILPRKVL	1	0	0	0	0	YYILPRKVL	Sequence	0.041	32249.68	45.00
26	HLA-B4001	ALSLDEPF	A-LSLDEPF	0	1	1	0	0	ALSLDEPF	Sequence	0.039	32629.45	47.00
22	HLA-B4001	VHPAALSLD	VHPAALSLD	0	0	0	0	0	VHPAALSLD	Sequence	0.039	32770.61	48.00
14	HLA-B4001	KVLQMDFL	K-VLQMDFL	0	1	1	0	0	KVLQMDFL	Sequence	0.037	33398.46	50.00
5	HLA-B4001	ADIYYILP	ADIYYILP-	0	8	1	0	0	ADIYYILP	Sequence	0.037	33399.20	50.00
13	HLA-B4001	RKVLQMDF	RKVLQMD-F	0	7	1	0	0	RKVLQMDF	Sequence	0.036	33840.43	55.00
25	HLA-B4001	AALSLDEP	A-ALSLDEP	0	1	1	0	0	AALSLDEP	Sequence	0.035	34162.71	55.00
9	HLA-B4001	YILPRKVLQM	YILRKVLQM	0	0	0	3	1	YILPRKVLQM	Sequence	0.035	34297.88	55.00
9	HLA-B4001	YILPRKVL	Y-ILPRKVL	0	1	1	0	0	YILPRKVL	Sequence	0.034	34441.06	60.00
0	HLA-B4001	QVLPDADIYY	QVLPDADIY	0	0	0	8	1	QVLPDADIYY	Sequence	0.034	34724.68	60.00
1	HLA-B4001	VLPDADIYYI	VLDADIYYI	0	0	0	2	1	VLPDADIYYI	Sequence	0.034	34777.69	60.00
11	HLA-B4001	LPRKVLQMDF	LPRKVLQMF	0	0	0	8	1	LPRKVLQMDF	Sequence	0.033	35000.80	60.00
8	HLA-B4001	YYILPRKVLQ	YYILPRKVL	0	0	0	0	0	YYILPRKVL	Sequence	0.033	35156.78	60.00
0	HLA-B4001	QVLPDADI	-QVLPDADI	0	0	1	0	0	QVLPDADI	Sequence	0.032	35252.00	65.00
26	HLA-B4001	ALSLDEPFIQ	ASLDEPFIQ	0	0	0	1	1	ALSLDEPFIQ	Sequence	0.031	35732.79	65.00
8	HLA-B4001	YYILPRKV	Y-YILPRKV	0	1	1	0	0	YYILPRKV	Sequence	0.031	35943.73	65.00
28	HLA-B4001	SLDEPFIQ	SLDEPF-IQ	0	6	1	0	0	SLDEPFIQ	Sequence	0.030	35977.98	65.00
12	HLA-B4001	PRKVLQMDF	PRKVLQMDF	0	0	0	0	0	PRKVLQMDF	Sequence	0.030	36134.04	70.00
6	HLA-B4001	DIYYILPR	-DIYYILPR	0	0	1	0	0	DIYYILPR	Sequence	0.030	36333.17	70.00
15	HLA-B4001	VLQMDFLV	VLQMDFL-V	0	7	1	0	0	VLQMDFLV	Sequence	0.029	36378.02	70.00
1	HLA-B4001	VLPDADIY	VL-PDADIY	0	2	1	0	0	VLPDADIY	Sequence	0.028	36820.34	75.00
19	HLA-B4001	DFLVHPAA	-DFLVHPAA	0	0	1	0	0	DFLVHPAA	Sequence	0.028	36889.34	75.00
6	HLA-B4001	DIYYILPRKV	IYYILPRKV	1	0	0	0	0	IYYILPRKV	Sequence	0.028	36893.71	75.00
7	HLA-B4001	IYYILPRK	I-YYILPRK	0	1	1	0	0	IYYILPRK	Sequence	0.028	37056.15	75.00
10	HLA-B4001	ILPRKVLQMD	ILRKVLQMD	0	0	0	2	1	ILPRKVLQMD	Sequence	0.027	37419.57	80.00
3	HLA-B4001	PDADIYYILP	PDADIYYIL	0	0	0	0	0	PDADIYYIL	Sequence	0.026	37658.39	80.00
22	HLA-B4001	VHPAALSLDE	VHAALSLDE	0	0	0	2	1	VHPAALSLDE	Sequence	0.026	37849.55	80.00
11	HLA-B4001	LPRKVLQM	LPRKVLQ-M	0	7	1	0	0	LPRKVLQM	Sequence	0.026	37920.06	80.00
21	HLA-B4001	LVHPAALS	LVHPAALS-	0	8	1	0	0	LVHPAALS	Sequence	0.025	38203.81	85.00
19	HLA-B4001	DFLVHPAALS	DFLVHPAAL	0	0	0	0	0	DFLVHPAAL	Sequence	0.024	38542.18	85.00
2	HLA-B4001	LPDADIYY	LPDADIY-Y	0	7	1	0	0	LPDADIYY	Sequence	0.023	38926.92	90.00
27	HLA-B4001	LSLDEPFI	L-SLDEPFI	0	1	1	0	0	LSLDEPFI	Sequence	0.023	38982.97	90.00
10	HLA-B4001	ILPRKVLQ	ILPRKVL-Q	0	7	1	0	0	ILPRKVLQ	Sequence	0.021	39960.49	95.00
24	HLA-B4001	PAALSLDE	PAALSLDE-	0	8	1	0	0	PAALSLDE	Sequence	0.020	40088.24	95.00
3	HLA-B4001	PDADIYYI	PDADIYY-I	0	7	1	0	0	PDADIYYI	Sequence	0.020	40148.13	95.00
23	HLA-B4001	HPAALSLD	HPAALSL-D	0	7	1	0	0	HPAALSLD	Sequence	0.020	40387.33	99.00
12	HLA-B4001	PRKVLQMD	-PRKVLQMD	0	0	1	0	0	PRKVLQMD	Sequence	0.017	41792.53	99.00

Protein Sequence. Allele HLA-B4001. Number of high binders 0. Number of weak binders 0. Number of peptides 84

Link to Allele Frequencies in Worldwide Populations HLA-B4001

\# Rank Threshold for Strong binding peptides 0.500

\# Rank Threshold for Weak binding peptides 2.000

----

pos	HLA	peptide	Core	Offset	I_pos	I_len	D_pos	D_len	iCore	Identity	1-log50k(aff)	Affinity(nM)	%Rank	BindLeve
25	HLA-B5801	AALSLDEPF	AALSLDEPF	0	0	0	0	0	AALSLDEPF	Sequence	0.461	340.90	0.80	<= WB
14	HLA-B5801	KVLQMDFLV	KVLQMDFLV	0	0	0	0	0	KVLQMDFLV	Sequence	0.384	786.72	1.30	<= WB
0	HLA-B5801	QVLPDADIY	QVLPDADIY	0	0	0	0	0	QVLPDADIY	Sequence	0.304	1868.40	2.50	
25	HLA-B5801	AALSLDEPFI	AASLDEPFI	0	0	0	2	1	AALSLDEPFI	Sequence	0.265	2829.74	3.00	
21	HLA-B5801	LVHPAALSL	LVHPAALSL	0	0	0	0	0	LVHPAALSL	Sequence	0.249	3367.70	3.50	
27	HLA-B5801	LSLDEPFIQ	LSLDEPFIQ	0	0	0	0	0	LSLDEPFIQ	Sequence	0.194	6156.43	5.50	
0	HLA-B5801	QVLPDADIYY	QVLPADIYY	0	0	0	4	1	QVLPDADIYY	Sequence	0.162	8633.95	7.50	
27	HLA-B5801	LSLDEPFI	LSLDEPF-I	0	7	1	0	0	LSLDEPFI	Sequence	0.157	9130.49	8.00	
14	HLA-B5801	KVLQMDFLVH	KVLQMDFLV	0	0	0	0	0	KVLQMDFLV	Sequence	0.155	9311.16	8.00	
24	HLA-B5801	PAALSLDEPF	PAALSLDPF	0	0	0	7	1	PAALSLDEPF	Sequence	0.148	10038.55	9.00	
26	HLA-B5801	ALSLDEPFI	ALSLDEPFI	0	0	0	0	0	ALSLDEPFI	Sequence	0.134	11685.17	11.00	
13	HLA-B5801	RKVLQMDFLV	RVLQMDFLV	0	0	0	1	1	RKVLQMDFLV	Sequence	0.133	11830.84	11.00	
14	HLA-B5801	KVLQMDFL	KVLQMDF-L	0	7	1	0	0	KVLQMDFL	Sequence	0.120	13648.62	13.00	
26	HLA-B5801	ALSLDEPFIQ	ASLDEPFIQ	0	0	0	1	1	ALSLDEPFIQ	Sequence	0.120	13669.91	13.00	
20	HLA-B5801	FLVHPAALSL	FLVPAALSL	0	0	0	3	1	FLVHPAALSL	Sequence	0.116	14260.47	14.00	
2	HLA-B5801	LPDADIYYI	LPDADIYYI	0	0	0	0	0	LPDADIYYI	Sequence	0.109	15341.77	15.00	
9	HLA-B5801	YILPRKVLQM	YILRKVLQM	0	0	0	3	1	YILPRKVLQM	Sequence	0.107	15718.82	16.00	
26	HLA-B5801	ALSLDEPF	-ALSLDEPF	0	0	1	0	0	ALSLDEPF	Sequence	0.103	16332.91	17.00	
23	HLA-B5801	HPAALSLDEP	HAALSLDEP	0	0	0	1	1	HPAALSLDEP	Sequence	0.098	17255.40	18.00	
13	HLA-B5801	RKVLQMDFL	RKVLQMDFL	0	0	0	0	0	RKVLQMDFL	Sequence	0.098	17339.24	18.00	
21	HLA-B5801	LVHPAALSLD	LVHPAALSL	0	0	0	0	0	LVHPAALSL	Sequence	0.098	17362.70	18.00	
17	HLA-B5801	QMDFLVHPA	QMDFLVHPA	0	0	0	0	0	QMDFLVHPA	Sequence	0.095	17918.28	19.00	
10	HLA-B5801	ILPRKVLQM	ILPRKVLQM	0	0	0	0	0	ILPRKVLQM	Sequence	0.087	19429.99	22.00	
1	HLA-B5801	VLPDADIYYI	VLDADIYYI	0	0	0	2	1	VLPDADIYYI	Sequence	0.084	20124.52	23.00	
24	HLA-B5801	PAALSLDEP	PAALSLDEP	0	0	0	0	0	PAALSLDEP	Sequence	0.078	21487.57	26.00	

16	HLA-B5801	LQMDFLVHPA	LMDFLVHPA	0	0	0	1	1	LQMDFLVHPA	Sequence	0.072	22921.35	30.00
9	HLA-B5801	YILPRKVLQ	YILPRKVLQ	0	0	0	0	0	YILPRKVLQ	Sequence	0.072	23049.17	30.00
7	HLA-B5801	IYYILPRKV	IYYILPRKV	0	0	0	0	0	IYYILPRKV	Sequence	0.070	23446.33	31.00
1	HLA-B5801	VLPDADIYY	VLPDADIYY	0	0	0	0	0	VLPDADIYY	Sequence	0.069	23745.28	32.00
2	HLA-B5801	LPDADIYYIL	LPADIYYIL	0	0	0	2	1	LPDADIYYIL	Sequence	0.068	24050.14	33.00
17	HLA-B5801	QMDFLVHPAA	QMDFLVHPA	0	0	0	8	1	QMDFLVHPAA	Sequence	0.065	24636.94	34.00
20	HLA-B5801	FLVHPAALS	FLVHPAALS	0	0	0	0	0	FLVHPAALS	Sequence	0.062	25471.26	37.00
16	HLA-B5801	LQMDFLVHP	LQMDFLVHP	0	0	0	0	0	LQMDFLVHP	Sequence	0.062	25570.39	37.00
7	HLA-B5801	IYYILPRKVL	IYYILPRVL	0	0	0	7	1	IYYILPRKVL	Sequence	0.058	26551.53	40.00
15	HLA-B5801	VLQMDFLVH	VLQMDFLVH	0	0	0	0	0	VLQMDFLVH	Sequence	0.058	26771.93	41.00
11	HLA-B5801	LPRKVLQMDF	LPRKVQMDF	0	0	0	5	1	LPRKVLQMDF	Sequence	0.058	26778.59	41.00
23	HLA-B5801	HPAALSLDE	HPAALSLDE	0	0	0	0	0	HPAALSLDE	Sequence	0.055	27555.72	44.00
12	HLA-B5801	PRKVLQMDF	PRKVLQMDF	0	0	0	0	0	PRKVLQMDF	Sequence	0.053	28130.50	46.00
0	HLA-B5801	QVLPDADI	QVLPDAD-I	0	7	1	0	0	QVLPDADI	Sequence	0.052	28504.91	48.00
8	HLA-B5801	YYILPRKVL	YYILPRKVL	0	0	0	0	0	YYILPRKVL	Sequence	0.052	28594.48	48.00
22	HLA-B5801	VHPAALSL	-VHPAALSL	0	0	1	0	0	VHPAALSL	Sequence	0.050	29169.78	50.00
11	HLA-B5801	LPRKVLQMD	LPRKVLQMD	0	0	0	0	0	LPRKVLQMD	Sequence	0.050	29223.48	55.00
8	HLA-B5801	YYILPRKVLQ	YYIPRKVLQ	0	0	0	3	1	YYILPRKVLQ	Sequence	0.049	29423.04	55.00
12	HLA-B5801	PRKVLQMDFL	PRKVLQDFL	0	0	0	6	1	PRKVLQMDFL	Sequence	0.049	29487.10	55.00
3	HLA-B5801	PDADIYYIL	PDADIYYIL	0	0	0	0	0	PDADIYYIL	Sequence	0.049	29522.85	55.00
1	HLA-B5801	VLPDADIY	-VLPDADIY	0	0	1	0	0	VLPDADIY	Sequence	0.049	29546.83	55.00
18	HLA-B5801	MDFLVHPAAL	MFLVHPAAL	0	0	0	1	1	MDFLVHPAAL	Sequence	0.047	30047.50	55.00
4	HLA-B5801	DADIYYILP	DADIYYILP	0	0	0	0	0	DADIYYILP	Sequence	0.047	30094.03	55.00
28	HLA-B5801	SLDEPFIQ	-SLDEPFIQ	0	0	1	0	0	SLDEPFIQ	Sequence	0.045	30747.68	60.00
22	HLA-B5801	VHPAALSLD	VHPAALSLD	0	0	0	0	0	VHPAALSLD	Sequence	0.045	30750.67	60.00
10	HLA-B5801	ILPRKVLQMD	ILPRKVLQM	0	0	0	0	0	ILPRKVLQM	Sequence	0.045	30819.96	60.00
6	HLA-B5801	DIYYILPRKV	DIYYILRKV	0	0	0	6	1	DIYYILPRKV	Sequence	0.044	31107.07	60.00
15	HLA-B5801	VLQMDFLVHP	VLQDFLVHP	0	0	0	3	1	VLQMDFLVHP	Sequence	0.044	31166.71	60.00
21	HLA-B5801	LVHPAALS	LVHPAALS-	0	8	1	0	0	LVHPAALS	Sequence	0.042	31848.13	65.00
5	HLA-B5801	ADIYYILPRK	AIYYILPRK	0	0	0	1	1	ADIYYILPRK	Sequence	0.042	31849.86	65.00
13	HLA-B5801	RKVLQMDF	RKV-LQMDF	0	3	1	0	0	RKVLQMDF	Sequence	0.041	31912.65	65.00
4	HLA-B5801	DADIYYILPR	DAIYYILPR	0	0	0	2	1	DADIYYILPR	Sequence	0.041	31915.41	65.00
25	HLA-B5801	AALSLDEP	AALSLDEP-	0	8	1	0	0	AALSLDEP	Sequence	0.041	32084.71	65.00
6	HLA-B5801	DIYYILPRK	DIYYILPRK	0	0	0	0	0	DIYYILPRK	Sequence	0.040	32367.84	65.00
15	HLA-B5801	VLQMDFLV	VL-QMDFLV	0	2	1	0	0	VLQMDFLV	Sequence	0.040	32456.91	65.00
19	HLA-B5801	DFLVHPAAL	DFLVHPAAL	0	0	0	0	0	DFLVHPAAL	Sequence	0.039	32745.81	70.00
22	HLA-B5801	VHPAALSLDE	VHAALSLDE	0	0	0	2	1	VHPAALSLDE	Sequence	0.038	33221.15	70.00
3	HLA-B5801	PDADIYYILP	PADIYYILP	0	0	0	1	1	PDADIYYILP	Sequence	0.037	33425.59	70.00
17	HLA-B5801	QMDFLVHP	QMDFLVHP-	0	8	1	0	0	QMDFLVHP	Sequence	0.036	33752.66	75.00
9	HLA-B5801	YILPRKVL	YILPRK-VL	0	6	1	0	0	YILPRKVL	Sequence	0.036	33910.43	75.00
4	HLA-B5801	DADIYYIL	DA-DIYYIL	0	2	1	0	0	DADIYYIL	Sequence	0.035	34260.80	75.00
18	HLA-B5801	MDFLVHPAA	MDFLVHPAA	0	0	0	0	0	MDFLVHPAA	Sequence	0.034	34432.86	75.00
3	HLA-B5801	PDADIYYI	-PDADIYYI	0	0	1	0	0	PDADIYYI	Sequence	0.034	34659.73	75.00
24	HLA-B5801	PAALSLDE	PAALSLDE-	0	8	1	0	0	PAALSLDE	Sequence	0.034	34787.49	80.00
11	HLA-B5801	LPRKVLQM	LPRKVL-QM	0	6	1	0	0	LPRKVLQM	Sequence	0.033	34872.26	80.00
20	HLA-B5801	FLVHPAAL	FLVH-PAAL	0	4	1	0	0	FLVHPAAL	Sequence	0.033	34919.84	80.00
5	HLA-B5801	ADIYYILPR	ADIYYILPR	0	0	0	0	0	ADIYYILPR	Sequence	0.033	35061.44	80.00
10	HLA-B5801	ILPRKVLQ	IL-PRKVLQ	0	2	1	0	0	ILPRKVLQ	Sequence	0.033	35163.25	80.00
18	HLA-B5801	MDFLVHPA	-MDFLVHPA	0	0	1	0	0	MDFLVHPA	Sequence	0.032	35499.65	80.00
2	HLA-B5801	LPDADIYY	LP-DADIYY	0	2	1	0	0	LPDADIYY	Sequence	0.031	35690.68	85.00
19	HLA-B5801	DFLVHPAALS	FLVHPAALS	1	0	0	0	0	FLVHPAALS	Sequence	0.029	36492.74	85.00
23	HLA-B5801	HPAALSLD	HPAALS-LD	0	6	1	0	0	HPAALSLD	Sequence	0.028	36794.46	90.00
16	HLA-B5801	LQMDFLVH	LQMDFL-VH	0	6	1	0	0	LQMDFLVH	Sequence	0.028	36824.33	90.00
8	HLA-B5801	YYILPRKV	Y-YILPRKV	0	1	1	0	0	YYILPRKV	Sequence	0.028	37064.97	90.00
5	HLA-B5801	ADIYYILP	-ADIYYILP	0	0	1	0	0	ADIYYILP	Sequence	0.024	38375.73	95.00
7	HLA-B5801	IYYILPRK	I-YYILPRK	0	1	1	0	0	IYYILPRK	Sequence	0.024	38445.98	95.00
6	HLA-B5801	DIYYILPR	DIYYILPR-	0	8	1	0	0	DIYYILPR	Sequence	0.021	39624.24	99.00
12	HLA-B5801	PRKVLQMD	PRKVLQMD-	0	8	1	0	0	PRKVLQMD	Sequence	0.020	40271.70	99.00
19	HLA-B5801	DFLVHPAA	D-FLVHPAA	0	1	1	0	0	DFLVHPAA	Sequence	0.015	42443.27	99.00

Protein Sequence. Allele HLA-B5801. Number of high binders 0. Number of weak binders 2. Number of peptides 84

Link to Allele Frequencies in Worldwide Populations HLA-B5801

Rank Threshold for Strong binding peptides 0.500
Rank Threshold for Weak binding peptides 2.000

---

pos	HLA	peptide	Core	Offset	I_pos	I_len	D_pos	D_len	iCore	Identity	1-log50k(aff)	Affinity(nM)	%Rank	BindLeve
16	HLA-B1501	LQMDFLVHPA	LQMDLVHPA	0	0	0	4	1	LQMDFLVHPA	Sequence	0.636	51.19	0.50	<= SB
21	HLA-B1501	LVHPAALSL	LVHPAALSL	0	0	0	0	0	LVHPAALSL	Sequence	0.518	184.44	1.20	<= WB
25	HLA-B1501	AALSLDEPF	AALSLDEPF	0	0	0	0	0	AALSLDEPF	Sequence	0.499	225.08	1.40	<= WB
20	HLA-B1501	FLVHPAALSL	FLHPAALSL	0	0	0	2	1	FLVHPAALSL	Sequence	0.493	240.07	1.40	<= WB
16	HLA-B1501	LQMDFLVHP	LQMDFLVHP	0	0	0	0	0	LQMDFLVHP	Sequence	0.478	282.59	1.60	<= WB
1	HLA-B1501	VLPDADIYY	VLPDADIYY	0	0	0	0	0	VLPDADIYY	Sequence	0.441	421.13	2.00	<= WB
16	HLA-B1501	LQMDFLVH	LQMD-FLVH	0	4	1	0	0	LQMDFLVH	Sequence	0.388	752.63	3.00	
0	HLA-B1501	QVLPDADIYY	QLPDADIYY	0	0	0	1	1	QVLPDADIYY	Sequence	0.345	1192.34	4.00	
15	HLA-B1501	VLQMDFLVHP	VQMDFLVHP	0	0	0	1	1	VLQMDFLVHP	Sequence	0.299	1964.66	5.00	
26	HLA-B1501	ALSLDEPF	AL-SLDEPF	0	2	1	0	0	ALSLDEPF	Sequence	0.286	2276.21	5.50	
9	HLA-B1501	YILPRKVLQM	YILPRVLQM	0	0	0	5	1	YILPRKVLQM	Sequence	0.282	2363.01	5.50	
15	HLA-B1501	VLQMDFLVH	VLQMDFLVH	0	0	0	0	0	VLQMDFLVH	Sequence	0.282	2376.91	5.50	
14	HLA-B1501	KVLQMDFLVH	KLQMDFLVH	0	0	0	1	1	KVLQMDFLVH	Sequence	0.263	2892.23	6.00	
0	HLA-B1501	QVLPDADIY	QVLPDADIY	0	0	0	0	0	QVLPDADIY	Sequence	0.261	2963.57	6.50	
17	HLA-B1501	QMDFLVHPAA	QMFLVHPAA	0	0	0	2	1	QMDFLVHPAA	Sequence	0.240	3744.25	7.00	
24	HLA-B1501	PAALSLDEPF	AALSLDEPF	1	0	0	0	0	AALSLDEPF	Sequence	0.237	3834.44	7.50	
10	HLA-B1501	ILPRKVLQM	ILPRKVLQM	0	0	0	0	0	ILPRKVLQM	Sequence	0.193	6169.90	10.00	
21	HLA-B1501	LVHPAALSLD	LVHPAALSL	0	0	0	0	0	LVHPAALSL	Sequence	0.191	6344.98	10.00	
25	HLA-B1501	AALSLDEPFI	AALSLDEPF	0	0	0	0	0	AALSLDEPF	Sequence	0.182	6966.43	11.00	
1	HLA-B1501	VLPDADIYYI	VLPDADIYY	0	0	0	0	0	VLPDADIYY	Sequence	0.161	8719.00	13.00	

18	HLA-B1501	MDFLVHPAAL	MDFLVHAAL	0	0	0	6	1	MDFLVHPAAL	Sequence	0.152	9647.95	14.00
1	HLA-B1501	VLPDADIY	VLPDADI-Y	0	7	1	0	0	VLPDADIY	Sequence	0.142	10787.69	15.00
20	HLA-B1501	FLVHPAAL	FLV-HPAAL	0	3	1	0	0	FLVHPAAL	Sequence	0.136	11526.58	16.00
17	HLA-B1501	QMDFLVHPA	QMDFLVHPA	0	0	0	0	0	QMDFLVHPA	Sequence	0.130	12301.15	17.00
18	HLA-B1501	MDFLVHPAA	MDFLVHPAA	0	0	0	0	0	MDFLVHPAA	Sequence	0.125	12866.51	18.00
11	HLA-B1501	LPRKVLQMDF	LPRKVLQMF	0	0	0	8	1	LPRKVLQMDF	Sequence	0.115	14454.65	20.00
2	HLA-B1501	LPDADIYY	-LPDADIYY	0	0	1	0	0	LPDADIYY	Sequence	0.110	15259.32	21.00
26	HLA-B1501	ALSLDEPFI	ALSLDEPFI	0	0	0	0	0	ALSLDEPFI	Sequence	0.106	15809.05	21.00
9	HLA-B1501	YILPRKVLQ	YILPRKVLQ	0	0	0	0	0	YILPRKVLQ	Sequence	0.104	16284.74	22.00
8	HLA-B1501	YYILPRKVL	YYILPRKVL	0	0	0	0	0	YYILPRKVL	Sequence	0.103	16419.55	22.00
14	HLA-B1501	KVLQMDFLV	KVLQMDFLV	0	0	0	0	0	KVLQMDFLV	Sequence	0.088	19380.23	26.00
22	HLA-B1501	VHPAALSL	-VHPAALSL	0	0	1	0	0	VHPAALSL	Sequence	0.086	19725.06	27.00
20	HLA-B1501	FLVHPAALS	FLVHPAALS	0	0	0	0	0	FLVHPAALS	Sequence	0.086	19805.25	27.00
21	HLA-B1501	LVHPAALS	LVHPAALS-	0	8	1	0	0	LVHPAALS	Sequence	0.085	19829.70	27.00
13	HLA-B1501	RKVLQMDF	R-KVLQMDF	0	1	1	0	0	RKVLQMDF	Sequence	0.085	19900.42	27.00
17	HLA-B1501	QMDFLVHP	-QMDFLVHP	0	0	1	0	0	QMDFLVHP	Sequence	0.085	20015.94	27.00
9	HLA-B1501	YILPRKVL	YILPRK-VL	0	6	1	0	0	YILPRKVL	Sequence	0.073	22688.67	32.00
13	HLA-B1501	RKVLQMDFL	RKVLQMDFL	0	0	0	0	0	RKVLQMDFL	Sequence	0.068	23926.60	34.00
5	HLA-B1501	ADIYYILPR	ADIYYILPR	0	0	0	0	0	ADIYYILPR	Sequence	0.068	23996.34	34.00
2	HLA-B1501	LPDADIYYIL	LDADIYYIL	0	0	0	1	1	LPDADIYYIL	Sequence	0.066	24560.55	35.00
13	HLA-B1501	RKVLQMDFLV	RVLQMDFLV	0	0	0	1	1	RKVLQMDFLV	Sequence	0.066	24602.58	35.00
7	HLA-B1501	IYYILPRKVL	IYYILPRVL	0	0	0	7	1	IYYILPRKVL	Sequence	0.063	25199.87	36.00
8	HLA-B1501	YYILPRKVLQ	YILPRKVLQ	0	0	0	1	1	YYILPRKVLQ	Sequence	0.063	25288.09	37.00
15	HLA-B1501	VLQMDFLV	VLQMDFLV-	0	8	1	0	0	VLQMDFLV	Sequence	0.061	25910.71	38.00
27	HLA-B1501	LSLDEPFIQ	LSLDEPFIQ	0	0	0	0	0	LSLDEPFIQ	Sequence	0.057	27050.88	40.00
10	HLA-B1501	ILPRKVLQM	ILPRKVLQM	0	0	0	0	0	ILPRKVLQM	Sequence	0.052	28503.05	44.00
19	HLA-B1501	DFLVHPAAL	DFLVHPAAL	0	0	0	0	0	DFLVHPAAL	Sequence	0.052	28567.59	44.00
18	HLA-B1501	MDFLVHPA	MDFLVHPA-	0	8	1	0	0	MDFLVHPA	Sequence	0.051	28785.71	45.00
26	HLA-B1501	ALSLDEPFIQ	ALSDEPFIQ	0	0	0	3	1	ALSLDEPFIQ	Sequence	0.051	28837.77	45.00
0	HLA-B1501	QVLPDADI	-QVLPDADI	0	0	1	0	0	QVLPDADI	Sequence	0.049	29459.67	46.00
5	HLA-B1501	ADIYYILPRK	ADIYYILPK	0	0	0	8	1	ADIYYILPRK	Sequence	0.048	29616.60	47.00
14	HLA-B1501	KVLQMDFL	KVLQMD-FL	0	6	1	0	0	KVLQMDFL	Sequence	0.047	30196.77	48.00
12	HLA-B1501	PRKVLQMDF	PRKVLQMDF	0	0	0	0	0	PRKVLQMDF	Sequence	0.046	30323.15	49.00
5	HLA-B1501	ADIYYILP	ADIYYILP-	0	8	1	0	0	ADIYYILP	Sequence	0.045	30585.76	50.00
7	HLA-B1501	IYYILPRKV	IYYILPRKV	0	0	0	0	0	IYYILPRKV	Sequence	0.040	32273.77	55.00
2	HLA-B1501	LPDADIYYI	LPDADIYYI	0	0	0	0	0	LPDADIYYI	Sequence	0.038	33114.20	60.00
11	HLA-B1501	LPRKVLQM	-LPRKVLQM	0	0	1	0	0	LPRKVLQM	Sequence	0.037	33423.41	60.00
3	HLA-B1501	PDADIYYIL	PDADIYYIL	0	0	0	0	0	PDADIYYIL	Sequence	0.036	33847.75	60.00
19	HLA-B1501	DFLVHPAA	-DFLVHPAA	0	0	1	0	0	DFLVHPAA	Sequence	0.034	34498.86	65.00
27	HLA-B1501	LSLDEPFI	LSLDE-PFI	0	5	1	0	0	LSLDEPFI	Sequence	0.033	35103.56	65.00
6	HLA-B1501	DIYYILPRKV	DIYYILPRV	0	0	0	8	1	DIYYILPRKV	Sequence	0.032	35437.11	70.00
12	HLA-B1501	PRKVLQMDFL	RKVLQMDFL	1	0	0	0	0	RKVLQMDFL	Sequence	0.032	35442.85	70.00
6	HLA-B1501	DIYYILPRK	DIYYILPRK	0	0	0	0	0	DIYYILPRK	Sequence	0.031	35590.43	70.00
19	HLA-B1501	DFLVHPAALS	FLVHPAALS	1	0	0	0	0	FLVHPAALS	Sequence	0.031	35842.37	70.00
4	HLA-B1501	DADIYYILPR	ADIYYILPR	1	0	0	0	0	ADIYYILPR	Sequence	0.031	35866.44	70.00
8	HLA-B1501	YYILPRKV	YYILPRKV-	0	8	1	0	0	YYILPRKV	Sequence	0.031	35875.34	70.00
23	HLA-B1501	HPAALSLDEP	HAALSLDEP	0	0	0	1	1	HPAALSLDEP	Sequence	0.028	37021.28	75.00
11	HLA-B1501	LPRKVLQMD	LPRKVLQMD	0	0	0	0	0	LPRKVLQMD	Sequence	0.027	37227.75	75.00
25	HLA-B1501	AALSLDEP	AALSLDEP-	0	8	1	0	0	AALSLDEP	Sequence	0.027	37406.61	75.00
10	HLA-B1501	ILPRKVLQ	IL-PRKVLQ	0	2	1	0	0	ILPRKVLQ	Sequence	0.027	37473.86	75.00
23	HLA-B1501	HPAALSLDE	HPAALSLDE	0	0	0	0	0	HPAALSLDE	Sequence	0.025	38101.85	80.00
6	HLA-B1501	DIYYILPR	-DIYYILPR	0	0	1	0	0	DIYYILPR	Sequence	0.023	38942.92	80.00
4	HLA-B1501	DADIYYIL	-DADIYYIL	0	0	1	0	0	DADIYYIL	Sequence	0.023	39150.36	85.00
7	HLA-B1501	IYYILPRK	-IYYILPRK	0	0	1	0	0	IYYILPRK	Sequence	0.022	39364.01	85.00
24	HLA-B1501	PAALSLDEP	PAALSLDEP	0	0	0	0	0	PAALSLDEP	Sequence	0.020	40166.83	90.00
28	HLA-B1501	SLDEPFIQ	SL-DEPFIQ	0	2	1	0	0	SLDEPFIQ	Sequence	0.020	40317.04	90.00
4	HLA-B1501	DADIYYILP	DADIYYILP	0	0	0	0	0	DADIYYILP	Sequence	0.019	40567.35	90.00
22	HLA-B1501	VHPAALSLD	VHPAALSLD	0	0	0	0	0	VHPAALSLD	Sequence	0.017	41484.37	95.00
3	HLA-B1501	PDADIYYILP	PDADIYYIL	0	0	0	0	0	PDADIYYIL	Sequence	0.017	41580.55	95.00
3	HLA-B1501	PDADIYYI	-PDADIYYI	0	0	1	0	0	PDADIYYI	Sequence	0.016	41998.79	95.00
22	HLA-B1501	VHPAALSLDE	VHAALSLDE	0	0	0	2	1	VHPAALSLDE	Sequence	0.015	42675.80	95.00
23	HLA-B1501	HPAALSLD	HPAALSLD-	0	8	1	0	0	HPAALSLD	Sequence	0.014	42848.38	99.00
12	HLA-B1501	PRKVLQMD	PRKVLQMD-	0	8	1	0	0	PRKVLQMD	Sequence	0.011	44463.17	99.00
24	HLA-B1501	PAALSLDE	PAALSLDE-	0	8	1	0	0	PAALSLDE	Sequence	0.010	45042.27	99.00

Protein Sequence. Allele HLA-B1501. Number of high binders 1. Number of weak binders 5. Number of peptides 84

Link to Allele Frequencies in Worldwide Populations HLA-B1501

Go **back**.
