# Supplementary material for: Identification, design, and in vivo proof of concept of a shared APC neoantigen delivered via a self-amplifying RNA containing virus-like nanoparticle for cancer vaccination
Source: Front Immunol. 2026 Jun 4;17:1810178. doi: 10.3389/fimmu.2026.1810178 (PMC13275407; doi:10.3389/fimmu.2026.1810178)
Supplement: Supplementary file 3 [file DataSheet3.pdf]

## APC protein Nucleotide Sequence (8532 nt):

ATGGCTGCAGCTTCATATGATCAGTTGTTAAAGCAAGTTGAGGCACTGAAGATGGAGAACTCAAATCTTC
GACAAGAGCTAGAAGATAATTCCAATCATCTTACAAAACTGGAAACTGAGGCATCTAATATGAAGGAAGT
ACTTAAACAACTACAAGGAAGTATTGAAGATGAAGCTATGGCTTCTTCTGGACAGATTGATTTATTAGAG
CGTCTTAAAGAGCTTAACTTAGATAGCAGTAATTTCCCTGGAGTAAAACTGCGGTCAAAAATGTCCCTCC
GTTCTTATGGAAGCCGGGAAGGATCTGTATCAAGCCGTTCTGGAGAGTGCAGTCCTGTTCCTATGGGTTC
ATTTCCAAGAAGAGGGTTTGTAAATGGAAGCAGAGAAAGTACTGGATATTTAGAAGAACTTGAGAAAGAG
AGGTCATTGCTTCTTGCTGATCTTGACAAAGAAGAAAAGGAAAAAGACTGGTATTACGCTCAACTTCAGA
ATCTCACTAAAAGAATAGATAGTCTTCCTTTAACTGAAAATTTTTCCTTACAAACAGATATGACCAGAAG
GCAATTGGAATATGAAGCAAGGCAAATCAGAGTTG CGATGGAAGAACAACTAGGTACCTGCCAGGATATG
GAAAAACGAGCACAGCGAAGAATAGCCAGAATTCAGCAAATCGAAAAGGACATACTTCGTATACGACAGC
TTTTACAGTCCCAAGCAACAGAAGCAGAGAGGTCATCTCAGAACAAGCATGAAACCGGCTCACATGATGC
TGAGCGGCAGAATGAAGGTCAAGGAGTGGGAGAAATCAACATGGCAACTTCTGGTAATGGTCAGGGTTCA
ACTACACGAATGGACCATGAAACAGCCAGTGTTTTGAGTTCTAGTAGCACACACTCTGCACCTCGAAGGC
TGACAAGTCATCTGGGAACCAAGGTGGAAATGGTGTATTCATTGTTGTCAATGCTTGGTACTCATGATAA
GGATGATATGTCGCGAACTTTGCTAGCTATGTCTAGCTCCCAAGACAGCTGTATATCCATGCGACAGTCT
GGATGTCTTCCTCTCCTCATCCAGCTTTTACATGGCAATGACAAAGACTCTGTATTGTTGGGAAATTCCC
GGGGCAGTAAAGAGGCTCGGGCCAGGGCCAGTGCAGCACTCCACAACATCATTCACTCACAGCCTGATGA
CAAGAGAGGCAGGCGTGAAATCCGAGTCCTTCATCTTTTGGAACAGATACGCGCTTACTGTGAAACCTGT
TGGGAGTGGCAGGAAGCTCATGAACCAGGCATGGACCAGGACAAAAATCCAATGCCAGCTCCTGTTGAAC
ATCAGATCTGTCCTGCTGTGTGTGTTCTAATGAAACTTTCATTTGATGAAGAGCATAGACATGCAATGAA
TGAACTAGGGGGACTACAGGCCATTGCAGAATTATTGCAAGTGGACTGTGAAATGTATGGGCTTACTAAT
GACCACTACAGTATTACACTAAGACGATATGCTGGAATGGCTTTGACAAACTTGACTTTTGGAGATGTAG
CCAACAAGGCTACGCTATGCTCTATGAAAGGCTGCATGAGAGCACTTGTGGCCCAACTAAAATCTGAAAG
TGAAGACTTACAGCAGGTTATTGCGAGTGTTTTGAGGAATTTGTCTTGGCGAGCAGATGTAAATAGTAAA
AAGACGTTGCGAGAAGTTGGAAGTGTGAAAGCATTGATGGAATGTGCTTTAGAAGTTAAAAAGGAATCAA
CCCTCAAAAGCGTATTGAGTGCCTTATGGAATTTGTCAGCACATTGCACTGAGAATAAAGCTGATATATG
TGCTGTAGATGGTGCACTTGCATTTTTGGTTGGCACTCTTACTTACCGGAGCCAGACAAACACTTTAGCC
ATTATTGAAAGTGGAGGTGGGATATTACGGAATGTGTCCAGCTTGATAGCTACAAATGAGGACCACAGGC
AAATCCTAAGAGAGAACAACTGTCTACAAACTTTATTACAACACTTAAAATCTCATAGTTTGACAATAGT
CAGTAATGCATGTGGAACTTTGTGGAATCTCTCAGCAAGAAATCCTAAAGACCAGGAAGCATTATGGGAC
ATGGGGGCAGTTAGCATGCTCAAGAACCTCATTCATTCAAAGCACAAAATGATTGCTATGGGAAGTGCTG
CAGCTTTAAGGAATCTCATGGCAAATAGGCCTGCGAAGTACAAGGATGCCAATATTATGTCTCCTGGCTC
AAGCTTGCCATCTCTTCATGTTAGGAAACAAAAAGCCCTAGAAGCAGAATTAGATGCTCAGCACTTATCA
GAAACTTTTGACAATATAGACAATTTAAGTCCCAAGGCATCTCATCGTAGTAAGCAGAGACACAAGCAAA
GTCTCTATGGTGATTATGTTTTTGACACCAATCGACATGATGATAATAGGTCAGACAATTTTAATACTGG
CAACATGACTGTCCTTTCACCATATTTGAATACTACAGTGTTACCCAGCTCCTCTTCATCAAGAGGAAGC
TTAGATAGTTCTCGTTCTGAAAAAGATAGAAGTTTGGAGAGAGAACGCGGAATTGGTCTAGGCAACTACC
ATCCAGCAACAGAAAATCCAGGAACTTCTTCAAAGCGAGGTTTGCAGATCTCCACCACTGCAGCCCAGAT
TGCCAAAGTCATGGAAGAAGTGTCAGCCATTCATACCTCTCAGGAAGACAGAAGTTCTGGGTCTACCACT
GAATTACATTGTGTGACAGATGAGAGAAATGCACTTAGAAGAAGCTCTGCTGCCCATACACATTCAAACA
CTTACAATTTCACTAAGTCGGAAAATTCAAATAGGACATGTTCTATGCCTTATGCCAAATTAGAATACAA
GAGATCTTCAAATGATAGTTTAAATAGTGTCAGTAGTAGTGATGGTTATGGTAAAAGAGGTCAAATGAAA
CCCTCGATTGAATCCTATTCTGAAGATGATGAAAGTAAGTTTTGCAGTTATGGTCAATACCCAGCCGACC
TAGCCCATAAAATACATAGTGCAAATCATATGGATGATAATGATGGAGAACTAGATACACCAATAAATTA
TAGTCTTAAATATTCAGATGAGCAGTTGAACTCTGGAAGGCAAAGTCCTTCACAGAATGAAAGATGGGCA
AGACCCAAACACATAATAGAAGATGAAATAAAACAAAGTGAGCAAAGACAATCAAGGAATCAAAGTACAA
CTTATCCTGTTTATACTGAGAGCACTGATGATAAACACCTCAAGTTCCAACCACATTTTGGACAGCAGGA
ATGTGTTTCTCCATACAGGTCACGGGGAGCCAATGGTTCAGAAACAAATCGAGTGGGTTCTAATCATGGA
ATTAATCAAAATGTAAGCCAGTCTTTGTGTCAAGAAGATGACTATGAAGATGATAAGCCTACCAATTATA
GTGAACGTTACTCTGAAGAAGAACAGCATGAAGAAGAAGAGAGACCAACAAATTATAGCATAAAATATAA
TGAAGAGAAACGTCATGTGGATCAGCCTATTGATTATAGTTTAAAATATGCCACAGATATTCCTTCATCA

CAGAAACAGTCATTTTCATTCTCAAAGAGTTCATCTGGACAAAGCAGTAAAACCGAACATATGTCTTCAA
GCAGTGAGAATACGTCCACACCTTCATCTAATGCCAAGAGGCAGAATCAGCTCCATCCAAGTTCTGCACA
GAGTAGAAGTGGTCAGCCTCAAAAGGCTGCCACTTGCAAAGTTTCTTCTATTAACCAAGAAACAATACAG
ACTTATTGTGTAGAAGATACTCCAATATGTTTTTCAAGATGTAGTTCATTATCATCTTTGTCATCAGCTG
AAGATGAAATAGGATGTAATCAGACGACACAGGAAGCAGATTCTGCTAATACCCTGCAAATAGCAGAAAT
AAAAGAAAAGATTGGAACTAGGTCAGCTGAAGATCCTGTGAGCGAAGTTCCAGCAGTGTCACAGCACCCT
AGAACCAAATCCAGCAGACTGCAGGGTTCTAGTTTATCTTCAGAATCAGCCAGGCACAAAGCTGTTGAAT
TTTCTTCAGGAGCGAAATCTCCCTCCAAAAGTGGTGCTCAGACACCCAAAAGTCCACCTGAACACTATGT
TCAGGAGACCCCACTCATGTTTAGCAGATGTACTTCTGTCAGTTCACTTGATAGTTTTGAGAGTCGTTCG
ATTGCCAGCTCCGTTCAGAGTGAACCATGCAGTGGAATGGTAAGTGGCATTATAAGCCCCAGTGATCTTC
CAGATAGCCCTGGACAAACCATGCCACCAAGCAGAAGTAAAACACCTCCACCACCTCCTCAAACAGCTCA
AACCAAGCGAGAAGTACCTAAAAATAAAGCACCTACTGCTGAAAAGAGAGAGAGTGGACCTAAGCAAGCT
GCAGTAAATGCTGCAGTTCAGAGGGTCCAGGTTCTTCCAGATGCTGATACTTTATTACATTTTGCCACGG
AAAGTACTCCAGATGGATTTTCTTGTTCATCCAGCCTGAGTGCTCTGAGCCTCGATGAGCCATTTATACA
GAAAGATGTGGAATTAAGAATAATGCCTCCAGTTCAGGAAAATGACAATGGGAATGAAACAGAATCAGAG
CAGCCTAAAGAATCAAATGAAAACCAAGAGAAAGAGGCAGAAAAAACTATTGATTCTGAAAAGGACCTAT
TAGATGATTCAGATGATGATGATATTGAAATACTAGAAGAATGTATTATTTCTGCCATGCCAACAAAGTC
ATCACGTAAAGCAAAAAAGCCAGCCCAGACTGCTTCAAAATTACCTCCACCTGTGGCAAGGAAACCAAGT
CAGCTGCCTGTGTACAAACTTCTACCATCACAAAACAGGTTGCAACCCCAAAAGCATGTTAGTTTTACAC
CGGGGGATGATATGCCACGGGTGTATTGTGTTGAAGGGACACCTATAAACTTTTCCACAGCTACATCTCT
AAGTGATCTAACAATCGAATCCCCTCCAAATGAGTTAGCTGCTGGAGAAGGAGTTAGAGGAGGGGCACAG
TCAGGTGAATTTGAAAAACGAGATACCATTCCTACAGAAGGCAGAAGTACAGATGAGGCTCAAGGAGGAA
AAACCTCATCTGTAACCATACCTGAATTGGATGACAATAAAGCAGAGGAAGGTGATATTCTTGCAGAATG
CATTAATTCTGCTATGCCCAAAGGGAAAAGTCACAAGCCTTTCCGTGTGAAAAAGATAATGGACCAGGTC
CAGCAAGCATCTGCGTCTTCTTCTGCACCCAACAAAAATCAGTTAGATGGTAAGAAAAAGAAACCAACTT
CACCAGTAAAACCTATACCACAAAATACTGAATATAGGACACGTGTAAGAAAAAATGCAGACTCAAAAAA
TAATTTAAATGCTGAGAGAGTTTTCTCAGACAACAAAGATTCAAAGAAACAGAATTTGAAAAATAATTCC
AAGGTCTTCAATGATAAGCTCCCAAATAATGAAGATAGAGTCAGAGGAAGTTTTGCTTTTGATTCACCTC
ATCATTACACGCCTATTGAAGGAACTCCTTACTGTTTTTCACGAAATGATTCTTTGAGTTCTCTAGATTT
TGATGATGATGATGTTGACCTTTCCAGGGAAAAGGCTGAATTAAGAAAGGCAAAAGAAAATAAGGAATCA
GAGGCTAAAGTTACCAGCCACACAGAACTAACCTCCAACCAACAATCAGCTAATAAGACACAAGCTATTG
CAAAGCAGCCAATAAATCGAGGTCAGCCTAAACCCATACTTCAGAAACAATCCACTTTTCCCCAGTCATC
CAAAGACATACCAGACAGAGGGGCAGCAACTGATGAAAAGTTACAGAATTTTGCTATTGAAAATACTCCG
GTTTGCTTTTCTCATAATTCCTCTCTGAGTTCTCTCAGTGACATTGACCAAGAAAACAACAATAAAGAAA
ATGAACCTATCAAAGAGACTGAGCCCCCTGACTCACAGGGAGAACCAAGTAAACCTCAAGCATCAGGCTA
TGCTCCTAAATCATTTCATGTTGAAGATACCCCAGTTTGTTTCTCAAGAAACAGTTCTCTCAGTTCTCTT
AGTATTGACTCTGAAGATGACCTGTTGCAGGAATGTATAAGCTCCGCAATGCCAAAAAAGAAAAAGCCTT
CAAGACTCAAGGGTGATAATGAAAAACATAGTCCCAGAAATATGGGTGGCATATTAGGTGAAGATCTGAC
ACTTGATTTGAAAGATATACAGAGACCAGATTCAGAACATGGTCTATCCCCTGATTCAGAAAATTTTGAT
TGGAAAGCTATTCAGGAAGGTGCAAATTCCATAGTAAGTAGTTTACATCAAGCTGCTGCTGCTGCATGTT
TATCTAGACAAGCTTCGTCTGATTCAGATTCCATCCTTTCCCTGAAATCAGGAATCTCTCTGGGATCACC
ATTTCATCTTACACCTGATCAAGAAGAAAAACCCTTTACAAGTAATAAAGGCCCACGAATTCTAAAACCA
GGGGAGAAAAGTACATTGGAAACTAAAAAGATAGAATCTGAAAGTAAAGGAATCAAAGGAGGAAAAAAAG
TTTATAAAAGTTTGATTACTGGAAAAGTTCGATCTAATTCAGAAATTTCAGGCCAAATGAAACAGCCCCT
TCAAGCAAACATGCCTTCAATCTCTCGAGGCAGGACAATGATTCATATTCCAGGAGTTCGAAATAGCTCC
TCAAGTACAAGTCCTGTTTCTAAAAAAGGCCCACCCCTTAAGACTCCAGCCTCCAAAAGCCCTAGTGAAG
GTCAAACAGCCACCACTTCTCCTAGAGGAGCCAAGCCATCTGTGAAATCAGAATTAAGCCCTGTTGCCAG
GCAGACATCCCAAATAGGTGGGTCAAGTAAAGCACCTTCTAGATCAGGATCTAGAGATTCGACCCCTTCA
AGACCTGCCCAGCAACCATTAAGTAGACCTATACAGTCTCCTGGCCGAAACTCAATTTCCCCTGGTAGAA
ATGGAATAAGTCCTCCTAACAAATTATCTCAACTTCCAAGGACATCATCCCCTAGTACTGCTTCAACTAA
GTCCTCAGGTTCTGGAAAAATGTCATATACATCTCCAGGTAGACAGATGAGCCAACAGAACCTTACCAAA
CAAACAGGTTTATCCAAGAATGCCAGTAGTATTCCAAGAAGTGAGTCTGCCTCCAAAGGACTAAATCAGA
TGAATAATGGTAATGGAGCCAATAAAAAGGTAGAACTTTCTAGAATGTCTTCAACTAAATCAAGTGGAAG

TGAATCTGATAGATCAGAAAGACCTGTATTAGTACGCCAGTCAACTTTCATCAAAGAAGCTCCAAGCCCA
ACCTTAAGAAGAAAATTGGAGGAATCTGCTTCATTTGAATCTCTTTCTCCATCATCTAGACCAGCTTCTC
CCACTAGGTCCCAGGCACAAACTCCAGTTTTAAGTCCTTCCCTTCCTGATATGTCTCTATCCACACATTC
GTCTGTTCAGGCTGGTGGATGGCGAAAACTCCCACCTAATCTCAGTCCCACTATAGAGTATAATGATGGA
AGACCAGCAAAGCGCCATGATATTGCACGGTCTCATTCTGAAAGTCCTTCTAGACTTCCAATCAATAGGT
CAGGAACCTGGAAACGTGAGCACAGCAAACATTCATCATCCCTTCCTCGAGTAAGCACTTGGAGAAGAAC
TGGAAGTTCATCTTCAATTCTTTCTGCTTCATCAGAATCCAGTGAAAAAGCAAAAAGTGAGGATGAAAAA
CATGTGAACTCTATTTCAGGAACCAAACAAAGTAAAGAAAACCAAGTATCCGCAAAAGGAACATGGAGAA
AAATAAAAGAAAATGAATTTTCTCCCACAAATAGTACTTCTCAGACCGTTTCCTCAGGTGCTACAAATGG
TGCTGAATCAAAGACTCTAATTTATCAAATGGCACCTGCTGTTTCTAAAACAGAGGATGTTTGGGTGAGA
ATTGAGGACTGTCCCATTAACAATCCTAGATCTGGAAGATCTCCCACAGGTAATACTCCCCCGGTGATTG
ACAGTGTTTCAGAAAAGGCAAATCCAAACATTAAAGATTCAAAAGATAATCAGGCAAAACAAAATGTGGG
TAATGGCAGTGTTCCCATGCGTACCGTGGGTTTGGAAAATCGCCTGAACTCCTTTATTCAGGTGGATGCC
CCTGACCAAAAAGGAACTGAGATAAAACCAGGACAAAATAATCCTGTCCCTGTATCAGAGACTAATGAAA
GTTCTATAGTGGAACGTACCCCATTCAGTTCTAGCAGCTCAAGCAAACACAGTTCACCTAGTGGGACTGT
TGCTGCCAGAGTGACTCCTTTTAATTACAACCCAAGCCCTAGGAAAAGCAGCGCAGATAGCACTTCAGCT
CGGCCATCTCAGATCCCAACTCCAGTGAATAACAACACAAAGAAGCGAGATTCCAAAACTGACAGCACAG
AATCCAGTGGAACCCAAAGTCCTAAGCGCCATTCTGGGTCTTACCTTGTGACATCTGTTTAA

**APC protein DNA Translation (2843 aa):**

MAAASYDQLLKQVEALKMENSNLRQELEDNSNHLTKLETEASNMKEVLKQLQGSIEDEAMASSGQIDLLE
RLKELNLDSSNFPGVKLRSKMSLRSYGSREGSVSSRSGECSPVPMGSFPRRGFVNGSRESTGYLEELEKE
RSLLLADLDKEEKEKDWYYAQLQNLTKRIDSLPLTENFSLQTDMTRRQLEYEARQIRVAMEEQLGTCQDM
EKRAQRRIARIQQIEKDILRIRQLLQSQATEAERSSQNKHETGSHDAERQNEGQGVGEINMATSGNGQGS
TTRMDHETASVLSSSSTHSAPRRLTSHLGTKVEMVYSLLSMLGTHDKDDMSRTLLAMSSSQDSCISMRQS
GCLPLLIQLLHGNDKDSVLLGNSRGSKEARARASAALHNIIHSQPDDKRGRREIRVLHLLEQIRAYCETC
WEWQEAHEPGMDQDKNPMPAPVEHQICPAVCVLMKLSFDEEHRHAMNELGGLQAIAELLQVDCEMYGLTN
DHYSITLRRYAGMALTNLTFGDVANKATLCSMKGCMRALVAQLKSESEDLQQVIASVLRNLSWRADVNSK
KTLREVGSVKALMECALEVKKESTLKSVLSALWNLSAHCTENKADICAVDGALAFLVGTLTYRSQTNTLA
IIESGGGILRNVSSLIATNEDHRQILRENNCLQTLLQHLKSHSLTIVSNACGTLWNLSARNPKDQEALWD
MGAVSMLKNLIHSKHKMIAMGSAAALRNLMANRPAKYKDANIMSPGSSLPSLHVRKQKALEAELDAQHLS
ETFDNIDNLSPKASHRSKQRHKQSLYGDYVFDTNRHDDNRSDNFNTGNMTVLSPYLNTTVLPSSSSSRGS
LDSSRSEKDRSLERERGIGLGNYHPATENPGTSSKRGLQISTTAAQIAKVMEEVSAIHTSQEDRSSGSTT
ELHCVTDERNALRRSSAAHTHSNTYNFTKSENSNRTCSMPYAKLEYKRSSNDSLNSVSSSDGYGKRGQMK
PSIESYSEDDESKFCSYGQYPADLAHKIHSANHMDDNDGELDTPINYSLKYSDEQLNSGRQSPSQNERWA
RPKHIIEDEIKQSEQRQSRNQSTTYPVYTESTDDKHLKFQPHFGQQECVSPYRSRGANGSETNRVGSNHG
INQNVSQSLCQEDDYEDDKPTNYSERYSEEEQHEEEERPTNYSIKYNEEKRHVDQPIDYSLKYATDIPSS
QKQSFSFSKSSSGQSSKTEHMSSSSENTSTPSSNAKRQNQLHPSSAQSRSGQPQKAATCKVSSINQETIQ
TYCVEDTPICFSRCSSLSSLSSAEDEIGCNQTTQEADSANTLQIAEIKEKIGTRSAEDPVSEVPAVSQHP
RTKSSRLQGSSLSSESARHKAVEFSSGAKSPSKSGAQTPKSPPEHYVQETPLMFSRCTSVSSLDSFESRS
IASSVQSEPCSGMVSGIISPSDLPDSPGQTMPPSRSKTPPPPPQTAQTKREVPKNKAPTAEKRESGPKQA
AVNAAVQRVQVLPDADTLLHFATESTPDGFSCSSSLSALSLDEPFIQKDVELRIMPPVQENDNGNETESE
QPKESNENQEKEAEKTIDSEKDLLDDSDDDDIEILEECIISAMPTKSSRKAKKPAQTASKLPPPVARKPS
QLPVYKLLPSQNRLQPQKHVSFTPGDDMPRVYCVEGTPINFSTATSLSDLTIESPPNELAAGEGVRGGAQ
SGEFEKRDTIPTEGRSTDEAQGGKTSSVTIPELDDNKAEEGDILAECINSAMPKGKSHKPFRVKKIMDQV
QQASASSSAPNKNQLDGKKKKPTSPVKPIPQNTEYRTRVRKNADSKNNLNAERVFSDNKDSKKQNLKNNS
KVFNDKLPNNEDRVRGSFAFDSPHHYTPIEGTPYCFSRNDSLSSLDFDDDDVDLSREKAELRKAKENKES
EAKVTSHTELTSNQQSANKTQAIAKQPINRGQPKPILQKQSTFPQSSKDIPDRGAATDEKLQNFAIENTP
VCFSHNSSLSSLSDIDQENNNKENEPIKETEPPDSQGEPSKPQASGYAPKSFHVEDTPVCFSRNSSLSSL
SIDSEDDLLQECISSAMPKKKKPSRLKGDNEKHSPRNMGGILGEDLTLDLKDIQRPDSEHGLSPDSENFD
WKAIQEGANSIVSSLHQAAAAACLSRQASSDSDSILSLKSGISLGSPFHLTPDQEEKPFTSNKGPRILKP
GEKSTLETKKIESESKGIKGGKKVYKSLITGKVRSNSEISGQMKQPLQANMPSISRGRTMIHIPGVRNSS

SSTSPVSKKGPPLKTPASKSPSEGQTATTSPRGAKPSVKSELSPVARQTSQIGGSSKAPSRSGSRDSTPS
RPAQQPLSRPIQSPGRNSISPGRNGISPPNKLSQLPRTSSPSTASTKSSGSGKMSYTSPGRQMSQQNLTK
QTGLSKNASSIPRSESASKGLNQMNNGNGANKKVELSRMSSTKSSGSESDRSERPVLVRQSTFIKEAPSP
TLRRKLEESASFESLSPSSRPASPTRSQAQTPVLSPSLPDMSLSTHSSVQAGGWRKLPPNLSPTIEYNDG
RPAKRHDIARSHSESPSRLPINRSGTWKREHSKHSSSLPRVSTWRRTGSSSSILSASSESSEKAKSEDEK
HVNSISGTKQSKENQVSAKGTWRKIKENEFSPTNSTSQTVSSGATNGAESKTLIYQMAPAVSKTEDVWVR
IEDCPINNPRSGRSPTGNTPPVIDSVSEKANPNIKDSKDNQAKQNVGNGSVPMRTVGLENRLNSFIQVDA
PDQKGTEIKPGQNNPVPVSETNESSIVERTPFSSSSSSKHSSPSGTVAARVTPFNYNPSPRKSSADSTSA
RPSQIPTPVNNNTKKRDSKTDSTESSGTQSPKRHSGSYLVTSV

# 1421 – 1556 aa.

## Hotspot 1 1421aa-1465aa

AA Change: S1421Vfs*52 (1/4,767)

chr5:g.112839851delC

WT peptide:

SSVQSEPCSGMVSGIISPSDLPDSPGQTMPPSRSKTPPPPPQTAQTKREVPKNKAPTAEKRESGPKQAAVNAA

Mutant:

SSVQSEPCSGMVSGIISPVIFQIALDKPCHQAEVKHLHHLLKQLKPSEKYLKIKHLLLKRERVDLSKLQ-

AA Change: S1421Vfs*52 (1/4,767)

chr5:g.112839855delA

WT peptide:

SSVQSEPCSGMVSGIISPSDLPDSPGQTMPPSRSKTPPPPPQTAQTKREVPKNKAPTAEKRESGPKQAAVNAA

Mutant:

SSVQSEPCSGMVSGIISPVIFQIALDKPCHQAEVKHLHHLLKQLKPSEKYLKIKHLLLKRERVDLSKLQ-

AA Change: S1421Mfs*52 (1/4,767)

chr5:g.112839856delG

WT peptide:

SSVQSEPCSGMVSGIISPSDLPDSPGQTMPPSRSKTPPPPPQTAQTKREVPKNKAPTAEKRESGPKQAAVNAA

Mutant:

SSVQSEPCSGMVSGIISPMIFQIALDKPCHQAEVKHLHHLLKQLKPSEKYLKIKHLLLKRERVDLSKLQ-

AA Change: P1424Qfs*49 (1/4,767)

chr5:g.112839864delC

WT peptide:

SSVQSEPCSGMVSGIISPSDLPDSPGQTMPPSRSKTPPPPPQTAQTKREVPKNKAPTAEKRESGPKQAAVNAA

Mutant:

SSVQSEPCSGMVSGIISPSDLQIALDKPCHQAEVKHLHHLLKQLKPSEKYLKIKHLLLKRERVDLSKLQ-

AA Change: APC P1427Lfs*46 (2/4,767)

chr5:g.112839872delC

WT peptide:

SSVQSEPCSGMVSGIISPSDLPDSPGQTMPPSRSKTPPPPPQTAQTKREVPKNKAPTAEKRESGPKQAAVNAA

Mutant:

SSVQSEPCSGMVSGIISPSDLPDSLDKPCHQAEVKHLHHLLKQLKPSEKYLKIKHLLLKRERVDLSKLQ-

AA Change: APC Q1429Dfs*45 (1/4,767)

chr5:g.112839875_112839876insGG

WT peptide:

SSVQSEPCSGMVSGIISPSDLPDSPGQTMPPSRSKTPPPPPQTAQTKREVPKNKAPTAEKRESGPKQAAVNAA

Mutant:

SSVQSEPCSGMVSGIISPSDLPDSPGDKPCHQAEVKHLHHLLKQLKPSEKYLKIKHLLLKRERVDLSKLQ-

AA Change: APC M1431Cfs*42 (1/4,767)

chr5:g.112839883delC

WT peptide:

SSVQSEPCSGMVSGIISPSDLPDSPGQTMPPSRSKTPPPPPQTAQTKREVPKNKAPTAEKRESGPKQAAVNAA

Mutant:

SSVQSEPCSGMVSGIISPSDLPDSPGQTCHQAEVKHLHHLLKQLKPSEKYLKIKHLLLKRERVDLSKLQ-

AA Change: APC R1435Lfs*31 (1/4,767)

chr5:g.112839896delCAGAAGTAAAACACCTCCACCA

WT peptide:

SSVQSEPCSGMVSGIISPSDLPDSPGQTMPPSRSKTPPPPPQTAQTKREVPKNKAPTAEKRESGPKQAAVNAA

Mutant:

SSVQSEPCSGMVSGIISPSDLPDSPGQTMPPSLLKQLKPSEKYLKIKHLLLKRERVDLSKLQ-

AA Change: APC R1435Efs*38 (1/4,767)

chr5:g.112839897delA

WT peptide:

SSVQSEPCSGMVSGIISPSDLPDSPGQTMPPSRSKTPPPPPQTAQTKREVPKNKAPTAEKRESGPKQAAVNAA

Mutant:

SSVQSEPCSGMVSGIISPSDLPDSPGQTMPPSEVKHLHHLLKQLKPSEKYLKIKHLLLKRERVDLSKLQ-

AA Change: APC R1435Kfs*38 (1/4,767)

chr5:g.112839898delG

WT peptide:

SSVQSEPCSGMVSGIISPSDLPDSPGQTMPPSRSKTPPPPPQTAQTKREVPKNKAPTAEKRESGPKQAAVNAA

Mutant:

SSVQSEPCSGMVSGIISPSDLPDSPGQTMPPSKVKHLHHLLKQLKPSEKYLKIKHLLLKRERVDLSKLQ-

AA Change: APC S1436Rfs*38 (1/4,767)

chr5:g.112839899_112839900insAG

WT peptide:

SSVQSEPCSGMVSGIISPSDLPDSPGQTMPPSRSKTPPPPPQTAQTKREVPKNKAPTAEKRESGPKQAAVNAA

Mutant:

SSVQSEPCSGMVSGIISPSDLPDSPGQTMPPSKEVKHLHHLLKQLKPSEKYLKIKHLLLKRERVDLSKLQ-

AA Change: APC S1436Vfs*37 (1/4,767)

chr5:g.112839899delA

WT peptide:

SSVQSEPCSGMVSGIISPSDLPDSPGQTMPPSRSKTPPPPPQTAQTKREVPKNKAPTAEKRESGPKQAAVNAA

Mutant:

SSVQSEPCSGMVSGIISPSDLPDSPGQTMPPSRVKHLHHLLKQLKPSEKYLKIKHLLLKRERVDLSKLQ-

AA Change: APC S1436Rfs*37 (2/4,767)

chr5:g.112839902delT

WT peptide:

SSVQSEPCSGMVSGIISPSDLPDSPGQTMPPSRSKTPPPPPQTAQTKREVPKNKAPTAEKRESGPKQAAVNAA

Mutant:

SSVQSEPCSGMVSGIISPSDLPDSPGQTMPPSRRKHLHHLLKQLKPSEKYLKIKHLLLKRERVDLSKLQ-

AA Change: APC P1439Lfs*34 (3/4,767)

chr5:g.112839908delA

WT peptide:

SSVQSEPCSGMVSGIISPSDLPDSPGQTMPPSRSKTPPPPPQTAQTKREVPKNKAPTAEKRESGPKQAAVNAA

Mutant:

SSVQSEPCSGMVSGIISPSDLPDSPGQTMPPSRSKTLHHLLKQLKPSEKYLKIKHLLLKRERVDLSKLQ-

AA Change: P1439Lfs*34 (2/4,767)

chr5:g.112839909delC

WT peptide:

SSVQSEPCSGMVSGIISPSDLPDSPGQTMPPSRSKTPPPPPQTAQTKREVPKNKAPTAEKRESGPKQAAVNAA

Mutant:

SSVQSEPCSGMVSGIISPSDLPDSPGQTMPPSRSKTLHHLLKQLKPSEKYLKIKHLLLKRERVDLSKLQ-

P1442Lfs*31 (2/4,767)

chr5:g.112839918delC

WT peptide:

SSVQSEPCSGMVSGIISPSDLPDSPGQTMPPSRSKTPPPPPQTAQTKREVPKNKAPTAEKRESGPKQAAVNAA

Mutant:

SSVQSEPCSGMVSGIISPSDLPDSPGQTMPPSRSKTPPPLLKQLKPSEKYLKIKHLLLKRERVDLSKLQ-

P1443Lfs*30 (1/4,767)

chr5:g.112839920delT

WT peptide:

SSVQSEPCSGMVSGIISPSDLPDSPGQTMPPSRSKTPPPPPQTAQTKREVPKNKAPTAEKRESGPKQAAVNAA

Mutant:

SSVQSEPCSGMVSGIISPSDLPDSPGQTMPPSRSKTPPPPLKQLKPSEKYLKIKHLLLKRERVDLSKLQ-

T1445Kfs*28 (1/4,767)

chr5:g.112839928delC

WT peptide:

SSVQSEPCSGMVSGIISPSDLPDSPGQTMPPSRSKTPPPPPQTAQTKREVPKNKAPTAEKRESGPKQAAVNAA

Mutant:

SSVQSEPCSGMVSGIISPSDLPDSPGQTMPPSRSKTPPPPPQKLKPSEKYLKIKHLLLKRERVDLSKLQ-

AA Change: A1446Vfs*27

chr5:g.112839931delC (1/4,767)

WT peptide:

SSVQSEPCSGMVSGIISPSDLPDSPGQTMPPSRSKTPPPPPQTAQTKREVPKNKAPTAEKRESGPKQAAVNAA

Mutant:

SSVQSEPCSGMVSGIISPSDLPDSPGQTMPPSRSKTPPPPPQTVKPSEKYLKIKHLLLKRERVDLSKLQ-

chr5:g.112839929delA

AA Change: A1446Lfs*27 (1/4,767)

WT peptide:

SSVQSEPCSGMVSGIISPSDLPDSPGQTMPPSRSKTPPPPPQTAQTKREVPKNKAPTAEKRESGPKQAAVNAA

Mutant:

SSVQSEPCSGMVSGIISPSDLPDSPGQTMPPSRSKTPPPPPQTLKPSEKYLKIKHLLLKRERVDLSKLQ-

AA Change: Q1447Tfs*27 (1/4,767)

chr5:g.112839932_112839933insAC

WT peptide:

SSVQSEPCSGMVSGIISPSDLPDSPGQTMPPSRSKTPPPPPQTAQTKREVPKNKAPTAEKRESGPKQAAVNAA

Mutant:

SSVQSEPCSGMVSGIISPSDLPDSPGQTMPPSRSKTPPPPPQTATKPSEKYLKIKHLLLKRERVDLSKLQ-

AA Change: APC P1453Dfs*7 (1/4,767)

chr5:g.112839947delAGTACCTAAAAATAAAGCACCTACTGCTGAAAAGAGAGAG

WT peptide:

SSVQSEPCSGMVSGIISPSDLPDSPGQTMPPSRSKTPPPPPQTAQTKREVPKNKAPTAEKRESGPKQAAVNAA

Mutant:

SSVQSEPCSGMVSGIISPSDLPDSPGQTMPPSRSKTPPPPPQTAQTKREVDLSKLQ-

AA Change: Q1447Sfs*24 (1/4,767)

chr5:g.112839931delCTCAAAC

WT peptide:

SSVQSEPCSGMVSGIISPSDLPDSPGQTMPPSRSKTPPPPPQTAQTKREVPKNKAPTAEKRESGPKQAAVNAA

Mutant:

SSVQSEPCSGMVSGIISPSDLPDSPGQTMPPSRSKTPPPPPQTASEKYLKIKHLLLKRERVDLSKLQ-

AA Change: P1453Lfs*20 (1/4,767)

chr5:g.112839950delA

WT peptide:

SSVQSEPCSGMVSGIISPSDLPDSPGQTMPPSRSKTPPPPPQTAQTKREVPKNKAPTAEKRESGPKQAAVNAA

Mutant:

SSVQSEPCSGMVSGIISPSDLPDSPGQTMPPSRSKTPPPPPQTAQTKREVLKIKHLLLKRERVDLSKLQ-

AA Change: P1453Lfs*20 (1/4,767)

chr5:g.112839951delC

WT peptide:

SSVQSEPCSGMVSGIISPSDLPDSPGQTMPPSRSKTPPPPPQTAQTKREVPKNKAPTAEKRESGPKQAAVNAA

Mutant:

SSVQSEPCSGMVSGIISPSDLPDSPGQTMPPSRSKTPPPPPQTAQTKREVLKIKHLLLKRERVDLSKLQ-

AA Change: K1454Lfs*20 (1/4,767)

chr5:g.112839952_112839953insTT

WT peptide:

SSVQSEPCSGMVSGIISPSDLPDSPGQTMPPSRSKTPPPPPQTAQTKREVPKNKAPTAEKRESGPKQAAVNAA

Mutant:

SSVQSEPCSGMVSGIISPSDLPDSPGQTMPPSRSKTPPPPPQTAQTKREVPLKIKHLLLKRERVDLSKLQ-

AA Change: N1455Ifs*18 (3/4,767)

chr5:g.112839954delA

WT peptide:

SSVQSEPCSGMVSGIISPSDLPDSPGQTMPPSRSKTPPPPPQTAQTKREVPKNKAPTAEKRESGPKQAAVNAA

Mutant:

SSVQSEPCSGMVSGIISPSDLPDSPGQTMPPSRSKTPPPPPQTAQTKREVPKIKHLLLKRERVDLSKLQ-

AA Change: APC K1456Hfs*16 (1/4,767)

chr5:g.112839960delAAAG

WT peptide:

SSVQSEPCSGMVSGIISPSDLPDSPGQTMPPSRSKTPPPPPQTAQTKREVPKNKAPTAEKRESGPKQAAVNAA

Mutant:

SSVQSEPCSGMVSGIISPSDLPDSPGQTMPPSRSKTPPPPPQTAQTKREVPKNHLLLKRERVDLSKLQ-

E1464Vfs*8 (4/4,767)

chr5:g.112839979delAGAG

WT peptide:
SSVQSEPCSGMVSGIISPSDLPDSPGQTMPPSRSKTPPPPPQTAQTKREVPKNKAPTAEKRESGPKQAAVNAA

Mutant:

SSVQSEPCSGMVSGIISPSDLPDSPGQTMPPSRSKTPPPPPQTAQTKREVPKNKAPTAEKRVDLSKLQ-

S1465Rfs*9 (6/4,767)

chr5:g.112839978_112839979insAG

WT peptide:

SSVQSEPCSGMVSGIISPSDLPDSPGQTMPPSRSKTPPPPPQTAQTKREVPKNKAPTAEKRESGPKQAAVNAA

Mutant:

SSVQSEPCSGMVSGIISPSDLPDSPGQTMPPSRSKTPPPPPQTAQTKREVPKNKAPTAEKRERVDLSKLQ-

# Hotspot 2 1481aa-1495aa

: V1481Mfs*23 (1/4,767)

chr5:g.112840030delTCCAGGTTCT

WT peptide:

SGPKQAAVNAAVQRVQVLPDADTLLHFATESTPDGFSCSSSLSALSLDEPFIQKDV

New mutant:

SGPKQAAVNAAVQRVQMLILYYILPRKVLQMDFLVHPA-

: D1486Ifs*21 (4/4,767)

chr5:g.112840049delT

WT peptide: SGPKQAAVNAAVQRVQVLPDADTLLHFATESTPDGFSCSSSLSALSLDEPFIQKDV

New mutant:

SGPKQAAVNAAVQRVQVLPDAILYYILPRKVLQMDFLVHPA-

: T1487Yfs*18 (1/4,767)

chr5:g.112840051delATACTTT

WT peptide: SGPKQAAVNAAVQRVQVLPDADTLLHFATESTPDGFSCSSSLSALSLDEPFIQKDV

New mutant:

SGPKQAAVNAAVQRVQVLPDADYILPRKVLQMDFLVHPA-

AA Change: T1487Ifs*20 (1/4,767)

chr5:g.112840054delC

WT peptide: SGPKQAAVNAAVQRVQVLPDADTLLHFATESTPDGFSCSSSLSALSLDEPFIQKDV

New mutant:

SGPKQAAVNAAVQRVQVLPDADIYYILPRKVLQMDFLVHPA-

L1488Yfs*19 (7 / 4,767)

chr5:g.112840055delT

WT peptide:

SGPKQAAVNAAVQRVQVLPDADTLLHFATESTPDGFSCSSSLSALSLDEPFIQKDV

New mutant (Strong binding)

SGPKQAAVNAAVQRVQVLPDADTYYILPRKVLQMDFLVHPA-

AA Change: L1488Ffs*17 (1/4,767)

DNA Change: chr5:g.112840057delTATTACA

WT peptide:

SGPKQAAVNAAVQRVQVLPDADTLLHFATESTPDGFSCSSSLSALSLDEPFIQKDV

New mutant:

SGPKQAAVNAAVQRVQVLPDADTFLPRKVLQMDFLVHPA-

AA Change: L1488Kfs*13 (1/4,767)

chr5:g.112840056delTTATTACATTTTGCCACGG

WT peptide:

SGPKQAAVNAAVQRVQVLPDADTLLHFATESTPDGFSCSSSLSALSLDEPFIQKDV

New mutant:

SGPKQAAVNAAVQRVQVLPDADTKVLQMDFLVHPA-

AA Change: L1488Yfs*20 (1/4,767)

chr5:g.112840055_112840056insTA

WT peptide:

SGPKQAAVNAAVQRVQVLPDADTLLHFATESTPDGFSCSSSLSALSLDEPFIQKDV

New mutant:

SGPKQAAVNAAVQRVQVLPDADTYYYILPRKVLQMDFLVHPA-

AA Change: L1489Yfs*19 (1/4,767)

chr5:g.112840056_112840057insTA

WT peptide:

SGPKQAAVNAAVQRVQVLPDADTLLHFATESTPDGFSCSSSLSALSLDEPFIQKDV

New mutant:

SGPKQAAVNAAVQRVQVLPDADTLYYILPRKVLQMDFLVHPA-

L1489Yfs*18: (3/4,767)

chr5:g.112840059delT
WT peptide:

SGPKQAAVNAAVQRVQVLPDADTLLHFATESTPDGFSCSSSLSALSLDEPFIQKDV

New mutant:

SGPKQAAVNAAVQRVQVLPDADTLYILPRKVLQMDFLVHPA-

AA Change: H1490Yfs*18 (1/4,767)

chr5:g.112840059_112840060insTA

WT peptide:

SGPKQAAVNAAVQRVQVLPDADTLLHFATESTPDGFSCSSSLSALSLDEPFIQKDV

New mutant:

SGPKQAAVNAAVQRVQVLPDADTLLYILPRKVLQMDFLVHPA-

AA Change: H1490Lfs*17 (1/4,767)

chr5:g.112840063delA

WT peptide:

SGPKQAAVNAAVQRVQVLPDADTLLHFATESTPDGFSCSSSLSALSLDEPFIQKDV

New mutant:

SGPKQAAVNAAVQRVQVLPDADTLLLLPRKVLQMDFLVHPA-

F1491Lfs*16 (3/4,767)

chr5:g.112840064delT

WT peptide:

SGPKQAAVNAAVQRVQVLPDADTLLHFATESTPDGFSCSSSLSALSLDEPFIQKDV

New mutant:

SGPKQAAVNAAVQRVQVLPDADTLLHLPRKVLQMDFLVHPA-

A1492Pfs*15 (1/4,767)

chr5:g.112840068delG

WT peptide:

SGPKQAAVNAAVQRVQVLPDADTLLHFATESTPDGFSCSSSLSALSLDEPFIQKDV

New mutant:

SGPKQAAVNAAVQRVQVLPDADTLLHFPRKVLQMDFLVHPA-

: T1493Rfs*14 (4 / 4,767)

chr5:g.112840069delC

WT peptide:

SGPKQAAVNAAVQRVQVLPDADTLLHFATESTPDGFSCSSSLSALSLDEPFIQKDV

new mutant (strong binding)

SGPKQAAVNAAVQRVQVL PDADTLLHFARKVLQMDFLVHPA-

E1494Kfs*13 (2/4,767)

chr5:g.112840073delG

WT peptide:

SGPKQAAVNAAVQRVQVLPDADTLLHFATESTPDGFSCSSSLSALSLDEPFIQKDV

new mutant:

SGPKQAAVNAAVQRVQVLPDADTLLHFATKVLQMDFLVHPA-

S1495Vfs*12 (3/4,767)

chr5:g.112840075delA

WT peptide:

SGPKQAAVNAAVQRVQVLPDADTLLHFATESTPDGFSCSSSLSALSLDEPFIQKDV

new mutant:

SGPKQAAVNAAVQRVQVLPDADTLLHFATEVLQMDFLVHPA-

# Hotspot 3 1552aa-1556aa

E1552Gfs*6 (3/4,767)

chr5:g.112840247delAG

WT peptide:

QPKESNENQEKEAEKTIDSEKD

Mutant peptide:

QPKESNENQEKGRKNY-

T1556Nfs*3  (47 / 4,767)

chr5:g.112840254_112840255insA

WT peptide:

QPKESNENQEKEAEKTIDSEKD

New mutant

QPKESNENQEKEAEKNY –
