# Supplementary material for: Identification, design, and in vivo proof of concept of a shared APC neoantigen delivered via a self-amplifying RNA containing virus-like nanoparticle for cancer vaccination
Source: Front Immunol. 2026 Jun 4;17:1810178. doi: 10.3389/fimmu.2026.1810178 (PMC13275407; doi:10.3389/fimmu.2026.1810178)
Supplement: Supplementary file 4 [file DataSheet4.pdf]

Supplemental Table 4:

Flow Cytometry Metrics, fold-change increase in percent positivity for IFN-γ + CD3 in PBMC population

Well	Sample	IFN+CD3	Fold Change over Unstim.
A2.fcs	Unstim	3.68 %	N/A
A3.fcs	Pos. Ctrl.	14.6 %	3.967
A4.fcs	HS1-1	7.28 %	1.978
A5.fcs	HS1-2	7.10 %	1.929
A6.fcs	HS1-3	3.78 %	1.027
A7.fcs	HS1-4	4.05 %	1.101
A8.fcs	HS1-5	3.72 %	1.011
A9.fcs	HS1-6	4.89 %	1.329
A10.fcs	HS1-7	5.33 %	1.448
A11.fcs	HS1-8	3.07 %	0.834
B2.fcs	P/I	32.9 %	8.940
B4.fcs	HS1-9	6.85 %	1.861
B5.fcs	HS1-10	6.53 %	1.774
B6.fcs	HS1-11	6.21 %	1.688
B7.fcs	HS1-12	7.77 %	2.111
B8.fcs	HS1-13	6.37 %	1.731
B9.fcs	HS2-1	7.16 %	1.946
B10.fcs	HS2-2	5.55 %	1.508
B11.fcs	HS2-3	4.35 %	1.182
C3.fcs	HS2-4	4.06 %	1.103
C4.fcs	HS2-5	5.76 %	1.565
C5.fcs	HS3-1	6.58 %	1.788
C6.fcs	SB-1	3.75 %	1.019
C7.fcs	SB-2	3.86 %	1.049
C8.fcs	SB-3	6.37 %	1.731
C9.fcs	SB-4	6.25 %	1.698
C10.fcs	SB-5	2.39 %	0.649
C11.fcs	SB-6	5.16 %	1.402
D2.fcs	SB-16	4.59 %	1.247
D3.fcs	SB-7	6.10 %	1.658
D4.fcs	SB-8	6.09 %	1.655
D5.fcs	SB-9	6.29 %	1.709
D6.fcs	SB-10	4.69 %	1.274
D7.fcs	SB-11	3.84 %	1.043
D8.fcs	SB-12	6.21 %	1.688
D9.fcs	SB-13	6.36 %	1.728
D10.fcs	SB-14	3.29 %	0.894
D11.fcs	SB-15	5.50 %	1.495
