# Supplementary material for: Identification, design, and in vivo proof of concept of a shared APC neoantigen delivered via a self-amplifying RNA containing virus-like nanoparticle for cancer vaccination
Source: Front Immunol. 2026 Jun 4;17:1810178. doi: 10.3389/fimmu.2026.1810178 (PMC13275407; doi:10.3389/fimmu.2026.1810178)
Supplement: Supplementary file 5 [file DataSheet5.pdf]

Supplementary Material

1 Supplementary Tables

1. APCmut Peptide Fragment MHC Binding Bioinformatics 1 (Attached Separately)
2. APCmut Peptide Fragment MHC Binding Bioinformatics 2 (Attached Separately)
3. Shared APC Frameshift Mutation Table (Attached Separately)
4. Flow Cytometry Metrics, fold-change increase in percent positivity for IFN-γ + CD3 in PBMC population (Attached Separately)

2 Supplementary Figures

A

Plasmid ID	Features	Remarks
pCBIO-1146	pCMV-VEEV-empty	No GOI
pCBIO-1289	pCMV-VEEV-mouseAPCWT-SP1	Mouse WT APC (947-1558aa) with signal peptide 1
pCBIO-1263	pCMV-VEEV-chimericAPC-SP2	Mouse APC (947-1558aa) with mutations and signal peptide 2
pCBIO-1264	pCMV-VEEV-chimericAPC-SP1	Mouse APC (947-1560aa) with mutations and signal peptide 1
pCBIO-1265	pCMV-VEEV-mutAPC-SP3	Human APC (947-1560aa) with mutations and signal peptide 3
pCBIO-1266	pCMV-VEEV-mutAPC-SP4	Human APC (947-1560aa) with mutations and signal peptide 4
pCBIO-1267	pCMV-VEEV-mutAPC-SP5	Human APC (947-1560aa) with mutations and signal peptide 5
pCBIO-1268	pCMV-VEEV-mutAPC-SP6	Human APC (947-1560aa) with mutations and signal peptide 6

B

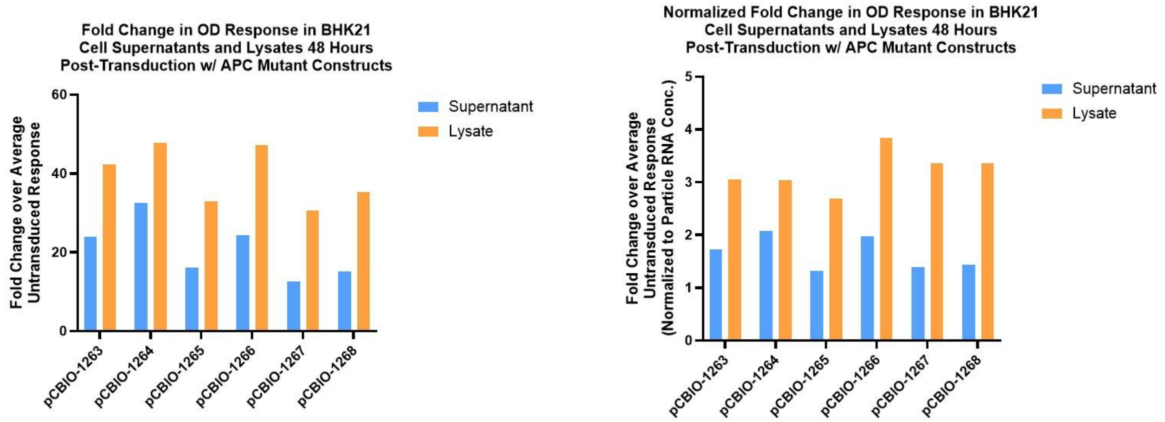

C

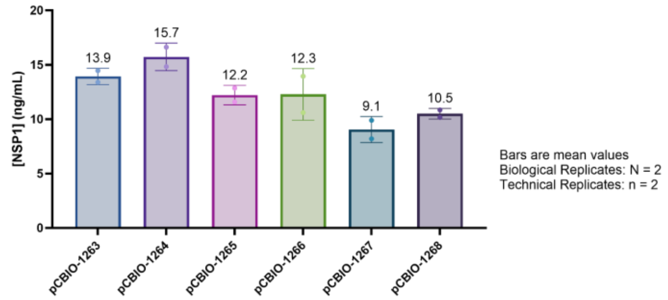

Supplemental Figure 1: Design of APC mutant replicons featuring multiple different secretory peptides. A) Constructs pCBIO-1263 and pCBIO-1264 express WT mouse APC (with human APC mutation regions) to limit immune responses against the WT human APC regions during in vivo mouse experiments. This differs from previous constructs, which expressed WT human APC. Constructs pCBIO-1265 through pCBIO-1268 express a larger region of WT human APC in their sequence to facilitate improved in vitro and in vivo detectability. The nanoparticle constructs were transduced into BHK21 cells to determine in vitro APC mutant expression. B) 48 hours after transduction, cell supernatants and lysates were harvested and analyzed via sandwich ELISA, revealing significant expression across all 6 constructs relative to untransduced control cells. The fold change increase in APC mutant expression was then normalized to the replicon RNA concentration transduced into each cell suspension for improved comparison between constructs. C) Particle RNA concentration was assessed via RNA isolation assay (Thermo Fisher) for all nanoparticle constructs used in this study. Particle RNA concentrations were the basis of the reported dosing concentration for in vivo experiments.

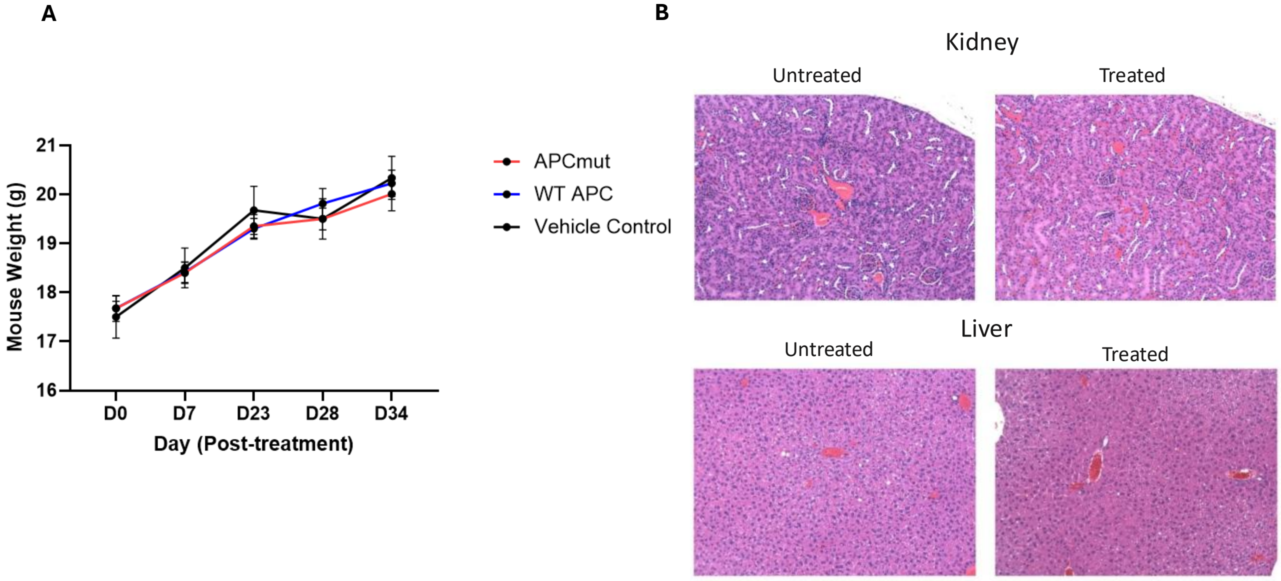

Supplemental Figure 1: No significant indication of toxicity observed in mice following IM dosing of chimeric nanoparticle constructs. A) Mouse subject weights were routinely measured during performance of in vivo APCmut vaccine dosing study. No significant difference was observed in subject weight between each arm, and no arms exhibited decreasing body weights indicative of toxicity. B) Previous IM dosing studies of the chimeric nanoparticle construct featured end-of-study H&E histopathology staining of kidney and liver tissues. No significant histopathological differences were observed between untreated versus nanoparticle-treated study arms, indicating no detectable tissue toxicity.
